# Tris-Azo Triangular Paraphenylenes: Synthesis and Reversible Interconversion into Radial π-Conjugated Macrocycles

**DOI:** 10.1021/jacs.4c00657

**Published:** 2024-04-03

**Authors:** Tomohito Ide, Wei-Ci Huang, Masaki Horie

**Affiliations:** † Department of Chemical Science and Engineering, National Institute of Technology, Tokyo College, 1220-2 Kunugida-machi, Hachioji-shi, Tokyo 193-0997, Japan; ‡ Department of Chemical Engineering, National Tsing Hua University, 101, Sec. 2, Kuang-Fu Road, Hsinchu, 30013, Taiwan

## Abstract

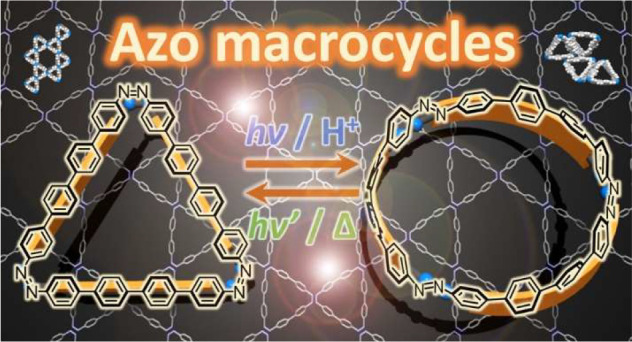

We report the synthesis of cycloparaphenylene derivatives featuring tris-azo groups. The smaller derivative, [3]cycloazobenzene, adopts a triangular all-*cis* form and exhibits thermally and photochemically stable characteristics due to significant ring strain as well as symmetric Kagome-patterned crystal packing. In contrast, the as-synthesized [3]cycloazobenzene with three biphenylene bridges adopts a triangular all-*cis* form, which undergoes photoinduced isomerization, leading to a photostationary state. Interestingly, the addition of an excess of acid selectively leads to the formation of an all-*trans* form. DFT calculations reveal that the interconversion from a triangular to a circular shape correlates with an increase in HOMO and a decrease in LUMO, characteristics intrinsic to radial π-conjugated systems.

π-Conjugated macrocycles have garnered significant attention due to their unique structures and properties.^1−3^ These materials, known for their rigidity, are often referred to as shape-persistent macrocycles.^1^ Several macrocycles have been synthesized containing *ortho*- or *meta*-phenylene units, forming planar structures and π-conjugated systems. Over the past decade, substantial research has focused on a new class of π-conjugated macrocycles consisting of benzene linked at the *para* position, yielding a curved structure characteristic of a radial π-conjugated system. Typical examples of these are cycloparaphenylenes (CPPs) and related nanohoops.^2^ These molecules possess a radial π-conjugate system with a shallow highest occupied molecular orbital (HOMO) level and a deeper lowest unoccupied molecular orbital (LUMO) level compared with linear oligomers, resulting in a narrower HOMO–LUMO gap.^4^

Azobenzene, a well-known stimuli-responsive molecule, undergoes *trans*-to-*cis* isomerization upon photoirradiation, a process reversible by subsequent photoirradiation, heat, or acid.^5^ While there are reports on macrocycles containing azobenzene units, π-conjugated macrocycles with three or more azobenzene units remain unexplored. The first macrocycle containing three *trans* azo groups linked with orthophenylene was synthesized by Dreiding et al. (Figure 1a).^6^ Wegner et al. attempted photoinduced isomerization, but the macrocycle did not adopt a *cis* form.^7^ Subsequently, they reported a macrocycle with three azobenzene units by *meta*-linkage, which exhibits photoisomerization (Figure 1b).^8^ Additionally, Tamaoki et al. reported π-conjugated macrocycles containing two to four azobenzene units linked with orthophenylene that exhibit photoisomerization (Figure 1c).^9^

**Figure 1:**
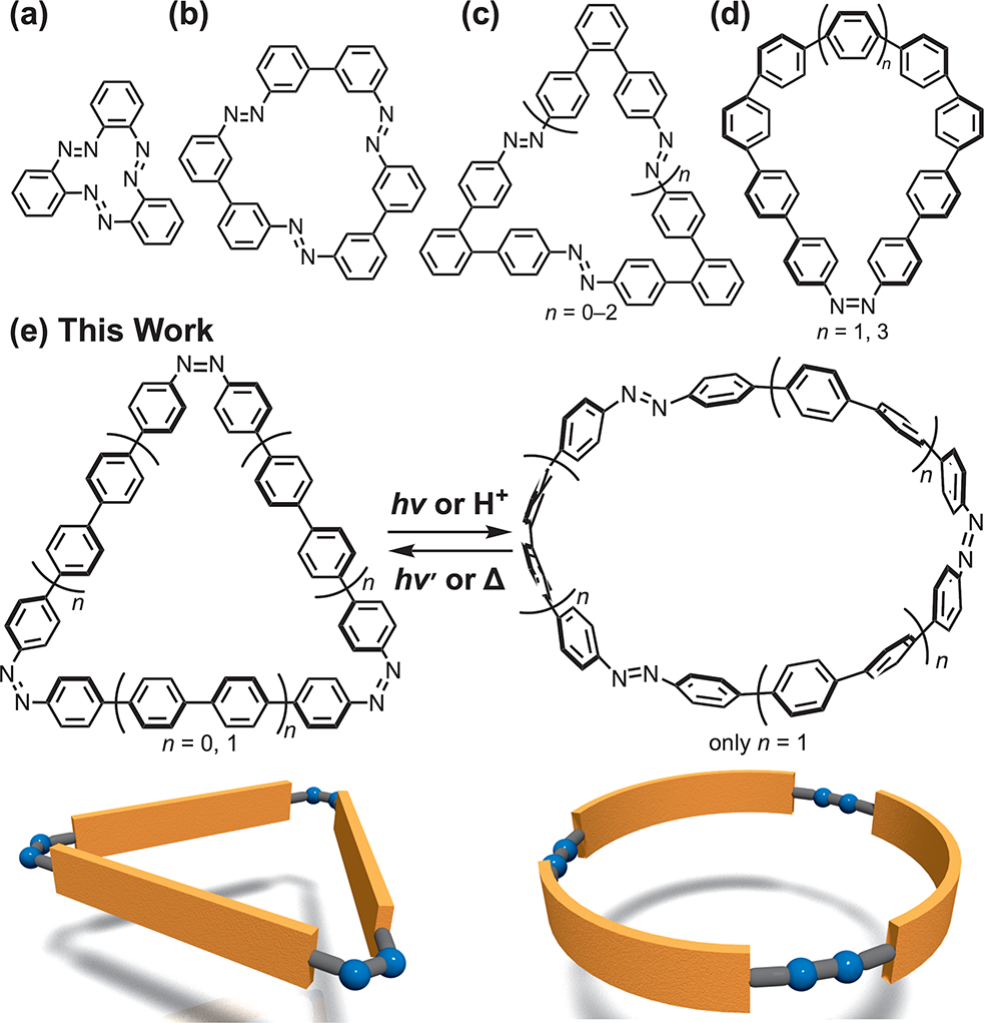
Examples of π-conjugated azo-containing macrocycles: (a) *ortho*-linked three azo groups, (b) *meta*-linked azobenzene, (c) azobenzenes linked with *ortho*-phenylenes, (d) CPPs with one azo group. (e) Tris-azo macrocycles in this work.

Jasti et al. reported CPP incorporating a single azobenzene unit, *cis*-azo[9]CPP, which does not exhibit photoisomerization (Figure 1d).^10^ They further described *cis*-azo[11]CPP, which is capable of photoisomerizable ring size alteration. CPPs with two or more azo groups would exhibit more pronounced shape changes in the macrocycle; however, exploration of such CPPs has been primarily theoretical, focusing on their potential host function.^11^ This study synthesizes CPP derivatives with three azo groups, one of which demonstrates reversible interconversion between triangular and radial π-conjugated macrocycle shapes *via cis*-*trans* isomerization, triggered by photo- or acid-stimuli (Figure 1e).

The target CPPs containing three azo groups were successfully synthesized using the macrocyclic gold complex approach (Scheme 1 and Figures S1–S17).^12^ The synthesis of the smaller ring [3]cycloazobenzene (*n* = 0, **[3]CAB-0**), comprising three azobenzenes, proceeded in two steps starting from azobenzene derivative **1** with boronic acid pinacol ester groups. Initially, **1** reacted with Au_2_Cl_2_(dcpm) **3** (dcpm: bis(dicyclohexylphosphino)methane) in the presence of Cs_2_CO_3_ at 50 °C, forming the macrocyclic gold complex intermediate **4**. Oxidative chlorination with 3 equiv of PhICl_2_, conducted in the absence of light, provided **[3]CAB-0** (45% yield). The larger ring [3]cycloazobenzene (*n* = 1, **[3]CAB-1**) was synthesized following a similar procedure using azobiphenyl derivative **2** instead of **1**, resulting in a 43% yield.

**Scheme 1:**
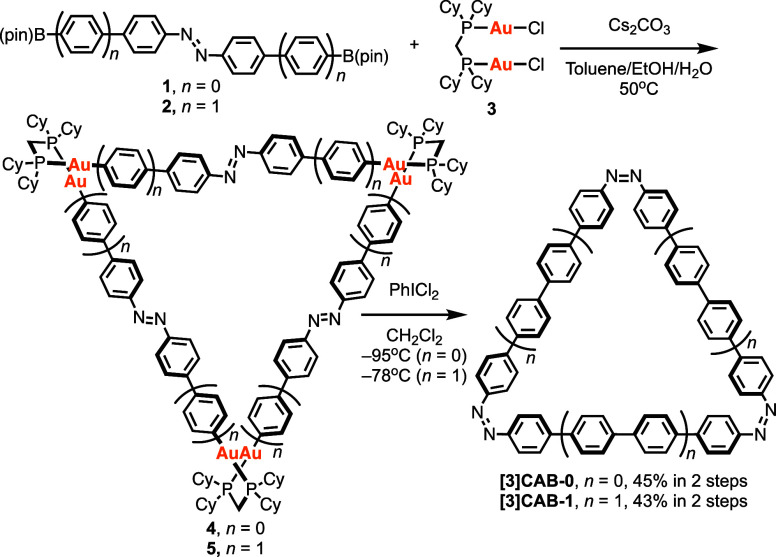
Synthetic Route to Tris-azo Macrocycles

Single crystals of the respective tris-azo macrocycles were obtained via the vapor diffusion of cyclohexane into a chloroform solution. Single-crystal X-ray crystallography of **[3]CAB-0** (CCDC: 2321643) and **[3]CAB-1** (CCDC: 2321642) revealed all-*cis* forms, leading to triangular molecular structures with respective void heights of 9.9 and 17.5 Å (Figure 2, Figures S18–S21, and Tables S1 and S2). Notably, **[3]CAB-0** exhibits Kagome-patterned packing, forming a large 21.3 Å void, with this symmetric packing being stabilized by intermolecular CH···N hydrogen bonds (Figures S22 and S23). Conversely, **[3]CAB-1** has dense packing without an additional large void. Furthermore, disordered phenylene groups in **[3]CAB-1** imply enhanced mobility, suggesting a trend toward isomerization.

**Figure 2:**
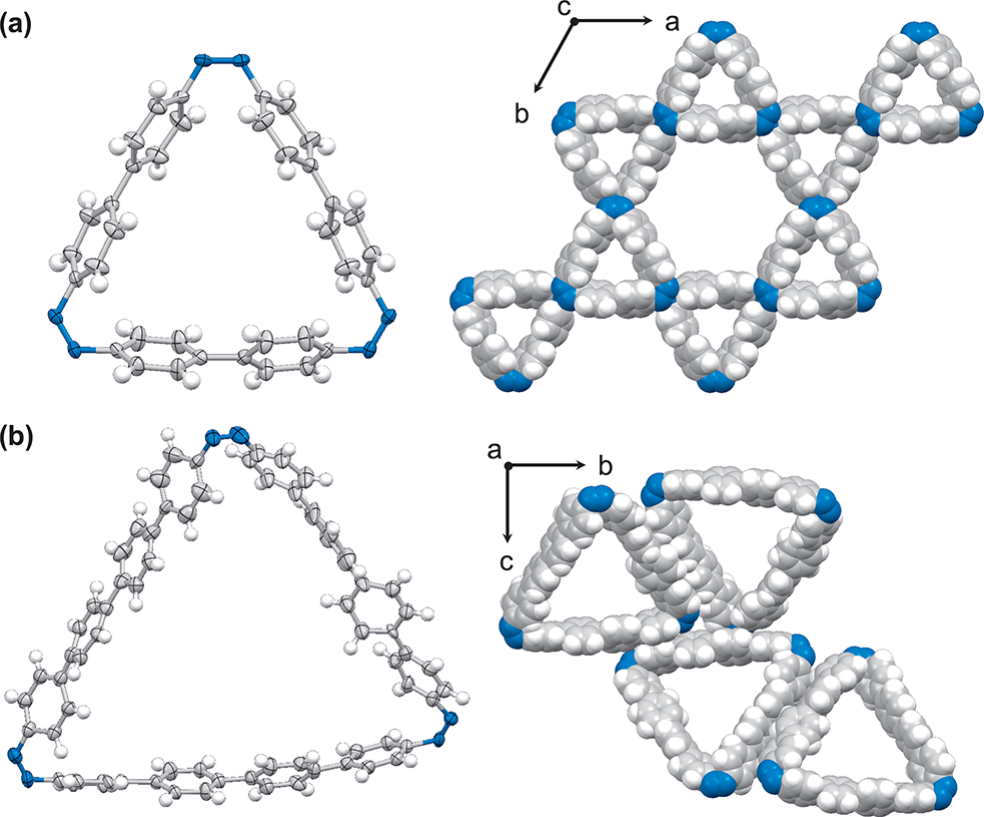
ORTEP drawing (30% probability) and packing diagram (space-filling model) of (a) **[3]CAB-0** and (b) **[3]CAB-1**. Gray, blue, and white balls represent carbon, nitrogen, and hydrogen atoms, respectively. Solvents were removed using the SQUEEZE procedure for both crystals, and disordered molecules were omitted for **[3]CAB-1**.

Similar to our molecules, previously reported CPPs containing a single azo group were obtained solely as *cis* isomers from *trans*-4,4′-dibromoazobenzene.^10^ Additionally, a macrocyclic stilbene tetramer was found to adopt a partial *cis* form, though it was synthesized from a *trans*-stilbene derivative.^13^ This *cis* formation resulted from ring strain in the intermediate macrocyclic Pt-complex. Strained phenylene bonds formation via the Au(III)-intermediate has been reported previously.^12a^ In this work, the Au(III) azodiphenylene intermediates would exhibit high ring strain during the elimination of macrocyclic Au-complexes, causing the bending of *trans* azobenzene and thus leading to the formation of *cis* azobenzene.

The UV–vis absorption spectrum of all-*cis***[3]CAB-0** in 1,2-dichloroethane shows a peak at 429 nm, attributed to the azo group. Photoirradiation of the **[3]CAB-0** solution did not induce significant changes in the absorption band (Figure S24). Consequently, our investigation shifted to larger **[3]CAB-1**. Its UV–vis spectrum, recorded immediately after dissolution in 1,2-dichloroethane, exhibits an absorption band at 446 nm (Figure 3a). Irradiation of the solution at 254 nm demonstrated the significant change, showing an increase in absorption at 383 nm and a decrease at 329 nm within 15 min, indicative of *cis*-to-*trans* photoisomerization. Irradiation at 365 or 445 nm, as well as exposure to ambient light, resulted in different levels of photostationary states (Figures S25–S30). These spectral changes could be repeated (Figure S31).

**Figure 3:**
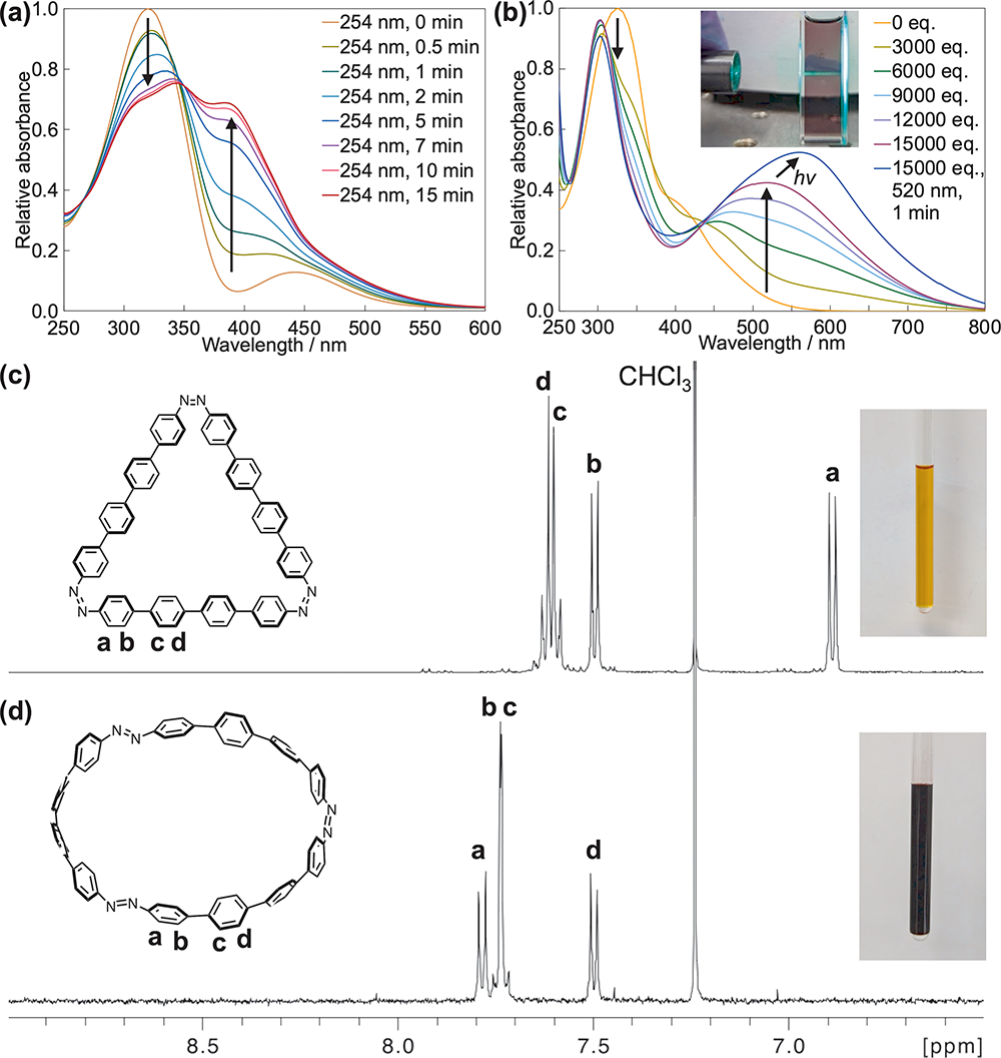
(a) UV–vis spectra of **[3]CAB-1** (0.02 mM, 1,2-dichloroethane, 25 °C) following irradiation at 254 nm. (b) UV–vis spectra of **[3]CAB-1** (0.02 mM, 1,2-dichloroethane, 25 °C) after the addition of a large excess of CF_3_COOH, with a photo of the solution irradiated at 520 nm and containing 15000 equiv of acid depicted. ^1^H NMR spectra of **[3]CAB-1** (500 MHz, 27 °C) (c) in CDCl_3_ and (d) in CDCl_3_ containing 1.2 M CF_3_COOH.

The all-*cis* forms of both macrocycles were further confirmed by ^1^H NMR spectroscopy, revealing a signal at 6.70 ppm for **[3]CAB-0** (Figure S4) and 6.89 ppm for **[3]CAB-1** (Figure 3c). Upon irradiation of the **[3]CAB-1** solution at 254 nm, additional small signals appeared in the range 7.7–7.9 ppm, attributable to the *trans* azo groups (Figure S50, Tables S30–S37).

Given that *cis*-azobenzene is known to rapidly isomerize to its *trans* form upon acid addition,^5a−5d^ we explored the acid response of tris-azo macrocycles (Figures S40–S53). In a CDCl_3_ solution, **[3]CAB-1** exhibited an orange color, which shifted to deep purple upon the addition of 1.2 M CF_3_COOH (approximately 1300 equiv). The ^1^H NMR spectrum with CF_3_COOH (Figure 3d) shows a shift from 6.89 to 7.78 ppm, confirming the formation of the all-*trans* state consistent with a previous report.^5d^ According to the report, the protonated state (−N=NH^+^−) of azobenzene was not observed with CF_3_COOH within the NMR time scale, and an excess of strong acids like triflic acid (CF_3_SO_3_H) is needed to observe the protonated state of azobenzene. Conversely, a CDCl_3_ solution of **[3]CAB-0** exhibited a minor color change after adding 1.2 M CF_3_COOH, with the ^1^H NMR spectrum confirming only the presence of the *cis* form (Figure S51).

UV–vis spectra were recorded for **[3]CAB-1** in 1,2-dichloroethane at the photostationary state under ambient light at 25 °C, with varying concentrations of CF_3_COOH (Figure 3b). Increasing the CF_3_COOH concentration caused the absorption shoulder around 400 nm to shift to longer wavelengths, and a new absorption peak emerged at 519 nm. Excess acid is necessary due to the weak basicity of azobenzene (Figure S64). In comparison, the addition of CF_3_COOH to a 1,2-dichloroethane solution of **[3]CAB-0** did not significantly alter the absorption wavelength to around 430 nm (Figure S40). Interestingly, when the acidic solution of **[3]CAB-1** was irradiated at 520 nm, the solution color promptly changed from purple to blue at the line irradiated by the 520 nm laser, accompanied by a shift in absorption maxima from 519 to 565 nm (Figure 3b, its inset photo, and Figure S44). The color and spectrum rapidly reverted to their original state once the light source was removed (Figure S45). Irradiation at 365 and 405 nm also induced a bathochromic shift of about 20 nm (Figure S44). However, irradiation at 254 nm resulted in the disappearance of the absorption band at 519 nm, suggesting compound decomposition (Figure S46).

The reversibility of **[3]CAB-1** was investigated by introducing triethylamine to a 1,2-dichloroethane solution containing 15000 equiv of CF_3_COOH. Upon adding only 6000 equiv of triethylamine, less than half of the CF_3_COOH amount, the spectrum largely recovered (Figure S48). Subsequent readdition of CF_3_COOH caused the absorption at 450 nm to reappear. Methanol also facilitated deprotonation, reverting the spectrum to the all-*cis***[3]CAB-1** upon adding 30700 equiv (Figure S49).

Thermal isomerization of **[3]CAB-1** was also studied (Figures S32–S39). Heating to 60 °C led to a decrease in the absorption band around 400 nm, indicating an increase in the all-*cis* form (Figure S37). In contrast, **[3]CAB-0** exhibited no significant change from its as-synthesized *cis* form (Figure S24), thus confirming its stability as a photo-, acid-, and thermally stable *cis*-azobenzene. Similar examples of stable *cis*-azobenzene include azo[9]CPP^10^ and methylene-bridged azobenzene macrocyclic dimers.^14^

The difficulty in inducing the stimuli-responsive isomerization of **[3]CAB-0** is largely attributed to its ring strain. This was quantified using DFT calculations based on the reaction enthalpy of the homodesmotic reaction (Table S4). The calculations (ωB97M-V^15^/def2-QZVP^16^//r^2^SCAN-3c,^17^ using ORCA^18^) revealed the ring strain energies for all-*cis*, *cis*-*cis*-*trans*, *cis*-*trans*-*trans*, and all-*trans***[3]CAB-0** to be 34.4, 52.2, 62.6, and 76.3 kcal mol^–1^, respectively (Figure S54). In comparison, **[3]CAB-1** showed lower strain energies of 34.7, 40.6, 40.7, and 42.0 kcal mol^–1^, respectively (Figure 4a and Figure S55). Notably, the ring strain in **[3]CAB-0** increased by 17.8 kcal mol^–1^ with the inclusion of one *trans* azo group. However, the difference in ring strain in **[3]CAB-1** was reduced to 5.9–7.3 kcal mol^–1^ when transitioning from one to all-*trans* form due to its larger macrocyclic size. The relative Gibbs energy is also within a range of 5.5–7.1 kcal mol^–1^, indicating that the slight energy differences in **[3]CAB-1** facilitate isomerization under external stimuli such as photoirradiation and acids (Figure 4a). Additionally, relative Gibbs energies for the double-protonated states of **[3]CAB-1** were estimated, with the double-protonated all-*trans* state being the most stable (Figure 4b and Figure S64).

**Figure 4:**
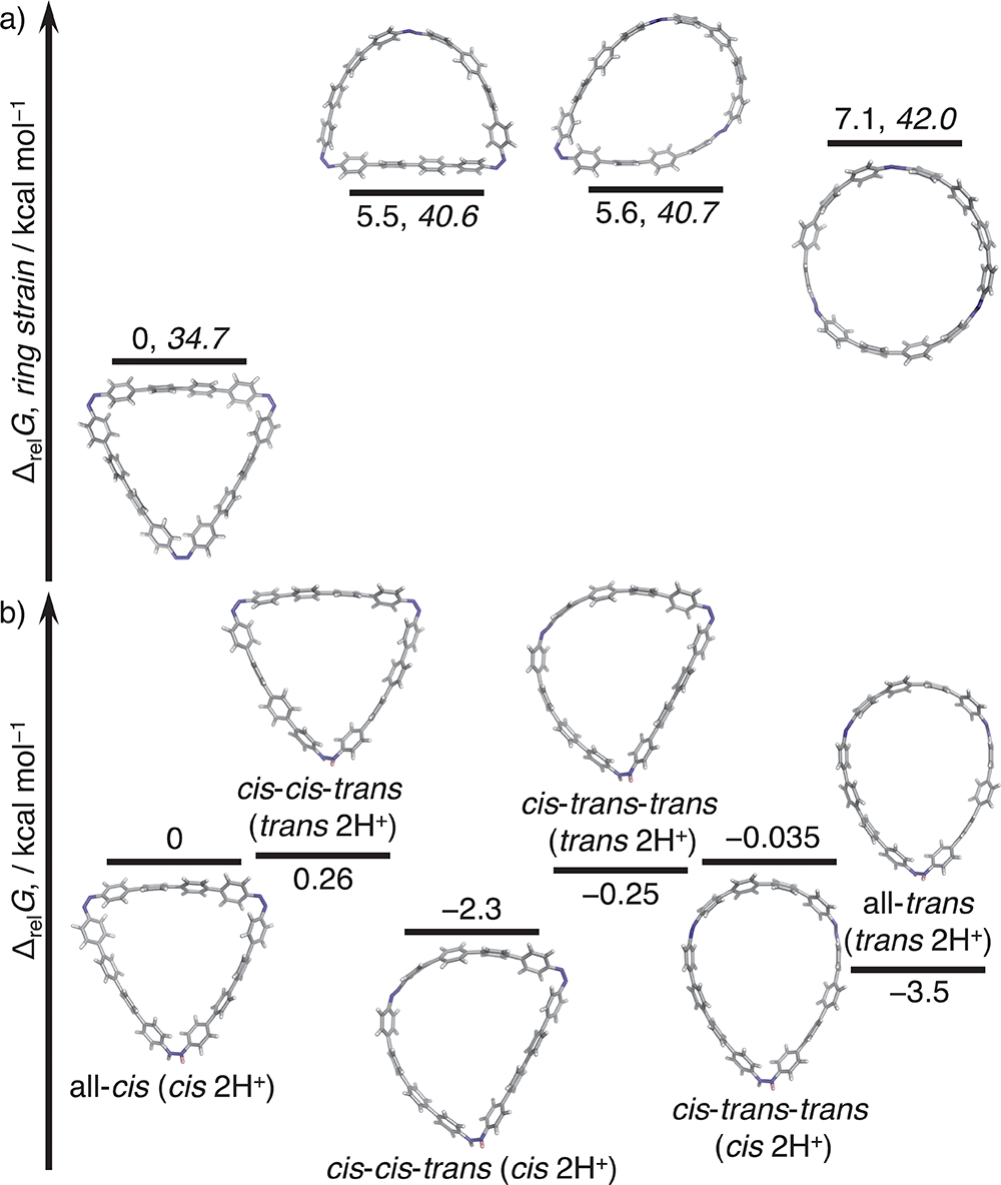
Relative Gibbs energies and ring strains for (a) neutral and (b) double-protonated **[3]CAB-1**.

Under acidic conditions, the tris-azo macrocycles exhibit absorption bands at significantly longer wavelengths in the UV–vis spectra compared to those of cycloparaphenylenes,^4^ neutral azobenzene, and its cation.^5d^ TD(TDA)-DFT calculations (SOS-ωPBEPP86^19^/cc-pVDZ^20^//r^2^SCAN-3c) were conducted for the tris-azo macrocycles to predict their UV–vis absorption properties (Figures S56–S62 and Tables S6–S29). The results suggest that the protonated species of **[3]CAB-1** exhibit absorption at longer wavelengths. Thus, the observed UV–vis spectra comprise contributions from both neutral and protonated states. The calculated absorption spectra for both all-*cis* and all-*trans* forms demonstrate a shift toward shorter wavelengths with an increase in the number of protons, with the all-*trans* form displaying absorption at longer wavelengths and higher intensity compared to the all-*cis* form. The reversible photochromism of **[3]CAB-1** is attributed to both deprotonation in excited states and isomerization from the remaining *cis* form to the *trans* form. As photochromism is not observed in **[3]CAB-0** upon photoirradiation (Figure S42), isomerization is considered the main driver of the photochromic process.

Furthermore, the electronic structure of **[3]CAB-1**, determined by DFT (CAM-B3LYP^21^/def2-TZVP^16^//r^2^SCAN-3c) calculations, reveals that in the all-*trans* form, both HOMO and LUMO are degenerate, while in the all-*cis* form, they are nondegenerate (Figure 5 and Figure S63). Similar to previously reported CPPs, which demonstrated an increase in HOMO and decrease in LUMO due to radial π-conjugation,^4^ we observed a comparable trend in orbital energies for **[3]CAB-1** transitioning from triangular to radial π-conjugation. Accompanying the structural change from all-*cis* to all-*trans*, the HOMO increased by +0.17 eV, and the LUMO decreased by −0.44 eV, narrowing the HOMO–LUMO gap by 0.60 eV from 5.79 to 5.19 eV. The orbital shapes reveal that the all-*cis* form prominently includes a lone pair from the azo group in both HOMO and LUMO, while the all-*trans* form predominantly involves the π orbital, distributed on the exterior of the azo groups with the lone pair orbital located on the interior of the macrocycle.

**Figure 5:**
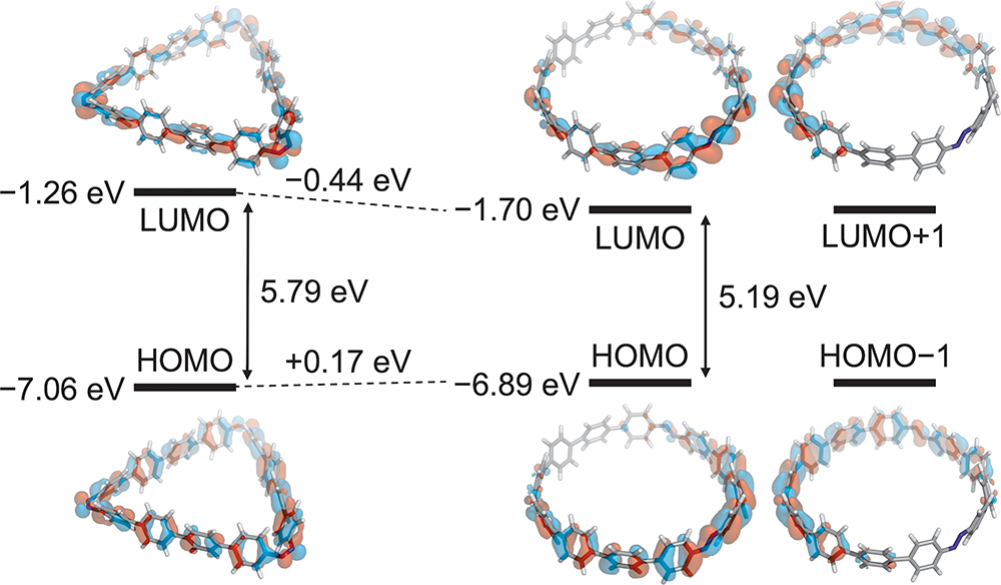
Frontier orbitals of all-*cis* and all-*trans***[3]CAB-1** (isosurface = 0.02).

In conclusion, we have successfully synthesized tris-azo macrocycles featuring three symmetrically placed azo groups within CPP skeletons. The smaller macrocycle maintains a persistent all-*cis* structure, whereas the larger macrocycle exhibits reversible *cis*-*trans* isomerization upon photoirradiation or acid/base addition. Rapid and reversible photochromism is also observed in an acidic solution of the larger macrocycle. This represents a first example of reversible interconversion between triangular and radial π-systems, distinct from reported irreversible conversions of acetylene-incorporated CPPs with azides.^22^ We anticipate that these switchable macrocycles could serve in photo- or chemically stimulated molecular machines and host–guest chemistry, leveraging their significant shape and electronic state changes.

## Supporting Information Available

Additional experimental details, materials and methods, ^1^H and ^13^C NMR, MS, and UV–vis spectra, crystallographic data, and computational details (PDF)
