# Supplementary material for: Tris-Azo Triangular Paraphenylenes: Synthesis and Reversible Interconversion into Radial π-Conjugated Macrocycles
Source: J Am Chem Soc. 2024 Apr 3;146(15):10246–50. doi: 10.1021/jacs.4c00657 (PMC11027133; doi:10.1021/jacs.4c00657)
Supplement: Supplementary file 1 — ja4c00657_si_001.pdf [file ja4c00657_si_001.pdf]

Supporting Information for

Tris-Azo Triangular Paraphenylenes: Synthesis and Reversible Interconversion into Radial π-Conjugated Macrocycles

Tomohito Ide*[1], Wei-Ci Huang[2], Masaki Horie*[2]

[1] *Department of Chemical Science and Engineering, National Institute of Technology, Tokyo College, 1220-2 Kunugida-machi, Hachioji-shi, Tokyo 193-0997, Japan*
E-mail: ide@tokyo-ct.ac.jp

[2] *Department of Chemical Engineering, National Tsing Hua University, 101, Sec. 2, Kuang-Fu Road, Hsinchu 30013, Taiwan*
E-mail: mhorie@mx.nthu.edu.tw

Table of Contents

S1. Materials and Methods 3

S1.1. Crystallography 3

S2. Synthesis and Characterization 4

S2.1. 4,4′-Dihydroxyazobenzene 4

S2.2. 4,4′-Bis(trifluorosulfonyloxy)azobenzene 5

S2.3. 4,4′-Bis(pinacolatoboryl)azobenzene **1** 6

S2.4. **[3]CAB-0** 7

S2.5. 4,4′-Bis(chlorophenyl)azobenzene 10

S2.6. 4,4′-Bis(pinacolatoborylphenyl)azobenzene **2** 13

S2.7. **[3]CAB-1** 16

S3. Crystallographic Studies 20

S4. Analysis of Intermolecular Interaction in Crystal of **[3]CAB-0** 24

S5. Photoisomerization Experiments 26

S6. Photo isomerization cycles 29

S7. Thermal Isomerization Experiments 30

S8. First-Order Analysis 33

S9. Acid Responses 35

S10. Photo Responses in Acidic Solution 36

S11. Heating in Acidic Solution 38

S12. Addition of the Bases to Acidic Solution 39

S13. ^{1}H NMR spectrum of **[3]CAB-1** after UV light irradiation 40

S14. ^{1}H NMR spectra of tris-azo macrocycles in $CDCl_3$ containing 1.2 M CF_3COOH 41

S15. Computational Details 44

S16. Ring Strain and Relative Gibbs Free Energy 44

S17. Transition Density of All-*cis* and All-*trans* **[3]CAB-1** 46

S18. Predicted UV-Vis Absorption Spectra 47

S19. Detailed Result of TDA-DFT Calculations 50

S20. Predicted ^{1}H NMR Chemical Shifts 73

S21. Frontier orbitals of **[3]CAB-1** 76

S22. Double-protonated State of **[3]CAB-1** 76

S23. Cartesian Coordinates of Optimized Geometry 79

S24. References 201

S1. Materials and Methods

All solvents and reagents were used without further purification. Au_2Cl_2(dcpm) and $PhICl_2$ were synthesized according to literature procedures.[1,2]

^{1}H and ^{13}C{^{1}H} NMR spectra were recorded on a Bruker AVANCE spectrometer (500 MHz), where chemical shifts were referenced to residual $CHCl_3$, CH_2Cl_2, or DMSO for ^{1}H (7.24, 5.30, and 2.49 ppm, respectively) and $CDCl_3$ for ^{13}C{^{1}H} (77.0 ppm) NMR spectra, respectively. FD-MS spectra were measured with a JEOL JMS-T200GC AccuTOF GCx system. IR spectra were measured using a Thermo Scientific Nicolet Summit FTIR spectrometer. UV-Vis absorption spectra were recorded on a JASCO V-630 spectrometer. Continuous-wave diode-pumped solid-state lasers emitting at 405 nm (TAN-YU, T405F200), 445 nm (TAN-YU, LSR445FP-1W), and 520 nm (TAN-YU, LSR520SD-FC-1W) were used to irradiate corresponding wavelengths. The power of the irradiated lasers at 405 nm, 445 nm, and 520 nm is 0.14 mW cm^{-2}, 1.6 mW cm^{-2}, and 0.64 mW cm^{-2}, respectively. A handheld UV lamp (BDTECK, BD405CAF) was used for irradiation at 254 nm and 365 nm. Thermogravimetry and differential thermal analysis (TG/DTA) were performed using SDT Q600 (TA instruments).

S1.1. Crystallography

Single-crystal X-ray diffraction data were collected using a Rigaku XtaLAB Synergy DW diffractometer equipped with monochromatic Cu Kα radiation (λ = 1.5406 Å) at 100 K. The collected data were analyzed using the SHELX program[3] and the OLEX2 software package.[4] The structure was solved by direct methods and refined by the full-matrix least-squares method on F^2 with anisotropic temperature factors for non-hydrogen atoms. **[3]CAB-0** and **[3]CAB-1** were resolved as two-component inversion twin. All the hydrogen atoms were located at the calculated positions and refined using the riding model. The disordered **[3]CAB-1** was refined using restrained distances, rigid bond restraints, and Uiso/Uaniso restraints and constraints. The disordered solvent molecules were removed using the SQUEEZE procedure for **[3]CAB-0** and **[3]CAB-1**.

S2. Synthesis and Characterization

S2.1. 4,4′-Dihydroxyazobenzene

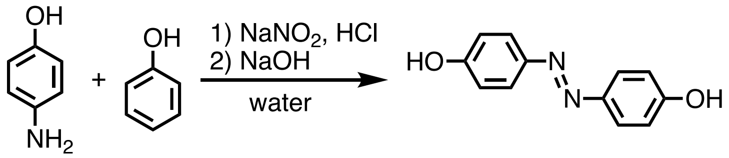

This compound was synthesized according to the literature.[5] 4-Aminophenol (9.82 g, 90.0 mmol), $NaNO_2$ (9.31 g, 135 mmol), and phenol (8.47 g, 90.0 mmol) were used. The title compound was obtained as a dark red powder (9.77 g, 51%). The observed ^{1}H NMR spectrum is in accordance with the literature.

^{1}H NMR (500 MHz, DMSO-d_6, 299 K): δ 10.09 (2H, s), 7.70 (4H, d, J = 8.6 Hz), 6.89 (4H, d, J = 8.6 Hz).

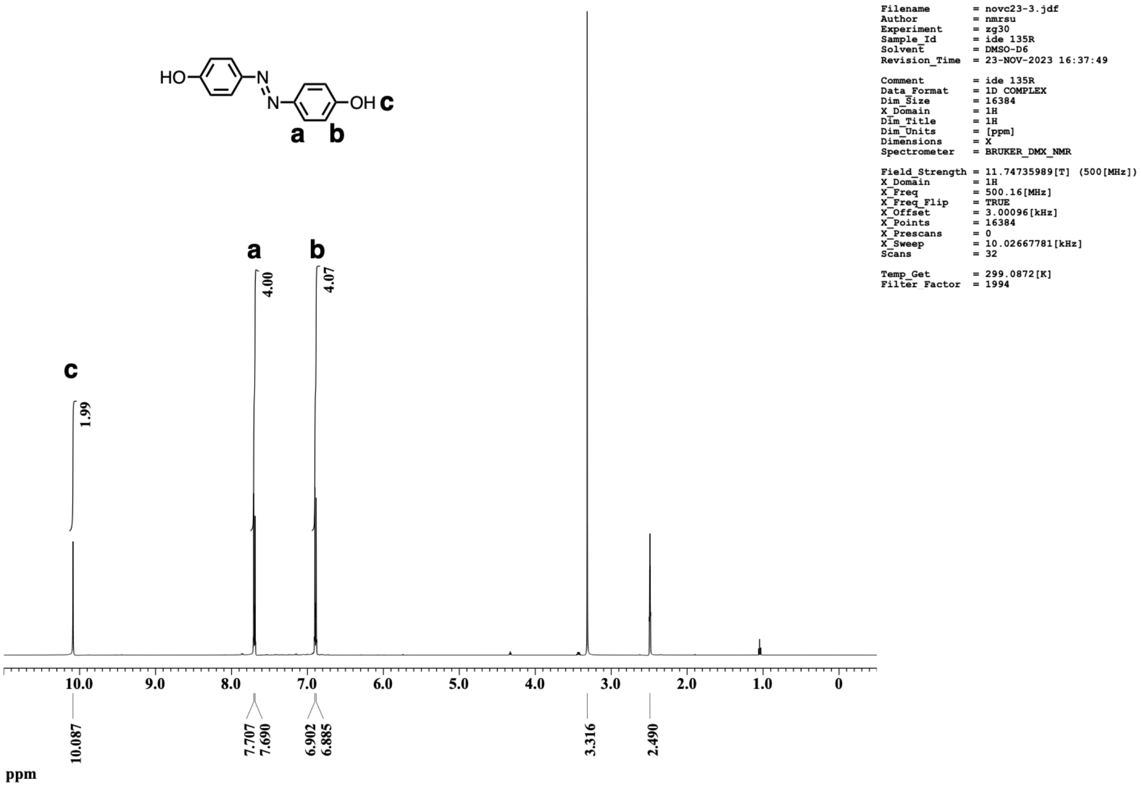

Figure S1. ^{1}H NMR spectrum (500 MHz, DMSO-d_6, 299 K) of 4,4′-dihydroxyazobenzene.

S2.2. 4,4′-Bis(trifluorosulfonyloxy)azobenzene

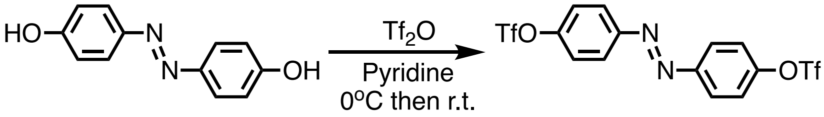

This compound was synthesized according to a literature procedure.[6] 4,4′-dihydroxyazobenzene (3.22 g, 15.0 mmol), Tf_2O (7.8 mL, 46 mmol), and pyridine (20 mL) were used. The title compound was obtained as an orange powder (3.25 g, 45%). The observed ^1H NMR spectrum is in accordance with the literature.

^1H NMR (500 MHz, DMSO-d_6, 299 K): δ 8.07 (4H, d, J = 8.5 Hz), 7.76 (4H, d, J = 8.5 Hz).

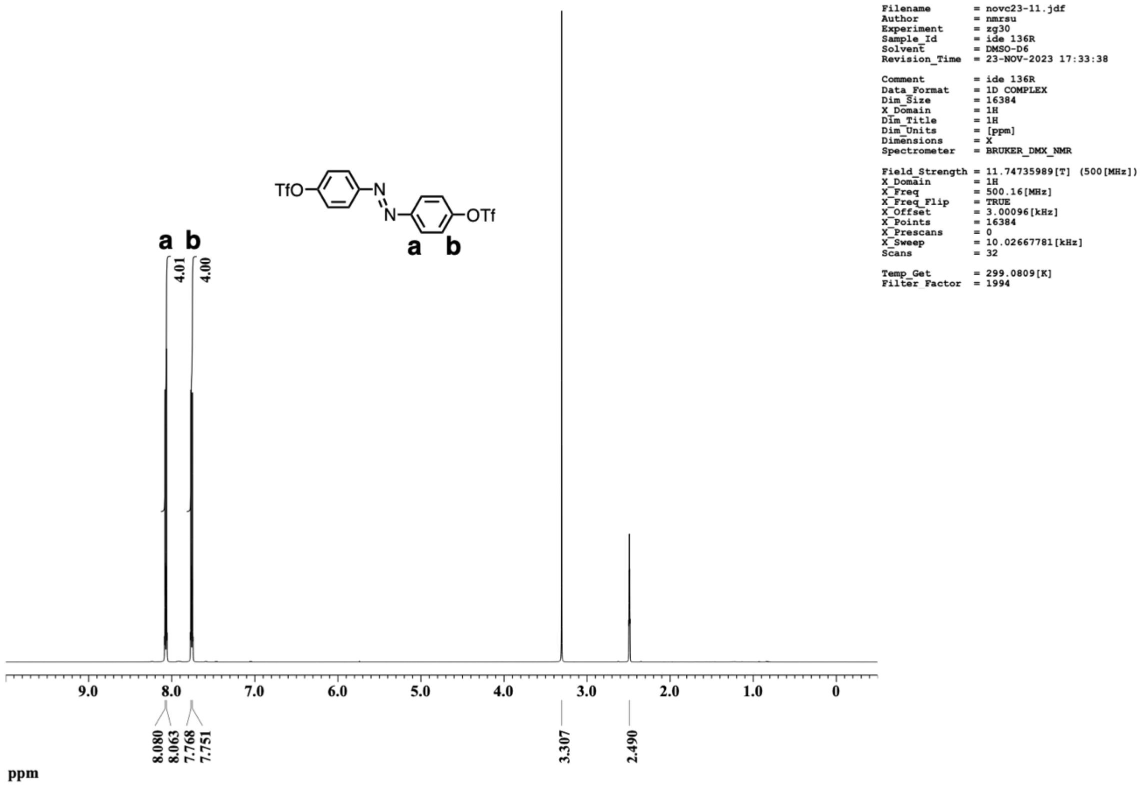

Figure S2. ^1H NMR spectrum (500 MHz, DMSO-d_6, 299 K) of 4,4′-bis(trifluorosulfonyloxy)azobenzene.

S2.3. 4,4′-Bis(pinacolatoboryl)azobenzene 1

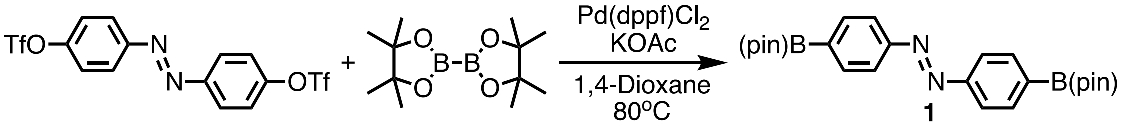

This compound was synthesized by a modified literature procedure.[6] 4,4′-bis(trifluorosulfonyloxy)azobenzene (1.24 g, 2.60 mmol), $B_2(pin)_2$ (1.66 g, 6.52 mmol), and KOAc (1.53 g, 15.6 mmol) were placed into a Schlenk tube and purged with N_2 gas. Vacuum-degassed 1,4-dioxane (13 mL) and $PdCl_2(dppf)$ (0.115 g, 0.158 mmol) were added to the Schlenk tube and stirred at 80 °C for 17 h. After cooling to room temperature, the reaction mixture was passed through a silica plug (eluent: Et_2O). The solution was concentrated, and the residual powder was washed twice with hexane. The residue was dried in vacuo to afford an orange powder (0.273 g, 24%). The observed ^{1}H NMR spectrum is in accordance with the literature.

^{1}H NMR (500 MHz, DMSO-d_6, 299 K): δ 7.94 (4H, d, J = 8.0 Hz), 7.89 (4H, d, J = 8.5 Hz), 1.35 (24H, s).

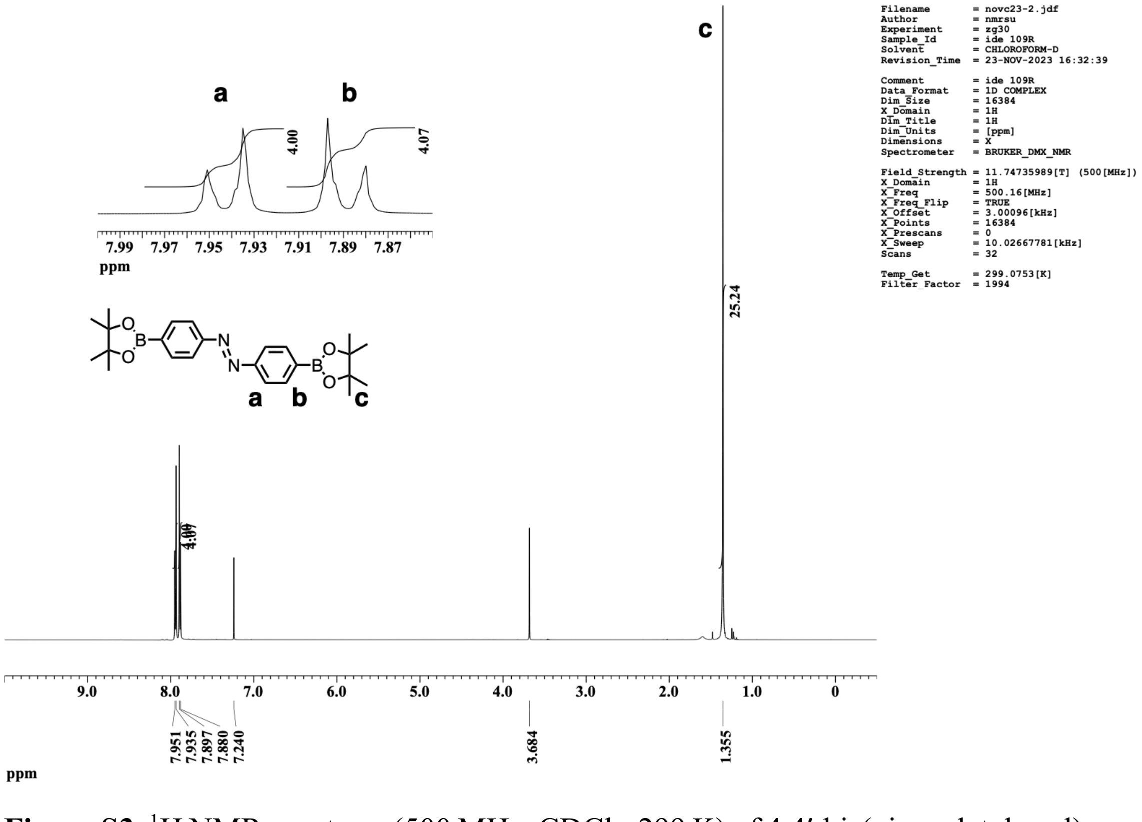

Figure S3. ^{1}H NMR spectrum (500 MHz, $CDCl_3$, 299 K) of 4,4′-bis(pinacolatoboryl)azobenzene.

S2.4. [3]CAB-0

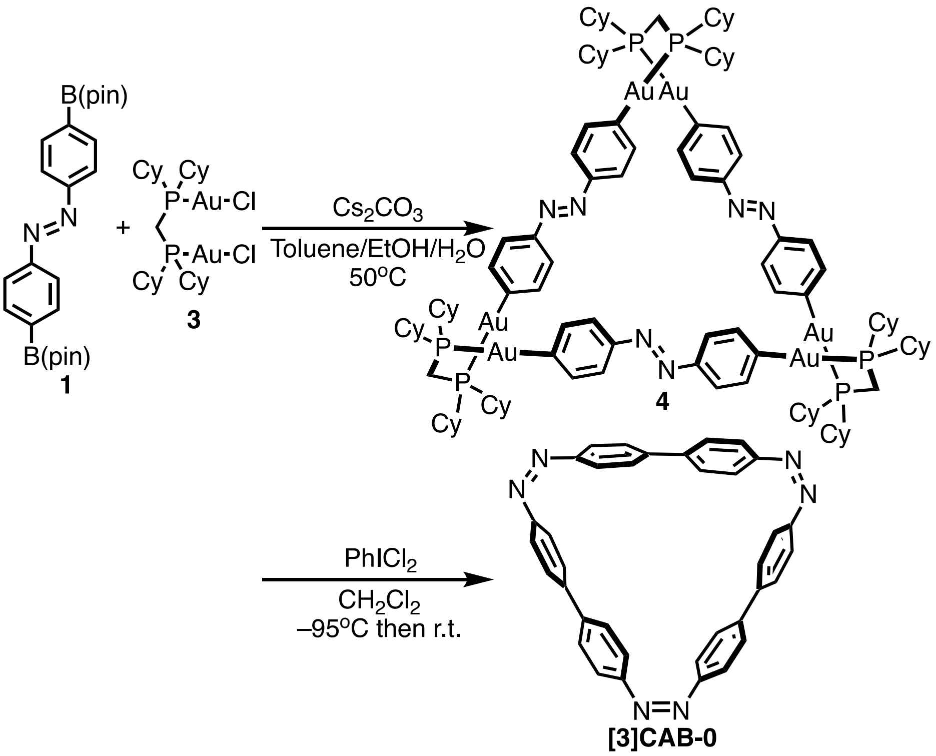

Toluene (8 mL), EtOH (2 mL), and H_2O (2 mL) were placed in a Schlenk tube, which was then vacuum degassed and filled with N_2 gas. 4,4′-Bis(pinacolatoboryl)azobenzene **1** (87.0 mg, 0.200 mmol), Au_2Cl_2(dcpm) **3** (175 mg, 0.200 mmol), and Cs_2CO_3 (392 mg, 1.20 mmol) were added to the Schlenk tube. The mixture was then stirred at 50 °C for 24 h. The resulting precipitate was collected by suction filtration and washed thrice with EtOH and H_2O. The residual solid was dried in vacuo to afford a macrocyclic gold complex **4** as an orange powder (160 mg). This compound was used in the next step without further purification.

The macrocyclic gold complex (124 mg, 0.0420 mmol) and dichloromethane (40 mL) were placed in a Schlenk tube and filled with N_2 gas. The mixture was then cooled to −95 °C in an acetone/liquid N_2 bath. After light shielding with aluminum foil, a dichloromethane (20 mL) solution of iodobenzene dichloride (34.6 mg, 0.126 mmol) was added dropwise to a Schlenk tube over 40 min. The reaction mixture was stirred and kept at −95 °C for 30 min, allowed to warm to room temperature, and stirred for 22 h covered with aluminum foil to avoid ambient light. After the reaction, the mixture was handled under ambient light. The resulting solution was concentrated under a vacuum. The obtained powder was dissolved in $CHCl_3$, and the insoluble fraction was removed by filtration. After concentration, silica gel column chromatography (eluent: $CHCl_3$/EtOAc = 10:1, v/v) of the residue gave **[3]CAB-0** (12.8 mg, 45% in 2 steps) as an orange solid.

^{1}H NMR (500 MHz, $CDCl_3$, 299 K): δ 7.34 (12H, d, J = 8.5 Hz, 3,5-N-C_6H_4), 6.70 (12H, d, J = 8.5 Hz, 2,6-N-C_6H_4).

$^{13}C\{^{1}H\}$ NMR (125 MHz, $CDCl_3$, 300 K): δ 154.3 (C_q), 137.7 (C_q), 126.5 (CH), 120.4 (CH).

FT-IR (KBr, cm^{-1}): 3056, 1729, 1710, 1599, 1494, 1480, 1394, 1004, 902, 854, 810, 738, 600.

HRMS (FD): calcd. for $C_{36}H_{24}N_6$: 540.2062, found: m/z 540.2075 ($[M]^{\cdot+}$).

Decomposition temperature: 350 °C.

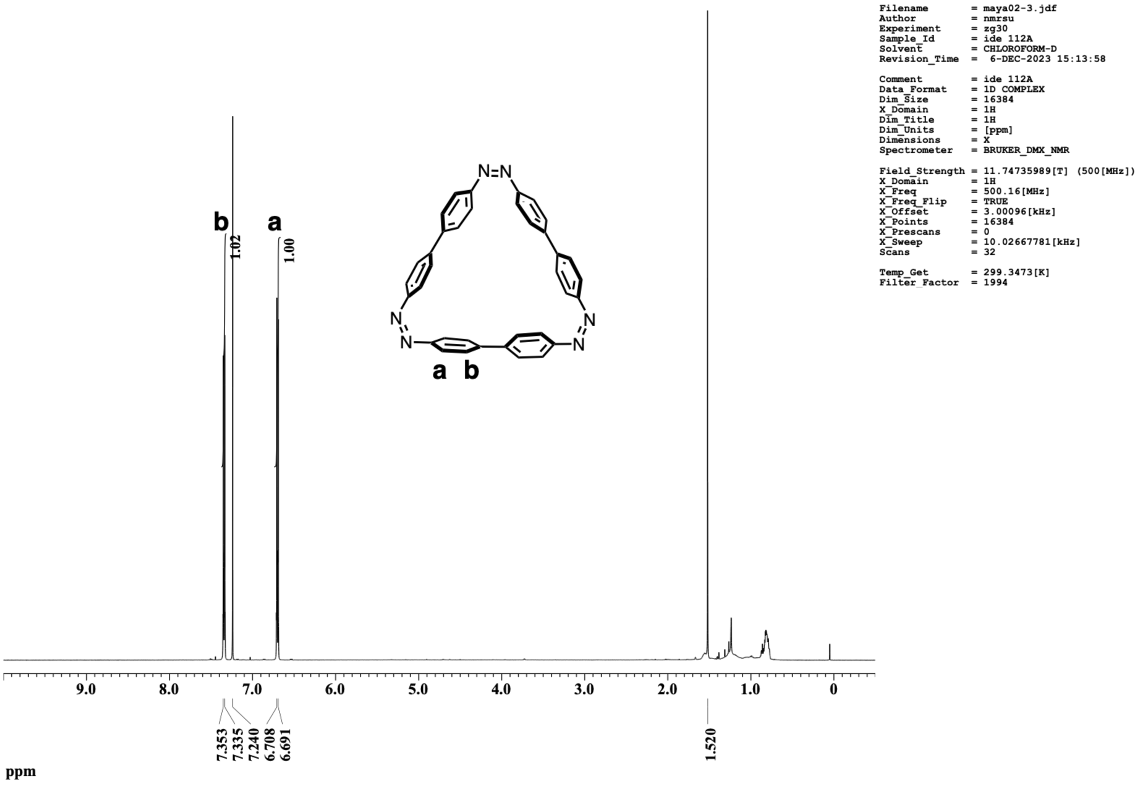

Figure S4. ^{1}H NMR spectrum (500 MHz, $CDCl_3$, 299 K) of **[3]CAB-0**.

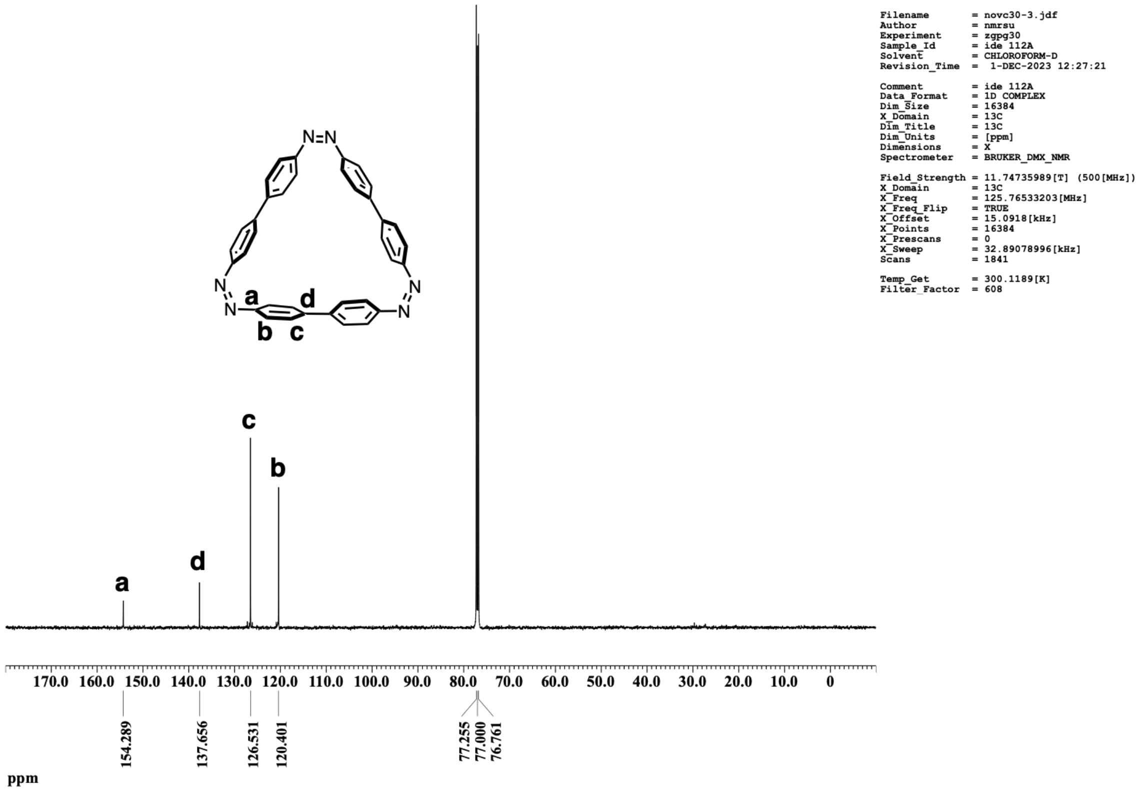

Figure S5. $^{13}C\{^{1}H\}$ NMR spectrum (125 MHz, $CDCl_3$, 300 K) of **[3]CAB-0**.

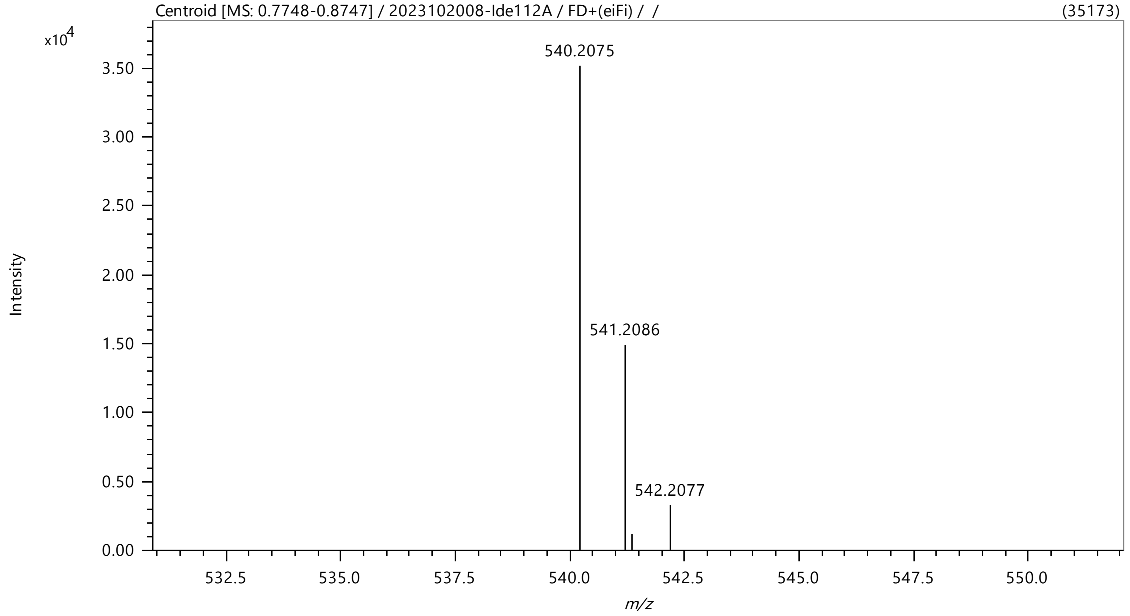

Figure S6. HR-FD-MS spectrum of **[3]CAB-0**.

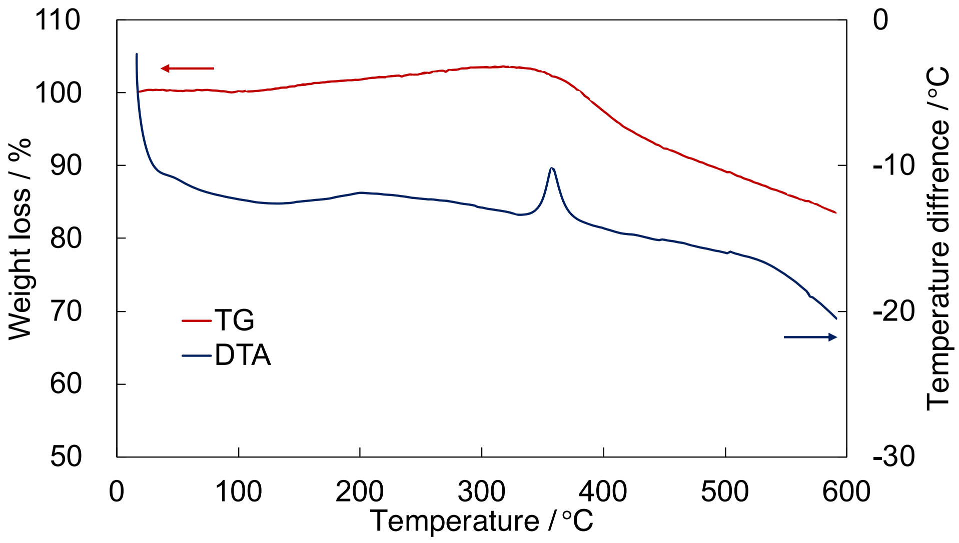

Figure S7. TG/DTA curves of **[3]CAB-0** (under N_2 flow, 10 °C min^{-1}). Decomposition temperature was found at 350 °C. Color of the sample was changed from orange to black after the measurement.

S2.5. 4,4′-Bis(chlorophenyl)azobenzene

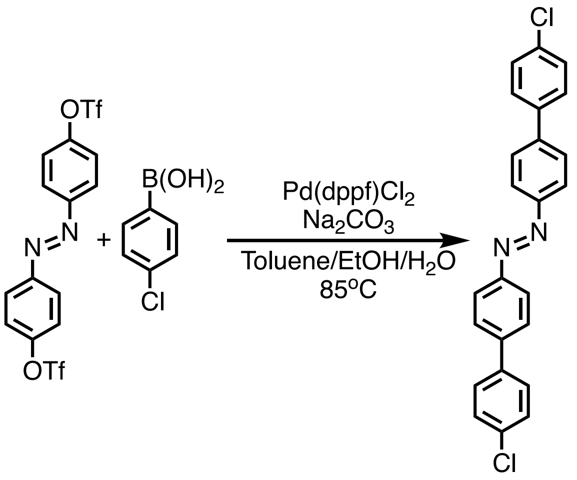

Toluene (8 mL), EtOH (2 mL), and H_2O (2 mL) were placed in a Schlenk tube, which was then vacuum degassed and filled with N_2 gas. 4,4′-Bis(trifluorosulfonyloxy)azobenzene (0.383 g, 0.801 mmol), 4-chlorobenzeneboronic acid (0.501 g, 3.20 mmol), KOAc (0.341 g, 3.21 mmol), and $PdCl_2$(dppf) (0.0367 g, 0.0480 mmol) were added to the Schlenk tube and stirred at 85 °C for 19 h. After cooling to room temperature, the precipitate was collected by suction filtration and washed with water and MeOH. The residue was dried in vacuo and passed through a silica plug (eluent: $CHCl_3$/AcOEt = 20:1, v/v). The solution was concentrated, and the residual powder was washed with hexane.

The insoluble fraction was collected by suction filtration and dried in vacuo to obtain an orange solid (0.230 g, 71%).

^{1}H NMR (500 MHz, $CDCl_3$, 299 K): δ 8.00 (d, J = 8.6 Hz, 4H), 7.70 (d, J = 8.6 Hz, 4H), 7.59 (d, J = 8.6 Hz, 4H), 7.43 (d, J = 8.6 Hz, 4H).

$^{13}C\{^{1}H\}$ NMR (125 MHz, $CDCl_3$, 300 K): δ 152.0 (C_q), 142.5 (C_q), 138.6 (C_q), 134.1 (C_q), 129.1 (CH), 128.4 (CH), 127.6 (CH), 123.5 (CH).

FT-IR (KBr, cm^{-1}): 1597, 1481, 1391, 1089, 1003, 857, 821, 741, 616.

HRMS (FD): calcd. for $C_{24}H_{16}N_2Cl_2$: 402.0691, found: m/z 402.0701 ($[M]^{\cdot+}$).

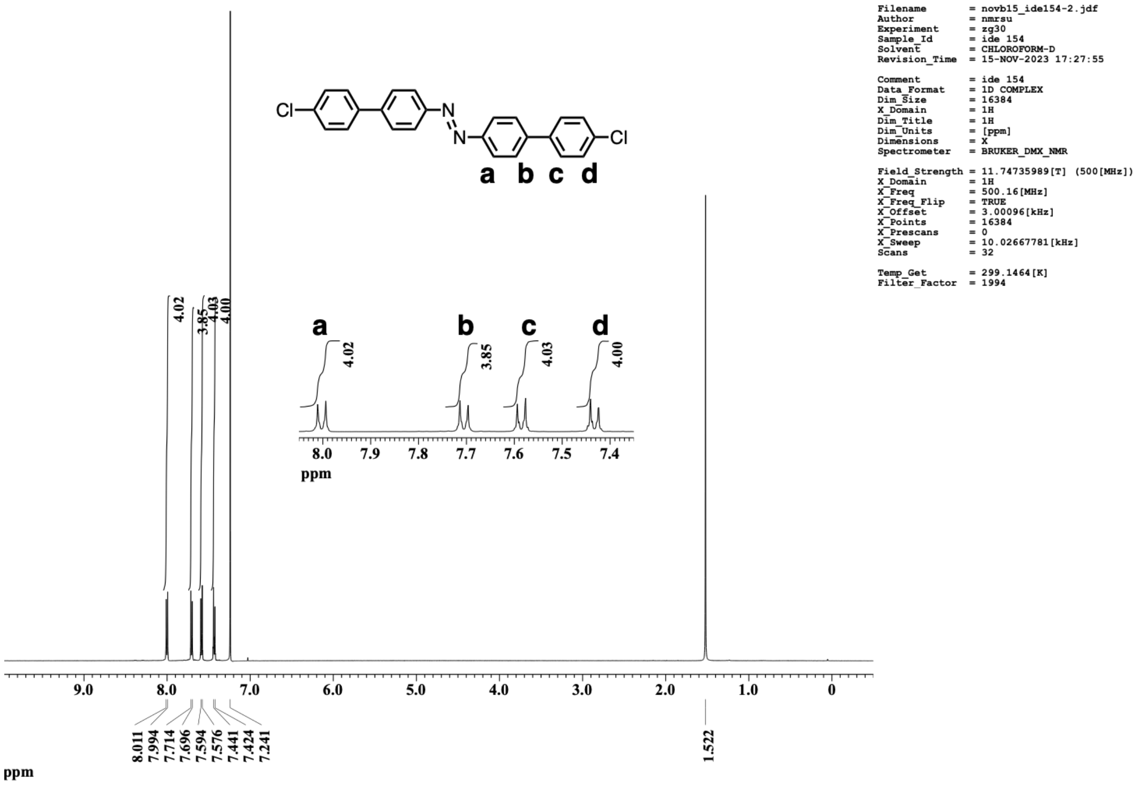

Figure S8. ^{1}H NMR spectrum (500 MHz, $CDCl_3$, 299 K) of 4,4′-bis(chlorophenyl)azobenzene.

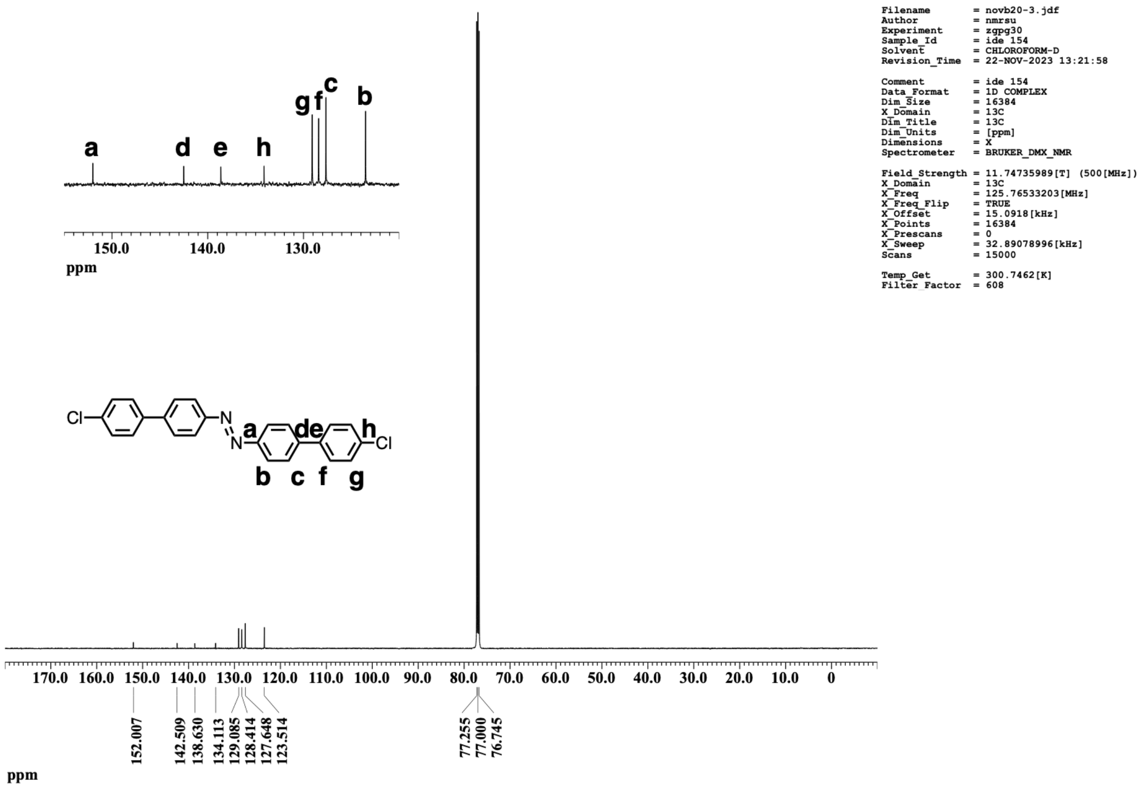

Figure S9. $^{13}C\{^{1}H\}$ NMR spectrum (125 MHz, $CDCl_3$, 300 K) of 4,4′-bis(chlorophenyl) azobenzene.

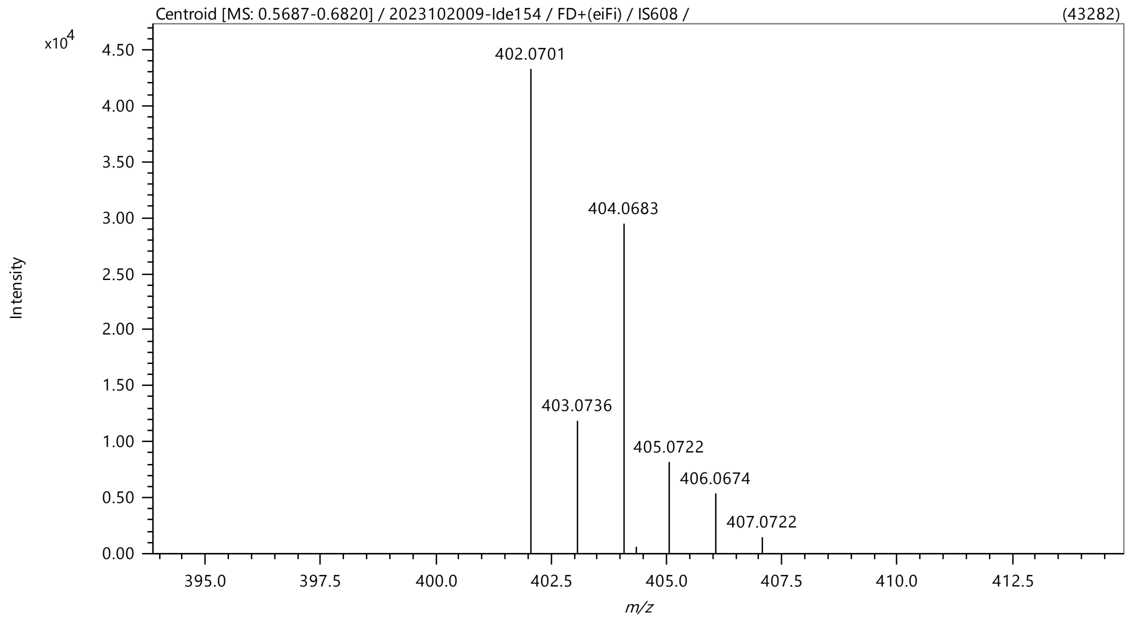

Figure S10. HR-FD-MS spectrum of 4,4′-bis(chlorophenyl)azobenzene.

S2.6. 4,4′-Bis(pinacolatoborylphenyl)azobenzene 2

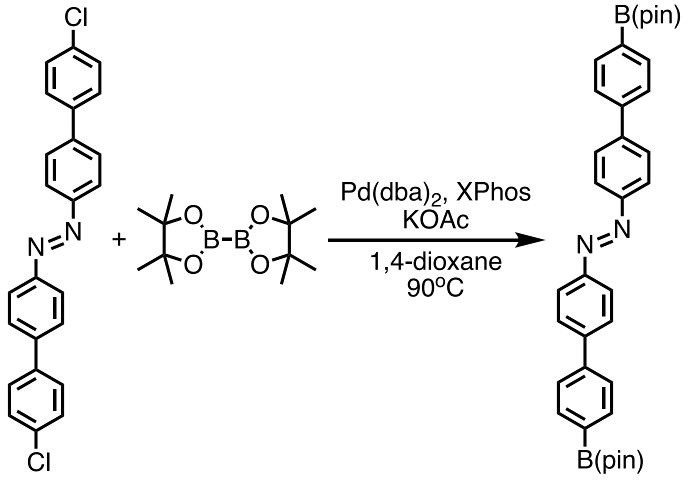

2

4,4′-Dichlorophenylazobenzene (0.208 g, 0.515 mmol), $B_2(pin)_2$ (0.507 g, 2.00 mmol), KOAc (0.304 g, 3.10 mmol), $Pd(dba)_2$ (0.0176 g, 0.0306 mmol), and XPhos (0.0293 g, 0.0615 mmol) were placed into a Schlenk tube and purged with N_2 gas. Vacuum-degassed 1,4-dioxane (5 mL) was added to a Schlenk tube and stirred at 90 °C for 20 h. After cooling to room temperature, the precipitate was collected by suction filtration and washed with water and MeOH. The residue was dried in vacuo and passed through a silica plug (eluenet: $CHCl_3$/AcOEt = 10:1, v/v). The solution was concentrated, and the residual powder was washed with hexane. The insoluble part was collected by suction filtration and dried in vacuo to obtain an orange powder (0.230 g, 62%).

^{1}H NMR (500 MHz, $CDCl_3$, 299 K): δ 8.01 (d, J = 8.6 Hz, 4H), 7.90 (d, J = 8.0 Hz, 4H), 7.77 (d, J = 8.6 Hz, 4H), 7.67 (d, J = 8.0 Hz, 4H), 1.36 (s, 24H).

^{13}C{^{1}H} NMR (125 MHz, $CDCl_3$, 300 K): δ 152.1 (C_q), 143.5 (C_q), 142.8 (C_q), 135.3 (CH), 127.9 (CH), 126.5 (CH), 123.4 (CH), 83.9 (C_q), 24.9 (CH_3)

FT-IR (KBr, cm^{-1}): 2976, 2928, 1608, 1399, 1361, 1324, 1274, 1215, 1144, 1092, 1023, 1004, 964, 861, 828, 732, 659.

HRMS (FD): calcd. for $C_{36}H_{40}B_2N_2O_4$: 586.3174, found: m/z 586.3180 ($[M]^{\cdot+}$).

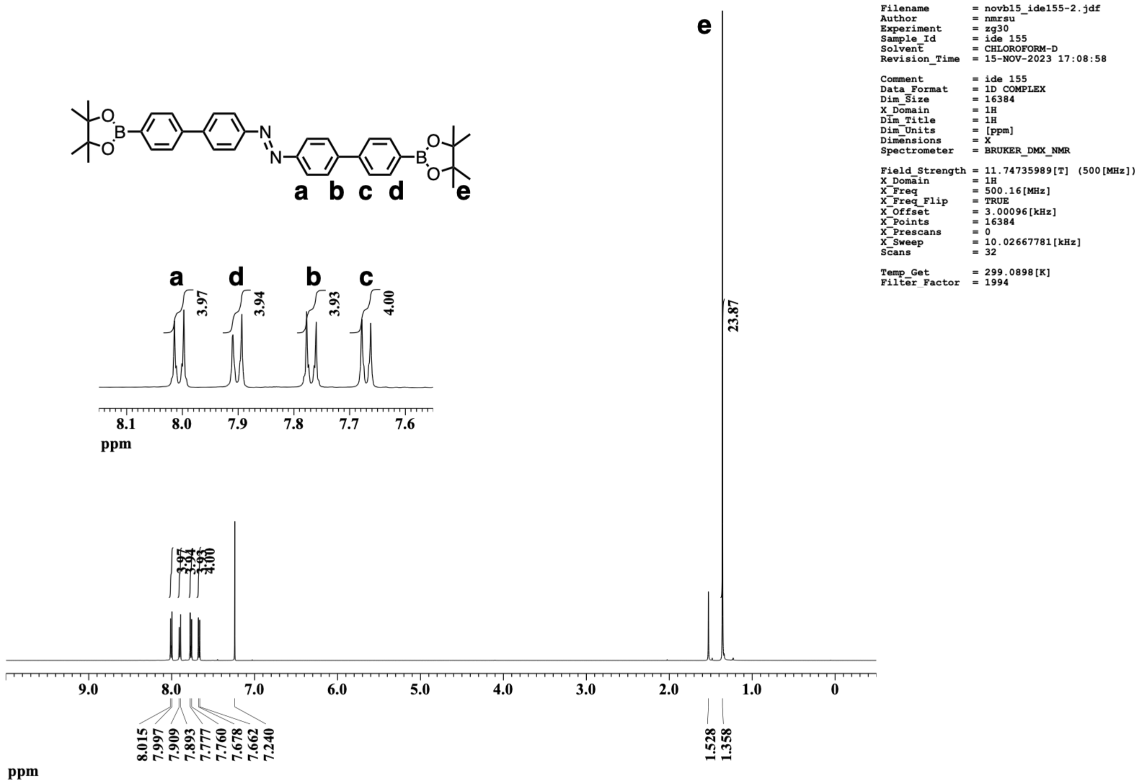

Figure S11. ^{1}H NMR spectrum (500 MHz, $CDCl_3$, 299 K) of 4,4′-bis(pinacolatoborylphenyl)azobenzene.

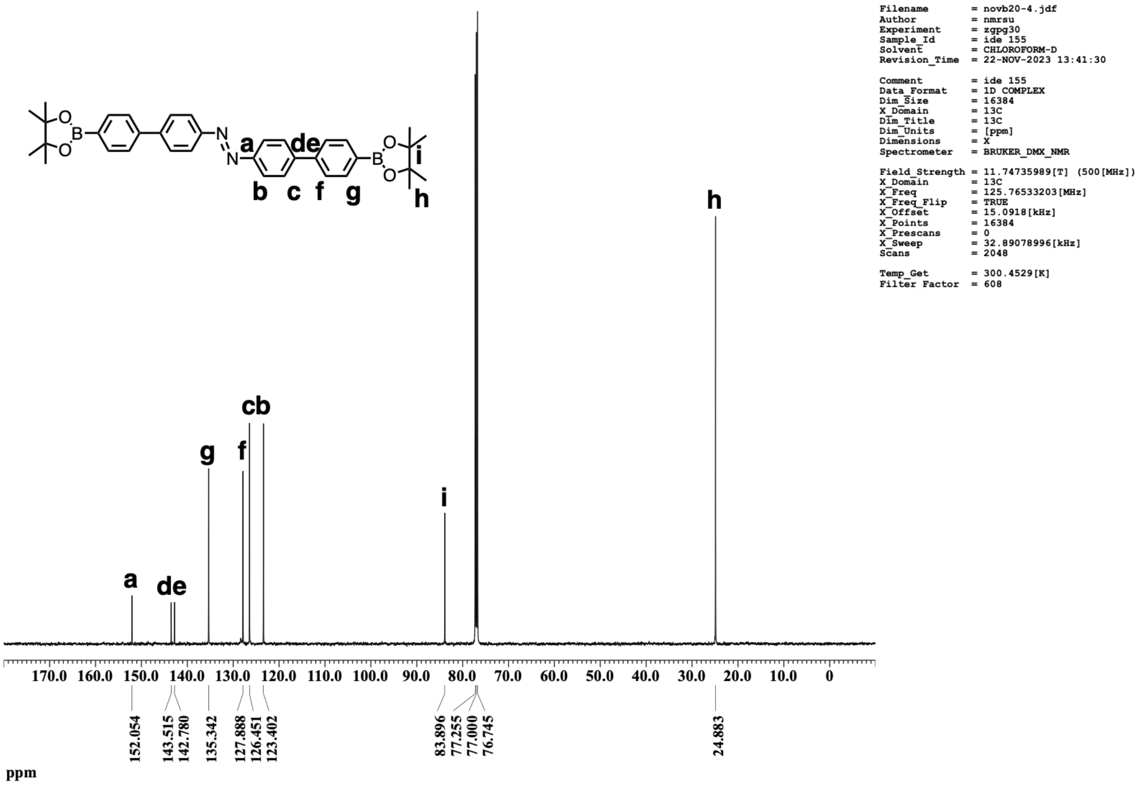

Figure S12. $^{13}C\{^{1}H\}$ NMR spectrum (125 MHz, $CDCl_3$, 300 K) of 4,4′-bis(pinacolato-borylphenyl)azobenzene.

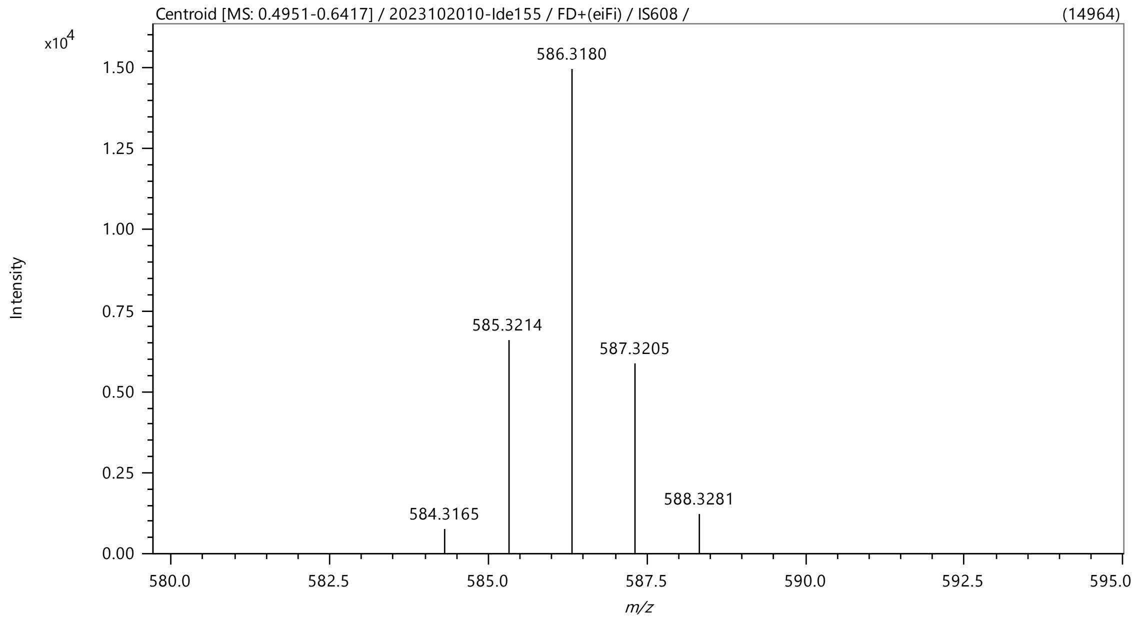

Figure S13. HR-FD-MS spectrum of 4,4′-bis(pinacolatoborylphenyl)azobenzene.

S2.7. [3]CAB-1

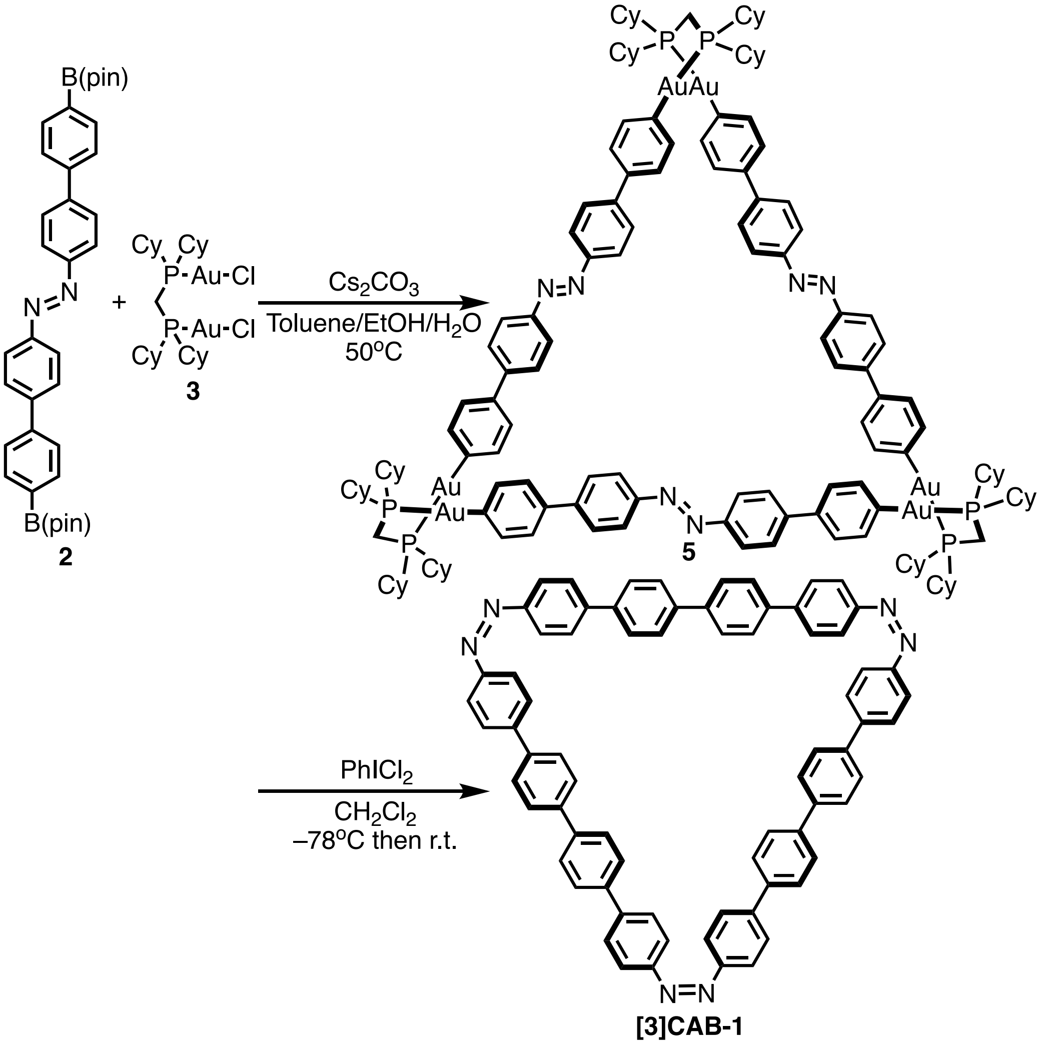

Toluene (8 mL), EtOH (2 mL), and H_2O (2 mL) were placed in a Schlenk tube, which was then vacuum degassed and filled with N_2 gas. 4,4′-Bis(pinacolatoborylphenyl)azobenzene **2** (117 mg, 0.201 mmol), Au_2Cl_2(dcpm) **3** (175 mg, 0.200 mmol), and Cs_2CO_3 (392 mg, 1.20 mmol) were added to the Schlenk tube. The mixture was stirred at 50 °C for 25 h. The resulting precipitate was collected by suction filtration and washed with H_2O, a small amount of toluene, and EtOH. The residual solid was dried in vacuo to afford the macrocyclic gold complex **5** as an orange powder (187 mg). This compound was used in the next step without further purification.

The macrocyclic gold complex (143 mg, 0.0420 mmol) and dichloromethane (40 mL) were placed in a Schlenk tube filled with N_2 gas. The mixture was then cooled to −78 °C. After light shielding with aluminum foil, a dichloromethane (20 mL) solution of iodobenzene dichloride (34.6 mg, 0.126 mmol) was added dropwise to a Schlenk tube over 40 min. The reaction mixture was stirred and kept at −78 °C for 30 min, allowed to

warm to room temperature, stirred for 25 h covered with aluminum foil to avoid ambient light. After the reaction, the mixture was handled under ambient light. The resulting solution was filtered to remove the orange precipitate. 0.40 M $Na_2S_2O_3$ aqueous solution (100 mL) was added to the orange solution followed by vigorous stirring for 30 min. After separating the aqueous layer, the organic layer was dried over $MgSO_4$. Filtration followed by removal of the solvent under reduced pressure. The residual solid was purified by silica gel column chromatography (eluent: CH_2Cl_2, then CH_2Cl_2/EtOAc = 10:1, v/v) to afford **[3]CAB-1** (20.9 mg, 41% in 2 steps) as an orange solid.

^{1}H NMR (500 MHz, $CDCl_3$, 299 K): δ 7.62 (12H, d, J = 8.5 Hz, 3′,5′-N-C_6H_4-C_6*H*$_4$), 7.59 (12H, d, J = 8.5 Hz, 2′,6′-N-C_6H_4-C_6*H*$_4$), 7.50 (12H, d, J = 8.5 Hz, 2,6-N-C_6*H*$_4$-C_6H_4), 6.89 (12H, d, J = 8.0 Hz, 3,5-N-C_6*H*$_4$-C_6H_4).

^{13}C{^{1}H} NMR (125 MHz, $CDCl_3$, 300 K): δ153.3 (Cq), 139.7 (Cq), 139.3 (Cq), 138.7 (Cq), 127.3 (CH), 127.2 (CH), 126.9 (CH), 121.1 (CH).

FT-IR (KBr, cm^{-1}): 3028, 1710, 1607, 1481, 1394, 1361, 1109, 896, 808, 779.

HRMS (FD): calcd. for $C_{72}H_{48}N_6$: 996.3940, found: *m/z* 996.3935 ($[M]^{\cdot+}$).

Decomposition temperature: 424 °C.

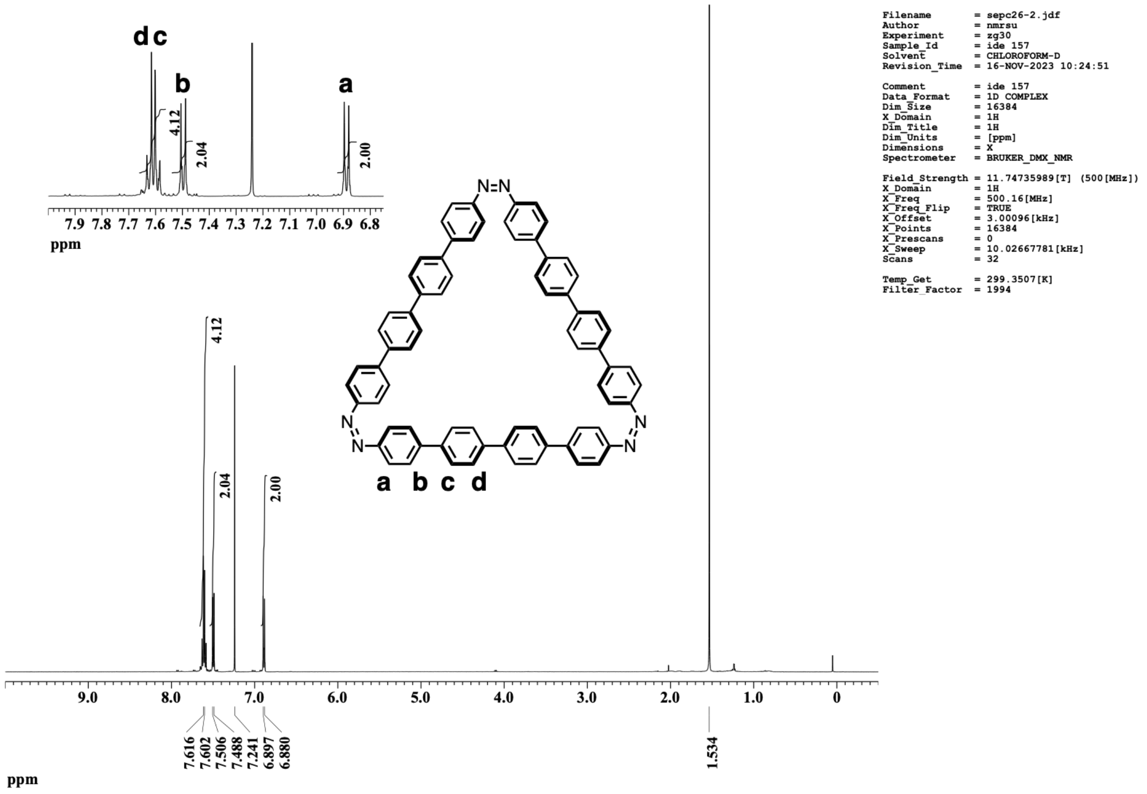

Figure S14. ^{1}H NMR spectrum (500 MHz, $CDCl_3$, 299 K) of **[3]CAB-1**.

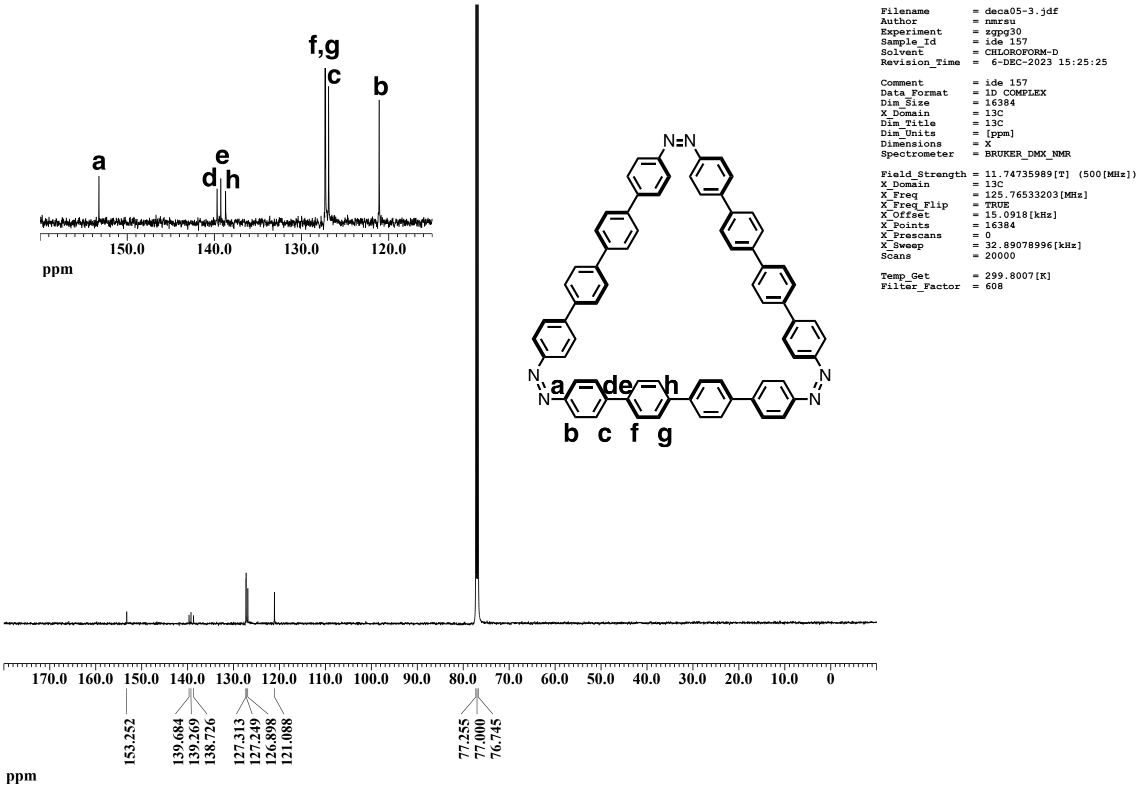

Figure S15. $^{13}C\{^{1}H\}$ NMR spectrum (125 MHz, $CDCl_3$, 300 K) of **[3]CAB-1**.

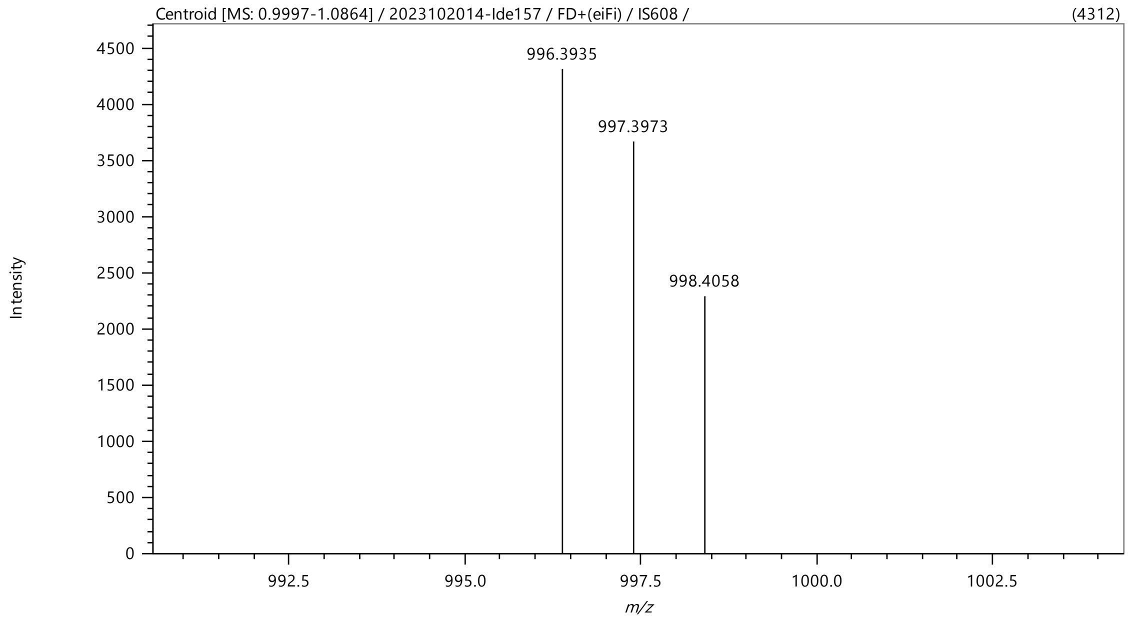

Figure S16. HR-FD-MS spectrum of **[3]CAB-1**.

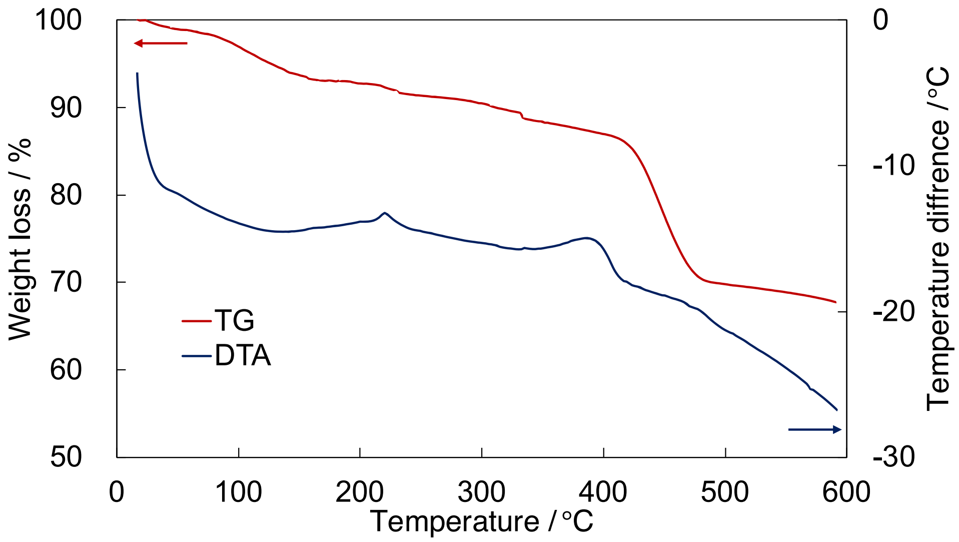

Figure S17. TG/DTA curves of **[3]CAB-1** (under N_2 flow, 10 °C min^{-1}). Decomposition temperature was found at 424 °C. Color of the sample was changed from orange to black after the measurement.

S3. Crystallographic Studies

Single crystals of macrocycles suitable for X-ray crystallography were obtained by vapor diffusion of cyclohexane into a $CHCl_3$ solution of the molecule. Disordered solvent molecules were removed using the SQUEEZE procedure.

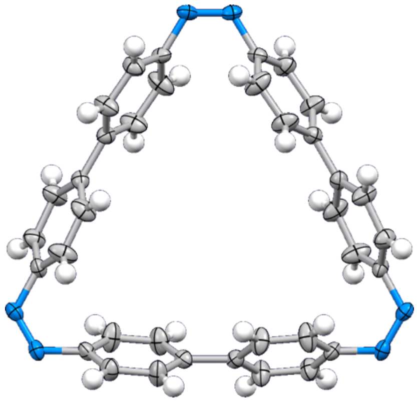

Figure S18. Crystal structure (30% probability) of **[3]CAB-0**; (blue) N; (grey) C; (white) H.

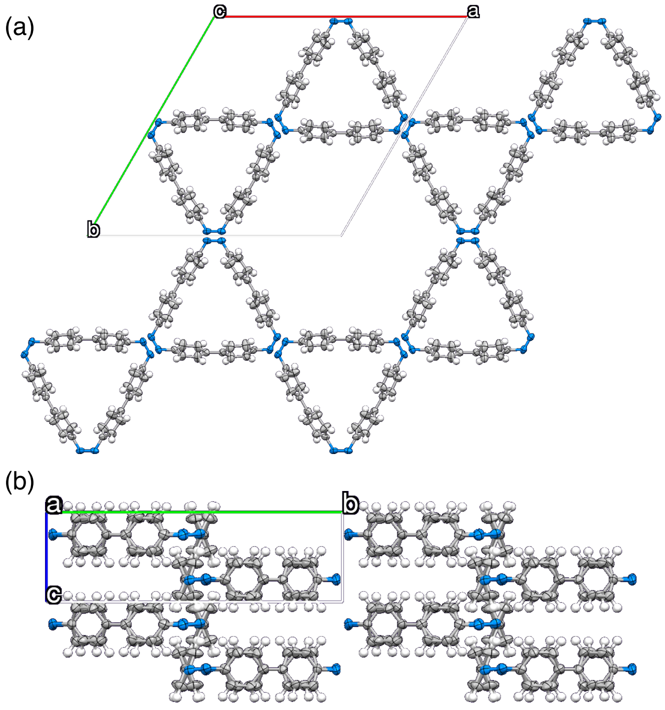

Figure S19. Packing diagram (30% probability) of **[3]CAB-0** view along (a) *c*-axis and (b) *a*-axis; (blue) N; (grey) C; (white) H. Solvents were removed using the SQUEEZE procedure.

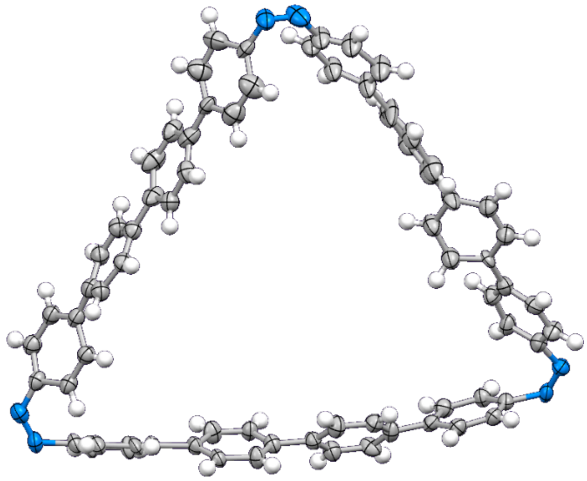

Figure S20. Crystal structure (30% probability) of **[3]CAB-1**; (blue) N; (grey) C; (white) H.

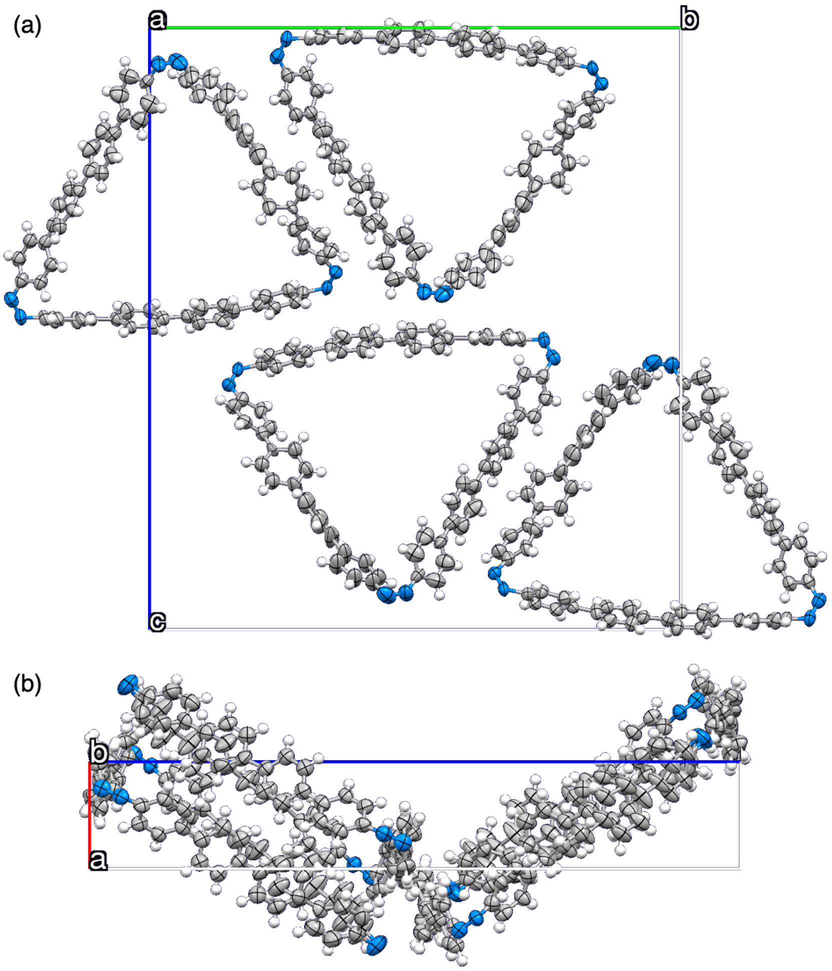

Figure S21. Packing diagram (30% probability) of **[3]CAB-1** view along (a) *a*-axis and (b) *b*-axis; (blue) N; (grey) C; (white) H. Disordered macrocycles are omitted for clarity. Solvents were removed using the SQUEEZE procedure.

Table S1. Crystal data and structure refinement for **[3]CAB-0**

CCDC Number	2321643
Empirical formula	$C_{72}H_{48}N_{12}$
Formula weight	1081.22
Temperature/K	100.00(10)
Crystal system	hexagonal
Space group	$P6_322$
a/Å	22.5473(4)
b/Å	22.5473(4)
c/Å	5.93370(10)
α/°	90
β/°	90
γ/°	120
Volume/Å^3	2612.43(10)
Z	1
ρ_{calc} g/cm^3	0.687
μ/mm^{-1}	0.328
F(000)	564.0
Crystal size/mm^3	0.228 × 0.079 × 0.045
Radiation	Cu K_α (λ = 1.54184)
2Θ range for data collection/°	4.526 to 134.032
Index ranges	-26 ≤ h ≤ 17, -26 ≤ k ≤ 26, -6 ≤ l ≤ 7
Reflections collected	22548
Independent reflections	1574 [R_{int} = 0.0402, R_{sigma} = 0.0152]
Data/restraints/parameters	1574/0/66
Goodness-of-fit on F^2	1.137
Final *R* indexes [$I \geqq 2\sigma(I)$]	R_1 = 0.0822, wR_2 = 0.2358
Final *R* indexes [all data]	R_1 = 0.0843, wR_2 = 0.2391
Largest diff. peak/hole / e Å^{-3}	0.35/-0.38
Flack parameter	1.2(14)

Table S2. Crystal data and structural refinement for **[3]CAB-1**

CCDC Number	2321642
Empirical formula	$C_{72}H_{48}N_6$
Formula weight	997.16
Temperature/K	100.00(10)
Crystal system	orthorhombic
Space group	$P2_12_12_1$
a/Å	5.9370(3)
b/Å	31.8015(17)
c/Å	36.073(2)
α/°	90
β/°	90
γ/°	90
Volume/Å^3	6810.7(7)
Z	4
ρ_{calc} g/cm^3	0.972
μ/mm^{-1}	0.443
F(000)	2088.0
Crystal size/mm^3	0.12 × 0.03 × 0.01
Radiation	Cu K_α (λ = 1.54184)
2Θ range for data collection/°	4.9 to 149.742
Index ranges	$-6 \leq h \leq 7$, $-39 \leq k \leq 37$, $-43 \leq l \leq 43$
Reflections collected	41031
Independent reflections	12338 [R_{int} = 0.0629, R_{sigma} = 0.0646]
Data/restraints/parameters	12338/1061/999
Goodness-of-fit on F^2	1.079
Final *R* indexes [$I \geqq 2\sigma(I)$]	R_1 = 0.1165, wR_2 = 0.2941
Final *R* indexes [all data]	R_1 = 0.1655, wR_2 = 0.3350
Largest diff. peak/hole / e Å^{-3}	0.54/-0.33
Flack parameter	−0.7(11)

S4. Analysis of Intermolecular Interaction in Crystal of [3]CAB-0

The crystal of **[3]CAB-1** showed short contacts between CH on benzene ring and N on azo group (Figure S22, dotted lines).

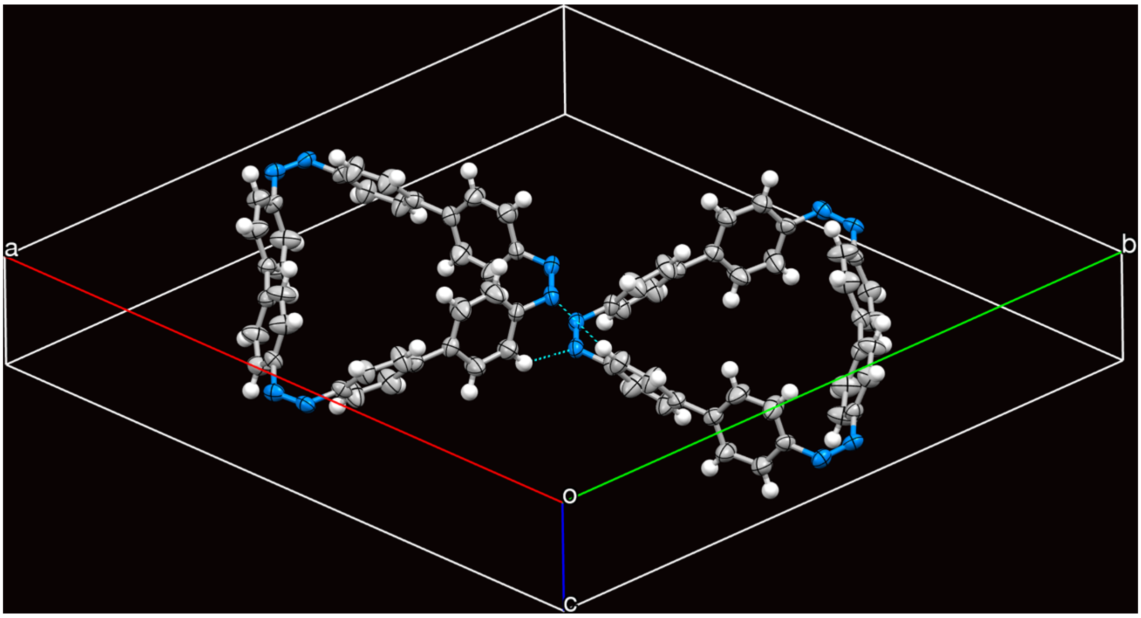

Figure S22. Short contacts (dotted line) in **[3]CAB-0** crystal.

Symmetry-adapted perturbation theory calculation at the SAPT0/jun-cc-pVDZ level was performed with respect to two contacted molecules in Figure S22. The calculation results are as follows:

- Electrostatics −8.75 kcal mol^{-1}
- Exchange 10.6 kcal mol^{-1}
- Induction −1.86 kcal mol^{-1}
- Dispersion −9.46 kcal mol^{-1}
- Total SAPT0 −9.48 kcal mol^{-1}

Interestingly, the electrostatics is comparable to the dispersion. This result suggests the existence of CH···N hydrogen bond. In contrast, the electrostatics are almost zero and the dispersive forces are dominant in the column direction (Figure S23).

- Electrostatics −0.09 kcal mol^{-1}
- Exchange 7.27 kcal mol^{-1}
- Induction −1.08 kcal mol^{-1}
- Dispersion −16.8 kcal mol^{-1}
- Total SAPT0 −10.7 kcal mol^{-1}

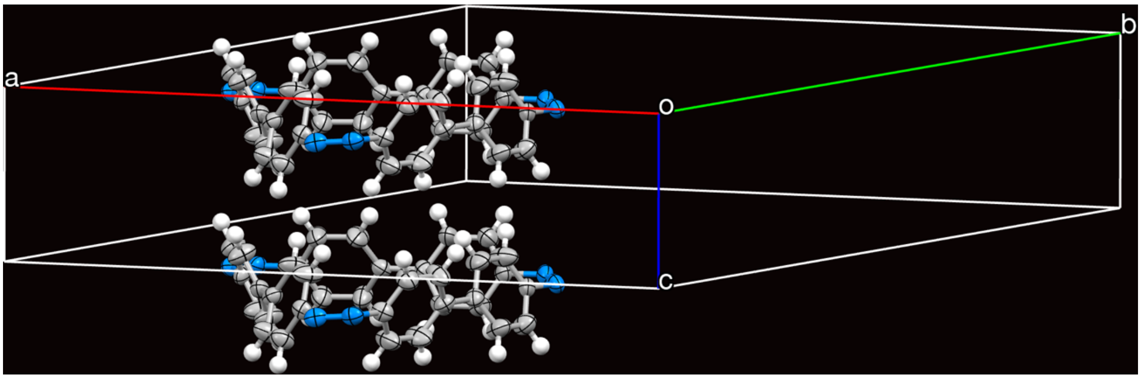

Figure S23. Stacking structure of **[3]CAB-0** in crystal.

It noted that the total interactions (Total SAPT0) in the two directions are comparable. It is speculated that **[3]CAB-0** takes the Kagome structure because of the balanced interaction of CH···N hydrogen bonds and dispersion force between biphenylenes. The dispersion force increases in proportion to the size of the molecules. Thus, in the case of **[3]CAB-1**, stronger dispersion forces between tetraphenylenes are considerably more dominant in forming the crystal structure, and the Kagome structure is not formed.

S5. Photoisomerization Experiments

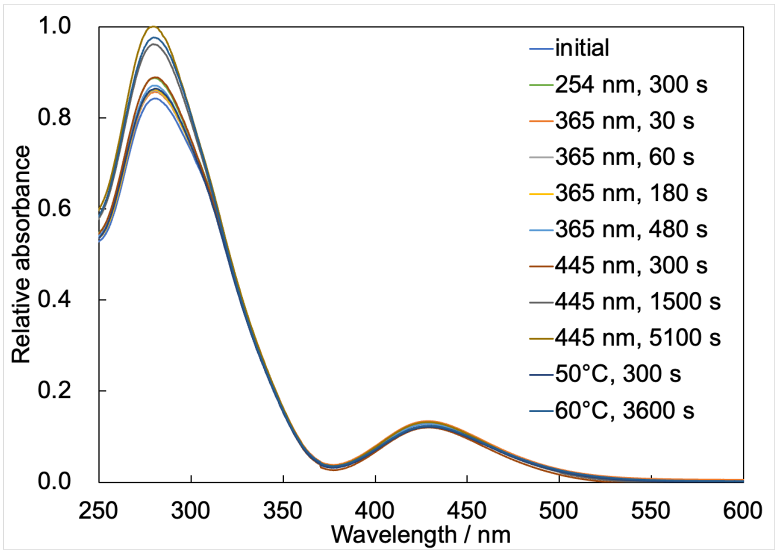

Figure S24. UV-Vis spectra of **[3]CAB-0** (0.02 mM 1,2-dichloroethane). Experiments were performed at 25°C unless otherwise specified.

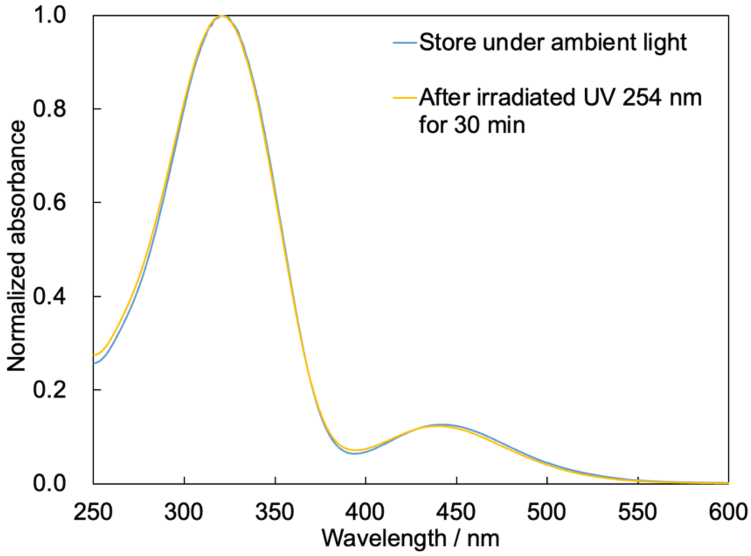

Figure S25. UV-Vis spectra of **[3]CAB-1** (1,2-dichloroethane, 25°C). Powders stored under ambient light and irradiated at 254 nm for 30 min were dissolved in 1,2-dichloromethane and measured immediately.

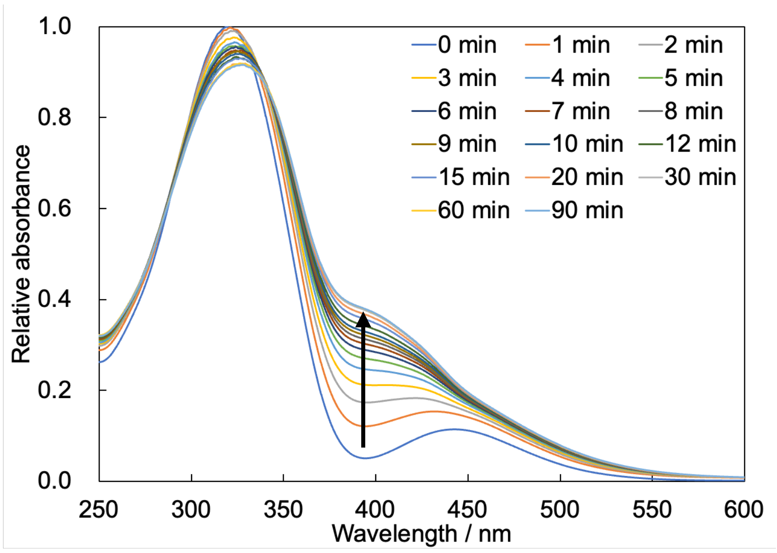

Figure S26. UV-Vis spectra of **[3]CAB-1** (0.02 mM 1,2-dichloroethane, 25°C). Solution was standing up to 90 min under ambient light at 22°C.

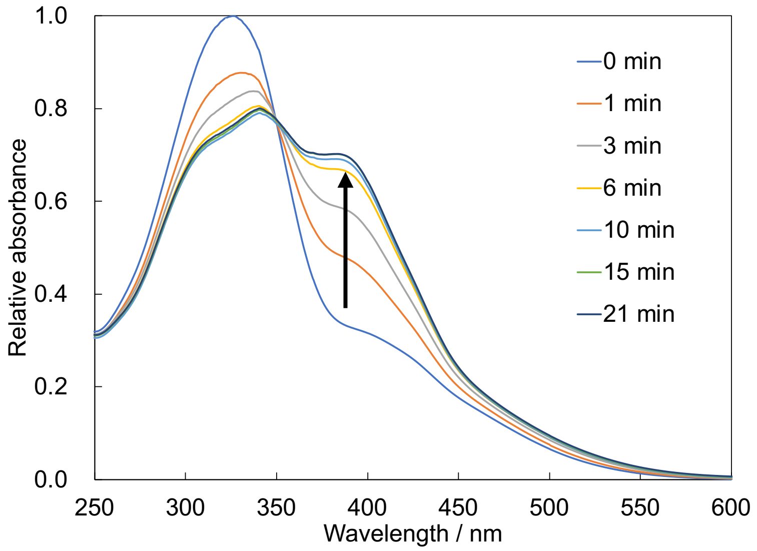

Figure S27. UV-Vis spectra of **[3]CAB-1** (0.02 mM 1,2-dichloroethane, 25°C). Solution was irradiated at 254 nm for up to 21 min from photostationary state at room temperature and under ambient light.

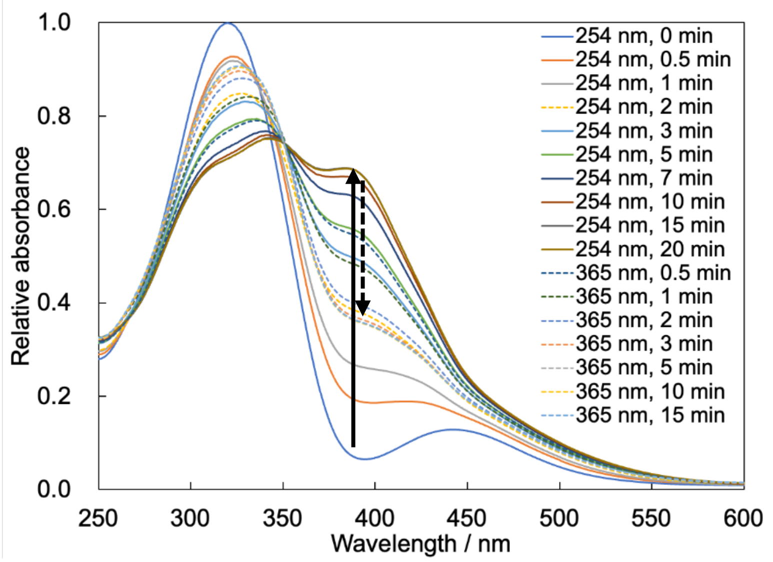

Figure S28. UV-Vis spectra of **[3]CAB-1** (0.02 mM 1,2-dichloroethane, 25°C). Solution was irradiated at 254 nm for up to 20 min and then at 365 nm for up to 15 min.

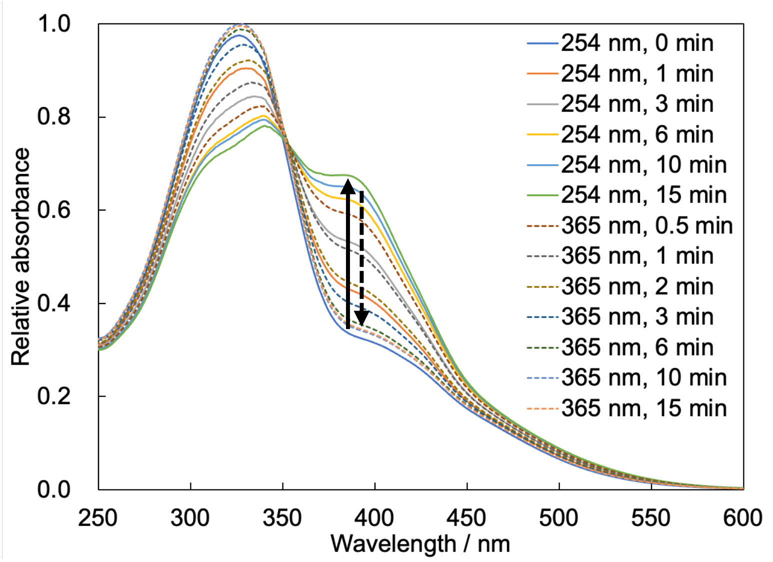

Figure S29. UV-Vis spectra of **[3]CAB-1** (0.02 mM 1,2-dichloroethane, 25°C). Solution was irradiated at 254 nm for up to 15 min and then at 365 nm for up to 15 min. Starting from a photostationary state at room temperature and under ambient light.

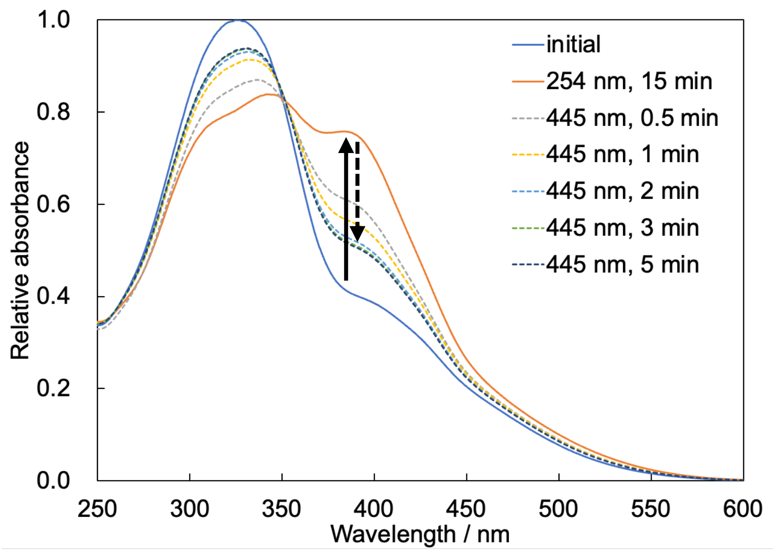

Figure S30. UV-Vis spectra of **[3]CAB-1** (0.02 mM 1,2-dichloroethane, 25°C). Solution was irradiated at 254 nm for 15 min and then at 445 nm for up to 5 min.

S6. Photo isomerization cycles

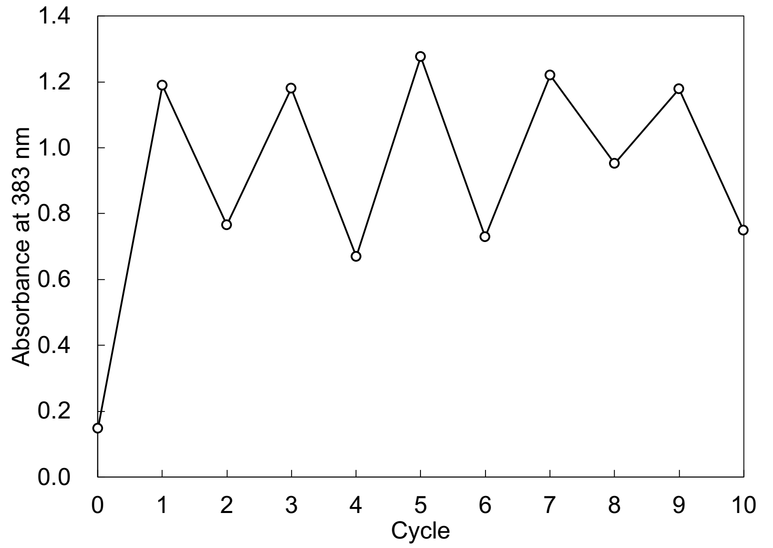

Figure S31. Photo isomerization cycle of **[3]CAB-1** irradiation at 254 nm and 356 nm, starting from all-*cis* isomer (0.02 mM 1,2-dichloroethane, 25°C).

S7. Thermal Isomerization Experiments

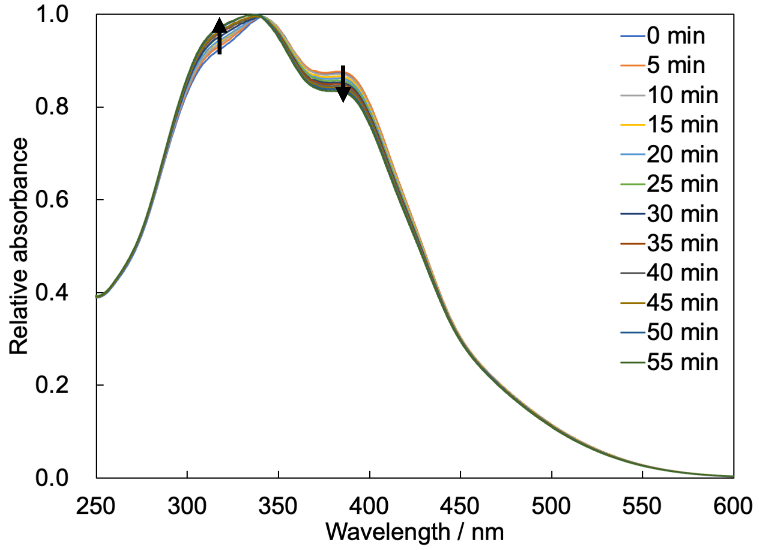

Figure S32. UV-Vis spectra of **[3]CAB-1** after irradiation at 254 nm for 15 min (0.02 mM 1,2-dichloroethane, 20°C). Solution was kept at 20°C for up to 55 min in the UV-Vis spectrometer.

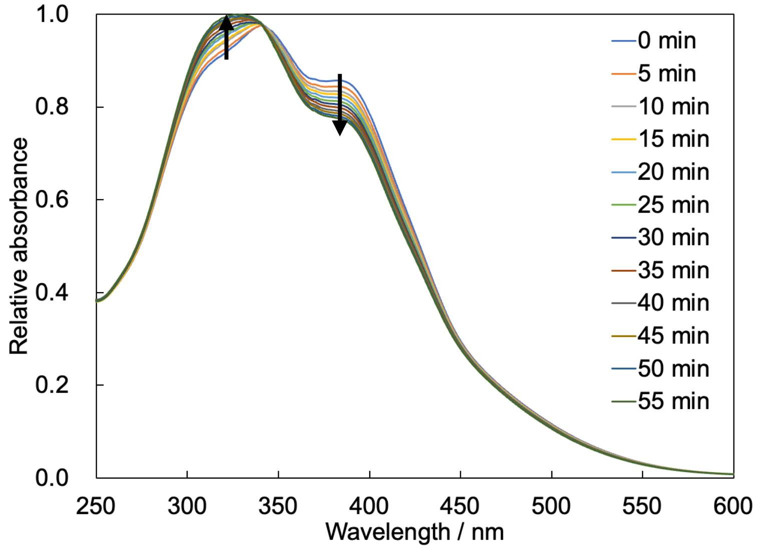

Figure S33. UV-Vis spectra of **[3]CAB-1** after irradiation at 254 nm for 15 min (0.02 mM 1,2-dichloroethane, 25°C). Solution was kept at 25°C for up to 55 min in the UV-Vis spectrometer.

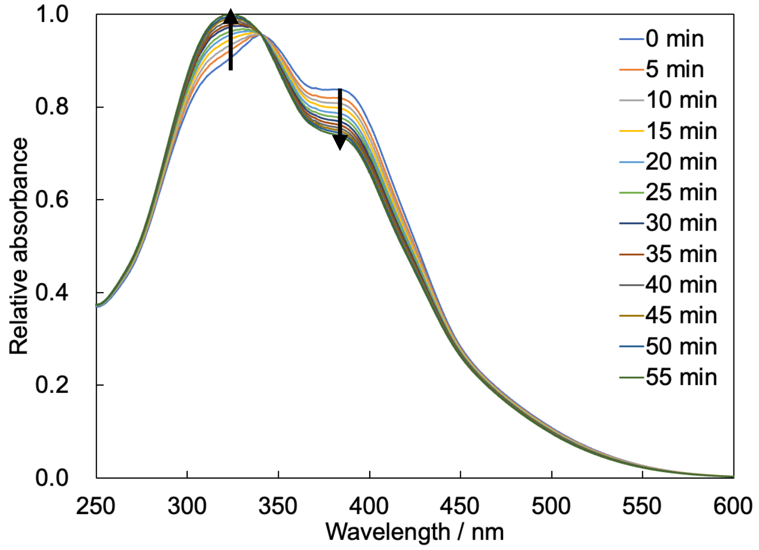

Figure S34. UV-Vis spectra of **[3]CAB-1** after irradiation at 254 nm for 15 min (0.02 mM 1,2-dichloroethane, 30°C). Solution was kept at 30°C for up to 55 min in the UV-Vis spectrometer.

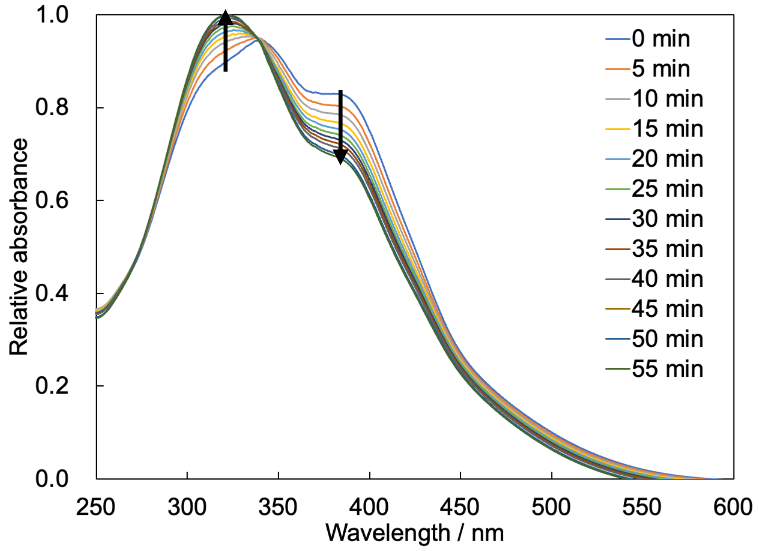

Figure S35. UV-Vis spectra of **[3]CAB-1** after irradiation at 254 nm for 15 min (0.02 mM 1,2-dichloroethane, 35°C). Solution was kept at 35°C for up to 55 min in the UV-Vis spectrometer.

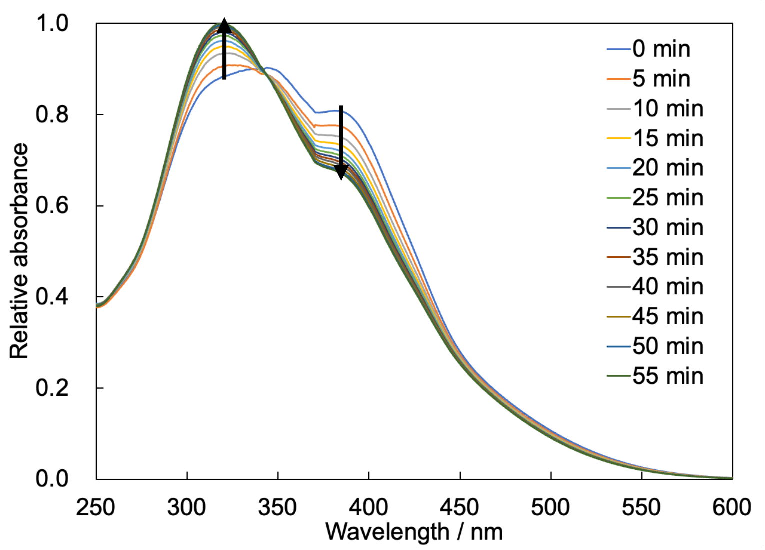

Figure S36. UV-Vis spectra of **[3]CAB-1** after irradiation at 254 nm for 15 min (0.02 mM 1,2-dichloroethane, 40°C). Solution was kept at 40°C for up to 55 min in the UV-Vis spectrometer.

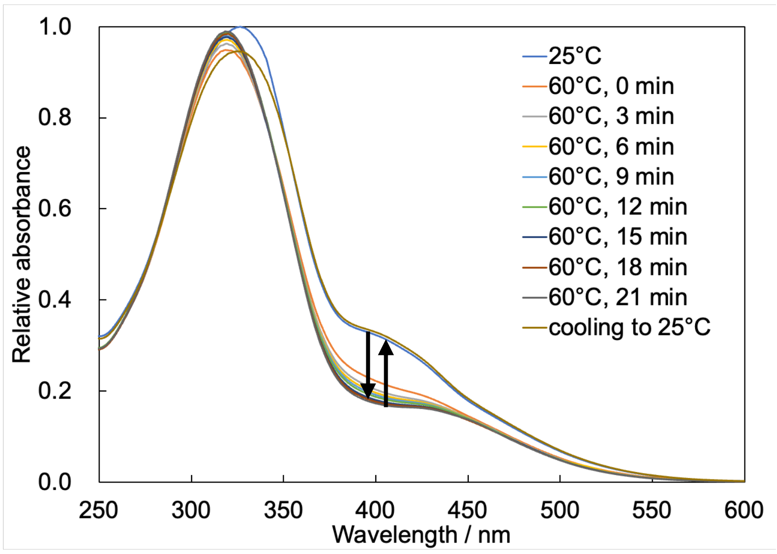

Figure S37. UV-Vis spectra of **[3]CAB-1** (0.02 mM 1,2-dichloroethane, 25°C → 60°C → 25°C).

S8. First-Order Analysis

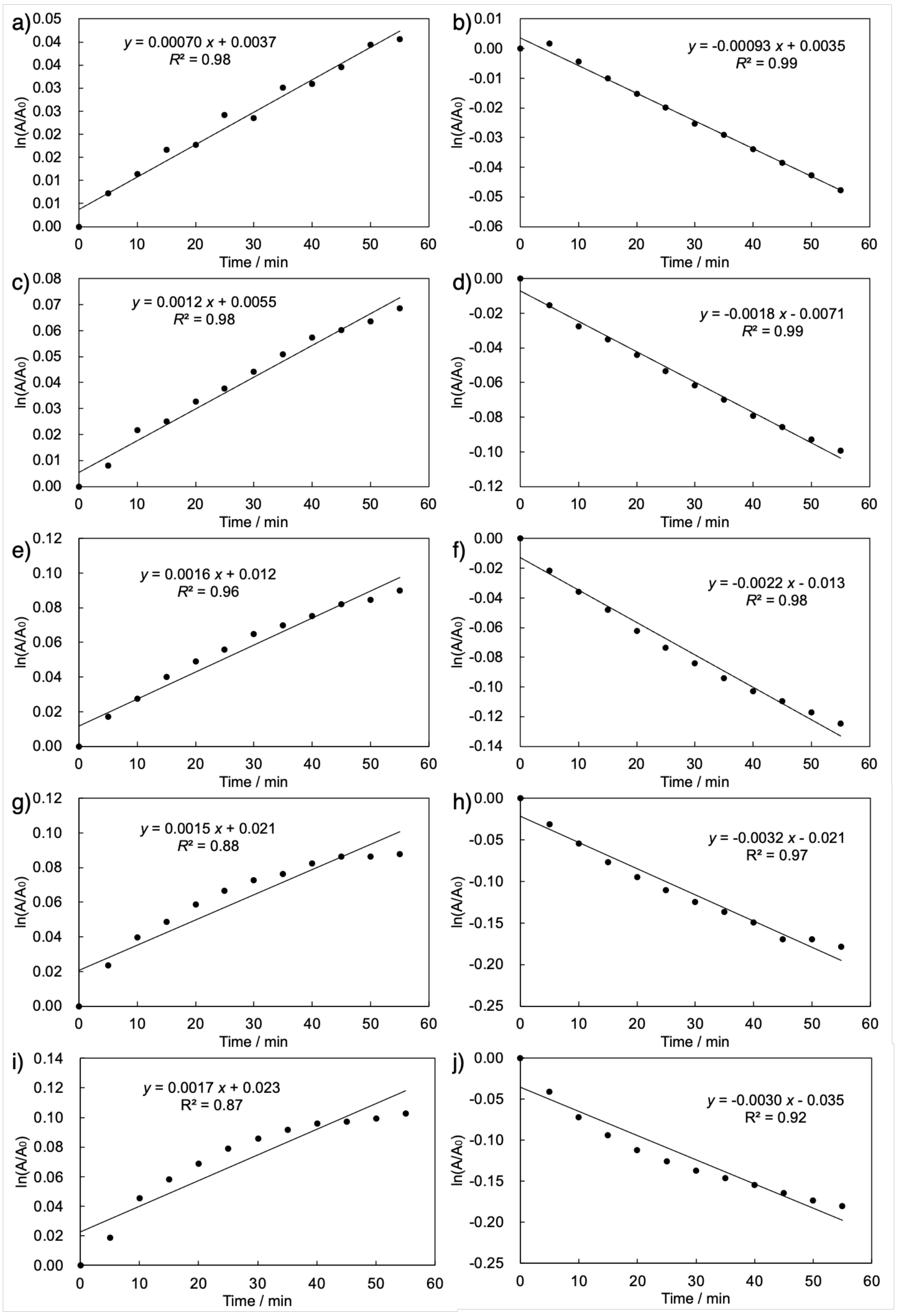

Figure S38. First-order plot of a) at 326 nm and 20°C, b) at 382 nm and 20°C, c) at 326 nm and 25°C, d) at 382 nm and 25°C, e) at 326 nm and 30°C, f) at 382 nm and 30°C, g) at 326 nm and 35°C, h) at 382 nm and 35°C, i) at 326 nm and 40°C, j) at 382 nm and 40°C. It no longer seemed to fit first-order plot as the temperature increased. The wavelength values are λ_{max} of 0 min (326 nm, 25°C) and 55 min (382 nm, 25°C).

The thermal relaxation from the photostationary state induced by 254 nm UV light can be regarded as a pseudo-first-order reaction around room temperature, despite it expecting a multi-step reaction, with a rate constant of $k_r = 1.8 \times 10^{-3}$ min^{-1} and a half-life $t_{1/2} = 385$ min at 25°C based on absorbance at 382 nm (Figure S34). The activation energy (E_a) determined within the highly linear temperature range (20°C to 35°C) for first-order analysis was found to be 14 kcal mol^{-1} (Table S3 and Figure S39).

Table S3. Result of first-order analysis at 382 nm.

T / °C	k_r / min^{-1}	$t_{1/2}$ / min
20	9.3×10^{-4}	745
25	1.8×10^{-3}	385
30	2.2×10^{-3}	315
35	3.2×10^{-3}	216
40	3.0×10^{-3}	231

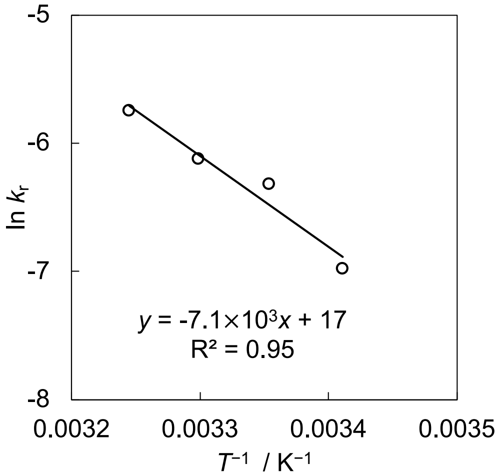

Figure S39. Arrhenius plot (0.02 mM 1,2-dichloroethane, 20–35°C) based on absorbance at 382 nm. Activation energy $E_a = 14$ kcal mol^{-1} was calculated from the slope of the plot.

S9. Acid Responses

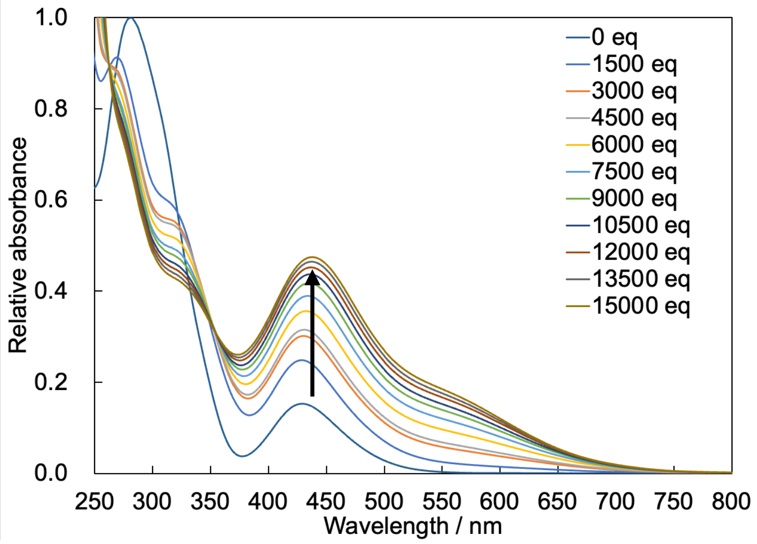

Figure S40. UV-Vis spectra of **[3]CAB-0** in the presence of different equivalents of TFA (trifluoroacetic acid) (0.02 mM 1,2-dichloroethane, 25°C).

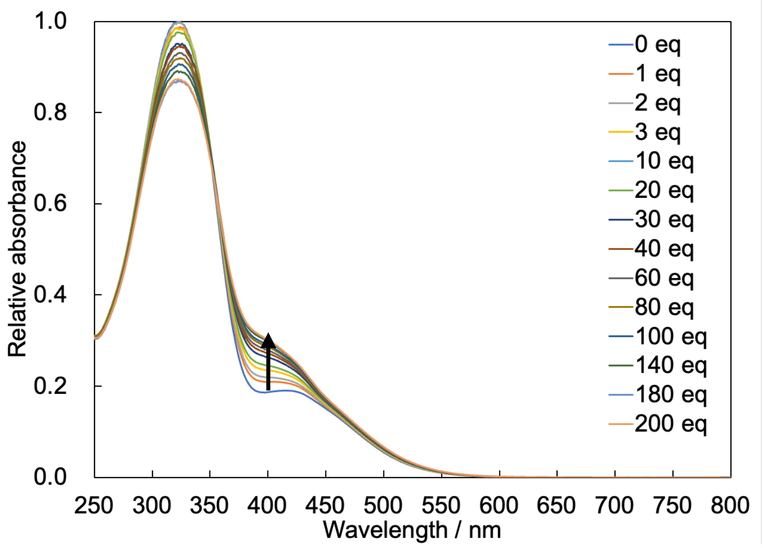

Figure S41. UV-Vis spectra of **[3]CAB-1** in the presence of different equivalents of TFA (0.02 mM 1,2-dichloroethane, 25°C).

S10. Photo Responses in Acidic Solution

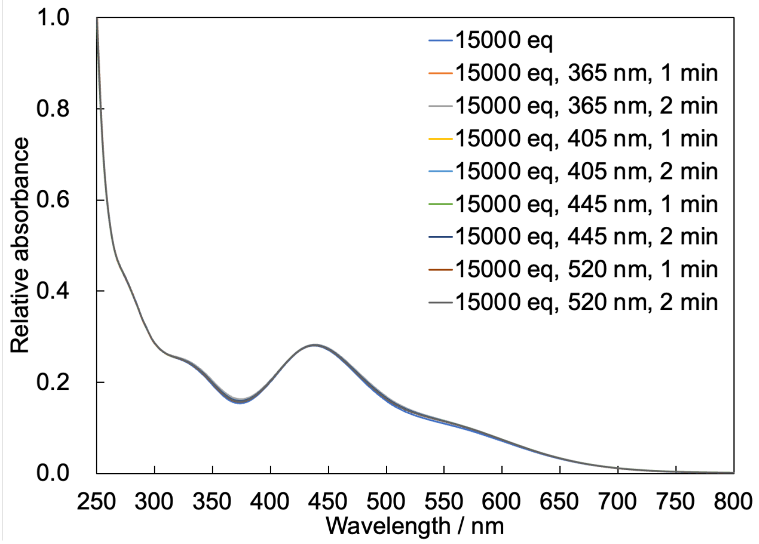

Figure S42. UV-Vis spectra of **[3]CAB-0** in the presence of 15000 eq. TFA irradiated at different wavelengths of light (0.02 mM 1,2-dichloroethane, 25°C).

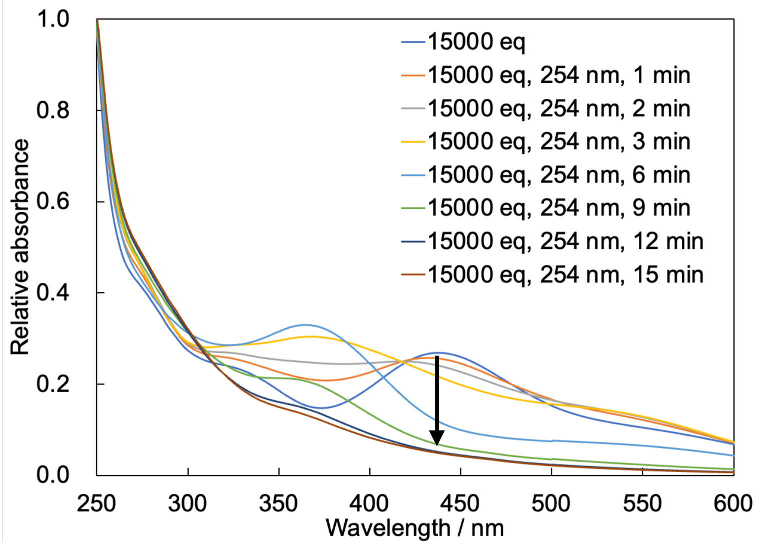

Figure S43. UV-Vis spectra of **[3]CAB-0** in the presence of 15000 eq. of TFA irradiated at 254 nm (0.02 mM 1,2-dichloroethane, 25°C).

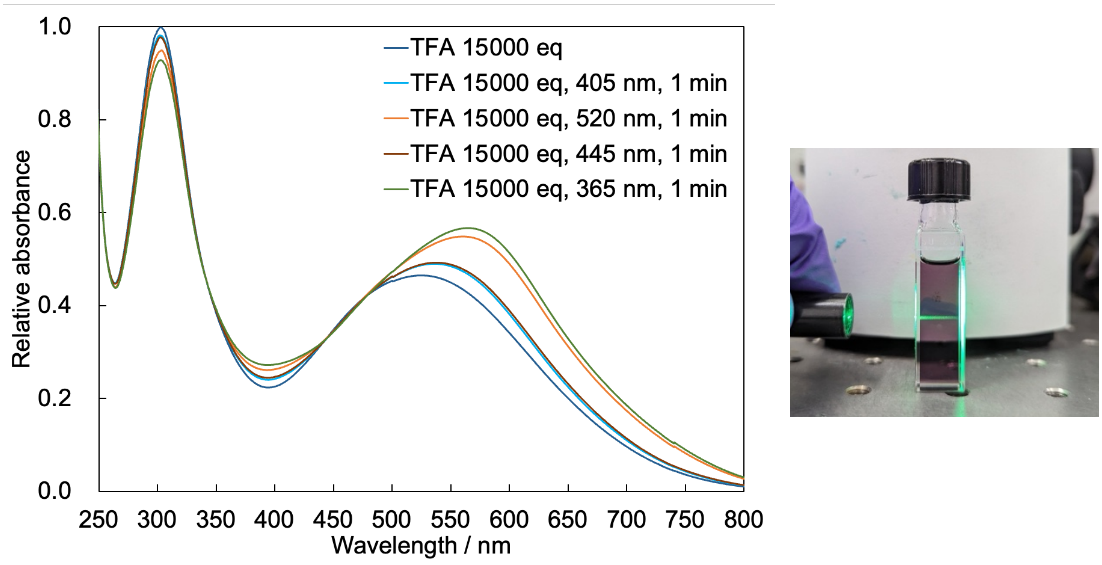

Figure S44. UV-Vis spectra of **[3]CAB-1** in the presence of 15000 eq. TFA irradiated at different wavelengths of light (0.02 mM 1,2-dichloroethane, 25°C).

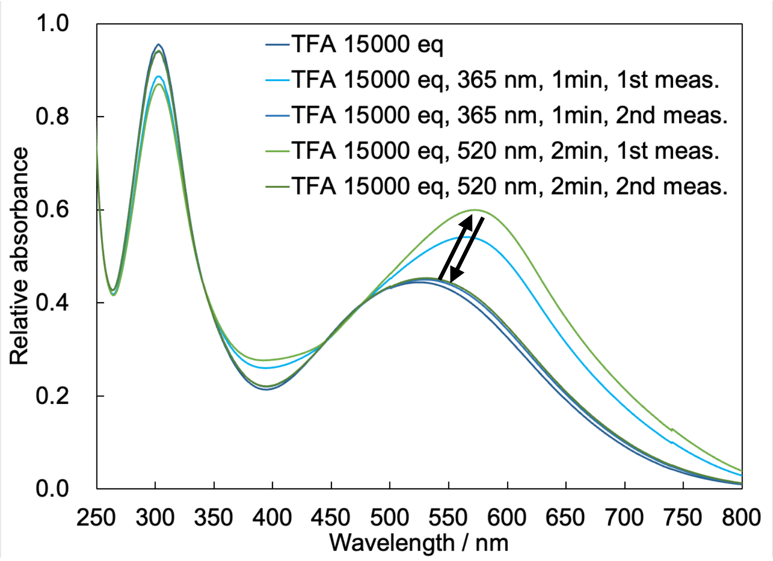

Figure S45. UV-Vis spectra of **[3]CAB-1** in the presence of 15000 eq. of TFA irradiated with different wavelengths (0.02 mM 1,2-dichloroethane, 25°C). Spectra were measured after photoirradiation 2 times. Each measurement took approximately 2 min.

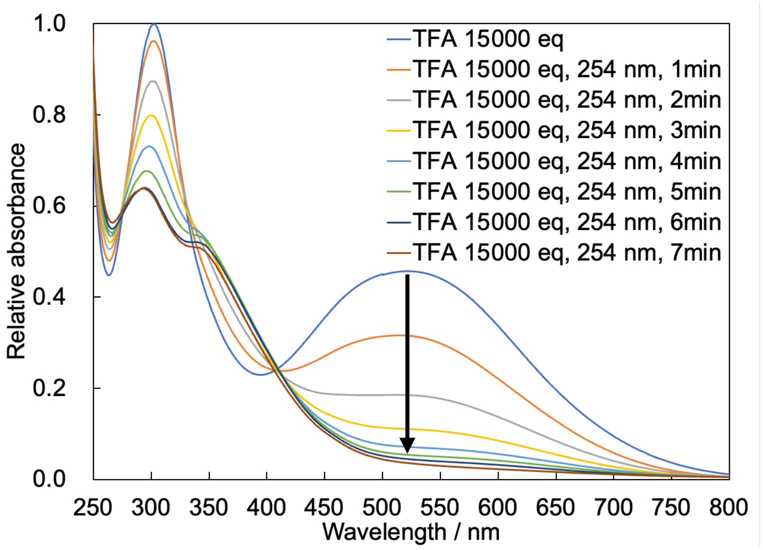

Figure S46. UV-Vis spectra of **[3]CAB-1** in the presence of 15000 eq. of TFA irradiated at 254 nm (0.02 mM 1,2-dichloroethane, 25°C).

S11. Heating in Acidic Solution

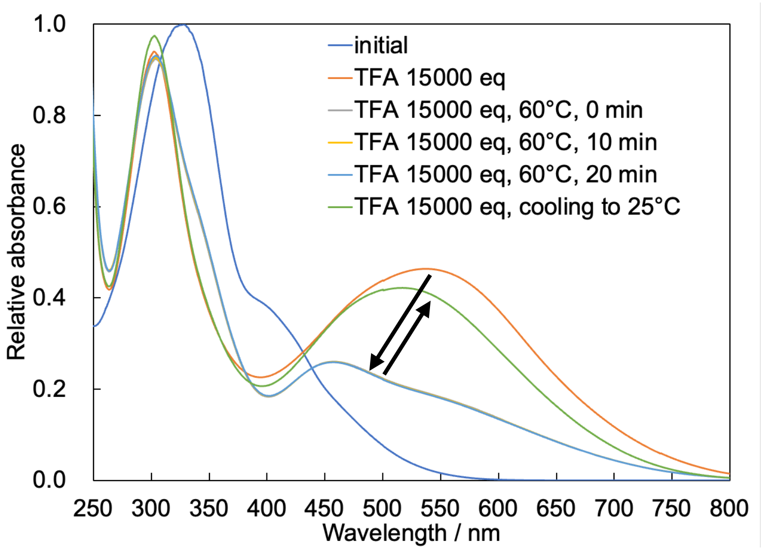

Figure S47. UV-Vis spectra of **[3]CAB-1** in the presence of 15000 eq. of TFA (0.02 mM 1,2-dichloroethane, 25°C → 60°C → 25°C).

S12. Addition of the Bases to Acidic Solution

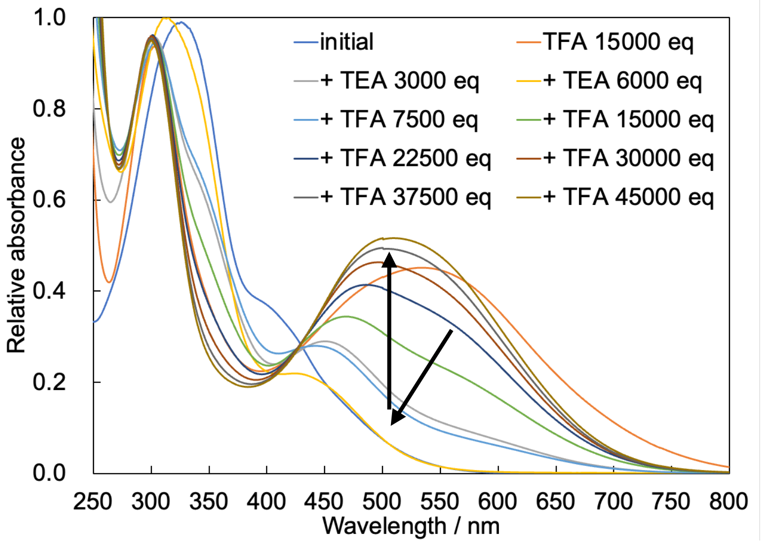

Figure S48. UV-Vis spectra of **[3]CAB-1** (0.02 mM 1,2-dichloroethane, 25°C). First acidification by 15000 eq. of TFA (trifluoroacetic acid, CF_3COOH) and then added TEA (triethylamine, Et_3N) up to 6000 eq. to return to the spectrum shape without TFA. Re-acidification by adding TFA up to 45000 eq.

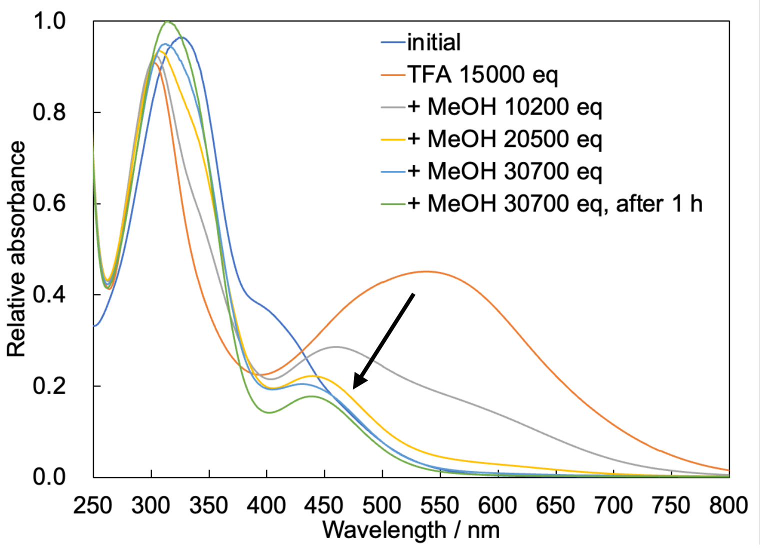

Figure S49. UV-Vis spectra of **[3]CAB-1** (0.02 mM 1,2-dichloroethane, 25°C). First acidification by 15000 eq. of TFA and then adding MeOH up to 30700 eq.

S13. ^{1}H NMR spectrum of [3]CAB-1 after UV light irradiation

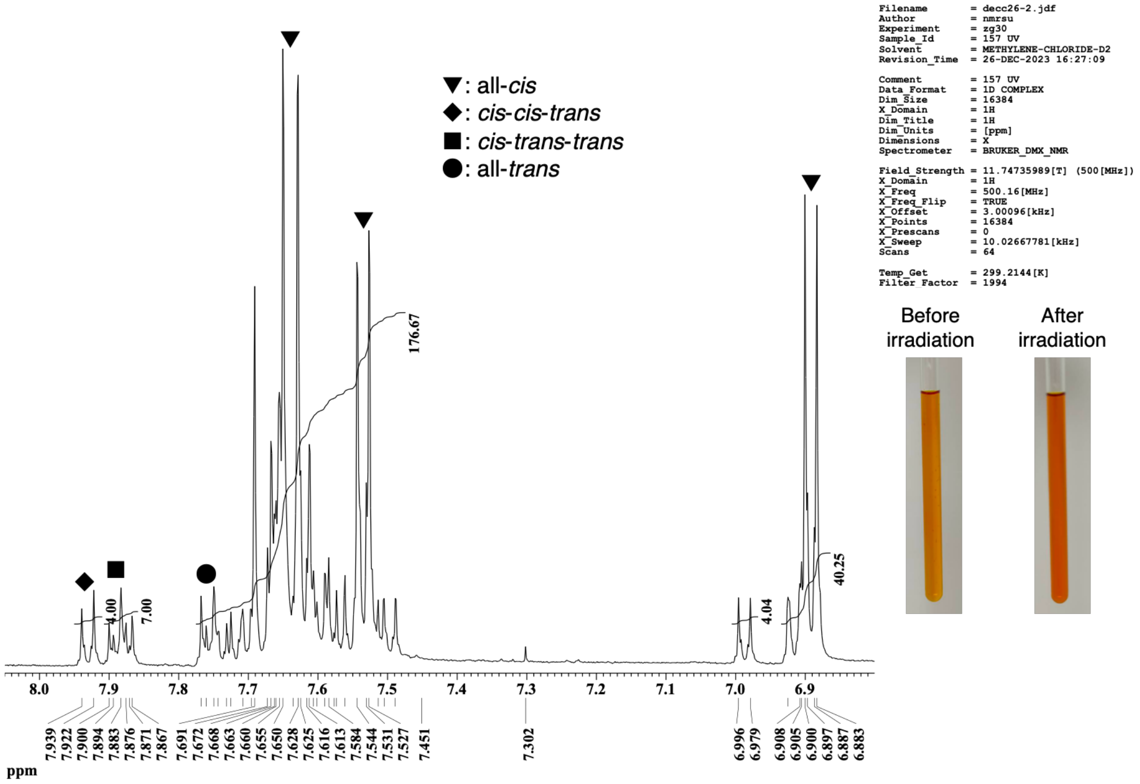

Figure S50. ^{1}H NMR spectrum (500 MHz, CD_2Cl_2, 298 K) of aromatic region of **[3]CAB-1**. Measured after photoirradiation at 254 nm for 30 min. Photos of solutions before and after irradiation at 254 nm are depicted. Signals are assigned based on the result of the DFT calculation (Table S34–S37).

S14. ¹H NMR spectra of tris-azo macrocycles in $CDCl_3$ containing 1.2 M CF_3COOH

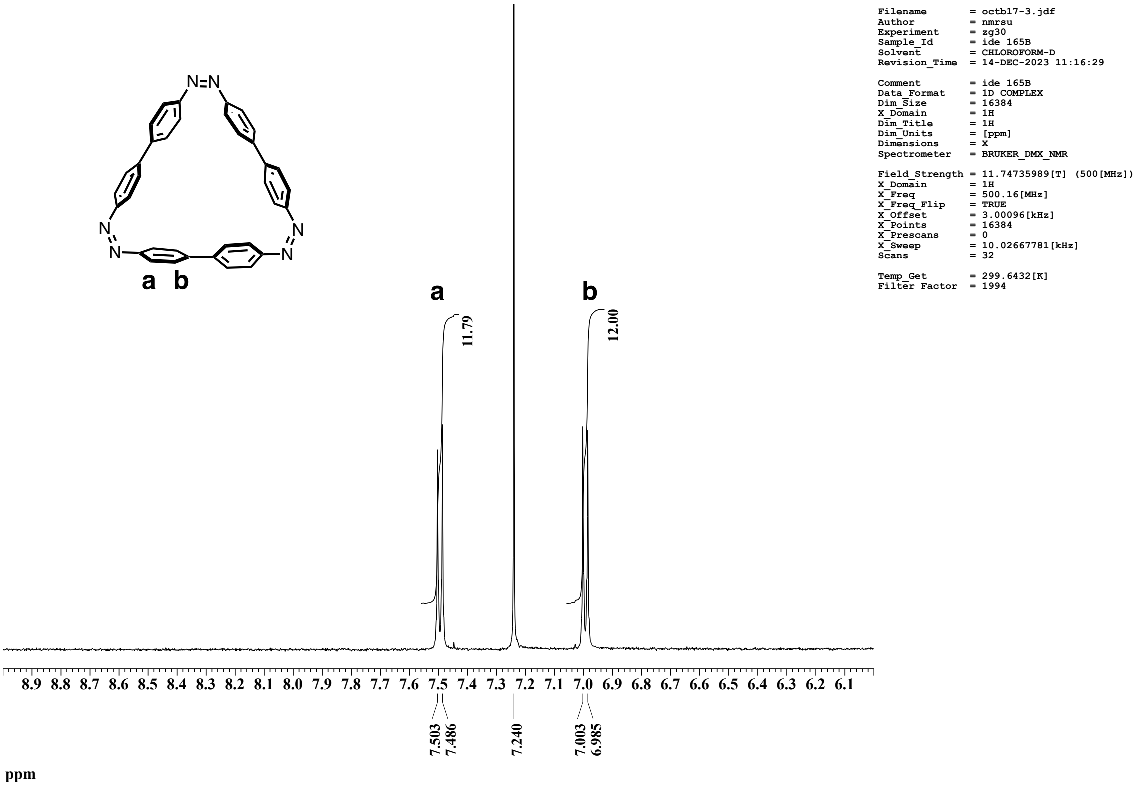

Figure S51. ¹H NMR spectrum (500 MHz, $CDCl_3$, 300 K) of the aromatic region of **[3]CAB-0** containing 1.2 M CF_3COOH (approximately 1300 eq.).

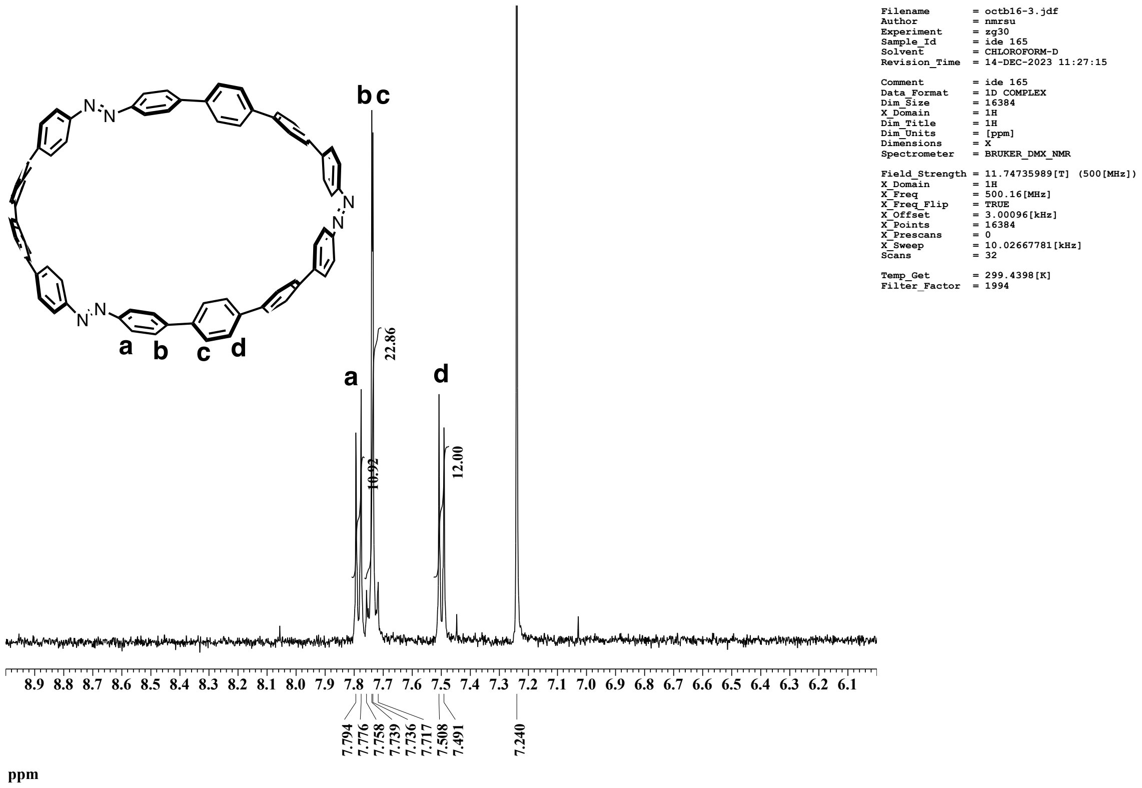

Figure S52. ^{1}H NMR spectrum (500 MHz, $CDCl_3$, 299 K) of the aromatic region of **[3]CAB-1** containing 1.2 M CF_3COOH (approximately 1300 eq.).

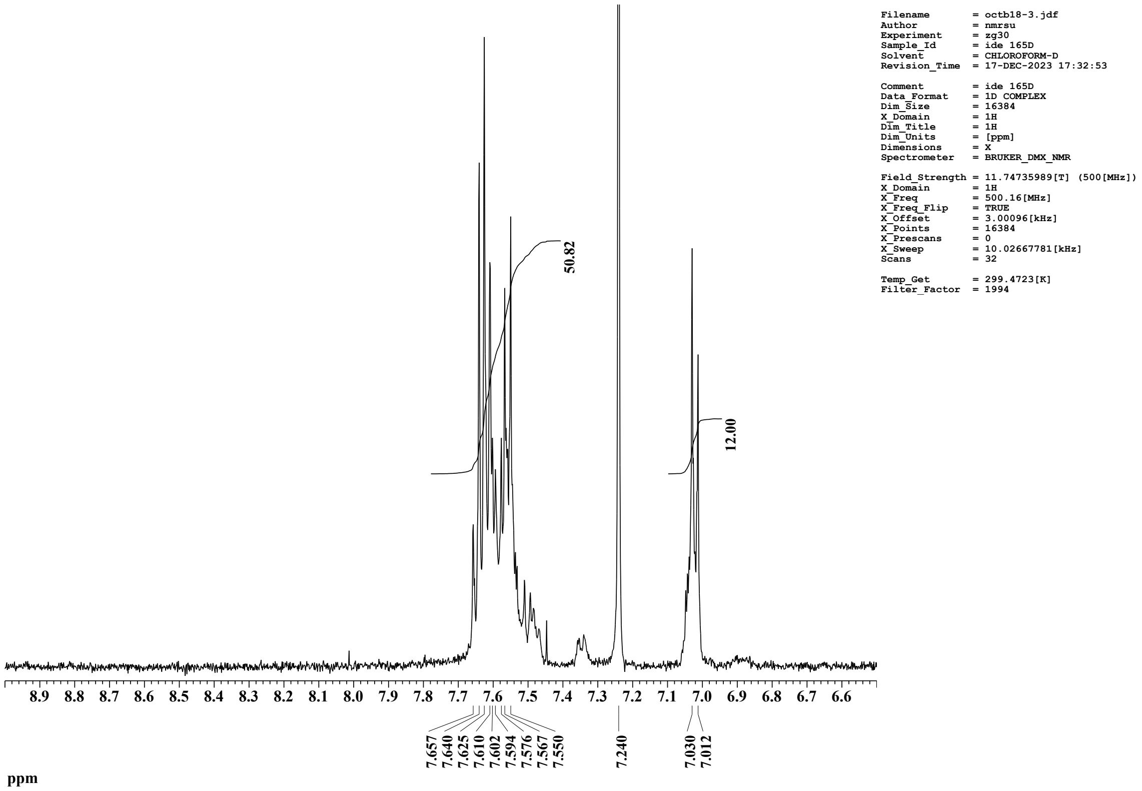

Figure S53. ^{1}H NMR spectrum (500 MHz, $CDCl_3$, 299 K) of aromatic region of **[3]CAB-1** containing 40 equivalents of CF_3COOH.

S15. Computational Details

All DFT calculations were performed using the ORCA software version 5.0.4.[7,8] Geometry optimization calculations were performed using the r^{2}SCAN-3c[9] composite method. The optimized structures were verified as minima by frequency calculations. Single-point energies for the optimized structure were obtained using the ωB97M-V[10]/def2-QZVP[11] level of theory. Time-dependent DFT (TD-DFT) calculations using the Tamm-Dancoff approximation (TDA-DFT) were used to evaluate the photophysical properties at the SOS-ωPBEPP86[12]/cc-pVDZ[13] level of theory. Kohn-Sham orbital was calculated at the CAM-B3LYP[14]/def2-TZVP[11] level of theory. The NMR chemical shifts were predicted by the GAIO method at the revTPSS[15]/def2-TZVPP[11] level of theory with the CPCM solvation model ($CHCl_3$). The RIJCOSX approximation[16] with the def2/J[17] auxiliary basis was used for single-point and TDA-DFT calculations to reduce computational cost. The RI-MP2 approximation with the cc-pVDZ/C[18] auxiliary was also applied for TDA-DFT calculations. Equilibrium constant *K* was calculated ωB97M-V/(ma-)def2-QZVP//r^{2}SCAN-3c(+ma) with SMD solvation model[19] (1,2-dichloroethane) for single-point energy. Here, the "ma" means additional minimal *sp* diffuse function on oxygen and fluorine.[20] Gaussian function broaden UV-Vis spectra were calculated by Gabedit 2.5.1.[19] The geometries, frontier orbitals (isosurface = 0.02) and transition density (isosurface = 0.0002) were drawn with PyMOL 2.5.0.[20] Symmetry-adapted perturbation theory analysis was performed at the SAPT0[21]/jun-cc-pVDZ[22] level of theory[23] using PSI4 1.9.1.[24]

S16. Ring Strain and Relative Gibbs Free Energy

Table S4. Strain energies of tris-azo macrocycles estimated by homodesmotic reactions (ωB97M-V/def2-QZVP//r^{2}SCAN-3c).

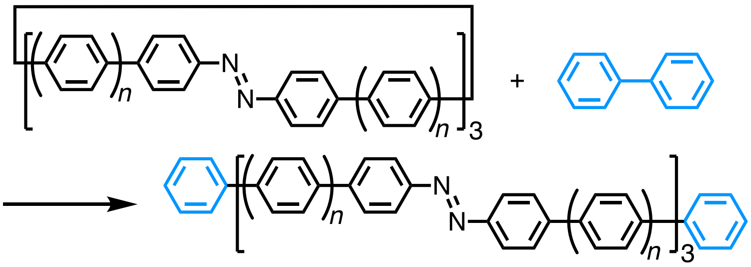

Compound	Strain energy / kcal mol^{-1}			
	All-*cis*	*cis*-*cis*-*trans*	*cis*-*trans*-*trans*	All-*trans*
**[3]CAB-0** (*n* = 0)	34.4	52.2	62.6	76.3
**[3]CAB-1** (*n* = 1)	34.7	40.6	40.7	42.0

Table S5. Relative Gibbs free energies of tris-azo macrocycles (ωB97M-V/def2-QZVP//r^{2}SCAN-3c).

Compound	Gibbs free energy / kcal mol^{-1}			
	All-*cis*	*cis-cis-trans*	*cis-trans-trans*	All-*trans*
**[3]CAB-0** (*n* = 0)	0	17.9	28.2	76.3
**[3]CAB-1** (*n* = 1)	0	5.5	5.6	7.1

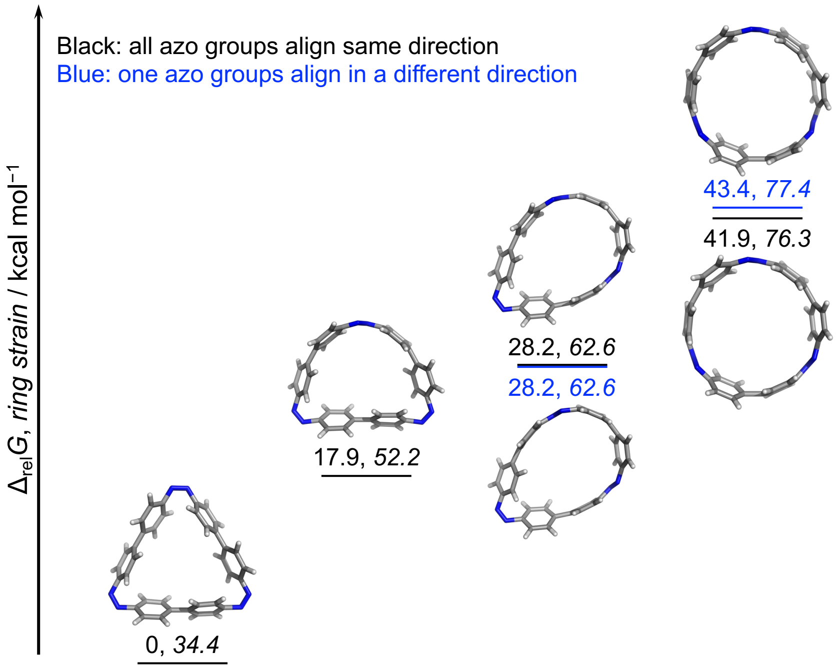

Figure S54. Relative energies and ring strains of all possible configurations of **[3]CAB-0** (ωB97M-V/def2-QZVP//r^{2}SCAN-3c).

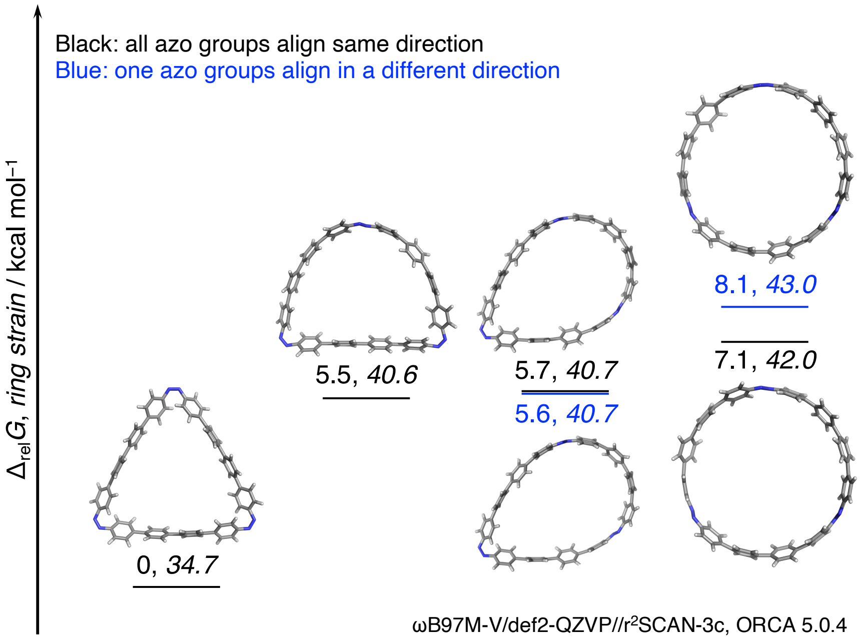

Figure S55. Relative energies and ring strains of all possible configurations of **[3]CAB-1** (ωB97M-V/def2-QZVP//r^{2}SCAN-3c).

S17. Transition Density of All-*cis* and All-*trans* [3]CAB-1

The transition density of the $S_0 \rightarrow S_1$ transition of **[3]CAB-1** indicated the contribution of the *n*-electron of the azo groups in the all-*cis* form, which seems to be the n-π* transition of azobenzene. In the all-trans form of **[3]CAB-1**, the transition density aligned the direction of π-orbital, indicating a higher π-π* nature in the all-*trans* form (Figure S56).

a) b)

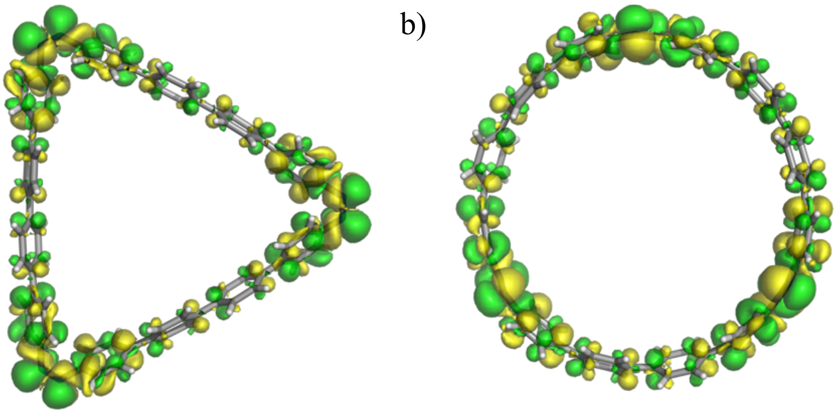

Figure S56. Transition density of a) all-*cis* **[3]CAB-1**, and b) all-*trans* **[3]CAB-1** (SOS-ωPBEPP86/def2-SVP//r^{2}SCAN-3c level of theory (not include perturbation term), isosurface = 0.0002).

S18. Predicted UV-Vis Absorption Spectra

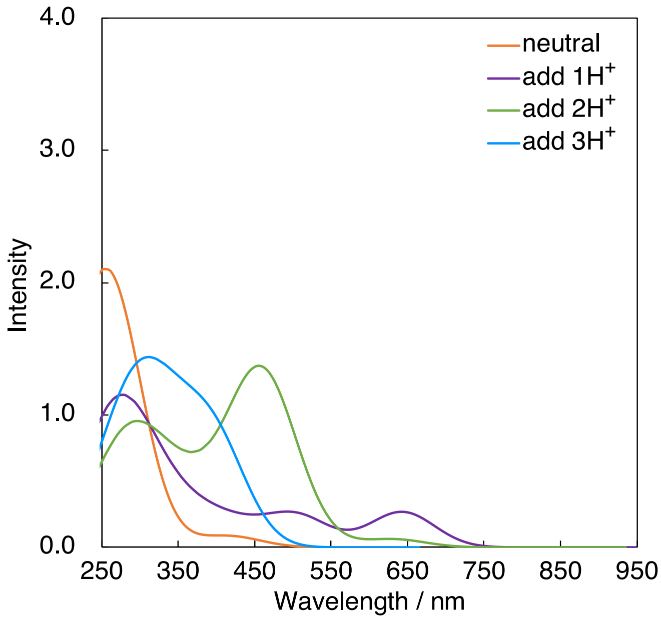

Figure S57. TDA-DFT predicted UV-Vis absorption spectra of all-*cis* **[3]CAB-0** and its protonated spices (add 1–3H^+). Including 24 states and broadened by Gaussian function with a half-width factor of 50 nm.

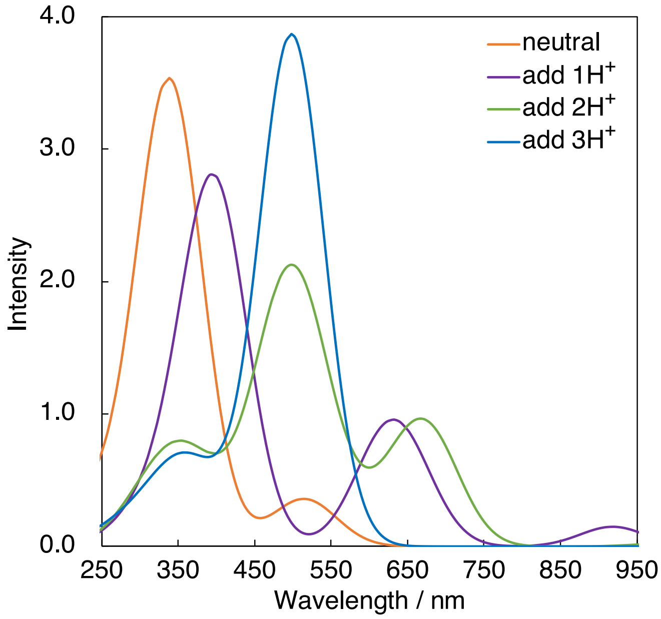

Figure S58. TDA-DFT predicted UV-Vis absorption spectra of all-*trans* **[3]CAB-0** and its protonated spices (add 1–3H^+). Including 24 states and broadened by Gaussian function with a half-width factor of 50 nm.

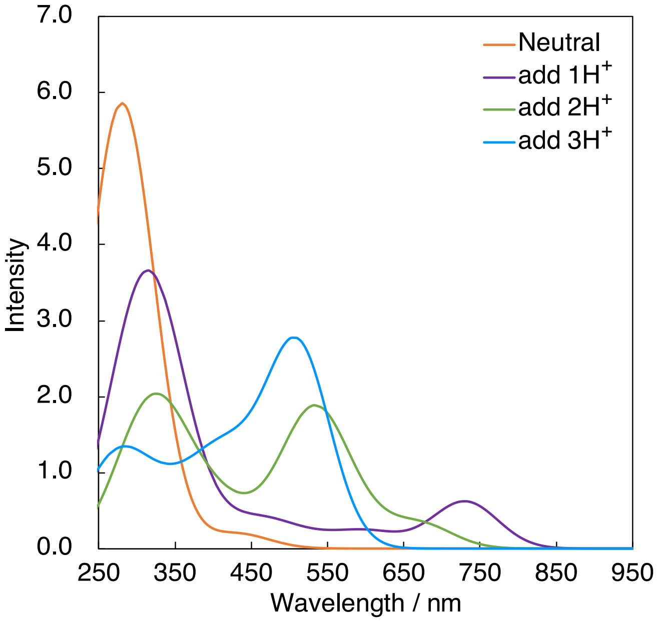

Figure S59. TDA-DFT predicted UV-Vis absorption spectra of all-*cis* **[3]CAB-1** and its protonated spices (add 1–3H$^+$). Including 24 states and broadened by Gaussian function with a half-width factor of 50 nm.

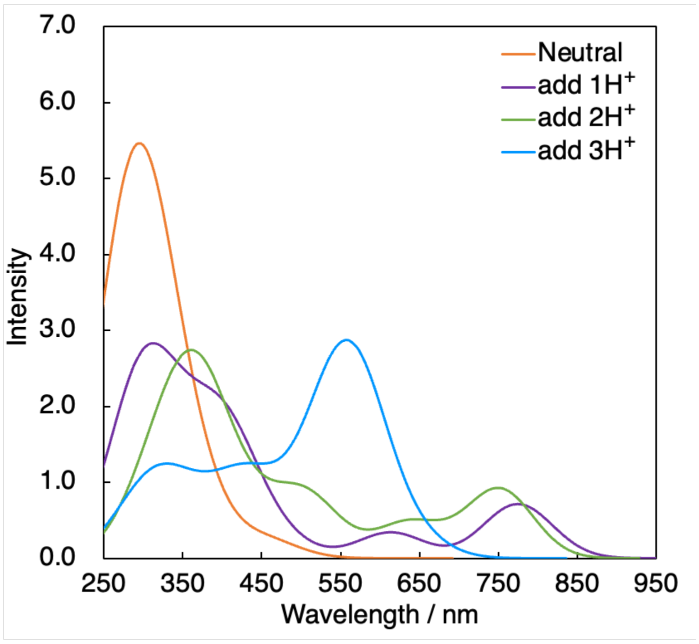

Figure S60. TDA-DFT predicted UV-Vis absorption spectra of *cis-cis-trans* **[3]CAB-1** and its protonated spices (add 1–3H$^+$). Including 24 states and broadened by Gaussian function with a half-width factor of 50 nm.

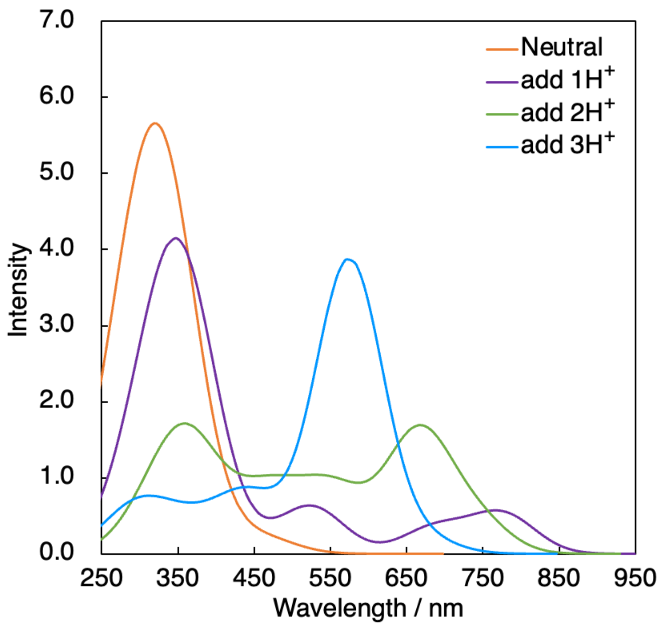

Figure S61. TDA-DFT predicted UV-Vis absorption spectra of *cis*-*trans*-*trans* **[3]CAB-1** and its protonated spices (add 1–3H^+). Including 24 states and broadened by Gaussian function with a half-width factor of 50 nm.

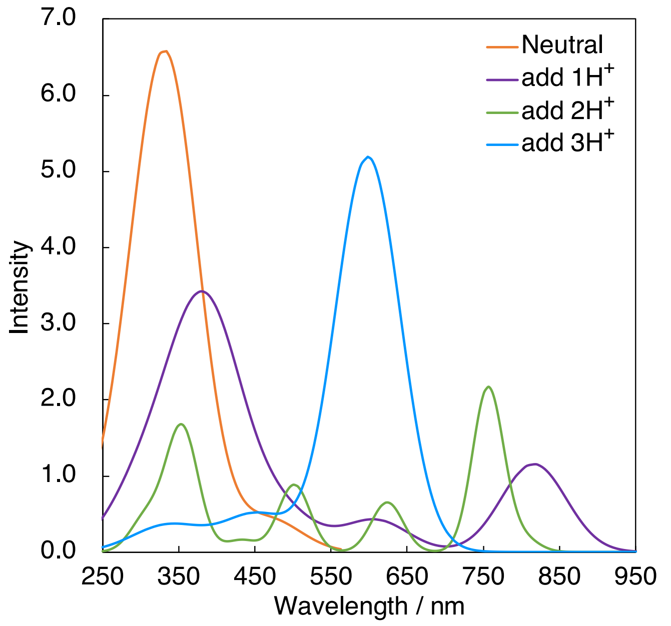

Figure S62. TDA-DFT predicted UV-Vis absorption spectra of all-*trans* **[3]CAB-1** and its protonated species (add 1–3H^+). Including 24 states and broadened by Gaussian function with a half-width factor of 50 nm.

S19. Detailed Result of TDA-DFT Calculations

This section provides a summary of the wavelengths and oscillator intensities for the 24 states. Configuration weights have been omitted, as most excitation configurations contribute less than 20%.

Table S6. Calculated 24 states of all-*cis* **[3]CAB-0**.

State	Wavelength / nm	f_{osc}
1	422	0.0073
2	414	0.040
3	414	0.040
4	281	0.0043
5	265	0.0089
6	265	0.0093
7	265	0.0024
8	263	0.0000067
9	261	0.80
10	261	0.80
11	260	0.018
12	260	0.019
13	253	0.0000028
14	249	0.14
15	249	0.14
16	224	0.029
17	224	0.028
18	218	0.025
19	215	0.0017
20	215	0.0017
21	212	0.00000073
22	210	0.11
23	210	0.10
24	209	0.00011

Table S7. Calculated 24 states of all-*cis* **[3]CAB-0** with one proton.

State	Wavelength / nm	f_{osc}
1	643	0.27
2	507	0.16
3	496	0.091
4	421	0.074
5	418	0.051
6	388	0.055
7	374	0.066
8	366	0.0033
9	363	0.0085
10	352	0.0032
11	342	0.042
12	342	0.00083
13	327	0.14
14	326	0.071
15	292	0.26
16	272	0.035
17	270	0.029
18	266	0.017
19	265	0.013
20	264	0.59
21	260	0.045
22	259	0.011
23	257	0.042
24	256	0.038

Table S8. Calculated 24 states of all-*cis* **[3]CAB-0** with two protons.

State	Wavelength / nm	f_{osc}
1	630	0.062
2	491	0.29
3	455	0.67
4	451	0.44
5	391	0.048
6	325	0.019
7	364	0.014
8	377	0.15
9	355	0.060
10	348	0.0076
11	344	0.12
12	338	0.041
13	350	0.022
14	296	0.33
15	282	0.14
16	289	0.0044
17	275	0.14
18	293	0.045
19	282	0.012
20	267	0.0068
21	272	0.031
22	281	0.012
23	293	0.0016
24	257	0.12

Table S9. Calculated 24 states of all-*cis* **[3]CAB-0** with three protons.

State	Wavelength / nm	f_{osc}
1	445	0.0028
2	396	0.39
3	396	0.39
4	366	0.012
5	366	0.012
6	350	0.0058
7	337	0.044
8	332	0.25
9	332	0.25
10	322	0.012
11	321	0.012
12	321	0.012
13	295	0.00022
14	288	0.46
15	288	0.46
16	263	0.0085
17	262	0.0020
18	262	0.0020
19	253	0.0027
20	253	0.0027
21	252	0.000041
22	238	0.00055
23	231	0.039
24	231	0.039

Table S10. Calculated 24 states of all-*trans* **[3]CAB-0**.

State	Wavelength / nm	f_{osc}
1	569	0.000088
2	515	0.18
3	514	0.18
4	402	0.00016
5	355	0.000000018
6	339	1.7
7	339	1.7
8	304	0.0013
9	304	0.0014
10	303	0.00000031
11	294	0.039
12	287	0.0016
13	287	0.0016
14	285	0.0029
15	285	0.0029
16	269	0.00050
17	269	0.00051
18	256	0.00031
19	256	0.00031
20	248	0.00056
21	248	0.00052
22	232	0.15
23	232	0.15
24	231	0.000000089

Table S11. Calculated 24 states of all-*trans* **[3]CAB-0** with one proton.

State	Wavelength / nm	f_{osc}
1	918	0.15
2	651	0.42
3	631	0.17
4	614	0.45
5	403	0.70
6	400	1.3
7	388	0.52
8	382	0.16
9	378	0.019
10	373	0.039
11	358	0.031
12	354	0.0094
13	353	0.027
14	348	0.021
15	348	0.011
16	338	0.0064
17	321	0.0045
18	312	0.018
19	309	0.0046
20	309	0.041
21	307	0.0040
22	305	0.16
23	298	0.00092
24	281	0.0089

Table S12. Calculated 24 states of all-*trans* **[3]CAB-0** with two protons.

State	Wavelength / nm	f_{osc}
1	986	0.021
2	678	0.77
3	632	0.31
4	523	1.0
5	482	1.4
6	398	0.000020
7	379	0.13
8	374	0.025
9	373	0.12
10	373	0.069
11	365	0.056
12	357	0.040
13	353	0.052
14	353	0.0048
15	334	0.022
16	331	0.015
17	331	0.19
18	330	0.040
19	311	0.060
20	310	0.061
21	298	0.011
22	298	0.027
23	289	0.0037
24	282	0.019

Table S13. Calculated 24 states of all-*trans* **[3]CAB-0** with three protons.

State	Wavelength / nm	f_{osc}
1	889	0.000028
2	543	0.0018
3	498	1.9
4	498	1.9
5	380	0.0015
6	380	0.0015
7	379	0.059
8	365	0.21
9	365	0.21
10	357	0.026
11	357	0.026
12	340	0.047
13	333	0.021
14	333	0.021
15	319	0.022
16	319	0.022
17	317	0.00063
18	316	0.0065
19	316	0.0065
20	315	0.043
21	294	0.025
22	294	0.025
23	263	0.042
24	263	0.042

Table S14. Calculated 24 states of all-*cis* **[3]CAB-1**.

State	Wavelength / nm	f_{osc}
1	433	0.0068
2	431	0.095
3	430	0.095
4	297	0.0017
5	281	2.9
6	280	2.9
7	268	0.0016
8	266	0.00079
9	266	0.00043
10	266	0.0013
11	265	0.000000079
12	264	0.018
13	264	0.018
14	264	0.0013
15	264	0.0013
16	263	0.021
17	263	0.021
18	261	0.00011
19	261	0.00010
20	261	0.00015
21	258	0.00000010
22	255	0.0050
23	255	0.0050
24	241	0.000018

Table S15. Calculated 24 states of all-*cis* **[3]CAB-1** with one proton.

State	Wavelength / nm	f_{osc}
1	731	0.60
2	684	0.031
3	603	0.21
4	531	0.11
5	503	0.0060
6	471	0.22
7	448	0.13
8	405	0.055
9	403	0.027
10	389	0.054
11	389	0.012
12	372	0.000047
13	366	0.0017
14	363	0.0022
15	362	0.0028
16	360	0.00043
17	357	0.054
18	356	0.0012
19	354	0.0042
20	339	0.72
21	336	0.20
22	317	1.3
23	314	0.49
24	288	1.2

Table S16. Calculated 24 states of all-*cis* **[3]CAB-1** with two protons.

State	Wavelength / nm	f_{osc}
1	672	0.31
2	593	0.24
3	542	0.88
4	523	0.68
5	516	0.17
6	512	0.12
7	428	0.28
8	409	0.096
9	401	0.10
10	371	0.013
11	369	0.0024
12	363	0.090
13	363	0.10
14	362	0.055
15	359	0.061
16	357	0.0017
17	347	0.21
18	342	0.018
19	342	0.0081
20	338	0.16
21	330	0.20
22	321	0.032
23	312	0.87
24	301	0.41

Table S17. Calculated 24 states of all-*cis* **[3]CAB-1** with three protons.

State	Wavelength / nm	f_{osc}
1	551	0.0011
2	510	1.3
3	510	1.3
4	417	0.53
5	417	0.53
6	409	0.0000044
7	376	0.038
8	372	0.077
9	372	0.077
10	344	0.17
11	344	0.17
12	343	0.0020
13	341	0.017
14	340	0.0045
15	340	0.00088
16	332	0.0021
17	332	0.0023
18	330	0.0031
19	326	0.022
20	325	0.022
21	321	0.0096
22	291	0.00015
23	273	0.59
24	273	0.59

Table S18. Calculated 24 states of *cis*-*cis*-*trans* **[3]CAB-1**.

State	Wavelength / nm	f_{osc}
1	462	0.10
2	446	0.083
3	440	0.084
4	350	1.3
5	293	1.9
6	290	2.4
7	271	0.58
8	269	0.0022
9	285	0.015
10	285	0.0038
11	263	0.027
12	271	0.0015
13	266	0.0075
14	268	0.0077
15	265	0.015
16	262	0.021
17	267	0.00090
18	261	0.0039
19	262	0.00014
20	257	0.00039
21	263	0.0014
22	262	0.00053
23	261	0.000063
24	247	0.033

Table S19. Calculated 24 states of *cis-cis-trans* **[3]CAB-1** with one proton.

State	Wavelength / nm	f_{osc}
1	769	0.57
2	795	0.17
3	478	0.14
4	453	0.096
5	614	0.34
6	608	0.0045
7	388	0.73
8	405	0.78
9	433	0.075
10	421	0.10
11	375	0.11
12	387	0.015
13	353	0.017
14	299	1.9
15	366	0.012
16	309	0.34
17	363	0.0043
18	352	0.15
19	375	0.00039
20	364	0.0150
21	306	0.24
22	338	0.072
23	364	0.00033
24	355	0.00089

Table S20. Calculated 24 states of *cis-cis-trans* **[3]CAB-1** with two protons.

State	Wavelength / nm	f_{osc}
1	745	0.25
2	754	0.67
3	492	0.29
4	635	0.12
5	638	0.36
6	514	0.49
7	482	0.12
8	364	1.4
9	423	0.40
10	405	0.0091
11	401	0.11
12	360	0.16
13	372	0.22
14	347	0.062
15	349	0.092
16	349	0.041
17	311	0.76
18	376	0.021
19	371	0.20
20	376	0.011
21	364	0.0020
22	376	0.0015
23	371	0.0010
24	333	0.012

Table S21. Calculated 24 states of *cis-cis-trans* **[3]CAB-1** with three protons.

State	Wavelength / nm	f_{osc}
1	641	0.26
2	570	2.0
3	527	1.1
4	436	0.60
5	432	0.31
6	406	0.15
7	339	0.32
8	372	0.055
9	374	0.093
10	352	0.031
11	347	0.028
12	354	0.0084
13	360	0.019
14	345	0.013
15	328	0.018
16	349	0.0039
17	313	0.039
18	329	0.0022
19	342	0.0044
20	335	0.011
21	330	0.012
22	314	0.16
23	331	0.044
24	298	0.52

Table S22. Calculated 24 states of *cis*-*trans*-*trans* **[3]CAB-1**.

State	Wavelength / nm	f_{osc}
1	468	0.13
2	444	0.074
3	440	0.038
4	360	0.50
5	335	3.5
6	295	1.9
7	276	0.67
8	276	0.070
9	285	0.0020
10	286	0.0097
11	267	0.028
12	281	0.0098
13	282	0.00084
14	267	0.0060
15	270	0.0020
16	265	0.014
17	268	0.0011
18	265	0.0017
19	263	0.0083
20	254	0.042
21	265	0.0079
22	263	0.0011
23	262	0.0055
24	248	0.016

Table S23. Calculated 24 states of *cis*-*trans*-*trans* **[3]CAB-1** with one proton.

State	Wavelength / nm	f_{osc}
1	775	0.52
2	529	0.27
3	694	0.035
4	685	0.32
5	454	0.031
6	490	0.026
7	522	0.34
8	376	0.82
9	345	2.6
10	388	0.088
11	385	0.16
12	386	0.0092
13	395	0.045
14	353	0.0040
15	295	0.76
16	348	0.13
17	362	0.017
18	351	0.037
19	301	0.24
20	373	0.0010
21	368	0.0024
22	361	0.00053
23	358	0.0015
24	359	0.00074

Table S24. Calculated 24 states of *cis-trans-trans* **[3]CAB-1** with two protons.

State	Wavelength / nm	f_{osc}
1	740	0.45
2	664	1.6
3	490	0.26
4	556	0.70
5	541	0.13
6	457	0.50
7	468	0.13
8	358	0.79
9	369	0.36
10	383	0.091
11	373	0.011
12	389	0.029
13	353	0.053
14	378	0.0070
15	362	0.088
16	351	0.014
17	347	0.0077
18	331	0.038
19	345	0.030
20	328	0.018
21	301	0.099
22	311	0.24
23	347	0.0036
24	336	0.0088

Table S25. Calculated 24 states of *cis*-*trans*-*trans* **[3]CAB-1** with three protons.

State	Wavelength / nm	f_{osc}
1	672	0.19
2	582	2.8
3	557	1.2
4	443	0.41
5	443	0.34
6	421	0.011
7	385	0.082
8	362	0.098
9	373	0.026
10	360	0.027
11	352	0.039
12	370	0.014
13	367	0.0018
14	352	0.026
15	320	0.0031
16	342	0.024
17	337	0.010
18	345	0.0081
19	343	0.021
20	295	0.031
21	322	0.044
22	331	0.013
23	331	0.013
24	291	0.53

Table S26. Calculated 24 states of all-*trans* **[3]CAB-1**.

State	Wavelength / nm	f_{osc}
1	473	0.00079
2	468	0.21
3	468	0.21
4	360	0.0013
5	334	3.1
6	334	3.1
7	286	0.00028
8	286	0.00028
9	285	0.0030
10	285	0.0030
11	283	0.36
12	283	0.36
13	280	0.015
14	278	0.000039
15	272	0.0000030
16	271	0.0016
17	271	0.0016
18	269	0.00000015
19	267	0.000017
20	267	0.0000092
21	266	0.0051
22	259	0.027
23	249	0.011
24	249	0.011

Table S27. Calculated 24 states of all-*trans* **[3]CAB-1** with one proton.

State	Wavelength / nm	f_{osc}
1	818	0.98
2	804	0.18
3	619	0.12
4	604	0.31
5	493	0.18
6	489	0.26
7	432	0.39
8	398	1.2
9	387	0.69
10	377	0.016
11	371	0.0031
12	370	0.019
13	364	0.0033
14	364	0.0040
15	363	0.013
16	363	0.063
17	362	1.3
18	361	0.0047
19	360	0.0051
20	357	0.0033
21	357	0.0011
22	316	0.23
23	300	0.41
24	294	0.22

Table S28. Calculated 24 states of all-*trans* **[3]CAB-1** with two protons.

State	Wavelength / nm	f_{osc}
1	802	0.17
2	756	2.2
3	629	0.24
4	621	0.42
5	506	0.40
6	499	0.46
7	487	0.034
8	435	0.037
9	432	0.12
10	383	0.02
11	381	0.011
12	376	0.0068
13	372	0.022
14	366	0.031
15	357	0.0091
16	356	0.013
17	355	1.4
18	353	0.0051
19	347	0.0063
20	344	0.040
21	343	0.043
22	338	0.043
23	334	0.10
24	306	0.42

Table S29. Calculated 24 states of all-*trans* **[3]CAB-1** with three protons.

State	Wavelength / nm	f_{osc}
1	711	0.0024
2	598	2.6
3	598	2.6
4	455	0.0052
5	452	0.24
6	452	0.24
7	362	0.010
8	362	0.010
9	359	0.0035
10	358	0.0075
11	357	0.0071
12	357	0.015
13	357	0.032
14	357	0.020
15	355	0.051
16	354	0.0066
17	354	0.0066
18	352	0.0032
19	345	0.00035
20	336	0.026
21	336	0.026
22	318	0.078
23	318	0.078
24	304	0.011

S20. Predicted ^{1}H NMR Chemical Shifts

Table S30. Predicted chemical shift of all-*cis* **[3]CAB-0** in $CHCl_3$ at the CPCM($CHCl_3$)-revTPSS/def2-TZVPP//r^{2}SCAN-3c level of theory.

Proton	δ (ppm)
a	6.62
b	7.02

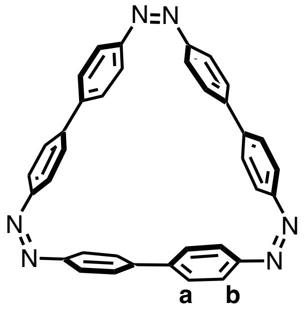

Table S31. Predicted chemical shift of all-*trans* **[3]CAB-0** in $CHCl_3$ at the CPCM($CHCl_3$)-revTPSS/def2-TZVPP//r^{2}SCAN-3c level of theory.

Proton	δ (ppm)
a	6.20
b	6.41

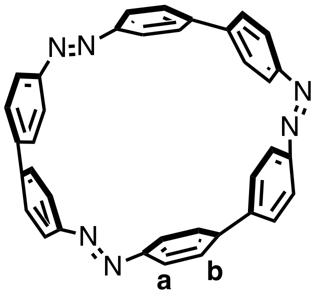

Table S32. Predicted chemical shift of all-*cis* **[3]CAB-1** in $CHCl_3$ at the CPCM($CHCl_3$)-revTPSS/def2-TZVPP//r^{2}SCAN-3c level of theory.

Proton	δ (ppm)
a	7.00
b	7.55
c	7.69
d	7.70

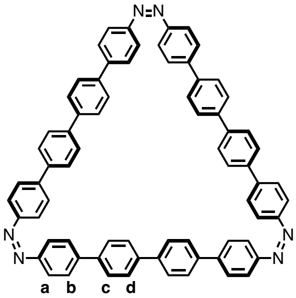

Table S33. Predicted chemical shift of all-*trans* **[3]CAB-1** in $CHCl_3$ at the CPCM($CHCl_3$)-revTPSS/def2-TZVPP//r^{2}SCAN-3c level of theory.

Proton	δ (ppm)
a	7.83
b	7.66
c	7.63
d	7.55

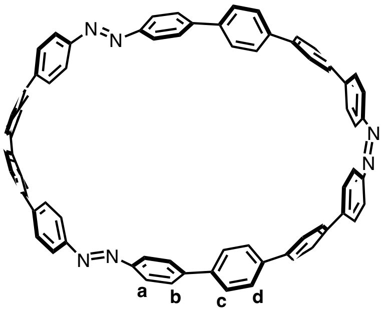

Table S34. Predicted chemical shift of all-*cis* **[3]CAB-1** in CH_2Cl_2 at the CPCM(CH_2Cl_2)-revTPSS/def2-TZVPP//r^{2}SCAN-3c level of theory.

Proton	δ (ppm)
a	6.99
b	7.55
c	7.68
d	7.70

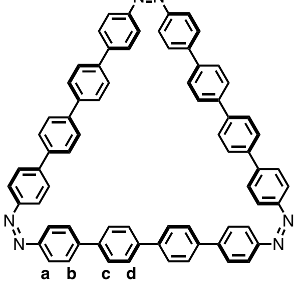

Table S35. Predicted chemical shift of *cis-cis-trans* **[3]CAB-1** in CH_2Cl_2 at the CPCM(CH_2Cl_2)-revTPSS/def2-TZVPP//r^{2}SCAN-3c level of theory.

Proton	δ (ppm)
a	7.92
b	7.64
c	7.38
d	6.98
e	6.86
f	7.29

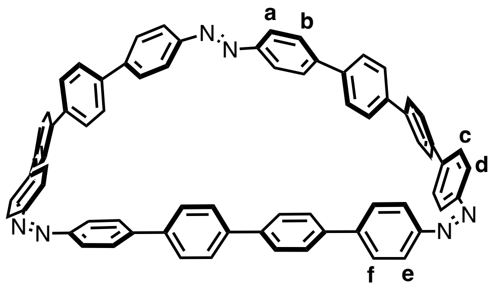

Table S36. Predicted chemical shift of *cis-trans-trans* **[3]CAB-1** in CH_2Cl_2 at the CPCM(CH_2Cl_2)-revTPSS/def2-TZVPP//r^{2}SCAN-3c level of theory.

Proton	δ (ppm)
a	6.93
b	7.37
c	7.63
d	7.88
e	7.88
f	7.70

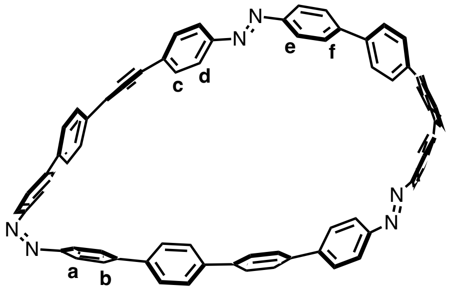

Table S37. Predicted chemical shift of all-*trans* **[3]CAB-1** in CH_2Cl_2 at the CPCM(CH_2Cl_2)-revTPSS/def2-TZVPP//r^{2}SCAN-3c level of theory.

Proton	δ (ppm)
a	7.81
b	7.66
c	7.62
d	7.55

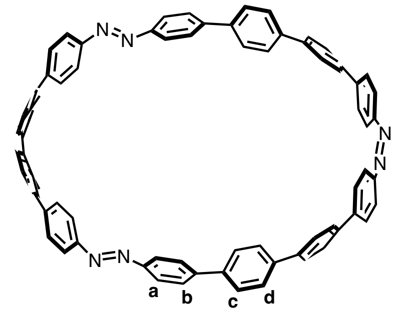

S21. Frontier orbitals of [3]CAB-1

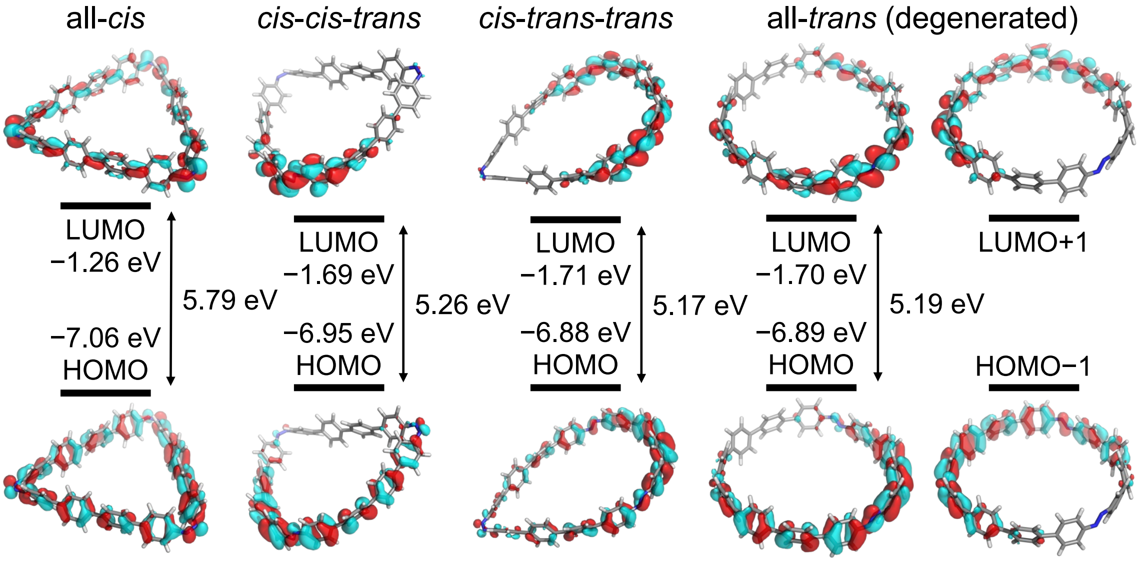

Figure S63. Frontier orbitals of **[3]CAB-1** (CAM-B3LYP/def2-TZVP//r^{2}SCAN-3c, isosurface = 0.02)).

S22. Double-protonated State of [3]CAB-1

Evaluating phenomena in solutions containing low concentrations of acids (in this case, ca. 0.3 M in UV-Vis measurements) by DFT calculations is challenging due to the low proportion of protons compared to the solvent. Therefore, we conducted calculations considering single-protonated state and a hypothetic double-protonated state (with protons bonded to one azo group) of both the all-*cis* and *cis*-*cis*-*trans* **[3]CAB-1** to elucidate *cis*-*trans* isomerization. In the case of the single-protonated state, the all-*cis* form was favored (*cis*-*cis*-*trans* form: $\Delta_{\text{rel}}G$ = +6.3 kcal mol^{-1} at the ωB97M-V/def2-QZVP//r^{2}SCAN-3c level of theory). However, in the double-protonated state, the energy difference between the two states was almost negligible (*cis*-*cis*-*trans* form: $\Delta_{\text{rel}}G$ = +0.26 kcal mol^{-1}), and a twisted form resembling the *trans* form was observed (Figure S64). This structural change is attributed to repulsion between proton charges, resulting in a similar structure between the two forms with the difference in the direction of added protons.

The double-protonated state *cis*-*cis*-*trans* (*cis* protonated) and *cis*-*trans*-*trans* (*trans* protonated) are also similar structures, with an energy difference of 2.5 kcal mol^{-1}. Also, the structure of double-protonated *cis*-*trans*-*trans* (*cis* protonated) has almost the same energy as the *cis*-*trans*-*trans* (*trans* protonated), with a similar structure to the all-*trans* form with two protons on the same *trans* azo group. Note that all-*trans* is the most stable form with double-protonated states on the same azo group (see main text in Figure 4).

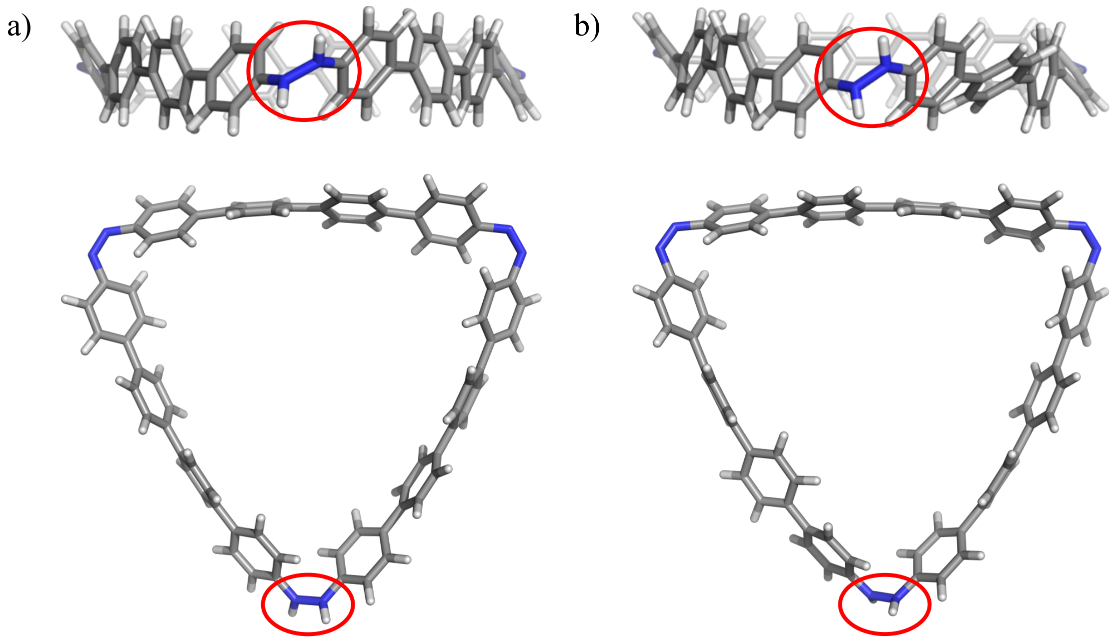

Figure S64. Side and top views of double-protonated a) all-*cis* and b) *cis*-*cis*-*trans* **[3]CAB-1** (r^2SCAN-3c).

The computational results suggest that *cis*-*trans* isomerization occurs *via* the double-protonated state. This finding is consistent with the requirement for a large excess of acid for *cis*-*trans* isomerization, considering the weak basicity of azobenzene.

Here, we consider the following equilibrium for **[3]CAB-1** and CF_3COOH:

$$[\mathbf{3}]\mathbf{CAB\text{-}1} + 2CF_3COOH \rightleftharpoons \left[[\mathbf{3}]\mathbf{CAB\text{-}1}\text{-}2H\right]^{2+} + 2CF_3COO^- \qquad \text{(S1)}$$

The equilibrium constant *K* is represented by:

$$K = \frac{\left[\left[[\mathbf{3}]\mathbf{CAB\text{-}1}\text{-}2H\right]^{2+}\right]\left[CF_3COO^-\right]^2}{\left[[\mathbf{3}]\mathbf{CAB\text{-}1}\right]\left[CF_3COOH\right]^2}$$

where,

$$\left[\left[[\mathbf{3}]\mathbf{CAB\text{-}1}\text{-}2H\right]^{2+}\right] = \left[CF_3COO^-\right] = x$$

$$\left[[\mathbf{3}]\mathbf{CAB\text{-}1}\right] = 2 \times 10^{-5}\ M$$

$$\left[CF_3COOH\right] = 2 \times 10^{-5} \times 15000\ M$$

The initial stage of protonation contains only all-*cis* **[3]CAB-1**. The Gibbs free energy of the reaction (eq. S1) in the case of double-protonated all-*cis* **[3]CAB-1** is 91.7 kcal mol^{-1}, estimated by DFT calculations at the SMD(1,2-dichloroethane)-ωB97M-V/(ma-)def2-

QZVP//r^{2}SCAN-3c(+ma) level of theory. Therefore, the DFT estimated equilibrium constant K is 8.55×10^{-17}. The equation to be solved is:

$$8.55 \times 10^{-17} = \frac{x^3}{2 \times 10^{-5} \times \left(2 \times 10^{-5} \times 15000\right)^2}$$

The only real solution to this equation is:

$$x = 5.35 \times 10^{-8}\ \mathrm{M}$$

This corresponds to 0.3% **[3]CAB-1** in a solution with a concentration of $2{\times}10^{-5}$ M. Thus, the double-protonated state is expected in the presence of the large excess acid.

S23. Cartesian Coordinates of Optimized Geometry

66

3CAB-0_all-cis, Eel(r2SCAN-3c) = -1714.264902127348 a.u.

C	-3.69927345550050	3.40289855513083	-0.48022606185220
C	-3.44240384575295	3.81202840695150	0.82768398232228
C	-3.80134623781259	2.04190027901433	-0.77960670420767
C	-3.59213881457908	1.10207454741039	0.21594208533205
C	-3.17442659113321	2.86244991926947	1.80359500189150
C	-3.23803597669841	1.49282099140850	1.51349520164225
H	-4.03802873439274	1.72763936151207	-1.79121302231679
H	-3.65069303388355	0.04395426025889	-0.02462397476266
H	-3.42433188528660	4.87281892513995	1.06121568615768
H	-2.94100229816738	3.18253662650390	2.81559231131257
N	-3.99954480121897	4.38989023239225	-1.47652867703730
N	-3.37012837275053	4.42532689486880	-2.55011448271148
H	-2.93073932474087	2.72964355234325	-4.53384659656007
C	-2.18650925067799	3.63679093400248	-2.73511268456038
C	-2.08985381333010	2.80542475057440	-3.85024672365892
C	-1.08643587653191	3.80308428242147	-1.88978630803871
C	0.07995395292690	3.09724953127746	-2.13355527671079
C	-0.93848371890653	2.05698281251048	-4.05049754699509
C	0.16262860676290	2.18485827796482	-3.19301553193906
H	-1.15285149120853	4.47946882932078	-1.04349176623304
H	0.92653484962976	3.21212900207058	-1.46196752135204
C	3.64061826994215	-0.27793246864233	-3.48655016741803
C	3.78331612940146	1.10599943721406	-3.39480433615517
C	2.36742513177020	-0.84391499384863	-3.59142880308761
C	1.24808542465538	-0.02943533864068	-3.54868985796406
C	2.65467562755226	1.90690511969426	-3.29154379533616
C	1.36909228705750	1.35314804242395	-3.36004797040434
H	2.26129614114847	-1.91866555183959	-3.69992849705160
H	0.25848092020993	-0.47440863186832	-3.60868934731115
H	4.77918160794883	1.53953855009041	-3.37387556065404
H	2.76658658704279	2.98275849395713	-3.18620912572445
N	4.81172981703948	-1.09533293076634	-3.61788179235117
N	5.01031789238072	-2.05453642896623	-2.84943047576751

H	3.69187963968664	-4.25656461971608	-2.20383274809543
C	4.20309374144640	-2.23797533439478	-1.67826397893199
C	3.59011882729291	-3.47116080350281	-1.46024303363642
C	4.14479339698387	-1.24520017195974	-0.69673245791603
C	3.43052009960267	-1.47195090697780	0.46794566927193
C	2.82679767130440	-3.66564956523119	-0.31767245473908
C	2.72853293102923	-2.66832936808684	0.66202056222986
H	4.65406218456873	-0.29964647188240	-0.85388293652103
H	3.36885062131091	-0.68997947565166	1.22002885256541
H	2.32137770620043	-4.61538293667577	-0.16392312843797
C	0.16711360601321	-2.98869804429394	4.06551046352534
C	1.49944759435393	-2.61642164684086	4.23968619800940
C	-0.29531912749626	-3.35963394833198	2.80022158097992
C	0.56012941962406	-3.30308476771686	1.71248747064355
C	2.33083758727444	-2.50464230966323	3.13416151064598
C	1.87432677934003	-2.83924610263749	1.85208545370110
H	-1.32339523038980	-3.68432082706025	2.67463901520847
H	0.19170153349628	-3.57132613659303	0.72596576525750
H	1.86357091346753	-2.38765749524241	5.23729547547950
H	3.36018197145482	-2.18188911149044	3.26591523787709
N	-0.67766088557343	-3.12541163822339	5.21635131830143
N	-1.77282805152238	-2.53656489930284	5.27812355092565
H	-3.97732509716985	-2.49735969215857	3.81461413043011
C	-2.12427152730044	-1.53387522805318	4.31477642254575
C	-3.33946445797801	-1.63576072450141	3.63875774823518
C	-1.32979683347215	-0.39536862505693	4.15660049932628
C	-1.72815933917735	0.60447183795026	3.28498275369056
C	-3.69929289423289	-0.65739820379675	2.72261259481214
C	-2.89772606383773	0.47512218502221	2.52527716084176
H	-0.87609437299070	1.38362722872275	-4.90125178009381
H	-4.63066203043053	-0.75380442036635	2.17093842710364
H	-0.40213120543488	-0.30248871514776	4.71243642923240
H	-1.09763483034037	1.47881666770724	3.14761656703496

67

3CAB-0_all-cis_1H, Eel(r2SCAN-3c) = -1714.643772776265 a.u.

C	-3.31952637550221	3.76782923646165	0.29030918482840
C	-2.91761325131562	4.14890156827111	1.56922324483856
C	-3.52549382351822	2.42356983400264	-0.03163387626871
C	-3.29399229129059	1.46008730508079	0.92741579189641
C	-2.64260513088574	3.16427856883779	2.50890805835193
C	-2.80749822515852	1.80527793397741	2.20012163594918
H	-3.86378638589676	2.14288560042513	-1.02405526081858
H	-3.44648216514965	0.41351461186158	0.68125652965641
H	-2.78268673862354	5.19887456677006	1.81513722135666
H	-2.30783007914820	3.45111251254028	3.50137166971436
N	-3.54168956019480	4.74490286992783	-0.72680293391207
N	-3.19222307089675	4.68402508672960	-1.96674091559186
H	-2.99563442925486	3.61724416172695	-4.28457818411330
C	-2.13338326166048	3.89090951438897	-2.32792022306009
C	-2.15350972891359	3.37654631346158	-3.64300538132595
C	-1.00224782080717	3.65660781044239	-1.50714166547332
C	0.04900135063351	2.92387790701411	-1.99495402510633
C	-1.14788298749299	2.53375534817436	-4.06753221387010
C	-0.02497234835907	2.28612086307913	-3.25357037544924
H	-0.93961173767928	4.09163796577015	-0.51614502003523
H	0.92668483575265	2.77009556360888	-1.37412484172334
C	3.01070972431341	-0.55449821976743	-4.09284257080264
C	3.37401659194108	0.73212191326020	-3.68473879379094
C	1.67380988729374	-0.85480980608998	-4.38527848653103
C	0.70126348442889	0.10607253705007	-4.18975106587731
C	2.38515217965630	1.65705922389175	-3.40793104538154
C	1.03060522940905	1.35896245246893	-3.64432453560664
H	1.41363037131715	-1.84326878264736	-4.75080009924874
H	-0.33908397729445	-0.14049490055746	-4.38226446801796
H	4.42199540951532	0.97902082805827	-3.54488074676991
H	2.66286004434180	2.64026010022376	-3.03815859061691
N	3.99669834864985	-1.53849911381959	-4.38694637603110
N	4.16347528495463	-2.54977230665856	-3.69615416403609
H	2.62084387850741	-4.54856182776192	-2.89465365409899

C	3.53070657436030	-2.66013643319380	-2.41578185278938
C	2.76056876435144	-3.78773828528294	-2.13259328297193
C	3.78932741957022	-1.71316227115663	-1.42145647056830
C	3.23592909039740	-1.87598004203902	-0.16207002515832
C	2.16239490721924	-3.90739469417292	-0.88605338582580
C	2.38887606123017	-2.95502691694040	0.11807890097439
H	4.44576596216528	-0.87430297007089	-1.62620438617350
H	3.43731795236133	-1.13889903252190	0.61033895762321
H	1.54325659971800	-4.77397041121508	-0.67141736509120
C	0.47148382445089	-3.10867237817651	3.93039242267826
C	1.85190707466374	-2.92217082133627	3.86157874859921
C	-0.26638784932637	-3.34116472532204	2.76667994202442
C	0.37351129122631	-3.33561872481036	1.53754064010823
C	2.47108894148910	-2.85735900982727	2.62197948516689
C	1.74266090871783	-3.05904032575711	1.44105020779988
H	-1.33051441618010	-3.54360204192186	2.82920118753300
H	-0.20280361614604	-3.51165174426475	0.63327885969369
H	2.42333205536804	-2.80959975812130	4.77819909872069
H	3.54307456298331	-2.68887237375917	2.56761154663146
N	-0.14700333869619	-3.20035696890223	5.21954927998080
N	-1.13250141384537	-2.50427057720066	5.50170764667418
H	-3.50436847832577	-2.28099146780198	4.31262745856173
C	-1.56171818947816	-1.42725960356850	4.66723088918099
C	-2.85045094167500	-1.43328593426552	4.13076087108400
C	-0.74199382303836	-0.30291638229673	4.52429340126274
C	-1.18772113052711	0.77744570511865	3.78373346858203
C	-3.25788646464783	-0.37937485091152	3.33056298499822
C	-2.43001834330779	0.73704492883516	3.13511690785100
H	-1.19357389039852	2.09814895856681	-5.06120757855715
H	-4.24384956646584	-0.40449241231548	2.87420209645972
H	0.24093255560239	-0.29070910187189	4.98481415310128
H	-0.53656313261730	1.63667598755028	3.64739062472227
H	-4.21008318287153	5.48667343875082	-0.53052325591069

68

3CAB-0_all-cis_2H, Eel(r2SCAN-3c) = -1714.953900100772 a.u.

C	-2.93751586607447	3.80052543503835	0.38245041474027
C	-3.06937443587923	4.07992774635210	1.75024182976371
C	-2.71019357229363	2.48639630337371	-0.05355700869458
C	-2.56921993338398	1.48877268871467	0.88001246437294
C	-2.89710656042014	3.06850016882160	2.67702173589020
C	-2.62009228614686	1.74983225110958	2.26810195730157
H	-2.66189631606692	2.24404031988664	-1.10772699513394
H	-2.37947262754538	0.48017366633344	0.52998525108269
H	-3.29115981720782	5.09009111872293	2.08664296842744
H	-3.00585932063933	3.30097089877387	3.73115220488017
N	-3.13930563335018	4.86138233768609	-0.52658703033119
N	-2.80087640898326	5.02379093010131	-1.74586970532835
H	-2.87564429108445	4.05392216671306	-4.14426146904818
C	-1.79263199508746	4.23597584775393	-2.29031603627692
C	-1.97596500756727	3.77694440443495	-3.60375203482277
C	-0.58823943735959	3.97509773335983	-1.61013586593923
C	0.36812752953734	3.18850688260953	-2.21327487728416
C	-1.04504991369844	2.91119902302659	-4.15645474773236
C	0.12531329544180	2.58093754984542	-3.45971382757859
H	-0.40592643484167	4.40167806613452	-0.62869046952859
H	1.29932026900936	2.98870696336803	-1.69059992056026
C	2.75394273295214	-0.59236290327054	-4.44680697669849
C	3.30378130051130	0.64900013550057	-4.14192342888540
C	1.38220239857087	-0.76524438853631	-4.60518053033932
C	0.54004297902455	0.30932384599517	-4.36535405368519
C	2.44724873145587	1.71042076958265	-3.87556543087134
C	1.05650011563415	1.54203396955118	-3.94137840159613
H	0.97868807642584	-1.73742220135206	-4.87240328091822
H	-0.53421965997707	0.17185181055232	-4.44640152573287
H	4.37981807766587	0.77203038369418	-4.05966959487747
H	2.86463670431266	2.67751573788418	-3.61032897847021
N	3.59536657715680	-1.76437641454798	-4.52441994414868
N	3.68551734221844	-2.72813731639062	-3.68599037139078
H	3.63284674962602	-4.82119295903148	-2.35923071487926

C	3.13824611576045	-2.72356292847409	-2.44096227248825
C	3.18904067430133	-3.99761455723847	-1.80834150926617
C	2.61235003068885	-1.62280122165559	-1.70474634617075
C	2.17719358222887	-1.81411554129855	-0.42216734768432
C	2.68455093823613	-4.18309741313912	-0.54260336970113
C	2.17094494239988	-3.09243332478066	0.19103017490699
H	2.57898979933332	-0.62609252412851	-2.11933918509884
H	1.77951109220684	-0.96514481847626	0.12394210453270
H	2.73854261969454	-5.16651704588602	-0.08671852929974
C	0.32326028167441	-3.32491122197638	4.00354511711088
C	1.09929855460570	-2.20464564284295	3.66998911268735
C	0.30687419066902	-4.44898244624765	3.16884952263558
C	0.93550562799699	-4.39024203256357	1.93847628059236
C	1.72709212464108	-2.15772348987382	2.44552681089059
C	1.62063509428865	-3.22728733919492	1.53243148994216
H	-0.23929904183136	-5.33526113084384	3.47724154275745
H	0.85029840276313	-5.23523560354521	1.26199823883692
H	1.20533820420853	-1.38628555903954	4.37449033104931
H	2.33877602779234	-1.29453004179890	2.20246777486771
N	-0.33254895181754	-3.39797817268377	5.25943099455147
N	-1.05108328087614	-2.48859873109176	5.68620474596073
H	-2.39786748324547	-2.74149265542186	3.38102336430748
C	-1.50734424574903	-1.43447832030894	4.85234838683356
C	-2.19151787456366	-1.71333142151397	3.66151155618138
C	-1.36771071381464	-0.11537388543762	5.29660628492775
C	-1.75348033370419	0.92178384607569	4.46673671642516
C	-2.60583324084367	-0.67098088645935	2.85977485380717
C	-2.34367951209501	0.66776489651612	3.21320786563442
H	-1.21690323072605	2.50580518106188	-5.14938549444253
H	-3.15802028781533	-0.89909576367844	1.95373117704800
H	-0.90074420895178	0.07975086591115	6.25727902769796
H	-1.54973601743286	1.94183090317357	4.77734817451455
H	-3.75625989615440	5.60386246008383	-0.19126868238435
H	4.11326765419521	-1.93457140501386	-5.38472051787006

69

3CAB-0_all-cis_3H, Eel(r2SCAN-3c) = -1715.162777807318 a.u.

C	-3.36499930469205	3.83615662870477	0.30412644628025
C	-2.76755093369705	4.18919045570547	1.50912248194755
C	-3.80226105698641	2.53845462302793	0.04495718804244
C	-3.59035630477942	1.56152101176765	1.00375844159199
C	-2.50170695021851	3.18411639243406	2.43280125357295
C	-2.89280941980269	1.86182913352445	2.18117763853924
H	-4.31087664290858	2.30008713895812	-0.88446094460949
H	-3.93139240277632	0.54735526856344	0.81690961212414
H	-2.47482432138195	5.21588927062810	1.71146660483484
H	-2.00243345240012	3.43624112094869	3.36409281052075
N	-3.58895520141311	4.81733732065477	-0.73973678193521
N	-3.16018802400320	4.79105076263600	-1.92890980352055
H	-2.94262664697611	3.82110709136421	-4.27987602657829
C	-2.10576425268349	3.97973550391673	-2.29488807040779
C	-2.11898722354798	3.53370885679209	-3.63310245317295
C	-0.99451973834498	3.70676405253298	-1.46497863950588
C	0.05383543137572	2.97526291124457	-1.97196510164981
C	-1.11764488909468	2.68855731941492	-4.07895113689604
C	-0.02043213431851	2.39757434428549	-3.25445675374724
H	-0.92721562648187	4.12603797819865	-0.46833351302089
H	0.93392015200919	2.80340410019075	-1.35902623145382
C	2.99462882332724	-0.45067865431261	-4.11734850116996
C	3.38115453873981	0.86877889536857	-3.90949324282267
C	1.65508603595966	-0.83024378702280	-4.18032539012246
C	0.68077265171518	0.13420894882816	-3.98245778538936
C	2.39342535738932	1.81165438899756	-3.64597429180486
C	1.03925959982195	1.45013456171189	-3.66161564678968
H	1.37980057852403	-1.85886892572629	-4.39356800732408
H	-0.36705342589406	-0.14699564935641	-4.03482693796304
H	4.42978160737392	1.15359472472860	-3.90944957009805
H	2.67999375229020	2.84010669934436	-3.44520865727330
N	3.97649473277779	-1.50043061285682	-4.30848255049269
N	4.12673460691741	-2.55142712299490	-3.62182703568465
H	3.39319222719832	-4.79670812021264	-2.65417280115776

C	3.58832762099624	-2.66620658275803	-2.35654179500847
C	3.24475112260650	-3.97544329561478	-1.95934429635273
C	3.50296868240706	-1.59776569723495	-1.43500366498173
C	3.05091078886172	-1.84938019082648	-0.16084376810560
C	2.66770303296844	-4.18097543284623	-0.71815869992942
C	2.56199885640060	-3.12142879902924	0.19537054659898
H	3.85627250140558	-0.60747081980128	-1.69600317685250
H	3.02956949643791	-1.04539763965055	0.56918514630532
H	2.34454670634978	-5.17801539302898	-0.43322270462064
C	0.49503412986278	-3.27921157167928	3.88997970454685
C	1.84869992154704	-2.96182170507258	3.91053406381471
C	-0.15763069688152	-3.67047378044224	2.72234134611653
C	0.55892588374878	-3.69735177805139	1.53724263428520
C	2.53673522080618	-2.92872441750900	2.70242271523392
C	1.89511765903140	-3.27704864990461	1.50585809420964
H	-1.20374768475913	-3.96123731873131	2.74282682028650
H	0.06382832952163	-4.00587224127837	0.62103080521070
H	2.35112280614233	-2.70351114419644	4.83875431948615
H	3.58592901090158	-2.64714309313626	2.69552205409529
N	-0.30360410688600	-3.24475506732116	5.09977656625831
N	-1.34356109607799	-2.55991876050360	5.31930931516652
H	-3.75666085770623	-1.83850684939466	4.90514183580584
C	-1.66044865921999	-1.46463924661756	4.54213648269757
C	-3.03775061179108	-1.18573222729787	4.41898193923964
C	-0.70335751914936	-0.57216936258790	4.00769855135516
C	-1.13591955325924	0.54999737748709	3.34077639339334
C	-3.44977734706681	-0.12489087263587	3.63119964423037
C	-2.50514751418704	0.75458702180913	3.08119147533860
H	-1.15750166309680	2.29284627553068	-5.08965549589526
H	-4.51055116436036	0.05562644971590	3.48329112326123
H	0.35557485170814	-0.72202286376147	4.18029789988257
H	-0.40921633285106	1.26747487717423	2.97076135237854
H	-4.29743701296348	5.53800017532700	-0.57520172550179
H	4.51523975957380	-1.49905407824524	-5.17918723555109
H	-0.12545470404002	-3.95916092987719	5.81129513073809

67
3CAB-0_all-trans_1H, Eel(r2SCAN-3c) = -1714.617652439912 a.u.

C	-3.77817920758882	3.40826472939178	-0.09869636412510
C	-3.53272417959372	3.93790674372723	1.18062524663456
C	-4.42795733623006	2.17038840228466	-0.22603200045648
C	-4.60704850785059	1.39285775524696	0.89542229932608
C	-3.70481168798008	3.13893819899792	2.29063678041936
C	-4.13307532988219	1.79915694022207	2.16436446695044
H	-4.67067186527455	1.78642260896080	-1.20956537567617
H	-4.99262988191764	0.38784298475555	0.76246138167881
H	-3.12864256670628	4.94154655484159	1.29122439900214
H	-3.44591491983457	3.53747578316879	3.26616277980437
N	-3.18176038635891	4.02460299335802	-1.19072865372875
N	-3.16275401504453	3.44405450921775	-2.36476336112580
H	-2.98657740297582	2.33060014527390	-4.64538779534455
C	-2.05484938188157	3.55549452423652	-3.12845844501530
C	-2.04067297286959	2.72092724568451	-4.28297230712963
C	-0.83487139935870	4.19957861756042	-2.76822085186694
C	0.34501558066851	3.78394008903727	-3.32576345150154
C	-0.85301165846267	2.31019157246291	-4.82566216776492
C	0.38633134249497	2.71373602012566	-4.26194910219326
H	-0.78850877196505	4.94274727768194	-1.97722077831403
H	1.27072832117584	4.19418658475487	-2.93723881762574
C	3.57891390310972	0.09192780830967	-3.57858217686256
C	3.83028054223231	1.48303733631369	-3.54666587426028
C	2.41743191199954	-0.37109780277633	-4.24184351078239
C	1.45526707489740	0.52153887946131	-4.63908822799306
C	2.85141870285997	2.36914737073584	-3.92771255947859
C	1.58488701898677	1.91023218443984	-4.37173691132827
H	2.24624607117026	-1.43904086097040	-4.32233039041952
H	0.52462484936931	0.13278451468058	-5.03829015272976
H	4.76455358285905	1.83321126023831	-3.11779456079333
H	3.03991786602207	3.43345056742158	-3.82888545758421
N	4.31814620208704	-0.67892165153394	-2.70544345101352
N	4.02877393607361	-1.91945557891057	-2.67031512529353
H	3.45920984238315	-4.38794652117568	-2.24319096199813

C	4.19050397733800	-2.52787034553551	-1.43929016083948
C	3.65952520370989	-3.83176262511529	-1.33216157791669
C	4.55708969000165	-1.84277833803336	-0.25763028739665
C	4.18542871464141	-2.34767989723654	0.96319790426060
C	3.27366820243148	-4.32020795350080	-0.10334590301303
C	3.41348142082211	-3.53270438122693	1.06172679193289
H	5.02613107731476	-0.86796669361864	-0.33272477422017
H	4.36509428794140	-1.74987410147748	1.85080336785222
H	2.79212244732056	-5.29133079697465	-0.04583047916231
C	0.50368152415997	-3.14308056851470	4.04686908090861
C	1.83915952385516	-2.82023306573359	4.36780868581106
C	0.25363339132016	-3.97340030240474	2.93165511248754
C	1.27139930704527	-4.26029106624425	2.05462982193381
C	2.84985846570857	-3.09978154367771	3.47433291928235
C	2.56696791563687	-3.71718355147240	2.23508966893564
H	-0.76532884315830	-4.27147923962238	2.71024055410627
H	1.03381216437293	-4.78923640769899	1.13736869445812
H	2.03443454542837	-2.25067662692559	5.27169307105170
H	3.86081407099593	-2.77459976644197	3.69944580964849
N	-0.46517217832369	-2.36049246724032	4.65702004259559
N	-1.65602921643780	-2.54639196083033	4.25532164455074
H	-4.14548658777782	-2.59274765393354	3.57065028101829
C	-2.47258124497918	-1.42547286571994	4.26300282676084
C	-3.74718527563299	-1.58835597974295	3.68077475701103
C	-1.99880998551944	-0.11651295443779	4.50322360755118
C	-2.66434830541547	0.95862926291044	3.96686492655610
C	-4.40265968127370	-0.50396814320513	3.13903941490674
C	-3.81347910050366	0.78013257017495	3.15888576849393
H	-0.86830138420384	1.60808008219451	-5.65262350442784
H	-5.34796767566157	-0.65969144264595	2.62840463655529
H	-1.04407391193029	0.01791982788797	4.99982058672007
H	-2.21245456665308	1.94155138149831	4.05224492234292
H	-2.51148871488156	4.75817819479279	-0.95861415136084

68

3CAB-0_all-trans_2H, Eel(r2SCAN-3c) = -1714.922327368700 a.u.

C	-3.82128834032806	3.36610924075137	-0.12448213958277
C	-3.58189633662829	3.91670997771572	1.14826319172919
C	-4.45869072271603	2.12021381184467	-0.24457787899974
C	-4.62573649322954	1.35109450942597	0.88327870305590
C	-3.73624804301320	3.12472825360535	2.26422083009863
C	-4.14236127830526	1.77606139228731	2.14492875604593
H	-4.71053889079885	1.72984060237188	-1.22351986326801
H	-5.01202542035015	0.34472153246128	0.76367858527536
H	-3.19997078271460	4.92997478557503	1.24898781092299
H	-3.48949862578882	3.53738188233096	3.23684512959970
N	-3.21449217138959	3.97402378121899	-1.21624243313126
N	-3.16472132957503	3.38908405086402	-2.37536741206821
H	-2.97011035935792	2.28665841033754	-4.66116918125561
C	-2.06956296774456	3.54873576332783	-3.15448208456507
C	-2.03586861730571	2.70675891324046	-4.30143994387003
C	-0.87309863574971	4.23551782048468	-2.79861169966770
C	0.31831843001256	3.84214494130577	-3.34613784061756
C	-0.83624211804736	2.33064742094194	-4.84635560959879
C	0.38690818044927	2.76240705738166	-4.27156799318645
H	-0.85155069572810	4.99612440380798	-2.02334697331640
H	1.22953328226919	4.28992051780128	-2.96575297548578
C	3.56853220863613	0.15511476928079	-3.55494623368761
C	3.81312559999855	1.53761500402168	-3.45033186381956
C	2.46333436869582	-0.30580938827494	-4.29115464224523
C	1.50883064640109	0.59748849035379	-4.69108177299269
C	2.83932699045897	2.42742347063893	-3.84302146808857
C	1.60187369698189	1.97402375933384	-4.36164904988017
H	2.31848197816395	-1.36831979498166	-4.44506109705575
H	0.61207002553101	0.20918388433889	-5.16002570098657
H	4.72882269842564	1.90253513748364	-2.99142730530624
H	3.02198915938411	3.48712191563184	-3.70129525409276
N	4.31417430601794	-0.70544579439094	-2.76482379898304
N	3.96226164743775	-1.95687243608707	-2.62693730304493
H	3.26141664136848	-4.36949027430561	-2.27018576477005

C	4.16109868492758	-2.57957081943263	-1.45071188887072
C	3.53206837421329	-3.85708172944413	-1.35219934785742
C	4.69018270727345	-2.00466345684147	-0.25477374442321
C	4.31712526515639	-2.51176002104619	0.95874657153809
C	3.15891100958156	-4.34682882027219	-0.13190314225796
C	3.39940043377019	-3.60050164269334	1.05439843012560
H	5.31143425049115	-1.11359033216236	-0.26994587912316
H	4.63143713459434	-1.99034366012788	1.85611769565652
H	2.61316051981951	-5.28296804876664	-0.08456557769837
C	0.52831102720017	-3.08301114746397	4.04603472628463
C	1.87723317892590	-2.77844896431095	4.35369334598875
C	0.25011851268432	-3.92984125899048	2.94357955329626
C	1.25157492621150	-4.25801723432896	2.06988226883032
C	2.86794708919575	-3.08280545874371	3.45388903917537
C	2.56233467316131	-3.72538041542671	2.22365702064354
H	-0.77451618155789	-4.22968629623809	2.75096786629434
H	0.99736088082949	-4.82891669705834	1.18373117091775
H	2.09356877818047	-2.21533783467728	5.25668724788730
H	3.88718577595132	-2.78305026538181	3.67452041749636
N	-0.42233113993445	-2.30121067750580	4.65597595146346
N	-1.63456148607481	-2.50615965501722	4.33444683904409
H	-4.11586658915714	-2.59001444370775	3.65765516098186
C	-2.44016389062685	-1.38797135398843	4.29292213001062
C	-3.72088260524451	-1.58071236784869	3.72568809319954
C	-1.96933885116319	-0.06873058969540	4.49964791506589
C	-2.65031737370673	0.98488400256345	3.94805066951704
C	-4.38212828562415	-0.51784123222273	3.15235723135523
C	-3.79980807919261	0.77132375874057	3.14307098776524
H	-0.83493191214061	1.63056709048806	-5.67471753927998
H	-5.33109785520240	-0.69084986387791	2.65482014134396
H	-1.02558455837918	0.08690978201289	5.01090316214834
H	-2.22264657708537	1.97879343938599	4.02733170956114
H	-2.59325680259366	4.75374778286980	-0.98945229821287
H	4.95585093405428	-0.25810238091467	-2.10790765102867

69

3CAB-0_all-trans_3H, Eel(r2SCAN-3c) = -1715.136310483804 a.u.

C	-3.76947985958796	3.39389983498760	-0.06412078769609
C	-3.52181058542627	3.92195715100230	1.21724548209476
C	-4.47248975678010	2.18545297795254	-0.20800842074603
C	-4.69110387468288	1.41365046429606	0.90714503496213
C	-3.73243443635290	3.12840854216127	2.32274447115005
C	-4.20231231348749	1.79898140706631	2.18384991014050
H	-4.75053126489308	1.82728028607549	-1.19229803719173
H	-5.14627705290420	0.44028112957912	0.76565961284974
H	-3.09771655514511	4.91626740036708	1.33596977452285
H	-3.48697127809159	3.53040015698860	3.29970345957441
N	-3.12374451788973	3.98468680700662	-1.14558772210377
N	-3.08772754832301	3.40001908000281	-2.30083054273120
H	-2.97569494005181	2.27208983472989	-4.57265214565275
C	-2.02027463070602	3.55615903954271	-3.11435365795414
C	-2.02841878943627	2.69289137379522	-4.24872218029166
C	-0.81428005032235	4.25514362265430	-2.81018419200673
C	0.35686982567166	3.84693397155234	-3.38665310299776
C	-0.84795873573048	2.30499961717086	-4.82606482246885
C	0.39102521575690	2.74096243482994	-4.28849350737698
H	-0.77392787480911	5.04483818454003	-2.06512384811627
H	1.28017705115603	4.31081161747110	-3.05836774791827
C	3.55834632165613	0.11642370545982	-3.61269917328238
C	3.82257497851843	1.49633241191804	-3.52286065304665
C	2.44096408222796	-0.34516675458215	-4.32934929008461
C	1.48845853695752	0.56362971504925	-4.72161699153237
C	2.85014859248877	2.39256901193711	-3.90675837699344
C	1.59952964310887	1.94194130202468	-4.39702993430889
H	2.28967013863928	-1.40707766496598	-4.48386833565814
H	0.58679498503939	0.18086254669441	-5.18545382383704
H	4.75092534667372	1.85700422025503	-3.08619309713540
H	3.04783719910785	3.45167558536099	-3.78229407918832
N	4.30215608551498	-0.74078837266212	-2.80802529418879
N	3.94257558039282	-1.97324374056215	-2.63633537666083
H	3.25482029348863	-4.38767882305112	-2.25711771416336

C	4.18543128124696	-2.60049861773478	-1.46447320561047
C	3.55156182653738	-3.87206075666230	-1.34858703715849
C	4.74359846364676	-2.01787573936431	-0.28783974488708
C	4.38287765739903	-2.51041751755460	0.93601993098679
C	3.21038190401381	-4.36036175939650	-0.11472688913829
C	3.46509550831267	-3.59727832143128	1.05445096467631
H	5.37608341328399	-1.13518199499765	-0.32191995297002
H	4.72227483181861	-1.98519276479680	1.82169246214294
H	2.67041865954980	-5.29891398297758	-0.05409882445046
C	0.58112748923568	-3.04708269863834	4.01359962750536
C	1.90257557993696	-2.66192791333509	4.30941483075315
C	0.32654465824081	-3.97277626303172	2.98717568145976
C	1.34626564323271	-4.29931515274591	2.12660932101420
C	2.90955649135980	-2.98512530880464	3.42753899109617
C	2.63139863120080	-3.70510674719371	2.23937293080561
H	-0.67753208760377	-4.34572784731177	2.82239474839382
H	1.10946518569725	-4.93672247814304	1.28263273405777
H	2.12120704912698	-2.05175882450193	5.18262113156695
H	3.91380387066851	-2.63619374025542	3.64207338399160
N	-0.45239284649320	-2.33518134972344	4.61409506282594
N	-1.67237281641755	-2.44962465397722	4.19426329503807
H	-4.07170988014507	-2.63011795614175	3.38358615485943
C	-2.51938418228074	-1.39873629831083	4.25668873066771
C	-3.75183275105988	-1.60957970110253	3.57206156362738
C	-2.19853727812339	-0.07146198005905	4.67009772633669
C	-2.88847226606653	0.97989119188833	4.13234811111403
C	-4.44060179962109	-0.54521184332804	3.05249189667284
C	-3.92404582117584	0.77122354982916	3.17230109090824
H	-0.87438435285065	1.59609980339442	-5.64635589335436
H	-5.34148856476185	-0.73413862516144	2.47906606628232
H	-1.36250124471440	0.14428393131798	5.32952236688568
H	-2.55727611202641	1.98536993505981	4.36638592462739
H	-2.49833141864043	4.76255271601651	-0.91750548988361
H	4.98597326668851	-0.29015380410519	-2.19357004153814
H	-0.16427980099481	-1.56487456336735	5.22390346073279

66

3CAB-0_all-trans_c1, Eel(r2SCAN-3c) = -1714.2246227797 a.u.

C	-3.82127746729601	3.40930743164121	-0.11672816759080
C	-3.63966634688634	3.94366534236694	1.17164430311738
C	-4.42891069569362	2.14458598664336	-0.23952258372742
C	-4.62018360683066	1.36392861420582	0.87938183712806
C	-3.84103762505295	3.15534171629833	2.28893403906237
C	-4.21654210718324	1.80359201835185	2.15931746508286
H	-4.60070742036713	1.73524540900513	-1.22906853927650
H	-4.94186072672725	0.33585318681087	0.74512393951561
H	-3.22714400286103	4.94427691600125	1.26582694265694
H	-3.61399065809356	3.56108038522789	3.27050682677998
N	-3.08006105597177	4.01369407721150	-1.13789585230741
N	-3.15046071536802	3.43912131618301	-2.26628122534475
H	-2.96422664718992	2.46195291974202	-4.64875664145829
C	-2.01371557589748	3.57044994438800	-3.07128264205492
C	-2.01468183752539	2.82012650695040	-4.26101174520019
C	-0.79197926547657	4.10051592268715	-2.61280206828948
C	0.39143540821924	3.69445644950212	-3.18951952220085
C	-0.82249447517638	2.42337354955250	-4.83686524301067
C	0.41072611315157	2.74902329339955	-4.23845581928590
H	-0.78352575953297	4.70517625367468	-1.71247970789340
H	1.32474229198691	3.99088209381978	-2.72099649762471
C	3.58561893195898	0.06098226459332	-3.65648428572896
C	3.85909937058507	1.44052270087714	-3.63315329313344
C	2.40295707043725	-0.37854643739303	-4.28254364269497
C	1.43968225492009	0.53596524195766	-4.64827537061329
C	2.88918625151726	2.35069633534245	-4.00892848731765
C	1.60919011923543	1.91713377454714	-4.40717523342626
H	2.19577539087507	-1.44197230397102	-4.33228346537412
H	0.47485798906171	0.16692226090844	-4.98198275138676
H	4.79920305820486	1.77680433790884	-3.20492517224901
H	3.09108642321442	3.41266026439716	-3.90342502536976
N	4.32607473713201	-0.72295002195420	-2.76508990950843
N	3.97755719160959	-1.94067998389487	-2.70184438696578
H	3.45398813334497	-4.42424802315098	-2.22891010922178

C	4.19092263225858	-2.55565961598415	-1.46340651954829
C	3.67764114751820	-3.85830211339069	-1.32893704511099
C	4.55407153585267	-1.85347455542883	-0.29769300462282
C	4.19499699625262	-2.34410047869230	0.93855264582845
C	3.32690598454684	-4.34514176443662	-0.08382592105230
C	3.46282526986043	-3.54483717533498	1.06768417586509
H	4.97818071641188	-0.85960383257162	-0.39054850597572
H	4.34393381213089	-1.71348815715082	1.80952143459874
H	2.85397533402201	-5.32018047900719	-0.01104317080802
C	0.53401608755343	-3.09641908661024	4.04488426640900
C	1.86680411101063	-2.77404008298737	4.35825213755130
C	0.28821389035140	-3.93910197156171	2.94336352179609
C	1.30920061274165	-4.24028751946373	2.06883414238004
C	2.88579852860273	-3.08664623686742	3.47833372421121
C	2.60900458312927	-3.71855537232549	2.24990218556188
H	-0.73348747660828	-4.21813072660551	2.70979299980833
H	1.06549912190862	-4.75742938240155	1.14600200356265
H	2.06223828123701	-2.17433559525502	5.24279922081177
H	3.89596306353058	-2.75427177526211	3.69914116903291
N	-0.44383537414534	-2.28632391932321	4.63223961782676
N	-1.62959219731935	-2.50239657246771	4.23779270879635
H	-4.12356892121162	-2.58509992623028	3.57450770604960
C	-2.47572570930202	-1.38845705575876	4.26257769620822
C	-3.74887268751157	-1.57233875692413	3.69360238728266
C	-2.02484478407326	-0.07337286170082	4.48853127460894
C	-2.71549898476685	0.99051385770246	3.95089015703005
C	-4.43800613292988	-0.49803431678404	3.16324217819947
C	-3.87518092941609	0.79357142656402	3.16934955167090
H	-0.84569615331463	1.77393001316530	-5.70711458834835
H	-5.38075340903691	-0.67389351720053	2.65338654825802
H	-1.05704019119588	0.08026653372429	4.95333082054806
H	-2.26762350441187	1.97806127273935	4.00183651648238

66

3CAB-0_all-trans_c2, Eel(r2SCAN-3c) = -1714.222435009059 a.u.

C	-3.33358615693238	3.92767919272066	-1.19593896415826
C	-3.12018589765893	4.35312094934111	0.12823825081020
C	-3.85733065506190	2.63761403762167	-1.41017177104301
C	-3.93219202266681	1.74474354278617	-0.36369249664193
C	-3.20599989699288	3.45253910565489	1.17259058301576
C	-3.49151556576087	2.09347630932706	0.93194761040501
H	-4.05178633517513	2.30930552333696	-2.42550952196161
H	-4.18670131676695	0.71215236075911	-0.58126794869467
H	-2.77454019872867	5.36885587121966	0.29882956463663
H	-2.95617466962033	3.78221646927711	2.17684370829255
N	-2.68949488694037	4.67088502145968	-2.18895766869770
N	-2.80280804991534	4.21656534728496	-3.36790948131284
H	-2.74684810308230	3.61771566466586	-5.89416926472994
C	-1.71720621671981	4.47275469554435	-4.21029602126146
C	-1.77908426089123	3.91693505651626	-5.50142240073373
C	-0.47181279773000	4.93123413694848	-3.73878913643215
C	0.67955060282742	4.62664172524896	-4.42987015722546
C	-0.61870307230189	3.63075152220082	-6.19527856897967
C	0.64652153049097	3.86203139410386	-5.61704757109459
H	-0.41597072819717	5.38752906139357	-2.75643706198733
H	1.63226564369850	4.85462859042307	-3.96366044319500
C	3.80707922405515	1.09911469436925	-5.66824187694804
C	4.08458434085951	2.43459664861421	-5.31117318057270
C	2.64878200362558	0.83260390381180	-6.41307288100660
C	1.67482458869509	1.80415237356224	-6.55742595142940
C	3.11852265223117	3.40118405063141	-5.48237039996907
C	1.83378096379189	3.07794503924072	-5.98182627894093
H	2.45911451863698	-0.19015308746255	-6.72499946148047
H	0.71934280408718	1.51719991254722	-6.98363652761548
H	5.01771330734732	2.66986581515250	-4.81074835438847
H	3.32482829135564	4.41268091857560	-5.14500734468282
N	4.39228916262363	-0.03481268291380	-5.08797965490507
N	5.16437743716890	0.22181102109352	-4.11460891942096
H	4.44562593364322	-2.46603610750807	-4.32176998621244

C	5.29541706565538	-0.82915841753350	-3.19628735234252
C	4.72582311975985	-2.11139089133046	-3.33575929867213
C	5.75356081917634	-0.43463479352019	-1.93063362849719
C	5.41178482904222	-1.16586794477803	-0.80731940431449
C	4.41254391205369	-2.84432906163292	-2.21259062809603
C	4.62053160345132	-2.32430231780638	-0.91226117211199
H	6.23188905842864	0.53547305485535	-1.83370873099327
H	5.62859973426312	-0.74394740768005	0.16834488525991
H	3.91754931860768	-3.80325834724207	-2.33480474012637
C	1.71393254552650	-2.54126722735795	2.12074725213650
C	3.03637608157649	-2.21544693686106	2.47402208461156
C	1.49059462229954	-3.18582861938305	0.88860842902019
C	2.50841349635063	-3.27675789099285	-0.03431669170395
C	4.05493740166011	-2.32663338949962	1.54664221584283
C	3.78895572874975	-2.73995003726570	0.22488394229906
H	0.47899170654202	-3.46761378446804	0.61730925805004
H	2.26678639508671	-3.63410434621362	-1.02976235615360
H	3.22158496562720	-1.77367079276248	3.44915496533576
H	5.05264788998469	-1.99771988177147	1.82121033602542
N	0.70017378154199	-1.90234804061629	2.84030035191634
N	-0.48035002314125	-2.16268409085504	2.45589877767175
H	-2.99541919967438	-2.38694275157112	1.90219770230235
C	-1.40866307298524	-1.13286940525803	2.63243806784732
C	-2.69236592561758	-1.36342064008558	2.10431515696134
C	-1.04810113190028	0.18696522019712	2.96786107204853
C	-1.84385615768439	1.23832730482758	2.56868707221930
C	-3.48713330149274	-0.30250439887842	1.71457727033253
C	-3.02384778245605	1.02417180921408	1.82283847344834
H	-0.69199085064229	3.13303818445892	-7.15752327276910
H	-4.43930229257015	-0.50434881035495	1.23264649858281
H	-0.07137022525110	0.37285929930146	3.40140576308683
H	-1.46911628596455	2.24903727531732	2.69670727934360

66

3CAB-0_cis-cis-trans, Eel(r2SCAN-3c) = -1714.246061658788 a.u.

C	-5.33713849474775	4.64877267453194	0.03990348543900
C	-4.99715105962921	5.05068744426965	1.34075583678732
C	-5.91221272461152	3.37862082508422	-0.15122151608099
C	-5.92010091635638	2.46909004684283	0.88610676020555
C	-5.00877224895170	4.13143301889644	2.37469902778059
C	-5.35752701501578	2.79242507702245	2.13545288043420
H	-6.19963501554520	3.07323484297298	-1.15141786678117
H	-6.22562785538854	1.44494937771690	0.68939812255859
H	-4.60093374487969	6.05121440810701	1.48930635029763
H	-4.65494587496383	4.42672706585369	3.35851307784099
N	-4.71885509851545	5.38449107914939	-0.98522673624987
N	-4.83711175081457	4.88285605324981	-2.13896980531623
H	-4.68925563287846	3.93223557009217	-4.51701845752946
C	-3.74619867883609	5.11526416666547	-2.99365285904959
C	-3.74927044599733	4.35398716245070	-4.17254175257694
C	-2.54533657288026	5.71941718370964	-2.57715183021226
C	-1.35822011529818	5.37295977969084	-3.18880448868642
C	-2.55364733925488	4.01219566379516	-4.77910490359964
C	-1.32972348809420	4.41959779671312	-4.22428245051643
H	-2.54328248185732	6.32944703314856	-1.68043244564904
H	-0.42195811551982	5.72583398743356	-2.76476476417289
C	2.21104496404271	2.07088373917823	-4.37513902473137
C	2.32972098516874	3.46259063145258	-4.40130544534080
C	0.95370362915222	1.47854779072890	-4.52120342367600
C	-0.17365838960068	2.27397075450107	-4.58649088205617
C	1.18872836734296	4.25169227566969	-4.41397920434830
C	-0.08795168054035	3.67183242733126	-4.47513870101611
H	0.86399894336950	0.39782625298088	-4.55632164704983
H	-1.15028696834154	1.80206246486445	-4.63405877253986
H	3.31820757582625	3.91132493905390	-4.36459864407689
H	1.28664351855047	5.33330713718259	-4.37684347889365
N	3.39369089243423	1.27311882215316	-4.34440865281879
N	3.50457243712314	0.30795708737933	-3.56569238829670
H	2.22325286611813	-1.94876059507917	-3.07572300127820

C	2.59073354040563	0.08322769965023	-2.48237839061140
C	2.03827563347598	-1.18884524196830	-2.32158076475697
C	2.41193692698133	1.04709800501112	-1.48493796371097
C	1.68349464563640	0.73522846503352	-0.34601666222901
C	1.25173312011997	-1.46662476053639	-1.21259724767881
C	1.07352638007304	-0.51679438141704	-0.19696358686743
H	2.86110602529097	2.02937396985376	-1.59739089180812
H	1.55681853520396	1.48459844688946	0.43104897073685
H	0.80939473627489	-2.45310301564614	-1.10072516551318
C	-1.18170162590451	-1.49320100602752	3.31598700517617
C	0.13113776284966	-1.03196038322560	3.42978262036878
C	-1.74978572355513	-1.65983869016127	2.04891497093689
C	-1.00801942399469	-1.36162968053875	0.91496867287373
C	0.83822020515057	-0.66889498053357	2.29228560873029
C	0.28912174533132	-0.84253674650628	1.01407621712012
H	-2.76251593119627	-2.04105306883669	1.95775289175222
H	-1.45061307262873	-1.50343360565740	-0.06759888360420
H	0.58412389636811	-0.95287120725254	4.41414433865054
H	1.85392497554844	-0.29393859203355	2.38751762456907
N	-1.84530147135571	-1.98540989591378	4.48919931090295
N	-2.98857000265063	-1.61554154779387	4.81537151698135
H	-5.46742109476491	-1.53302468212379	3.91267106736305
C	-3.61066297457561	-0.49100303460167	4.19529894282555
C	-4.94168323979919	-0.59142021629240	3.78307929799364
C	-2.95341254584549	0.74039383320584	4.12581166815804
C	-3.59058470682654	1.82445956171282	3.55419099441012
C	-5.55449965168948	0.48759619003152	3.16271237694621
C	-4.87952482710934	1.70835536260912	3.00749677858699
H	-2.55906113156212	3.34542695811780	-5.63681519669365
H	-6.57122120204162	0.38475026607902	2.79318531374550
H	-1.93779168015246	0.83473533354442	4.49576813145754
H	-3.04787029367231	2.75852666053075	3.44584803439072

66

3CAB-0_cis-trans-trans_c1, Eel(r2SCAN-3c) = -1714.235406381245 a.u.

C	-3.27205759529816	2.87683400838917	-0.21184050193629
C	-2.95914163994281	3.36638247300296	1.06547494155161
C	-3.79268389937509	1.57573005386569	-0.33436695793125
C	-3.84407661397199	0.74894027053014	0.77219389444038
C	-3.01279799160268	2.53080850123175	2.16670437789182
C	-3.37793724817608	1.18286326302399	2.02658036041255
H	-4.03662127925186	1.19486359410989	-1.32035160856112
H	-4.13806682315902	-0.28997845651749	0.64687329044350
H	-2.58475119627199	4.38227373413449	1.15307936536665
H	-2.71178684456190	2.90518847356177	3.14117834226673
N	-2.73607067815689	3.61713537422893	-1.27726156854576
N	-2.91170723365200	3.13023652222933	-2.43040700072657
H	-2.84284460220896	2.28614136919620	-4.88146239933835
C	-1.91017507633800	3.47826941781344	-3.35522133587496
C	-1.92046404671960	2.76474028070753	-4.56439786690608
C	-0.74276723977540	4.18044679864988	-3.00336107859636
C	0.43376906352380	3.92255999579312	-3.67045006982635
C	-0.73553083815728	2.52407249329691	-5.23854198663068
C	0.49220875447085	2.96359861533589	-4.70640341887098
H	-0.73761985260694	4.78451617913917	-2.10286692761655
H	1.35279690878509	4.33582902410958	-3.26794123678894
C	3.56831499217104	0.35538523990419	-3.76275013552962
C	3.86303210610419	1.72805580896310	-3.78055922233660
C	2.50310792133967	-0.10984002231451	-4.55562895572547
C	1.60332090525609	0.78943631504488	-5.08236505283594
C	2.96119661549343	2.62471006400613	-4.32752441176033
C	1.72946792971780	2.17608739652071	-4.84258776389540
H	2.29935759557551	-1.17395703840199	-4.59981298493428
H	0.69553080236176	0.40221706268613	-5.53336601264772
H	4.73229800941824	2.08352895033529	-3.23430730393505
H	3.15591681746347	3.68860493159105	-4.23112011013903
N	4.10556639639964	-0.37226958781783	-2.68514071446719
N	3.63667953270576	-1.54050060531563	-2.57087895106738
H	2.79914126206725	-3.89344399254259	-2.04488653353881

C	3.62445609563051	-2.07079826394780	-1.27118869524002
C	2.98579691773450	-3.31392159903533	-1.14533738128771
C	3.90497124698788	-1.32799278977877	-0.10995744775757
C	3.41685746858470	-1.75197936160788	1.11179057822355
C	2.49989420915559	-3.72899494713642	0.08161735601825
C	2.63483066896760	-2.91654100438141	1.21808245421773
H	4.39969059577413	-0.36718822195327	-0.20394969656815
H	3.53844385465963	-1.11428017423529	1.98359455573767
H	1.95547566204190	-4.66629656031057	0.15543501656857
C	0.07207601116047	-3.27338793366207	4.61696502333056
C	1.42897461613946	-3.00746452610878	4.81400378297222
C	-0.39703388058363	-3.56695988402940	3.33350603902629
C	0.47149425483959	-3.52123093667852	2.25811495895798
C	2.28075454050726	-2.90918335814627	3.72215758821164
C	1.81593787174621	-3.15031772945504	2.42102156036304
H	-1.44329995648602	-3.81326990811790	3.18238634546267
H	0.08849257475898	-3.70748786872781	1.25900309788935
H	1.79666804184797	-2.84517933158796	5.82323094458828
H	3.32686980757344	-2.66074240458883	3.88022173558721
N	-0.78434504989606	-3.39122464597230	5.75807529045439
N	-1.88677787427836	-2.81289183901358	5.81325576409295
H	-4.18239673248199	-2.69914892332024	4.51627045455829
C	-2.25913074492316	-1.80854866439234	4.86341885778317
C	-3.52604706953369	-1.86686247212506	4.27839307078526
C	-1.43928178092087	-0.70065946086677	4.63120948665650
C	-1.85879177371125	0.29469448513354	3.76738825734726
C	-3.91471096222634	-0.89141122697238	3.37022960393326
C	-3.08298951547517	0.20160145348821	3.08601075464483
H	-0.75050037306660	1.87655154611058	-6.11006894272175
H	-4.88551094787079	-0.96606263477843	2.88749386032042
H	-0.47029793022299	-0.63281065216497	5.11562184374661
H	-1.19630476005982	1.12972332987178	3.55897142068704

66

3CAB-0_cis-trans-trans_c2, Eel(r2SCAN-3c) = -1714.234972900642 a.u.

C	-3.04529379078749	3.14887749126274	-0.72578376188460
C	-2.76808857037397	3.63250211788994	0.56209378423713
C	-3.59947328975531	1.86350220222925	-0.86593581380622
C	-3.71875016071088	1.04224403503159	0.23882510067845
C	-2.89272233782020	2.80308310347643	1.66238771741826
C	-3.29180593760230	1.46572110771257	1.51096961276606
H	-3.81533828686179	1.48700659156802	-1.86007625098334
H	-4.03677850968464	0.01197405004322	0.10240976136473
H	-2.36718584951942	4.63698619857737	0.66307058289830
H	-2.62180604739016	3.17311658634310	2.64724789718832
N	-2.44887767570336	3.86632609685104	-1.77417407053505
N	-2.61279121158607	3.38626219632027	-2.93215249239857
H	-2.53133396325427	2.56339252867997	-5.39293072904991
C	-1.57425274270949	3.68766604167749	-3.83034863075584
C	-1.59342729895216	2.99045012625689	-5.04899122493271
C	-0.37991726804114	4.32393760210214	-3.44633357816703
C	0.79547965319197	4.01476911457186	-4.09268502261439
C	-0.40844245882754	2.69936521000706	-5.70213288510068
C	0.83041628686573	3.06563364858144	-5.13951180066806
H	-0.36358701748412	4.92081599022801	-2.54113525844763
H	1.72072293527621	4.39001749171810	-3.66965206857304
C	3.69089162938876	0.19708599816910	-4.27461545316810
C	4.03781926681558	1.54745471488089	-4.07933451108913
C	2.71406885376823	-0.10992982050268	-5.23036637657899
C	1.88460733737514	0.88810513664122	-5.71288932424075
C	3.22993836321725	2.53565134197381	-4.59764908980476
C	2.02757616039966	2.21669971593705	-5.27549598812195
H	2.49371836430419	-1.15409797127817	-5.43128217254480
H	1.01552797404814	0.59310356709582	-6.29038716062296
H	4.86563838330955	1.80036266499687	-3.42552060687562
H	3.46589766927727	3.57038836764429	-4.36968482546213
N	3.94937426317045	-0.85263345216542	-3.37538769663780
N	4.36616934677732	-0.45719764525625	-2.24911866094864
H	3.17514981162537	-2.94451475863995	-2.27453178148741

C	4.10339671385034	-1.33570217270664	-1.18050700177467
C	3.34692848373127	-2.51626003165937	-1.29301421156420
C	4.36207797889514	-0.81465641774272	0.09467639435496
C	3.74829443016129	-1.35804139817980	1.21208343862852
C	2.73768698139389	-3.04832806472865	-0.17512792353449
C	2.85607311654444	-2.43251445346743	1.08655622773205
H	4.95402281241046	0.09261853508571	0.17225180385919
H	3.86648695972050	-0.86797719245005	2.17434433417603
H	2.11248750005027	-3.93118658006303	-0.27855905119328
C	-0.00300533850086	-3.03431639659180	4.20414398800545
C	1.34248386545957	-2.85802876180661	4.53691529742253
C	-0.36867016723391	-3.17665697054723	2.86210526690657
C	0.58944974401058	-3.05607356733480	1.87262337106043
C	2.28919495568982	-2.69184105558593	3.53586047716042
C	1.92903399349916	-2.76579304423167	2.18181762682238
H	-1.40557829539349	-3.35799786203741	2.59796258895614
H	0.28252035574005	-3.11605028638445	0.83278358618529
H	1.62712310948417	-2.81992998777358	5.58451492766411
H	3.32712972239359	-2.51876212699696	3.80688577788314
N	-0.94626962683746	-3.21088783656483	5.26496336723425
N	-2.05036768523749	-2.63170289128833	5.27366071848006
H	-4.27093093551772	-2.44683693772687	3.88062886338978
C	-2.36328093502459	-1.58210600191774	4.35323097059569
C	-3.59788745004427	-1.61343025309611	3.70053579195290
C	-1.52757786423991	-0.47213242852044	4.20002162399421
C	-1.88969717423686	0.54532419789386	3.33589021463110
C	-3.92853373760986	-0.61362783324488	2.79659690897573
C	-3.07128743607327	0.47546079113576	2.57957576278641
H	-0.44342041450704	2.06443998115957	-6.58169688032638
H	-4.87214156194752	-0.66873958037761	2.26041531282940
H	-0.58605982945145	-0.42414006193744	4.73826194147026
H	-1.20880615292360	1.37771929906014	3.18415126418604

126

3CAB-1_all-cis, Eel(r2SCAN-3c) = -3100.290073742309 a.u.

C	-6.57750464779806	3.56723376235245	0.06563760508835
C	-6.44887881822485	5.03551051828784	0.07475448037992
C	-6.30236308659911	2.81482291998691	1.21389957558558
C	-6.93877898133718	2.87930529791894	-1.09981194913347
C	-7.00567841500924	1.49501532824175	-1.12004754281233
C	-6.35170722900155	1.42966873583500	1.18770906476253
C	-6.69648903172202	0.73732353441186	0.01830149770329
H	-7.20121257808476	3.44215364001165	-1.99163740123971
H	-5.99017015672094	3.32334329519427	2.12222794645941
H	-6.06878148365938	0.87380225441562	2.07685135813650
C	-6.94023603922546	5.80548240343348	1.13820568395190
C	-5.81658719504781	5.69600074137616	-0.98782952971397
C	-5.62764405410334	7.06720576234711	-0.96707096995969
C	-6.80742299846143	7.18614007156935	1.13997674138647
C	-6.10696796811572	7.81762332081071	0.11107894718877
H	-5.42905934040502	5.11563217534276	-1.82069445049366
H	-5.11048791132819	7.56199164627151	-1.78312751125911
H	-7.46925670144324	5.31555938799011	1.95125533056051
H	-7.22648305792060	7.78669512627094	1.94238244235804
N	-6.04793913915281	9.24690993390649	0.10596203592489
N	-4.98509542674372	9.86108880773888	-0.10373263997643
C	-3.71732498848694	9.19843123342381	-0.10975174752779
C	-3.30621074765588	8.40755299421892	0.96765849081755
C	-2.82065236612647	9.49086945966797	-1.13859810723383
C	-1.55804769140598	8.91664210762556	-1.13756548439231
C	-2.02376434899979	7.88673912588764	0.98775028883184
C	-1.13598418099732	8.10573823176301	-0.07480891587703
H	-3.13194000839077	10.15445674572160	-1.94040048552868
H	-0.86963695976017	9.13107781446840	-1.95061718587369
H	-3.99286723391273	8.20579908761321	1.78375036902134
H	-1.71414894557758	7.26054649601190	1.82003069427490
C	0.20037979641277	7.48403489496453	-0.06621610878210
C	0.71467503231025	6.87020987726825	-1.21470493576108
C	0.97690990023300	7.45304506519000	1.09913745443914

C	2.20941528367592	6.81927370596618	1.11920071957225
C	1.93914789551157	6.22076854223038	-1.18867513025647
C	2.71108482910632	6.17291269300956	-0.01924811819526
H	0.62050169463931	7.96135483911569	1.99110960048459
H	2.80492563787498	6.84730881893518	2.02716589148906
H	0.11820861242912	6.85415828283503	-2.12304863820226
H	2.27924164070129	5.69817442897636	-2.07797273953725
C	6.37808597524467	3.91548078871674	0.06501721789851
C	7.58465608439153	3.06894067911424	0.07416080780188
C	5.58888178146390	4.05387126061696	1.21320142525410
C	5.96362316941325	4.57275076688843	-1.10043087641506
C	4.79879418499044	5.32369293060221	-1.12079524157895
C	4.41451778438218	4.79001918678098	1.18686756884200
C	3.98793492814742	5.43516477034223	0.01744163737623
H	6.58237385594965	4.51822415032136	-1.99216697850741
H	4.52582735202333	5.85372863833485	-2.02874715912055
H	5.87267312977578	3.52898380587832	2.12153610375000
H	3.79147227760212	4.82327033319040	2.07586224635840
C	8.49729372779038	3.10888111065693	1.13748661135568
C	7.83956111354868	2.19066070918415	-0.98827308579234
C	8.93188449273946	1.34053471859150	-0.96756711317013
C	9.62596969028897	2.30267469926543	1.13915046957472
C	9.82175151892839	1.38000228530991	0.11035854527393
H	7.14297518614910	2.14575287236454	-1.82098958950160
H	9.10106908289662	0.64501410367270	-1.78356310945991
H	8.33819484343668	3.81223521373388	1.95045304968200
H	10.35582226324004	2.36494062100522	1.94137789722436
N	11.02969310317113	0.61374581303984	0.10486092346909
N	11.02968704177287	-0.61375617807378	-0.10486683735599
C	9.82173770494438	-1.37999832888609	-0.11036610519065
C	8.93186904630042	-1.34052128119764	0.96755792804287
C	9.62594873039172	-2.30267069133959	-1.13915656455832
C	8.49726605561674	-3.10886773544763	-1.13749307865534
C	7.83953950521867	-2.19063901197356	0.98826499785231
C	7.58462820481966	-3.06891932816709	-0.07416734919706
H	10.35580172201098	-2.36494434849594	-1.94138279200501

H	8.33816365843549	-3.81222308291597	-1.95045780668851
H	9.10105890529771	-0.64500227687179	1.78355395005013
H	7.14295357520003	-2.14572395242573	1.82098108002834
C	6.37805350161544	-3.91545289999142	-0.06501915523371
C	5.58885196913156	-4.05385137136869	-1.21320434407207
C	5.96358484125829	-4.57271078454649	1.10043406102724
C	4.79875389607464	-5.32364951942485	1.12080175398163
C	4.41448549116482	-4.78999494577108	-1.18686670699564
C	3.98789795697310	-5.43512924169640	-0.01743633974790
H	6.58233433622689	-4.51818048184260	1.99217071441227
H	4.52578275130458	-5.85367689529283	2.02875735712175
H	5.87264734505543	-3.52897347490538	-2.12154319946262
H	3.79144279466639	-4.82325412090100	-2.07586329130454
C	-6.57749519845680	-3.56727018019934	-0.06565378589443
C	-6.44886904444774	-5.03554706019926	-0.07477075479232
C	-6.30233738220579	-2.81485835483982	-1.21391080325021
C	-6.93878880864081	-2.87934385193807	1.09979079973686
C	-7.00569186799083	-1.49505393118604	1.12002620729661
C	-6.35168476881270	-1.42970437729201	-1.18771998027518
C	-6.69648635945243	-0.73736099888281	-0.01831761962362
H	-7.20123417527152	-3.44219384334192	1.99161183573577
H	-5.99013147480261	-3.32337783135671	-2.12223526959026
H	-6.06874722478344	-0.87383664977587	-2.07685776960266
C	-6.94022983252561	-5.80552039037431	-1.13821908914836
C	-5.81657577305941	-5.69603508006847	0.98781279840211
C	-5.62763783217092	-7.06724072592541	0.96705888525759
C	-6.80742119426538	-7.18617875785923	-1.13998619419088
C	-6.10696748126806	-7.81766012767112	-0.11108646987532
H	-5.42904615286753	-5.11566453430896	1.82067577734568
H	-5.11048261257093	-7.56202642308026	1.78311631465065
H	-7.46924907376780	-5.31559760509309	-1.95126980689264
H	-7.22648605515045	-7.78673513336935	-1.94238819190913
N	-6.04794760082730	-9.24694798795696	-0.10595125788517
N	-4.98509713330466	-9.86112328980027	0.10373536973261
C	-3.71733847342620	-9.19844347670774	0.10977082095263
C	-3.30623362909409	-8.40755771614466	-0.96763683053271

C	-2.82066443159648	-9.49087015582871	1.13861886514184
C	-1.55806699677878	-8.91662633498671	1.13758827893117
C	-2.02379402115418	-7.88672815578402	-0.98772727290667
C	-1.13601179047391	-8.10571847019852	0.07483132666071
H	-3.13194384746226	-10.15446332446678	1.94041927754122
H	-0.86965428506769	-9.13105365066565	1.95064052460687
H	-3.99289107281719	-8.20581421268520	-1.78373042144073
H	-1.71418479252606	-7.26053236984401	-1.82000777155757
C	0.20034760233807	-7.48400498243971	0.06623263028463
C	0.71463671296440	-6.87016321059527	1.21471489764453
C	0.97688071274658	-7.45302714658204	-1.09911934501240
C	2.20938417018429	-6.81925220708246	-1.11918687915323
C	1.93910717043710	-6.22071809246543	1.18868013007224
C	2.71104747561919	-6.17287549291435	0.01925534765328
H	0.62047741613481	-7.96135096236835	-1.99108543919757
H	2.80489850115715	-6.84729820399806	-2.02714923011135
H	0.11816852005868	-6.85410403044243	2.12305723447078
H	2.27919834575442	-5.69811340151406	2.07797277700324
H	-7.32774922429112	-0.99333943356428	2.02798336120608
H	-7.32772018859410	0.99329955111546	-2.02800952650949

127

3CAB-1_all-cis_1H, Eel(r2SCAN-3c) = -3100.682120056549 a.u.

C	-6.83031342971307	3.44930143728642	0.04162372004905
C	-6.63167050040515	4.90342200514843	0.03843536634558
C	-6.76990454248039	2.71526065819941	1.23414543193144
C	-7.02384887086361	2.74701702370412	-1.15714955012761
C	-7.12659608680349	1.36812448202954	-1.16421807380029
C	-6.83519515893122	1.33250859977180	1.21960860438403
C	-6.99985515525668	0.62132391482231	0.01931022152642
H	-7.13296602917248	3.29483939142192	-2.08928866050233
H	-6.59657650585283	3.23118471344241	2.17481157243342
H	-6.70221344187858	0.79316909839445	2.15186433802875
C	-7.20620425384680	5.73548841235023	1.01129036341097
C	-5.82046068015228	5.49348876173987	-0.94808524813192
C	-5.54838243856854	6.84549026224278	-0.94060099075703

C	-6.96970144006023	7.10204977426084	1.01134941738738
C	-6.11970575538257	7.65153963590033	0.04959798931468
H	-5.37297510382030	4.86639538919599	-1.71327671009398
H	-4.90504324071564	7.27943692324750	-1.69886993719202
H	-7.86467068232380	5.30774231768447	1.76133934229293
H	-7.42418383437323	7.73800244289202	1.76672887879466
N	-5.88276254558831	9.05065033251455	0.04493765681021
N	-4.81815977411974	9.70730431532051	-0.30520133642444
C	-3.58747268415846	9.11734101971983	-0.21639854597386
C	-3.23956351744746	8.09020882320625	0.69792073071229
C	-2.57397970780797	9.68360718351690	-1.02448779168728
C	-1.29292236138725	9.17798237735064	-0.99447244242845
C	-1.93748637598497	7.66607239785430	0.78328582846991
C	-0.93914555342820	8.15952558105274	-0.08714259152494
H	-2.83828269230989	10.50621029508265	-1.68180737128167
H	-0.53138957722435	9.60972367855725	-1.63684220399977
H	-3.98278472454656	7.66780644914232	1.36450222813321
H	-1.67868823412144	6.89398902905663	1.50167409398571
C	0.40675697114928	7.60218476789330	-0.03676527628693
C	1.14509489426672	7.38128633168261	-1.21179399481395
C	0.98853640728388	7.21938788114582	1.18495490507691
C	2.23463667324650	6.62802826881969	1.22536442765157
C	2.36989073769451	6.74325574485086	-1.17095756419780
C	2.94720549335919	6.33589158746364	0.04665844782091
H	0.47386330214376	7.43623868721721	2.11667930483794
H	2.67054571325288	6.40179044856444	2.19230139162483
H	0.71410939673628	7.64292906284804	-2.17410241631142
H	2.86469120083260	6.51375672419975	-2.10835389832833
C	6.58215409999515	4.03109096119537	0.10607498804662
C	7.76201435845632	3.15479521879445	0.09094110346597
C	5.71902425372858	4.05046698027741	1.21034464716498
C	6.25714918812252	4.83563389647002	-0.99592024195182
C	5.11032977420057	5.60918797155736	-1.00066034446172
C	4.55741355214548	4.80081529720942	1.19270736375375
C	4.21544056205392	5.59442606913829	0.08363536143201
H	6.93466166251938	4.87641842364796	-1.84416470659081

H	4.92198313527002	6.25177598457819	-1.85451502708424
H	5.93438277563584	3.41644415362869	2.06582304737306
H	3.88142484698631	4.72476123808513	2.03773433418501
C	8.60694324576015	3.05087832056327	1.20635440666662
C	8.05169073570687	2.38308842030252	-1.04408862794087
C	9.10588383446513	1.48816840927284	-1.04559522551268
C	9.70856447863612	2.21087169170210	1.18797501330050
C	9.92701922151740	1.38472145185841	0.08316937373020
H	7.40629671169196	2.45058242803165	-1.91551513991557
H	9.30288920992951	0.86747679110141	-1.91416313220651
H	8.41950001748414	3.67093562314321	2.07883506274925
H	10.39103586088963	2.16555246187752	2.03162955037282
N	11.09564787783757	0.56943015315585	0.04202660687750
N	11.05678003287605	-0.64000256514496	-0.22739911729463
C	9.83400620700450	-1.37497882435772	-0.23108993071698
C	8.95176190893277	-1.31784828018190	0.85359541511409
C	9.61173140772708	-2.28646137897669	-1.26565932722344
C	8.46379119516296	-3.06390312340491	-1.26129622045630
C	7.84190327402558	-2.14343873237119	0.87693434331377
C	7.56206566269416	-3.01157255490669	-0.18805617200044
H	10.33729042881294	-2.36431120357198	-2.07010380426565
H	8.28388338259897	-3.76007773037655	-2.07582145822781
H	9.14890899776492	-0.64211049149270	1.67935204059215
H	7.15677671295158	-2.09498415090559	1.71867879476741
C	6.34737577432829	-3.84568262337914	-0.16105976398873
C	5.53513136102339	-3.97475322350972	-1.29397632165109
C	5.95358096809855	-4.50383077933109	1.01115917727743
C	4.78543911827414	-5.24800343541437	1.05278537039332
C	4.35559316319072	-4.70195797353989	-1.24472532384008
C	3.94992632645041	-5.34847575662602	-0.06857633339254
H	6.59240132469116	-4.45983437254104	1.88912586848032
H	4.53093982961405	-5.78293610524101	1.96303107431807
H	5.80596491739894	-3.45313363252859	-2.20815320159761
H	3.71596156964135	-4.73101737501377	-2.12194280809654
C	-6.80065467016708	-3.67755985015209	-0.01568326155889
C	-6.62296125431054	-5.13795604788938	-0.00333908779440

C	-6.48583794376488	-2.92915153886307	-1.15757694834282
C	-7.24255297578929	-2.98761763789236	1.12201372345926
C	-7.34434560239833	-1.60678775930109	1.12393584771461
C	-6.56493884177554	-1.54727467985783	-1.14712157649370
C	-6.98659446921069	-0.85055010295076	-0.00348297258372
H	-7.53998807850886	-3.54825269245087	2.00383546331811
H	-6.11487026215468	-3.43798477323344	-2.04292811778659
H	-6.24115087610032	-0.99964525436754	-2.02660987953037
C	-7.03299103868699	-5.93097649076871	-1.08552463410146
C	-6.01510186015185	-5.76539364973512	1.09409103954300
C	-5.76401263591991	-7.12543278508994	1.08812546298710
C	-6.84691139849302	-7.30402674758844	-1.07213556901217
C	-6.16105056675781	-7.89718072902313	-0.00988201479396
H	-5.69142228485322	-5.16595256506976	1.94046840839533
H	-5.26342668143165	-7.59780382361682	1.92757185087674
H	-7.54263446997971	-5.46693732820539	-1.92556331304106
H	-7.20573478047263	-7.92518342710805	-1.88759494293665
N	-6.03176910450862	-9.31652657889432	0.01925743528576
N	-4.95593310612616	-9.88568913783174	0.25427571413570
C	-3.70764813614510	-9.19604158150157	0.23167261978922
C	-3.34239352070308	-8.38799626689512	-0.85098223498339
C	-2.78117019743299	-9.47639552501023	1.23850763451736
C	-1.53428207933936	-8.87140705351089	1.21235011234863
C	-2.07331336769047	-7.83892573853906	-0.89718006536591
C	-1.15588137953120	-8.04605497992201	0.14307475586596
H	-3.05715294181014	-10.15594273055226	2.03954810908302
H	-0.82127421242872	-9.07701506495031	2.00594565700356
H	-4.04801575371007	-8.20571510741385	-1.65505122748097
H	-1.79440640176497	-7.20691922536752	-1.73570810483503
C	0.17124931627367	-7.40700274262715	0.10041227878800
C	0.71140496811266	-6.79603696144736	1.23850515644314
C	0.91460873627322	-7.36416792242611	-1.08614322332574
C	2.14338392659148	-6.72487080893610	-1.13513075409654
C	1.93073821808769	-6.13908502200650	1.18279670720272
C	2.67187171134280	-6.08337143380598	-0.00613065892451
H	0.53931937459447	-7.87189141765149	-1.97058022094938

H	2.71572726728077	-6.74847692267853	-2.05787432474617
H	0.14171213679723	-6.79168183231616	2.16397209352400
H	2.29300392174595	-5.62240949235935	2.06667192286050
H	-7.73185367576308	-1.11116665747407	2.00881883230485
H	-7.32448562714320	0.86164522506160	-2.10335417765743
H	-6.70020423961101	9.64631399864951	0.13997011277668

128

3CAB-1_all-cis_2H, Eel(r2SCAN-3c) = -3101.032068678213 a.u.

C	-6.80593029037509	3.44124718640980	0.14445410217501
C	-6.60732071983572	4.89291744898779	0.16044765074223
C	-6.76316848512399	2.69131631109623	1.32918237459588
C	-6.98781910654410	2.75422258178925	-1.06630654966094
C	-7.09562809918231	1.37713681336554	-1.09170408967350
C	-6.82892691395656	1.30965015572080	1.29566932040510
C	-6.98095301304427	0.61336648397476	0.08369524298472
H	-7.09028835537418	3.31305629842149	-1.99247180819079
H	-6.60338061145004	3.19369910565467	2.27938571764262
H	-6.71009935537316	0.76019388187178	2.22365009829604
C	-7.17923152000890	5.71258879502878	1.14716764170332
C	-5.80380313072210	5.49705393995837	-0.82648808476224
C	-5.53160164155175	6.84760441403169	-0.80356177556859
C	-6.95299930000257	7.07962839281412	1.15637775606049
C	-6.10448750069124	7.63959302180051	0.19790820870748
H	-5.36355100637715	4.88099822589295	-1.60452439166725
H	-4.89581510552170	7.29142394396811	-1.56239055987673
H	-7.83419442712550	5.27580640454560	1.89484144492630
H	-7.41355441664558	7.70701682283313	1.91530964898819
N	-5.88514884188951	9.04068576261984	0.20324846075341
N	-4.84476727812492	9.72355925169306	-0.12869488396472
C	-3.59407975466863	9.15563847386039	-0.10679301097783
C	-3.19590903362562	8.11979932289889	0.77242073270839
C	-2.62539933953178	9.77518806713014	-0.92611465156835
C	-1.32614260664155	9.31056824632640	-0.93996093445856
C	-1.87778328697053	7.73740767060976	0.81593176516258
C	-0.92037727381468	8.28731480136045	-0.06460080283869

H	-2.93154752723984	10.60614972243877	-1.55406928587803
H	-0.59503327278999	9.78113473081576	-1.59041250065252
H	-3.90839301056423	7.65977875827384	1.44780350674756
H	-1.57338137173248	6.96084634044516	1.51123324138753
C	0.45038936887265	7.77489419766436	-0.04987344221462
C	1.14960335969410	7.54495318117881	-1.24434506493674
C	1.08048884279502	7.44534704550627	1.16102471125479
C	2.34274278103485	6.88286221631194	1.17307357070003
C	2.39481649154769	6.93996483219443	-1.22969737005827
C	3.01470201943717	6.57843705232504	-0.02258766060807
H	0.59016700494416	7.67430305457151	2.10299897802885
H	2.82114381486339	6.69022763003948	2.12786492491961
H	0.67926484707382	7.77426797105818	-2.19647418765065
H	2.86950153198959	6.69945239071347	-2.17555283455214
C	6.66732298316495	4.30925816280416	-0.03350639126289
C	7.84584299393168	3.42291596153812	-0.07241954431658
C	5.77882846479330	4.24112824463736	1.04562481205272
C	6.37121583911778	5.18491594145926	-1.08497240488826
C	5.21655179724657	5.95173323155121	-1.06464457494876
C	4.61137395137181	4.98535555336432	1.04994997148375
C	4.29726237887109	5.85066410782709	-0.00901483084058
H	7.06825261607509	5.28841811939539	-1.91222186550756
H	5.03881471240882	6.65230282745666	-1.87456978495995
H	5.97685495613401	3.55214432776797	1.86231313102646
H	3.91096784808742	4.85201805822768	1.86797072851122
C	8.66681142329606	3.26025235500343	1.05161139611903
C	8.13059350404625	2.68132401223320	-1.22893563551293
C	9.16018153792217	1.75517982564295	-1.24495048415629
C	9.71846581467399	2.35280553733499	1.04170645860698
C	9.93177522029142	1.58218140140636	-0.09729070916391
H	7.51006111651809	2.80701959514432	-2.11139967977596
H	9.35511177189713	1.15589891639760	-2.12953426718974
H	8.48399479384404	3.85762487346542	1.93997797358301
H	10.34310042819343	2.21905012317912	1.92035221525011
N	10.95534603566573	0.57438486670723	-0.09892586119075
N	10.84620186502752	-0.69729555338005	-0.27476917303183

C	9.67091814782444	-1.37401818043316	-0.20388923006001
C	8.42347659187511	-0.93076993804818	0.32405123283293
C	9.77918331161759	-2.72795554278106	-0.62637478899487
C	8.70087772834420	-3.57570202329065	-0.57810498800165
C	7.37633325469156	-1.80799768606781	0.41953777123347
C	7.46491064986239	-3.14453459207628	-0.04626744780442
H	10.74466398082322	-3.07046717091667	-0.98539949486261
H	8.82106047848414	-4.60548369294489	-0.89794748795001
H	8.29021066234780	0.07999991043958	0.68432109870192
H	6.43545214330644	-1.44893755649890	0.82293294588390
C	6.30782011784047	-4.02061840649525	0.02712965050751
C	6.10197745548786	-5.04453356192338	-0.91872553775541
C	5.33534353350078	-3.85002906150747	1.03292136820243
C	4.20904082000455	-4.64346406544466	1.07544494064062
C	4.95679835750389	-5.81526962905743	-0.89310254300886
C	3.97470293849966	-5.62708875710302	0.09719543960095
H	5.48107322437601	-3.10409442775177	1.80784996844854
H	3.47061030248490	-4.47459295764840	1.85290840951408
H	6.82642961751554	-5.20349911908077	-1.71165758554282
H	4.82180720911247	-6.58953582519810	-1.64211064782217
C	-6.80341303138659	-3.68530197509808	-0.02981226717762
C	-6.64417177766683	-5.14680748501668	-0.05851272627608
C	-6.51113161510322	-2.91728312658856	-1.16553432121554
C	-7.21002397879578	-3.01403200856903	1.13254589972815
C	-7.30412759134247	-1.63321295718895	1.16149192478015
C	-6.57942108238208	-1.53632990880253	-1.12742950209744
C	-6.97054373434879	-0.85729834726586	0.03864983365851
H	-7.49196613767501	-3.58789724707460	2.01084634679434
H	-6.16942291727351	-3.41103429729456	-2.07085106933798
H	-6.27589031886337	-0.97692368056817	-2.00630938552083
C	-7.08344991624270	-5.89975794707249	-1.15929634035842
C	-6.03021697988947	-5.81662945371088	1.01153468333219
C	-5.79319387022140	-7.17796182237788	0.95737003063591
C	-6.91681285999899	-7.27350377577017	-1.19218565240073
C	-6.21435946891310	-7.90630177797228	-0.16235997442037
H	-5.69351841473878	-5.24960827942369	1.87487473408655

H	-5.29180186363112	-7.68471569039063	1.77618068303701
H	-7.60366986314686	-5.40366573552350	-1.97384349464363
H	-7.29923134084836	-7.86596777506542	-2.01795340179192
N	-6.08378227272915	-9.31958489764158	-0.21130640526725
N	-5.02509297004493	-9.91215494449970	0.02432254006084
C	-3.75706048820621	-9.27431683521415	0.05948250346613
C	-3.35977294635555	-8.37377377906999	-0.93877234418001
C	-2.83265835161233	-9.71281858662177	1.01348838769819
C	-1.55759021548636	-9.17541085750763	1.03284670946331
C	-2.06882873016116	-7.88383703529121	-0.94054891065571
C	-1.14892654666948	-8.25072521196204	0.05689544045170
H	-3.13364884270366	-10.46614094134293	1.73520343605492
H	-0.84983392997530	-9.51532162216864	1.78317716454524
H	-4.06143241835139	-8.07007926253524	-1.70855915868256
H	-1.77675403411678	-7.16957831106099	-1.70450775062673
C	0.19887547490576	-7.66719741391311	0.06995200517063
C	0.83840332673434	-7.35263070099778	1.27987603063890
C	0.87032827325806	-7.36784957467224	-1.12663013573826
C	2.10773806376421	-6.75111102973579	-1.11541084359178
C	2.06556026456976	-6.71546496048815	1.29214286477851
C	2.72127500336761	-6.38549861370013	0.09409888955797
H	0.41928493096601	-7.63582025901719	-2.07732271178626
H	2.58579471170011	-6.50666738135024	-2.05938274171069
H	0.34526087507258	-7.57469644395508	2.22162448733994
H	2.53260163325388	-6.48440086033595	2.24501977797477
H	-7.66966397827007	-1.15429847518594	2.06440460324277
H	-7.29043216976081	0.88628060854771	-2.03935731282568
H	-6.70817182265760	9.62219833385640	0.34822551149539
H	11.92246987814282	0.88691219310050	-0.08334280046823

129

3CAB-1_all-cis_3H, Eel(r2SCAN-3c) = -3101.323322739775 a.u.

C	-6.95678605148271	3.57562458330112	0.13647493931129
C	-6.75184126128555	5.03490937859763	0.10536875176979
C	-6.55850335006265	2.82556808738677	1.24888803641685
C	-7.48949370910138	2.89735655997548	-0.96734047135666

C	-7.59579782852924	1.51702876531401	-0.96683804669515
C	-6.62643904223669	1.44273071580441	1.22949918073956
C	-7.13321641126301	0.75048516921859	0.11655848183516
H	-7.85356724941834	3.46109281566716	-1.82193577911281
H	-6.13228850881179	3.32787106250319	2.11304401174377
H	-6.23567260536182	0.89856488025421	2.08210911143178
C	-7.14838689650663	5.85872510223993	1.16764260699092
C	-6.11480594296403	5.61809530761205	-1.00233628329642
C	-5.82862784707945	6.96958890051441	-1.02929458053802
C	-6.90947717496752	7.22704595086319	1.13528360492400
C	-6.22223169775212	7.76350092748676	0.04915281840519
H	-5.81915541237988	4.99228105124349	-1.83920611306833
H	-5.32007463510209	7.41088862223292	-1.88106503664173
H	-7.67254878134593	5.42790282935591	2.01560993087431
H	-7.23046727085296	7.86273472054974	1.95600488338514
N	-5.95459387451161	9.17054016387309	-0.01008766463517
N	-4.86976102999140	9.77548464617824	-0.31567115812105
C	-3.64824953640539	9.16477718644201	-0.24919770334971
C	-3.31051596492582	8.07978144153402	0.60117116555859
C	-2.62888218113800	9.80196190226402	-0.99748125274130
C	-1.34148404721156	9.31380021961636	-0.97575863336911
C	-2.00168795128154	7.68313303080300	0.69756452923125
C	-0.99325230317957	8.25351662910824	-0.11574491399328
H	-2.89204696161182	10.66836242920955	-1.59651462122635
H	-0.57297944711476	9.79797624013980	-1.57029979885809
H	-4.05983879578195	7.60040050684012	1.21965823531031
H	-1.73979005955072	6.87750133848919	1.37658408748679
C	0.36667397737148	7.72982331076693	-0.04824525356892
C	1.13240934213174	7.55463359631978	-1.21258493986346
C	0.92992680712469	7.33984767487665	1.17948066310611
C	2.19198448147114	6.78279623493312	1.23469310453862
C	2.36967642008647	6.94031481090169	-1.15763312883944
C	2.93023176549070	6.52548841364834	0.06443792945699
H	0.39128973822239	7.52680894657786	2.10407297601996
H	2.61301091081730	6.55539585323457	2.20768744362562
H	0.71893256992426	7.83218293869168	-2.17798047783028

H	2.88745131411311	6.74524597356736	-2.09005568602080
C	6.57097180151739	4.23262985674937	0.13318406667128
C	7.73289998883904	3.32625143500942	0.10423914223583
C	5.72332371765917	4.26601211822512	1.24634398187427
C	6.24875572977233	5.02964029392691	-0.97280804587486
C	5.10662757714155	5.81205677547731	-0.97342843050528
C	4.55995144016028	5.01655041316732	1.22603962925454
C	4.21293476150699	5.79846716132217	0.11120350251080
H	6.91810256156602	5.06037512567988	-1.82820349171592
H	4.91984271993408	6.45003924670871	-1.83039576289633
H	5.94601740518614	3.64824262803132	2.11207758613850
H	3.89416601891004	4.95292091409858	2.07951904483669
C	8.64473395113379	3.26162884979156	1.16665970477109
C	7.92029709861174	2.48015762450909	-1.00118274423138
C	8.94870185104155	1.55770369995156	-1.02567338202636
C	9.71125604921521	2.37167475858574	1.13671426462711
C	9.83324248247941	1.50557455740479	0.05283495620277
H	7.23044114621847	2.53389220718282	-1.83821358369687
H	9.07736195201437	0.89454333206725	-1.87569909041983
H	8.53302261500281	3.93323471798060	2.01274776917022
H	10.42238400112489	2.33494499015696	1.95749019461296
N	10.91942396721490	0.57193830910361	-0.00412194439819
N	10.90320562661712	-0.67033063919016	-0.30854258760165
C	9.76425922058209	-1.42396993891350	-0.24282184103497
C	8.65441570262442	-1.17377042582851	0.60564753035662
C	9.80835817103918	-2.62604825150450	-0.98981021987367
C	8.74227727147865	-3.49740425223623	-0.96896344286361
C	7.65691040649855	-2.10946252389515	0.70152648178791
C	7.64850383309303	-3.26863657301871	-0.11082942525209
H	10.69127183693828	-2.83137493906345	-1.58734701885739
H	8.77865748730762	-4.40551478634601	-1.56269912327638
H	8.61257090789760	-0.28450664644641	1.22316947566119
H	6.82716947938846	-1.93339055165771	1.37918991388055
C	6.51523665586958	-4.18492258959855	-0.04439020784232
C	5.98196706177831	-4.76059299087682	-1.20925123818955
C	5.89465226964546	-4.47779493375628	1.18268161931779

C	4.78124558527503	-5.29240589304454	1.23670874420115
C	4.83131542185547	-5.52504395916007	-1.15552205961749
C	4.19048414343030	-5.80307707953553	0.06585084218077
H	6.32490323512312	-4.10477223899546	2.10771955722421
H	4.37292625954518	-5.54337464559295	2.20931704300510
H	6.43009957602284	-4.54131485200435	-2.17417998313727
H	4.40461162222755	-5.87596125047844	-2.08843155624754
C	-6.87986929743199	-3.54608185573865	-0.04328222321321
C	-6.65252040424900	-4.98547387040218	-0.11167174330628
C	-7.11669794330701	-2.79567617566814	-1.20668257882371
C	-6.81900558357526	-2.86303333499232	1.18407411148586
C	-6.96861685029545	-1.49163978499932	1.23972542949968
C	-7.20425344118141	-1.41708509212472	-1.15156691932879
C	-7.12059252117381	-0.72391021750286	0.07003873559211
H	-6.70709331021172	-3.42273587171146	2.10828319094846
H	-7.15411178546590	-3.29264403823129	-2.17188043651849
H	-7.29868380312451	-0.87142118435688	-2.08369755845774
C	-7.39950252818904	-5.81779326315635	-0.96861909343148
C	-5.65042245056771	-5.57281246693734	0.69741545462587
C	-5.33880223565504	-6.90435439060352	0.59963914890724
C	-7.17740931302884	-7.17656745039258	-0.99147271429955
C	-6.11235585796801	-7.73994450559993	-0.24758684643131
H	-5.08132524671437	-4.94273629123360	1.37405079718668
H	-4.54595669598267	-7.31290152767852	1.21472298515016
H	-8.20593729603770	-5.39501881812408	-1.55974224999210
H	-7.79801181004285	-7.83818509224091	-1.58801302222731
N	-6.02964290482911	-9.10303038979459	-0.31478989469575
N	-4.96227722164822	-9.73947022273227	-0.01158749043218
C	-3.61023005722420	-9.26703704250886	0.04626242485574
C	-3.12134702494131	-8.52683656373588	-1.03142075258161
C	-2.80021099134253	-9.59591300921389	1.13048169457927
C	-1.49584028510885	-9.11842293282114	1.16174788820565
C	-1.80792396628024	-8.09858759290174	-1.00562656812423
C	-0.98248701711677	-8.36077349867039	0.10021961067043
H	-3.18875222042283	-10.19358132047446	1.95057102573180
H	-0.85923413335327	-9.35866102275468	2.00815608326110

H -3.75916146622704 -8.30550645777256 -1.88176071134398
H -1.41534203172078 -7.52777260999647 -1.84195737827564
C 0.38377917557825 -7.80873286783795 0.13059428074260
C 0.83641144905776 -7.09304268845586 1.24487365536580
C 1.23562620747338 -7.92713832204365 -0.97511726912758
C 2.48448070111028 -7.32964782758910 -0.97444217744293
C 2.06828241528744 -6.46115263729877 1.22589019906179
C 2.91939657602138 -6.55030140914714 0.11127457807067
H 0.92782653021478 -8.52102258699802 -1.83140815749477
H 3.13061004470155 -7.48610120863241 -1.83138890417389
H 0.18980204438845 -6.97788274144393 2.11053553086493
H 2.34586760802078 -5.85409091877971 2.08039193819497
H -6.97835287442889 -1.01330729276859 2.21275386247104
H -8.05763897197286 1.03581391820077 -1.82207146293212
H -6.75821596850859 9.79556172837358 0.03961200348497
H 11.86184729210777 0.95692694434143 0.04648962162514
H -5.10117364266739 -10.74802316697241 0.03796308207436

127
3CAB-1_all-trans_1H, Eel(r2SCAN-3c) = -3100.685766626981 a.u.
C -8.40773599168208 3.50492577205918 0.23055904097756
C -7.79291099149098 4.83423303177014 0.23020622814776
C -8.27407904003344 2.67565815066844 1.35728392473212
C -8.97663715499163 2.94153499470880 -0.92644328820171
C -9.27570627398300 1.59330909361098 -0.99008162819619
C -8.57054440164997 1.32737271024557 1.29125600887253
C -9.01446497704890 0.73916247672866 0.09617857956065
H -9.17952282627155 3.56845025876276 -1.78999960579322
H -7.82684022020999 3.06585691708540 2.26624843815486
H -8.35054850144368 0.69811928411271 2.14803498086155
C -7.60528810299517 5.56929034262603 1.41693532090117
C -7.19940158380277 5.33454545990048 -0.94752544854735
C -6.35004823693100 6.41985594111118 -0.92946954826823
C -6.76169226295867 6.66065328900382 1.45422614479698
C -6.07919263011140 7.05178265743039 0.29229646177885
H -7.32829575443666 4.79518422180755 -1.88018068893568

H	-5.83067486164984	6.73569670731098	-1.82642712785288
H	-8.10955316372740	5.26263901924282	2.32755489030682
H	-6.59826902340453	7.18997547634344	2.39036611816265
N	-5.04557685597999	7.97299538789114	0.41658442051735
N	-4.31955801537390	8.34489712384612	-0.59362877343458
C	-3.06487875579769	8.81361245828620	-0.35759324892156
C	-2.37631828463367	8.78256851259735	0.88371762136282
C	-2.34235163819712	9.21027375557378	-1.51133915302624
C	-0.98712152035942	9.42184285865914	-1.45397423262157
C	-1.01850936162360	8.98794706381282	0.92328384988689
C	-0.26867861704913	9.23778384890662	-0.25073012806487
H	-2.88314479436938	9.29325920035046	-2.44890934231675
H	-0.45823528799756	9.70137770125180	-2.35924943756460
H	-2.86856643363372	8.49095416880875	1.80733639376490
H	-0.49996579646261	8.84583618804054	1.86561746001247
C	1.18272419528361	9.10328657026666	-0.22846364594399
C	1.85764089499471	8.61054544841724	-1.36178852643060
C	1.93714532721467	9.27170689222680	0.95070958407379
C	3.24901075041414	8.84893246986597	1.02504925911708
C	3.16729558942681	8.18149170200698	-1.28311572707697
C	3.87581643637894	8.22337754300143	-0.06992269830065
H	1.48564218116305	9.73890178545928	1.82093833905264
H	3.79920767395116	8.98727079735142	1.95094891430068
H	1.31615412806863	8.45437997633595	-2.28908395831490
H	3.61481979642543	7.70348600561318	-2.14865785239194
C	7.18542415929511	5.53206398056620	0.21703014289404
C	8.01342892331549	4.31845272015601	0.22575961545081
C	6.41102356872637	5.85649980335156	1.34252438254618
C	6.99548608199083	6.30084374609409	-0.94534636452972
C	5.99040188341042	7.24755362504509	-1.01888328556462
C	5.40969517432344	6.80731084870870	1.27155063721026
C	5.13113911376179	7.47832792148390	0.06993020547198
H	7.63545201421345	6.13798645969167	-1.80756693643828
H	5.86009014980534	7.81146709285422	-1.93817940681317
H	6.52254293164708	5.27901072060379	2.25479950404288
H	4.76212634897750	6.95033379490240	2.13122722978407

C	8.58995138975881	3.82052832628607	1.40714032924776
C	8.07434041464442	3.51661147919279	-0.93070672288349
C	8.57196100374762	2.23092614860930	-0.88586234702136
C	9.08548788333202	2.53103698124807	1.46318195538785
C	9.03393787906143	1.70259928559997	0.33194792044446
H	7.62888821075263	3.87622050216821	-1.85365918314654
H	8.53132778080281	1.58886283511813	-1.75896467752092
H	8.61495037923216	4.44060923220243	2.29843690069345
H	9.47185016932381	2.11969945351014	2.39112205599699
N	9.24722975743242	0.33486826667515	0.55469915201171
N	9.28147108760283	-0.38516894831245	-0.47930428570368
C	9.07607847472593	-1.75509296588286	-0.26834987284418
C	8.62082277354774	-2.30295479232813	0.94399373435230
C	9.13503687397399	-2.56896713509674	-1.40959156905842
C	8.64848156318098	-3.86289050388596	-1.37022963313231
C	8.12970547317812	-3.59088490944392	0.97089222646704
C	8.07020728185287	-4.37741203797658	-0.19724695859469
H	9.51842611286837	-2.14267749502930	-2.33205208730987
H	8.67576939237257	-4.47189963499468	-2.26891478530870
H	8.57957865220279	-1.67449669618770	1.82687871791898
H	7.68657113828528	-3.96732090854730	1.88811055666018
C	7.24255751313828	-5.59284315894312	-0.20636738655306
C	6.45808253726133	-5.88705825875938	-1.33200205880370
C	7.05413759704042	-6.38037231546623	0.94178793276182
C	6.04383705452110	-7.32517091336104	1.00111646328867
C	5.44766795776217	-6.83080851095389	-1.27181416681356
C	5.17463246180673	-7.52400157316295	-0.08378796833333
H	7.69773349446288	-6.23610813576042	1.80500722699351
H	5.90970981906629	-7.90508689772328	1.91001755653909
H	6.56659046616217	-5.29116802477687	-2.23303775618899
H	4.78922193480512	-6.95295971062493	-2.12664360748054
C	-8.38406217454133	-3.48173789489048	-0.18433985776059
C	-7.75702899700530	-4.81147823238172	-0.19388764756668
C	-8.27596688368401	-2.65306295426487	-1.31210698492347
C	-8.94074157594473	-2.92473799051556	0.98021844554873
C	-9.24472377002037	-1.57689479204359	1.05209371413067

C	-8.58470701392778	-1.30639459507525	-1.24217371074178
C	-9.01333802132275	-0.72362925527154	-0.03989546292573
H	-9.11870826309551	-3.55617281281842	1.84585791410524
H	-7.84090753435893	-3.04502184209415	-2.22613311265047
H	-8.38442490253716	-0.67655802392763	-2.10378140969288
C	-7.63153012849665	-5.56595204167896	-1.37296052509407
C	-7.08405325282125	-5.26453942935903	0.95747269252899
C	-6.22661988892208	-6.34392836044068	0.90847330103655
C	-6.76818148662734	-6.64450450727797	-1.43393262549713
C	-6.01548770563295	-7.01355587705789	-0.30906935251510
H	-7.15986961735239	-4.69407994472206	1.87856288906207
H	-5.64322902590822	-6.62972365300277	1.77694279244443
H	-8.18830302407483	-5.27747538483519	-2.25971434079565
H	-6.61698503423535	-7.18850351565757	-2.36172150917663
N	-4.94278615986299	-7.88684767784896	-0.53934919900303
N	-4.32425684400483	-8.27149714846555	0.48882526686998
C	-3.03645014229780	-8.77801553296912	0.26688206341550
C	-2.34140140360402	-8.64449250332047	-0.94796805148299
C	-2.35382230089167	-9.24528548285653	1.39991432566019
C	-0.98979539080323	-9.46793619884203	1.35045937217386
C	-0.97991665263527	-8.85977102305963	-0.98467774044407
C	-0.26209568220455	-9.21136322699405	0.17590608790719
H	-2.90898294020664	-9.37362604411363	2.32455972642967
H	-0.47025202990295	-9.80341333227420	2.24313822948205
H	-2.87062346067936	-8.28592906988273	-1.82408951564519
H	-0.43790998318244	-8.65326662102340	-1.90283327243430
C	1.20411827511587	-9.09692153582074	0.17878972260352
C	1.85442204269556	-8.57113166142879	1.30553049845695
C	1.97584554739125	-9.31540576015857	-0.97472579282596
C	3.29730937001337	-8.90689479664988	-1.03715748938893
C	3.17513827257545	-8.16245141953849	1.24250444523897
C	3.90671888956664	-8.26020144081810	0.05017322062505
H	1.52681613326859	-9.79526353572015	-1.83976900055169
H	3.86282035624904	-9.07095755061162	-1.95023263964925
H	1.28740775107714	-8.37658817193352	2.21071620632980
H	3.61217915848371	-7.65850677536330	2.09939505755462

H	-9.65559987846276	-1.17355167637823	1.97344735855974
H	-9.70294045053163	1.18630647848920	-1.90189811637937
H	-4.78188543162264	8.21575129110516	1.37317183108084

128

3CAB-1_all-trans_2H, Eel(r2SCAN-3c) = -3101.031795191500 a.u.

C	-8.51795697348655	3.55975397881989	0.18859931872731
C	-7.90737068512082	4.88738981028170	0.18359120603827
C	-8.40524300943894	2.74551157131246	1.33048972614482
C	-9.05841960065482	2.97673394714585	-0.97450777610682
C	-9.34228206426846	1.62637782287317	-1.02799967699021
C	-8.68442648591565	1.39449691205951	1.27367290164582
C	-9.09115382165738	0.78649380345072	0.07377245726987
H	-9.25457349506044	3.59075357377280	-1.84853076045209
H	-7.98733111455196	3.15031433848267	2.24665506699096
H	-8.48008068686572	0.77991383666820	2.14451599174097
C	-7.71763635513664	5.62596099753806	1.37004502665399
C	-7.31618773464512	5.38639334410544	-0.99900720473843
C	-6.45594456467147	6.46072302509019	-0.98187091316047
C	-6.86390308679584	6.70750720378430	1.40575187570395
C	-6.17552708000557	7.08637753145462	0.24207442281600
H	-7.45575613499077	4.85224036816430	-1.93278639337400
H	-5.93770675207437	6.77354513613556	-1.88067911606456
H	-8.22763939597389	5.32861389424476	2.28018951328974
H	-6.69706608813183	7.23815809755543	2.34054022650437
N	-5.12044047763266	7.98107319294668	0.37143552435051
N	-4.37592968974738	8.33223108993499	-0.62211001238004
C	-3.11590959986838	8.79313022811056	-0.37256586897510
C	-2.44137576768626	8.74204911932104	0.87343792104882
C	-2.38303301066373	9.18377342640888	-1.51930751914665
C	-1.02433620971843	9.37921238306165	-1.44964586869891
C	-1.08049979209083	8.92681752296507	0.92469098008191
C	-0.32126514714395	9.17470428711555	-0.24292425098353
H	-2.91506371045787	9.27828167712635	-2.46077143339649
H	-0.48428741015657	9.65846152410266	-2.34836499151505
H	-2.94552634941291	8.45028277780752	1.79063582466810

H	-0.57044582244001	8.77122975550835	1.86945799559914
C	1.13168883765169	9.01864034827520	-0.20996769149816
C	1.80125477554211	8.50299681455948	-1.33447465939071
C	1.87975559781227	9.19245882290826	0.97082583968030
C	3.18774677737509	8.75519183883165	1.05570509509053
C	3.10816882764123	8.06311049183879	-1.24648350011365
C	3.81004502392140	8.11710174718476	-0.03207400666047
H	1.42922503777948	9.67442936354161	1.83339768851324
H	3.73622710394734	8.89625580487643	1.98240933751004
H	1.26109871919752	8.34402854263980	-2.26205453903580
H	3.55744137554408	7.57492865767734	-2.10568911605709
C	7.14054752915067	5.44910182688825	0.26896306168661
C	7.99428512186132	4.25437482394734	0.27477154389258
C	6.32612879828919	5.72498462067531	1.37890776600144
C	6.97192913155446	6.24286829449855	-0.87917477716634
C	5.95667055060241	7.17960183161956	-0.95410627804172
C	5.31380769068975	6.66431611217422	1.30483890545133
C	5.06588324833311	7.36729977344366	0.11623376584527
H	7.63983684587239	6.11860243720593	-1.72656167546405
H	5.84439417506181	7.76956847059011	-1.85916399078453
H	6.41761658172622	5.12641561325061	2.27983612822254
H	4.64071838855030	6.77953034587609	2.14885240782902
C	8.53211705320268	3.73125701484762	1.46462601890348
C	8.14597771283717	3.49634689982508	-0.90351031513160
C	8.67894099170628	2.22446213239907	-0.88443537939486
C	9.07191790131082	2.46041496562220	1.50316609419273
C	9.08933061994302	1.68006840748775	0.33890619736401
H	7.74839459320911	3.87648945418631	-1.83889569889702
H	8.71118968338678	1.62117441275460	-1.78388768992519
H	8.50566450802454	4.31997730007389	2.37587810911041
H	9.44762588144425	2.05784322487654	2.44115219659721
N	9.38957510022963	0.32320444119395	0.46420768314151
N	9.36079172997354	-0.48508645754275	-0.54407838598436
C	9.19268633009608	-1.81462544086828	-0.33132488591715
C	8.89462335557216	-2.44093468290394	0.90917822075938
C	9.14577818304773	-2.60470731625300	-1.50924193117868

C	8.67533281235467	-3.89211684402680	-1.47159500786709
C	8.41397483721834	-3.72501746302302	0.92972627201307
C	8.20426810929286	-4.46230356178360	-0.26489443058833
H	9.44439099202279	-2.14409283191485	-2.44575214046009
H	8.63102024536082	-4.46305899115864	-2.39265761387255
H	8.94229679141050	-1.90384837939795	1.85244383525557
H	8.09165997412019	-4.14146990105515	1.87779654552798
C	7.36727346204655	-5.65072630718252	-0.24962073597166
C	6.60530989898361	-5.98628203689183	-1.38803394370457
C	7.13139272951623	-6.39499590011513	0.92740250190757
C	6.10582028233483	-7.31354028187871	0.99657277493583
C	5.57622862418159	-6.90049032404697	-1.31465392068961
C	5.24852295458233	-7.53340272748971	-0.10125980177094
H	7.76297198348787	-6.25060540491995	1.79852823109797
H	5.95037383347054	-7.86499653365484	1.91870798536019
H	6.74621267352917	-5.44290019723219	-2.31613362199254
H	4.94419979555062	-7.04657422807206	-2.18442438647498
C	-8.38870448106844	-3.42215380258766	-0.16474402032508
C	-7.74140888687188	-4.74038742603362	-0.16368781234629
C	-8.30364689941688	-2.60558982935390	-1.30416221150609
C	-8.94898802511273	-2.86274989730080	0.99808193602408
C	-9.27746826293942	-1.52119222094912	1.05623005997048
C	-8.63468207517061	-1.26446313458377	-1.24709307782906
C	-9.06530868675974	-0.67552723324916	-0.04720910614413
H	-9.11549574967165	-3.48805397486132	1.87023889384900
H	-7.87198199120716	-3.00089283242661	-2.21820788419911
H	-8.45372990593666	-0.64294598145205	-2.11862752703246
C	-7.61263167626407	-5.50647963885919	-1.33598079498870
C	-7.06357238353211	-5.17491002059000	0.99389049612151
C	-6.19623892037631	-6.24498503495114	0.95545343795235
C	-6.74318362461212	-6.57942882969484	-1.38564210618393
C	-5.98472169153501	-6.92728395940250	-0.25644187114928
H	-7.14978590236152	-4.59937768043589	1.91065951913398
H	-5.61458703560549	-6.52092116433457	1.82825425575995
H	-8.17654694023055	-5.23516151854112	-2.22330723381638
H	-6.59375401498887	-7.13759428016449	-2.30511222007493

N	-4.91687986251197	-7.80284477513633	-0.47858575548551
N	-4.27150632463087	-8.15660422839113	0.54292570793928
C	-2.98402748400334	-8.65290129855014	0.31383435665802
C	-2.29808167378247	-8.50432980268714	-0.90516680513676
C	-2.29460969253586	-9.13215342337258	1.43935630169052
C	-0.93261310128068	-9.35559022805920	1.37754043392690
C	-0.93930896318862	-8.72825829757694	-0.95627967699849
C	-0.21463950670474	-9.09598155138907	0.19595021322709
H	-2.84425057488368	-9.27426826353986	2.36508788087924
H	-0.40960501665772	-9.70676615234037	2.26191204371525
H	-2.83451350175362	-8.13920731221225	-1.77418338701655
H	-0.40689282752417	-8.52246134969033	-1.87999952166384
C	1.25005205228175	-9.00648025785356	0.18131765998447
C	1.93355982076910	-8.53748129479684	1.31587466186574
C	2.00005017933518	-9.21196561905806	-0.99215457089817
C	3.32606212138516	-8.83232931389064	-1.06577024644741
C	3.26170352504255	-8.16297737510887	1.24475345959251
C	3.97142066411381	-8.23573360495553	0.03374410283985
H	1.52882022353164	-9.66323639801314	-1.86012647289666
H	3.87138011782454	-8.99334643097231	-1.99108861852139
H	1.38863258940569	-8.36380703334257	2.23821249302803
H	3.72250219515954	-7.70519986255321	2.11449186400305
H	-9.69600901375019	-1.11751663469234	1.97365751222811
H	-9.75116573523595	1.20642426055606	-1.94205230585045
H	-4.87305024692678	8.23429775222431	1.33158771757447
H	9.46852352509704	-0.02482224653326	1.42202994339851

129

3CAB-1_all-trans_3H, Eel(r2SCAN-3c) = -3101.325798473946 a.u.

C	-8.39767234124777	3.47204604404631	0.23056836691587
C	-7.77972383537771	4.80302632018151	0.23694993905715
C	-8.25201371979502	2.63542880108607	1.34973829701846
C	-8.98574805048599	2.92520593284559	-0.92472286297884
C	-9.29126785986451	1.57856684815961	-0.99697617690950
C	-8.56124109354777	1.28971506497304	1.27809325589982
C	-9.02493204232027	0.71850793152348	0.08285060808439

H	-9.20115072474765	3.56125410005975	-1.77816184222868
H	-7.79719674212571	3.01744984837299	2.25811375559210
H	-8.34392799598055	0.65450843722482	2.13115249154653
C	-7.59342774746518	5.53047781532966	1.42770281001734
C	-7.19225310842695	5.30775885015417	-0.94275254884601
C	-6.34123622205432	6.39125392096373	-0.92110269660963
C	-6.74612275910442	6.61970436149159	1.46881045641932
C	-6.06862702321379	7.01140089338016	0.30524600542691
H	-7.33189006497818	4.77872260685352	-1.87959472862720
H	-5.83099796719172	6.71572367693310	-1.82037501211242
H	-8.10058225606716	5.22364905810767	2.33648241066376
H	-6.58225832350979	7.14544302973680	2.40682074145115
N	-5.01966719945694	7.92332407253386	0.43896733113193
N	-4.28723795859628	8.28481105336333	-0.55123569956040
C	-3.04549577638970	8.79796952540646	-0.32745917703005
C	-2.36364263851070	8.83444810455272	0.91658010197826
C	-2.33189953826462	9.14696487932333	-1.50089163175158
C	-0.97812851244665	9.37267323069205	-1.45471856874436
C	-1.00787694173013	9.04391295511707	0.94483583261067
C	-0.26031552132580	9.23273375969445	-0.24588700352852
H	-2.87465088771533	9.18374836162205	-2.44033014357780
H	-0.45350736196668	9.62117368563163	-2.37093885031642
H	-2.86029259537984	8.60494852951219	1.85537622517773
H	-0.48967854221360	8.96048386487426	1.89391960076802
C	1.19161310977981	9.08957865721875	-0.22382344016429
C	1.85495560168935	8.56589717494871	-1.35044998750902
C	1.95121911793419	9.28212063243805	0.94882010340874
C	3.26137306699205	8.85258608569235	1.02595309187989
C	3.16384952073266	8.13408853243889	-1.26986653104395
C	3.87555345741080	8.20299456244601	-0.06096002271730
H	1.51064142711723	9.77589080288581	1.80952962425003
H	3.81876502239077	9.01049720214986	1.94442899266900
H	1.31080868922686	8.39630142915095	-2.27337957819410
H	3.60869361924149	7.64270494265325	-2.12933111939816
C	7.20514035124455	5.53792235991481	0.23091861365602
C	8.04832565981023	4.33688174712891	0.23753625182265

C	6.40800171303785	5.83080163460527	1.35005597199491
C	7.02574513969952	6.32019632673831	-0.92467088824251
C	6.01240521276662	7.25818881964318	-0.99736749444011
C	5.39737487837588	6.77161366111963	1.27800789777636
C	5.13445261462317	7.45813687821846	0.08240615311933
H	7.68425869999092	6.18815670048528	-1.77803725678997
H	5.89262690799163	7.84017057018567	-1.90647718006743
H	6.51139665685280	5.24632886714324	2.25871117965806
H	4.73870647498796	6.90156520183569	2.13105898150109
C	8.58507685638590	3.81183095876482	1.42831976330395
C	8.19109810369157	3.57538342363014	-0.94201129304851
C	8.70342381837620	2.29646010920555	-0.92017364039393
C	9.10417179270802	2.53320112063635	1.46962470460227
C	9.10422302306615	1.75041136700502	0.30622213065961
H	7.80269978825471	3.96073803450513	-1.87886831560277
H	8.72886940528580	1.69211423866910	-1.81930127503531
H	8.57327614058617	4.40466206518830	2.33696759496784
H	9.47745562384265	2.12844700433662	2.40768404345834
N	9.36915902891835	0.38596539180790	0.44002684067280
N	9.31605062342075	-0.42905126988261	-0.55018874165689
C	9.13925402460709	-1.76098209227828	-0.32639658539710
C	8.82915798419529	-2.36944255952935	0.91758212845649
C	9.08531515844328	-2.55372101337549	-1.49969765625748
C	8.60416647058251	-3.83908066657757	-1.45347628115744
C	8.33288181110615	-3.64836656997576	0.94586424551787
C	8.12360998990159	-4.39061299220035	-0.24477519499298
H	9.38896488303161	-2.10224729097442	-2.43908098368029
H	8.55767474398563	-4.41785498031738	-2.36960713968229
H	8.87796782478343	-1.82431710403792	1.85626144371945
H	8.00102669842193	-4.05518828256552	1.89486744994740
C	7.27416740597968	-5.57682342305571	-0.22285606858453
C	6.48991583061217	-5.89030326930770	-1.34989489417893
C	7.06055195205800	-6.33044673074299	0.94998149025646
C	6.03360105061800	-7.25043772981545	1.02694775353387
C	5.46170539992146	-6.80816688515222	-1.26952829291963
C	5.16467514584183	-7.45823816430000	-0.06043697504676

H	7.70786601654835	-6.19516466036935	1.81103438966968
H	5.89113879124266	-7.81161927464081	1.94564172202373
H	6.61570534533274	-5.33474874923416	-2.27304646318011
H	4.81442684520497	-6.94836016804926	-2.12941608553190
C	-8.46727514530512	-3.51197658384140	-0.22352196341956
C	-7.86534924755206	-4.84098484797096	-0.24595044346732
C	-8.34639159223427	-2.67572448555577	-1.35031286403223
C	-9.01274834607001	-2.95023031897000	0.94953948366969
C	-9.29546891225915	-1.60076987904573	1.02689515498226
C	-8.62663838154755	-1.32625101578770	-1.26954433866410
C	-9.04083888917069	-0.74415970232321	-0.06028713774130
H	-9.21947089083956	-3.57854269471693	1.81047695877446
H	-7.92845016732296	-3.06226338870014	-2.27366195591206
H	-8.42427266534444	-0.69545977134010	-2.12929726590767
C	-7.62802092338123	-5.53242489140152	-1.45497027017028
C	-7.32763834926252	-5.39421995665085	0.94449072747144
C	-6.46841889319609	-6.46365212167832	0.91576672326575
C	-6.75563226786426	-6.59193185815970	-1.50162630261045
C	-6.09633684999844	-7.03574166349500	-0.32846558647918
H	-7.51407106401550	-4.90393903786229	1.89375693168549
H	-6.02098226801525	-6.77916897670507	1.85433666111014
H	-8.10580802458575	-5.20225408644406	-2.37101022042558
H	-6.51638809124389	-7.08014998793695	-2.44124506907379
N	-5.03123476782407	-7.85467271548123	-0.55257658200761
N	-4.35214366691991	-8.30883617356630	0.43744913590174
C	-3.03797616737558	-8.76148870020671	0.30362619873808
C	-2.36452021342305	-8.68698928846911	-0.92264330299482
C	-2.36017435545949	-9.15295073616952	1.46707670947953
C	-0.99323996482875	-9.34224337226416	1.42594866493465
C	-1.00069720792903	-8.88238492685221	-0.94431068076696
C	-0.27000313568983	-9.13943692311562	0.23533700941410
H	-2.89746802030202	-9.27423854175701	2.40500749721465
H	-0.47399137740173	-9.62852944206932	2.33460319037082
H	-2.90059056608503	-8.40679530723650	-1.82177596164883
H	-0.47261369684494	-8.73842217155109	-1.88104122933510
C	1.19166034766664	-9.00918259539524	0.22924553404779

C	1.84295661631225	-8.46453851044608	1.34855463492611
C	1.95966349978441	-9.24536669336219	-0.92569939553504
C	3.27872518954769	-8.83677863479763	-0.99751467181138
C	3.16303973239113	-8.05960597245921	1.27735543718101
C	3.89000540533272	-8.17589079509007	0.08238977274183
H	1.51679493316820	-9.75008156422741	-1.77919609622179
H	3.84337337921638	-9.02449857884228	-1.90606067530894
H	1.28430409200714	-8.26142254537635	2.25662688340572
H	3.60420750364547	-7.55361103979358	2.13044178905821
H	-9.71001778244486	-1.19692456589567	1.94574179383274
H	-9.73581398104756	1.18362112033257	-1.90577549235682
H	-4.77517675820917	8.17364718867944	1.40143525849269
H	9.46358405994565	0.04904196332265	1.40250275436697
H	-4.69133629537408	-8.22262139947915	1.39990313627481

126

3CAB-1_all-trans_c1, Eel(r2SCAN-3c) = -3100.291366043899 a.u.

C	5.57132734938919	7.23820899234350	0.53716505019317
C	4.36127942344599	8.07356071613528	0.43978798254481
C	5.96458985287179	6.46217755509539	-0.56242458673548
C	6.24610191165278	7.03299343130406	1.75077292490589
C	7.17701882227408	6.01508894382335	1.88807188253022
C	6.89614764557056	5.44633528426080	-0.42546387615411
C	7.47516610809334	5.15869567037685	0.81763383540973
H	6.01876099726259	7.66476851773577	2.60509651454836
H	5.45233685062486	6.58115193933716	-1.51247894856021
H	7.09408481657327	4.79353278616890	-1.27074897464963
C	3.97249457356334	8.67988142375000	-0.76516694992967
C	3.45382501954666	8.10136776473936	1.51526910652203
C	2.17438430666819	8.59787205472953	1.36366003117059
C	2.68654749432598	9.16582919852871	-0.93105263526181
C	1.75676535808250	9.08304070160972	0.11383260260808
H	3.72762736494328	7.62640752248097	2.45303991939230
H	1.45322887732661	8.52817210681636	2.17072924288303
H	4.67216397539379	8.72428053706969	-1.59495108365149
H	2.35886114587709	9.56512221398363	-1.88666565279976
N	0.40941145680351	9.28867109091009	-0.23678283059845

N	-0.40315866860615	9.26297148084614	0.72608869245547
C	-1.75045052788080	9.07630768117381	0.36487229494504
C	-2.16760658417901	8.65778175314840	-0.90895920987534
C	-2.68059187109507	9.10380790607034	1.41235672155064
C	-3.96655054552951	8.62754161265107	1.22046115369656
C	-3.44701402926130	8.17011217194491	-1.08711902165698
C	-4.35494531824864	8.08577614317826	-0.01499501359794
H	-2.35325501192414	9.45199410543882	2.38786929177680
H	-4.66659403174579	8.62814258808286	2.05111760489453
H	-1.44606110370665	8.63069373288672	-1.71823926863722
H	-3.72044680651117	7.74532530008719	-2.04876483568352
C	-5.56518031458625	7.25709546869263	-0.15683106404993
C	-5.95946282883801	6.42469220778466	0.90035464285560
C	-6.23923575713010	7.11602416108526	-1.37994215360534
C	-7.17060810760641	6.10721384239285	-1.57087479872492
C	-6.89153589482184	5.41800502365262	0.70990131312743
C	-7.46998250401917	5.19623927929146	-0.54687456908055
H	-6.01098214009254	7.79150462611536	-2.19989763958056
H	-7.65646099702104	6.00535835336755	-2.53764776378272
H	-5.44772279691837	6.49349585165067	1.85562624809208
H	-7.09042877606901	4.72196991958060	1.51973227839847
C	-8.99755612718682	1.23378049969141	-1.09598221184294
C	-9.11059813471511	-0.23474156084702	-1.14252423055615
C	-8.33250452443162	1.90811710201166	-2.12989453913921
C	-9.35851199591569	1.97862559651969	0.03765528277462
C	-8.96057375633433	3.29856562643557	0.18248093620922
C	-7.93663125396486	3.22758797805649	-1.98576893577326
C	-8.18561290294726	3.93209537242882	-0.80046425205739
H	-9.93780104319070	1.50790304335347	0.82729968716802
H	-9.23143491072301	3.84185599199511	1.08390236612863
H	-8.02194315062422	1.35966611614752	-3.01415769257605
H	-7.32663079005164	3.68375825979023	-2.76014310921686
C	-9.23348256090235	-0.93222857230195	-2.35460022649990
C	-8.88109637476208	-0.98160671232215	0.02813848524293
C	-8.66088130953913	-2.34382078208399	-0.01978336678651
C	-8.99863955893930	-2.29554120299563	-2.41439377925195

C	-8.65829252442378	-3.00805962476450	-1.25696032345368
H	-8.76880926408053	-0.46217186581499	0.97552827715179
H	-8.39157169810313	-2.89387878748577	0.87531107481533
H	-9.46679334896743	-0.38927012159297	-3.26608925003678
H	-9.01684682144100	-2.82477805912120	-3.36285871812567
N	-8.12140962113079	-4.29377003539869	-1.45506598795731
N	-7.87230363804743	-4.93705069235886	-0.40044030603713
C	-6.99721766501370	-6.02765729596584	-0.55948091102768
C	-6.21664145788264	-6.24310773046298	-1.70669710600044
C	-6.75073747545212	-6.79434345960703	0.58707380420934
C	-5.68612864048635	-7.67901757763505	0.62321170866065
C	-5.14786607040511	-7.11582514184565	-1.65649741734335
C	-4.82035572392021	-7.80614144168216	-0.47448786326496
H	-7.37608718870312	-6.63633631952827	1.46113322376564
H	-5.49067090311909	-8.24380145558862	1.53032917137703
H	-6.40346637700437	-5.64545738564231	-2.59224529326077
H	-4.48532609192242	-7.18826049483554	-2.51426741244678
C	-3.49874866026542	-8.44692316605111	-0.35494889508648
C	-2.77578317907546	-8.32045442803908	0.83977075261656
C	-2.84050824843330	-9.02025733304582	-1.45415005202452
C	-1.49101924537907	-9.33192076579462	-1.39654359785881
C	-1.42783463384780	-8.63378915371366	0.89767747996283
C	-0.74089291099875	-9.08550560226158	-0.23692919139697
H	-3.38932670013894	-9.20048536523690	-2.37448053948138
H	-1.00170064071515	-9.74908164340070	-2.27268559391645
H	-3.24728516994309	-7.86484750869973	1.70537179665263
H	-0.87392082010490	-8.41834962199792	1.80686030811598
C	8.99946609976151	1.17159208657848	1.15708503859928
C	9.11091945683940	-0.29747087462726	1.12648048345899
C	8.33590405311834	1.79139020667596	2.22550259011919
C	9.36032159401822	1.97455518973574	0.06380508406025
C	8.96360915086896	3.30066118679839	-0.01117407777001
C	7.94127490883031	3.11700065834167	2.15120381873766
C	8.18999997619488	3.88248103422332	1.00426412613119
H	9.93840868293110	1.54535904887312	-0.75001527012257
H	8.02545365142593	1.19756164996158	3.07999406919967

H	7.33223422415915	3.53241421132573	2.94891073822563
C	9.23359230124198	-1.05762758750356	2.30027909245734
C	8.88012506597297	-0.98170014812366	-0.08160508157612
C	8.65845851466086	-2.34432839869697	-0.10503502379587
C	8.99727222179118	-2.42194584403241	2.28866270964943
C	8.65565030731873	-3.07247059715644	1.09564087388619
H	8.76810083856463	-0.41318566975315	-1.00041909093876
H	8.38823313991868	-2.84643608674140	-1.02761226132341
H	9.46790021309288	-0.56341864381755	3.23884609651084
H	9.01532877656179	-3.00018808642921	3.20807665845662
N	8.11747009493420	-4.36623874486881	1.22640179583683
N	7.86684959891620	-4.95297081582983	0.13965740830853
C	6.99101740649299	-6.04977162607492	0.24193163062926
C	6.21052476629608	-6.32395145238712	1.37660234300616
C	6.74390668248073	-6.75582867787609	-0.94279574791184
C	5.67895463825209	-7.63705031809333	-1.02441201751582
C	5.14136716431697	-7.19244792904288	1.28154104357579
C	4.81341354812407	-7.82058227002256	0.06548614931995
H	7.36918319179078	-6.55309101962643	-1.80762467125846
H	5.48310600716910	-8.15414043653643	-1.95945678470853
H	6.39784927074826	-5.77303010897185	2.29185270613895
H	4.47900494602508	-7.30901963437372	2.13457307304468
C	3.49171431002078	-8.45420667978683	-0.08664335866691
C	2.76859252568556	-8.26642177466982	-1.27318160717497
C	2.83351777241253	-9.08311791750799	0.98174498019408
C	1.48395762231268	-9.39117970157218	0.90840351097221
C	1.42061397629076	-8.57624447965743	-1.34696327832929
C	0.73374307346475	-9.08549783103264	-0.23694920351396
H	3.38238528454407	-9.31037055322875	1.89158046354847
H	0.99469556904100	-9.85271457485320	1.76203810956756
H	3.24004575793440	-7.76698737451696	-2.11428521984535
H	0.86660957045255	-8.31438442912905	-2.24382765558127
H	9.23423578010128	3.89020766625672	-0.88311232009725
H	7.66341064365635	5.86258900914073	2.84788506617864

126

3CAB-1_all-trans_c2, Eel(r2SCAN-3c) = -3100.289859279134 a.u.

C	1.68320932591333	-9.04175041420972	-0.23144792924478
C	3.11834342322853	-8.70679878456625	-0.22675802102507
C	0.90342772680532	-8.71956446373849	-1.35150553406632
C	1.01365917340784	-9.49634710052046	0.91552893948967
C	-0.37116613921051	-9.50445859992092	0.97942050173164
C	-0.48005813880003	-8.73119774540199	-1.28873159428138
C	-1.14744351985805	-9.06166216841345	-0.10192188036469
H	1.58744373327198	-9.82361037914604	1.77827534407679
H	1.38490096899630	-8.34665438339457	-2.25045217504189
H	-1.05063031321762	-8.36959539273237	-2.13934491728811
C	3.88510471688484	-8.71584530453150	-1.40343619742263
C	3.69628626845763	-8.15687360450019	0.93236751434904
C	4.92803298941772	-7.53309478727963	0.89471991505910
C	5.11037580445227	-8.07429872176511	-1.45592769608526
C	5.62295682883233	-7.43121106670782	-0.32108526022236
H	3.11722082332358	-8.12454294156445	1.85078368869962
H	5.32060296496404	-7.02746982472454	1.77047537545643
H	3.48948194087765	-9.18584503438484	-2.29926534170811
H	5.66743168024943	-8.00880286434153	-2.38622499474438
N	6.67845291053250	-6.52820234793251	-0.53860673651158
N	7.20372303177335	-6.04784624781059	0.50149401803284
C	7.93842511633464	-4.86508638497020	0.30126411401287
C	7.76412674501640	-4.04070309691928	-0.82148699591639
C	8.69479934496074	-4.39190702101099	1.38191016412234
C	9.19047348653956	-3.09883559392176	1.37735872139376
C	8.23743019049563	-2.74448771348518	-0.80495978926396
C	8.91312306413790	-2.22280010659361	0.31411388621526
H	8.84288318589930	-5.04251255455267	2.23917456689702
H	9.75959407261147	-2.74587897916034	2.23242528281807
H	7.16646860664464	-4.40121976141808	-1.65169099464663
H	7.98767796151034	-2.08699336806222	-1.63194933240314
C	9.10667869162060	-0.76573757624088	0.42246148285644
C	8.98016058764933	-0.12668587310390	1.66542851574396
C	9.22044514390170	0.05301237404002	-0.71166668592621

C	9.05727489234386	1.42626151945250	-0.62412467818536
C	8.83248164034194	1.24727001421576	1.75422771258408
C	8.78711956583107	2.04801396951803	0.60373341746874
H	9.38502214114929	-0.39618383904476	-1.68673247488618
H	9.05305576061791	2.01492513893020	-1.53703126365528
H	8.90749371613502	-0.72053871329617	2.57153515782420
H	8.69403851862772	1.70086524616872	2.73141366475549
C	6.83110266982277	5.86605433498955	0.46782280279101
C	5.86836107482117	6.95687222057193	0.23720438168360
C	7.90182778591709	5.59234186032793	-0.39662540498163
C	6.56942500481619	4.94154357818647	1.48871674843560
C	7.26993994666343	3.75191398183264	1.57411833974511
C	8.61002615241094	4.40262325397761	-0.30478953760036
C	8.27325560279486	3.42961059334265	0.64915503441638
H	5.73172409486787	5.10790686667065	2.15917893567776
H	6.96085866407000	3.01531463266122	2.30908939551529
H	8.17223244301278	6.31555303319694	-1.16148455950849
H	9.42584692642697	4.21660332415938	-0.99801692279145
C	5.46745816977457	7.27480618085676	-1.06811347898568
C	5.17234048416366	7.55317275100311	1.30736410934924
C	4.03872487180279	8.31076197997628	1.09292709922993
C	4.33097482731305	8.03452207826967	-1.29276089259862
C	3.56634774134045	8.50177694927521	-0.21698729025408
H	5.50968996566937	7.37432196983223	2.32485470769836
H	3.46620552047979	8.70485118200491	1.92560977190106
H	5.98510926961906	6.82760792817535	-1.91177285352571
H	3.95894813373564	8.19776646309809	-2.30000263330700
N	1.54466840982911	9.25538100584500	0.43319249879468
N	2.27175046802528	8.94333417898202	-0.54793134992370
C	0.16876742848353	9.33914226405288	0.14177205204896
C	-0.40393537861921	8.95404297939217	-1.08136587196237
C	-0.66978800003569	9.62566005438034	1.22671098024473
C	-2.03631860875183	9.41934673331282	1.13083659787342
C	-1.76548926516701	8.74335681576573	-1.16544910043942
C	-2.60328040895304	8.90697514358300	-0.04592445275864
H	-0.21747662312154	9.94963204676649	2.15966361167895

H	-2.66851944525099	9.61037224991284	1.99339598438084
H	0.24390757908729	8.73374373251903	-1.92254559392002
H	-2.18174118326838	8.34122858633392	-2.08477841317350
C	-3.96386256059149	8.34190186246441	-0.07637939240910
C	-4.44721155907333	7.64954275321041	1.04292535615172
C	-4.73050035958857	8.29289556620570	-1.25101046565676
C	-5.85512311451492	7.48714348305835	-1.33733873346545
C	-5.57095902404398	6.84431072960362	0.95626437759021
C	-6.26193625334280	6.69449258184574	-0.25352143967042
H	-4.42501433992282	8.87513015805212	-2.11614319773800
H	-6.41355455264709	7.44877447694175	-2.26883579530153
H	-3.87076907697140	7.65154305556357	1.96325176523207
H	-5.85049849200753	6.23445846282906	1.81050800773735
C	-5.13423400606957	-7.51887208647572	0.13807873188395
C	-6.29282584688699	-6.60859294353119	0.11030972341057
C	-4.28792556621801	-7.52712318245256	1.25600881745390
C	-4.72549098021699	-8.24642037733506	-0.99057393250733
C	-3.47559948524712	-8.84348646361608	-1.04230863284366
C	-3.03996517638368	-8.12615302717118	1.20508441152382
C	-2.58050557506629	-8.74125406203439	0.03302735730629
H	-5.38751226659644	-8.32307064900565	-1.84877206537864
H	-4.56346720033434	-6.96074795479560	2.14057964828744
H	-2.36651231417584	-8.01739221306939	2.05039772583187
C	-7.00040063640262	-6.27016789024006	1.27449124248482
C	-6.57967042317847	-5.89320396316960	-1.06741179157695
C	-7.43960294876949	-4.81303647017508	-1.06131534448895
C	-7.85269211907647	-5.17887969476123	1.29357554461782
C	-8.04270985963717	-4.40685839578190	0.13994092764316
H	-6.03433647004513	-6.12798178471793	-1.97704448178164
H	-7.58160938610340	-4.21222358154242	-1.95299161650142
H	-6.84171845039146	-6.84075753535144	2.18514394934769
H	-8.34309847950769	-4.86867120497479	2.21187412095302
N	-8.65385322400425	-3.15264797458465	0.32599823563697
N	-8.86041484587529	-2.49809560976585	-0.73087139233533
C	-9.10598919123233	-1.12489053329845	-0.54468916790613
C	-8.87766064304648	-0.44380035289934	0.66190591603151

C	-9.38562549396518	-0.38692516251842	-1.70239350194293
C	-9.33544871991622	0.99665035120011	-1.68098704591530
C	-8.81560021568733	0.93562479918283	0.67082470520233
C	-8.98114077533351	1.68411884918773	-0.50956811566496
H	-9.58455301956299	-0.92503149630086	-2.62481465936365
H	-9.52501640700461	1.55325045947076	-2.59440103345174
H	-8.65596206646701	-1.01490958663327	1.55687764720724
H	-8.52861985101178	1.44535956709016	1.58619455778802
C	-8.57442310881955	3.10029728170455	-0.53065338260066
C	-7.86466015755468	3.59722631993711	-1.63298193045265
C	-8.69839653364050	3.93458669698074	0.59117009536854
C	-8.03953477871150	5.15277661495372	0.65266770874736
C	-7.20673053155407	4.81441399970907	-1.57134640698858
C	-7.22742430625567	5.59005358324841	-0.40450914986252
H	-9.30059296119245	3.61198374112698	1.43618323448982
H	-8.13374094269589	5.76556120591530	1.54522537713847
H	-7.73282450214539	2.97190821998075	-2.51093523256719
H	-6.57522385307450	5.11561453921658	-2.40213334303752
H	-3.17521619002703	-9.37629043484385	-1.94063305989083
H	-0.86154411266207	-9.83465818384660	1.89134893980203

126

3CAB-1_cis-cis-trans, Eel(r2SCAN-3c) = -3100.286927541581 a.u.

C	3.55946964251124	-5.96567936026155	-0.21750696007265
C	5.03383746146097	-6.00367827079463	-0.21257469281773
C	2.85344219478259	-5.56903436782139	-1.36039913557892
C	2.82027218821719	-6.33093252282786	0.91469103633259
C	1.43359330025426	-6.31517800419165	0.89886497167697
C	1.46768269997755	-5.53522471708210	-1.36900744564830
C	0.72394823449611	-5.91560991757784	-0.24252752370574
H	3.34164334024619	-6.67784984286221	1.80288631161813
H	3.40280633587724	-5.24391740028317	-2.23998916676777
H	0.95370670966934	-5.17451984381827	-2.25520095769561
C	5.73965072658451	-6.54005692877667	-1.29906625289383
C	5.76653232194846	-5.52755468114136	0.88420374879269
C	7.15227414272654	-5.54181039866681	0.88214155181094

C	7.12373271592352	-6.61303338717538	-1.28499951483888
C	7.83975857734250	-6.07537315413020	-0.21310102921699
H	5.23799063957752	-5.10738357058244	1.73577643886979
H	7.70691644149631	-5.14711259588177	1.72820054487993
H	5.18913775837897	-6.95163276785749	-2.14070641776432
H	7.66690940360573	-7.07689775548774	-2.10346101356274
N	9.25579770976446	-6.26612695539172	-0.19934313168786
N	10.06262724408885	-5.36011421891243	0.08237750453132
C	9.68702335112877	-3.98702383824857	0.18121529848454
C	8.87111089225165	-3.35450935401446	-0.76366213225041
C	10.31877441962783	-3.22316653527456	1.16655646542400
C	10.06316845657445	-1.86442016284882	1.26552211226175
C	8.65541592113777	-1.99104966274104	-0.68195932691069
C	9.22163158979907	-1.22074169565156	0.34648439956667
H	11.00316639262073	-3.71425128250822	1.85234162515714
H	10.55323297307104	-1.28416366556599	2.04233484922439
H	8.40695655693160	-3.93162930054643	-1.55647935108989
H	7.99559877861084	-1.51709408006098	-1.40272865464754
C	8.89151385494992	0.21045784600610	0.45833095746016
C	8.69372639509928	0.81075911235371	1.70943846273442
C	8.67680899972440	0.99485955667091	-0.68316418612780
C	8.20161546310744	2.29195740810858	-0.58036412133172
C	8.21924159861044	2.10911570775267	1.81206367226198
C	7.92102803773371	2.86291991538966	0.66840365231482
H	8.86727620858631	0.57465587861755	-1.66662378845471
H	7.98225199442047	2.85050121942584	-1.48586602791221
H	8.85748541617870	0.23145016540566	2.61393154012661
H	8.05181245712912	2.53815089691505	2.79611685004600
C	5.41248336106067	6.34356617409686	0.64460517070130
C	4.29761574539613	7.28464125015568	0.44489430770526
C	6.48936466353103	6.24144241415454	-0.24858695563395
C	5.31123885438333	5.37640837452951	1.65495689953093
C	6.17979545865325	4.29992805109253	1.70522880361507
C	7.36024495089980	5.16366920364756	-0.19658624108117
C	7.19103670289381	4.14107375121046	0.74892923466010
H	4.47525926738527	5.41113280538274	2.34694636331664

H	6.00747257302620	3.51690094216907	2.43774202964907
H	6.63491928448687	7.00869734214027	-1.00425986692543
H	8.17741278619385	5.10401639389924	-0.91039217631898
C	3.84563938539500	7.56567320818542	-0.85224362829681
C	3.52546623102404	7.74573299897418	1.52943350186946
C	2.28329309391828	8.31418358567423	1.33395105503244
C	2.60182226915632	8.13877329091604	-1.05808592284732
C	1.77613042992092	8.44700334896774	0.02961021502735
H	3.89373853433028	7.60387213407471	2.54200097569276
H	1.65889087943378	8.59090846606975	2.17653231016832
H	4.42099073043188	7.22264371888518	-1.70705658742129
H	2.20101297603841	8.25732488915691	-2.06050690473443
N	0.41936487178799	8.63906225952294	-0.28841113092516
N	-0.34936379791474	8.75567526500839	0.70380791557582
C	-1.71060321814236	8.53272594009889	0.41952982843501
C	-2.19765723056375	8.09378981074031	-0.82283453491597
C	-2.56616067749919	8.52758994749308	1.52866839090296
C	-3.82862246968356	7.96618259155305	1.43519243162785
C	-3.45613169165665	7.53274035035048	-0.90590958840924
C	-4.26708700816519	7.38703721342190	0.23505679653034
H	-2.18803632978245	8.90010579784033	2.47638483098164
H	-4.46356473096142	7.92308696658404	2.31565180651080
H	-1.53834976310105	8.10516516716624	-1.68364293571523
H	-3.77943548976350	7.09330042300201	-1.84555045945325
C	-5.40874703687535	6.45735893592186	0.19664318489360
C	-5.60218216305644	5.57661092238611	1.27009350539268
C	-6.20202120655480	6.27600439596728	-0.94583900183087
C	-7.07696333796593	5.20453741174101	-1.04511949829562
C	-6.47067747952837	4.50396023516293	1.16769630165506
C	-7.19108662418484	4.26461424798975	-0.01004259799725
H	-6.12036056786086	6.97646870418979	-1.77269934213966
H	-7.67195445003856	5.08493429254270	-1.94647567826796
H	-4.98445617543184	5.67330055864652	2.15796115177325
H	-6.51663409071803	3.78449083855954	1.97976537413623
C	-3.58766066000360	-5.89565655692584	-0.30994327861542
C	-5.06222936233488	-5.90031959585652	-0.33976440235297

C	-2.88408201943704	-5.65810903504389	0.87755037009939
C	-2.84608243816096	-6.13743746104756	-1.47337377723840
C	-1.46025108120135	-6.15084581366970	-1.44801999589162
C	-1.49732031654612	-5.65236930082813	0.89783827299845
C	-0.75210507913597	-5.90388725629272	-0.26322174409312
H	-3.36680015052297	-6.36464556207808	-2.39974889471675
H	-3.43440413650721	-5.43331688069462	1.78743917696570
H	-0.98350439998994	-5.41374189389035	1.82457940030099
C	-5.80211754557239	-6.55495916608782	0.65531284886278
C	-5.76056333256043	-5.28014943601649	-1.38567202246108
C	-7.14526700487074	-5.28174268771157	-1.42431909837245
C	-7.18592197641346	-6.61327318295067	0.59292877308357
C	-7.86789768656699	-5.94664925903157	-0.42763656170591
H	-5.20522914971760	-4.76343428445960	-2.16383117445593
H	-7.67129644225207	-4.78322988372000	-2.23266225114286
H	-5.27865279411645	-7.07276147044276	1.45453338671889
H	-7.75439072824363	-7.17275455843002	1.33047524964567
N	-9.27984626231694	-6.14717746017925	-0.52925532946078
N	-10.07962798808913	-5.22217643857267	-0.76268086790614
C	-9.70396261067160	-3.84661006861292	-0.69038959312670
C	-9.04349424518695	-3.32489362338751	0.42653685011008
C	-10.17532400065948	-2.98408517561532	-1.68283566252042
C	-9.90327720812769	-1.62638775534360	-1.60661683316986
C	-8.82562218869233	-1.96171288989645	0.51992154054683
C	-9.22363147911150	-1.08960274024704	-0.50426688175149
H	-10.74111546374211	-3.39479410365541	-2.51422002658041
H	-10.25571394593275	-0.96320303873465	-2.39203278273374
H	-8.70596760637442	-3.98923693507990	1.21576167740451
H	-8.29403144697971	-1.56460487196540	1.38018926537284
C	-8.88429350063570	0.34161068344878	-0.41768338075365
C	-8.38365173336154	1.02873693644290	-1.53016445718785
C	-8.96577451035988	1.03278808853459	0.79897574345260
C	-8.49531159083599	2.33045969131125	0.91519444384732
C	-7.90096859655946	2.32326023524832	-1.41020162131718
C	-7.91002751491593	2.98813599801994	-0.17629207257563
H	-9.40977810324221	0.54382547500395	1.66190593668093

H	-8.57754822820930	2.84295924088863	1.86956934919391
H	-8.29536811500656	0.51171400762059	-2.48191950281585
H	-7.43353309721219	2.79576777726908	-2.26935155366831
H	-0.91820374424555	-6.39653894772235	-2.35651295923496
H	0.89172842850094	-6.65818088652981	1.77549794379213

126

3CAB-1_cis-trans-trans_c1, Eel(r2SCAN-3c) = -3100.289837790006 a.u.

C	3.12690052345531	-9.47426121969247	-0.19158503704194
C	4.38058100064146	-8.70386741158398	-0.11157516088403
C	2.32072251061597	-9.35046581364687	-1.33210138888045
C	2.57833499213351	-10.13556716740149	0.91938980081150
C	1.24077480588330	-10.49770466813781	0.94624300186444
C	0.98511641434107	-9.71577192635445	-1.30653566725496
C	0.39557177741155	-10.21732781693234	-0.13829512973568
H	3.19805625255603	-10.32715643550809	1.79112340967510
H	2.70642755702217	-8.83921265742458	-2.20887252054672
H	0.35912900628467	-9.48230633453343	-2.16284999773498
C	5.14598192778600	-8.39905372377521	-1.24831798421851
C	4.69311776237821	-8.03920031039419	1.08973588811143
C	5.61924256707164	-7.01707961553962	1.12555308443243
C	6.06720220516776	-7.36474286455665	-1.22494672773372
C	6.26720265936904	-6.61997899945611	-0.05527438885499
H	4.11150972378292	-8.25303579744218	1.98170658554981
H	5.77636254004943	-6.44416913599684	2.03272781286439
H	4.97206198033623	-8.93821156738218	-2.17522251443782
H	6.59788321709694	-7.06932743059761	-2.12560155233697
N	6.93238011662311	-5.38759896353042	-0.20783574870442
N	7.08846004424499	-4.74045875260524	0.86214187732803
C	7.39262248178062	-3.37632728561343	0.70572734418642
C	7.30662336569044	-2.67755201874307	-0.50969476691557
C	7.58586986258817	-2.66029923578655	1.89420805886185
C	7.59753469776506	-1.27648477200603	1.88420547227476
C	7.31138292028145	-1.29564608019675	-0.50911508043577
C	7.40053047725020	-0.56609004084648	0.69040161532212
H	7.67094686467333	-3.21484390949869	2.82430399470587

H	7.72325935090136	-0.73333030055740	2.81652267009411
H	7.14909204193593	-3.23246919565689	-1.42822994532800
H	7.14736054186855	-0.76307419051829	-1.44203200399503
C	7.12276391497241	0.88062981453793	0.70030705632608
C	6.33468264360281	1.42340794516231	1.72497426956098
C	7.51095705323040	1.73148563332572	-0.34434581351876
C	7.08564990154128	3.05127260647474	-0.38714067921521
C	5.90468684885603	2.73834942974091	1.67780516783180
C	6.24737111831698	3.57549230513001	0.60753049478287
H	8.16126728920144	1.35452612736868	-1.12921144319847
H	7.41386182096690	3.68991055622542	-1.20268746030493
H	5.98684305617646	0.78034618675327	2.52810706473629
H	5.22889305821020	3.10130997045414	2.44663249543589
C	4.26342497404269	7.38352358747711	0.33817029544626
C	3.43680899763356	8.60121307623067	0.25885316987226
C	4.59896716856085	6.66215946835440	-0.81463930698721
C	4.68887073856376	6.87840745854585	1.57438071671078
C	5.37591297812608	5.67824488071183	1.65741204328704
C	5.27806923122172	5.45573345717304	-0.73009660470687
C	5.65883547550082	4.92419653159496	0.50962744772138
H	4.49286324271274	7.44676465687038	2.47952656055250
H	5.71004645197528	5.32273312367742	2.62805625379659
H	4.26239910521022	7.01865136691186	-1.78447690554962
H	5.45690310189204	4.88299451395435	-1.63572073386840
C	3.62477150600144	9.55503311205322	-0.75147797719253
C	2.41806922407342	8.82265501256843	1.19789801783996
C	1.58293560314278	9.92244436994513	1.10606856181546
C	2.82696741788266	10.68718223058464	-0.82024907539175
C	1.77142632984843	10.85369874543978	0.07936177012422
H	2.25328792825146	8.09396023906682	1.98672677910805
H	0.78394877725520	10.06461979143301	1.82695758106914
H	4.43486394297192	9.42617343314926	-1.46417866519980
H	3.00175267208928	11.44851925079835	-1.57512515751792
N	1.03377676933937	12.07588170813099	0.02200956627569
N	-0.20690064326685	12.11827233808861	0.12409952327584
C	-1.02712880492026	10.95045464072186	0.05612053539530

C	-0.90412674435796	10.01889415326698	-0.98019022079834
C	-2.09082602354179	10.84789129202050	0.95569787628776
C	-2.96499529674234	9.77425867409064	0.87665416031285
C	-1.81347650323457	8.98056813339780	-1.08208099124120
C	-2.84436779986899	8.82045150329531	-0.14387752033269
H	-2.21196788610918	11.61160874859153	1.71864308909498
H	-3.78132246225276	9.69410090325167	1.58939557686024
H	-0.09769220346850	10.11284079997131	-1.70071039917578
H	-1.70022473700745	8.25033748984380	-1.87852034270793
C	-3.75330238880706	7.66376362489353	-0.23546390655441
C	-4.13528119435160	6.95252315196114	0.90912628054953
C	-4.21466142190848	7.20464167959960	-1.47673273589666
C	-4.98190992389048	6.05518627597831	-1.57297743415407
C	-4.89493163487959	5.79610268752708	0.81124031802329
C	-5.31297583396904	5.30729493779754	-0.43405274637871
H	-3.98203543580034	7.76978154895193	-2.37519812998072
H	-5.34126337672154	5.73566735832075	-2.54711288127993
H	-3.77352481752902	7.27304062068409	1.88245710548063
H	-5.11047726752931	5.22533545630633	1.71009520872596
C	-3.74642705012140	-9.24005622330750	0.05957831142867
C	-4.94457054836684	-8.38466276940055	-0.00657477463189
C	-2.93414883182298	-9.19106645638400	1.20144741985741
C	-3.24402528996346	-9.91867358159403	-1.06273816772481
C	-1.93446045963160	-10.37125080908721	-1.09663751799112
C	-1.62677459134312	-9.64669216708869	1.16879441144499
C	-1.07253316137144	-10.16786795631259	-0.00811612085340
H	-3.87507941170525	-10.05265458455257	-1.93709845025402
H	-3.28429518484462	-8.66943525581211	2.08698266851882
H	-0.98660925220905	-9.47117900249652	2.02846181337770
C	-5.68766563440658	-8.04713438236736	1.13574308485279
C	-5.21099864791455	-7.68074263380321	-1.19656466409611
C	-6.06619587821322	-6.59812049298023	-1.21499632255575
C	-6.53691933349040	-6.95272078227409	1.13001598688537
C	-6.68639099331714	-6.17760067559866	-0.02740407123472
H	-4.64483393931692	-7.91867688537531	-2.09235684742864
H	-6.18416130698085	-6.00119874120964	-2.11258006300655

H	-5.55076304485131	-8.61179059616521	2.05362743951646
H	-7.04659213082813	-6.63683891158373	2.03584032143110
N	-7.26771152319262	-4.90607643324401	0.14498197489706
N	-7.38346396975072	-4.23473134250561	-0.91513981335921
C	-7.59613451688766	-2.85580609745438	-0.73838889327670
C	-7.46109066941366	-2.18169442524019	0.48647064622252
C	-7.74442530188373	-2.11166973142116	-1.91600840321536
C	-7.66419673764223	-0.73042636553052	-1.88647118956388
C	-7.37411313282438	-0.80263700311680	0.50536806754907
C	-7.41747376097450	-0.05178407787169	-0.68346766264821
H	-7.86837570033319	-2.64604613352153	-2.85351041941645
H	-7.75578250108034	-0.16681873171778	-2.81059582993491
H	-7.33853803012721	-2.75884881390817	1.39659952233899
H	-7.17284296951576	-0.29546736579412	1.44512866364195
C	-7.04392298357849	1.37322801149542	-0.67429259318537
C	-6.22369678793386	1.87626298548227	-1.69399818476347
C	-7.37205458007463	2.23363778040702	0.38305047915888
C	-6.85964705201265	3.52150443285792	0.44232412434107
C	-5.70685228734691	3.15880802854828	-1.63036879337790
C	-5.99059162725059	4.00231209242609	-0.54785712028193
H	-8.04419449475743	1.89010059224800	1.16486310255836
H	-7.14263294805409	4.16931397109071	1.26751747722389
H	-5.92138649607813	1.22248821794930	-2.50684706067257
H	-5.00999803773907	3.48631873708431	-2.39629066494983
H	-1.55769646268146	-10.84822289435464	-1.99763843527390
H	0.83153813163222	-10.96276549300511	1.83927269226116

126

3CAB-1_cis-trans-trans_c2, Eel(r2SCAN-3c) = -3100.289294768595 a.u.

C	3.56142924393787	-9.33166248895863	-0.21430762039761
C	4.77206545872293	-8.49747835395241	-0.11199991706056
C	2.79727143970472	-9.29157159460580	-1.38939273473707
C	3.00029275991257	-9.97713308259926	0.89990104726706
C	1.68023685657649	-10.39859841098360	0.89109383935630
C	1.47988768020251	-9.71971654796275	-1.40060077467754
C	0.86763215902991	-10.19981050156998	-0.23529432007857

H	3.59283072691404	-10.10707165109517	1.80131358886278
H	3.19345176096623	-8.79693750802094	-2.27099301526806
H	0.87795440402831	-9.55152744349303	-2.28904832299781
C	5.56308628044347	-8.18244117349765	-1.22849700222874
C	5.00825300807186	-7.78904482320222	1.08173320896947
C	5.88459044066676	-6.72435490495374	1.12476221949662
C	6.43264181121912	-7.10444488724112	-1.19910250666267
C	6.55695592629425	-6.32508718757144	-0.04147563982249
H	4.40441890665338	-8.00719969057146	1.95760512979713
H	5.98137174892843	-6.12335429482870	2.02216988848731
H	5.44962832706695	-8.75102790296720	-2.14712309734726
H	6.98095187083738	-6.80537687673639	-2.08792985470357
N	7.17079275142316	-5.06690280981153	-0.19684798031192
N	7.28009352802983	-4.40206551470997	0.86811366446153
C	7.52461231111828	-3.02729547002547	0.70201515448388
C	7.41234427056438	-2.34360589762311	-0.51977982082577
C	7.68074955565335	-2.29294734192178	1.88482126256338
C	7.63246804693446	-0.91013339866779	1.86234155147484
C	7.35589612919402	-0.96291271108566	-0.53153166941983
C	7.40908486880717	-0.21940532414699	0.66153442288103
H	7.78619625608662	-2.83501720507277	2.82018111069890
H	7.73125331759188	-0.35393457703006	2.79018807215712
H	7.28254406831916	-2.91289375421943	-1.43387216452575
H	7.17125307182748	-0.44670829764795	-1.46974344075715
C	7.06931047592627	1.21406123095791	0.65689286204214
C	6.26038344713798	1.73434310671953	1.67703730785713
C	7.41891016613391	2.06886047993983	-0.39819086354452
C	6.93602616122142	3.36801975786109	-0.45598866956045
C	5.77377392284718	3.02889559566942	1.61550895478611
C	6.07715244229332	3.86685713326986	0.53411112432740
H	8.08411689862088	1.71159485650369	-1.17974716337681
H	7.23425830397596	4.01065922606400	-1.27986467421789
H	5.94200024948240	1.08648289765384	2.48846668945485
H	5.08446539592334	3.37176421555908	2.38159619464969
C	3.92526829975295	7.57901028425070	0.21706110700539
C	3.04626477498000	8.75812455692781	0.12111592296917

C	4.28430885664861	6.85283268450875	-0.92575892576876
C	4.38038225654265	7.11392793089199	1.45849524742951
C	5.11953270732019	5.94619038577517	1.55673008981660
C	5.01687826296387	5.67947179775259	-0.82606740552623
C	5.42869765732855	5.18643451513883	0.41958879455247
H	4.16599932055096	7.68841253113682	2.35554570107310
H	5.47461956123921	5.62153068142351	2.53077126647759
H	3.92558547908101	7.17685626635098	-1.89899813849151
H	5.21516334795747	5.09968006349530	-1.72311832454102
C	3.17928121844349	9.69259612212156	-0.91574847134425
C	2.03075664381405	8.96013793837521	1.06830089516992
C	1.14581848591039	10.01862180349488	0.95867969846239
C	2.32973203626501	10.78482771407965	-1.00441733829755
C	1.27787697504247	10.92776612878423	-0.09636694475953
H	1.90813709709879	8.24657392023253	1.87833412470427
H	0.34989208692899	10.14404584387173	1.68611190691982
H	3.98597505005620	9.58174785890740	-1.63525203189826
H	2.46007828998720	11.53217953475033	-1.78188815833002
N	0.48244837504887	12.11084964689892	-0.18449312442371
N	-0.75772452432389	12.10070997908452	-0.06643903062292
C	-1.52700124684276	10.89731748096292	-0.07794888485426
C	-1.36414400073395	9.91616700371163	-1.06201481804045
C	-2.58850093628991	10.80005828413189	0.82508339172321
C	-3.41760585152941	9.68887701413218	0.80524392175321
C	-2.22748041284771	8.83554795040643	-1.10441025181574
C	-3.25324534512515	8.68381869733394	-0.15864717288007
H	-2.74339416456758	11.59968346167115	1.54382139753906
H	-4.23284660231814	9.61654390184266	1.51993872226300
H	-0.56165301801830	10.00331538083731	-1.78766959438280
H	-2.07958582376943	8.06720878941989	-1.85805546420924
C	-4.10950037114889	7.48447272378042	-0.18142682281804
C	-4.48858038959204	6.84496862872867	1.00602163565844
C	-4.52061602739038	6.91237861572364	-1.39331116098054
C	-5.23257569889437	5.72426517777330	-1.41740274279048
C	-5.19649325045169	5.65247056783174	0.98127127931435
C	-5.55990430796125	5.05179718748487	-0.23154802889094

H	-4.29252870977598	7.41788549829443	-2.32766170738102
H	-5.55181204749533	5.31370671076568	-2.37126959773777
H	-4.16432275783866	7.25277600126837	1.95961988604394
H	-5.41145287256864	5.14245210077713	1.91615961560941
C	-3.31822945326536	-9.39585938052131	-0.23238401528984
C	-4.55089691488996	-8.60328706301600	-0.38974714427813
C	-2.54210826108460	-9.22837676192180	0.92378163927072
C	-2.75203761290748	-10.12964817072928	-1.28808920871410
C	-1.42231298180982	-10.52025490312076	-1.25526384910612
C	-1.21727394845814	-9.62848484288420	0.96117538412739
C	-0.60503288002619	-10.20557879638169	-0.15919448350217
H	-3.34876004304244	-10.35722550888208	-2.16699161886563
H	-2.93753099409324	-8.66133249591089	1.76056343796735
H	-0.61194202869565	-9.36690474747326	1.82422387657178
C	-5.30047214391646	-8.16852111910471	0.72301364318648
C	-4.86898968173864	-8.05998989047631	-1.64301041887550
C	-5.77874599999193	-7.02229073115430	-1.75676272143717
C	-6.18885940380341	-7.11619478848331	0.62394118350960
C	-6.37747463303523	-6.48022139436036	-0.61468509812467
H	-4.31423476859240	-8.37187371668208	-2.52234613430850
H	-5.95303721944569	-6.53528708719627	-2.71159953310099
H	-5.13840126935813	-8.63460125941854	1.69099227260276
H	-6.69859277512610	-6.73412097127540	1.50181016245943
N	-6.96387692795829	-5.21459693686697	-0.80455747132015
N	-7.18133226495706	-4.57338006415282	0.25867404872894
C	-7.46772698343433	-3.20623967515533	0.06513460979702
C	-7.46813136900685	-2.42578097355942	1.22649267657091
C	-7.56496540163422	-2.57467885485377	-1.18593649433727
C	-7.54283252913951	-1.19667444290961	-1.26000774207235
C	-7.43975182765665	-1.04253882447020	1.14221878749318
C	-7.41572965235380	-0.40197382726152	-0.10397143589365
H	-7.60337246529513	-3.18028018952096	-2.08467375744267
H	-7.59591375279310	-0.71383001438266	-2.23209120184983
H	-7.40015031061455	-2.92878556208410	2.18663091802201
H	-7.33855505334711	-0.45472314047164	2.04994570400185
C	-7.10666629858491	1.03501289960660	-0.20265834939301

C	-6.25736575633341	1.48689149396032	-1.22284391585230
C	-7.52521123369725	1.96715404645773	0.75770757877205
C	-7.06981501123003	3.27737756471327	0.72901647673398
C	-5.80132274047223	2.79349315046168	-1.24938102386712
C	-6.17462002567642	3.71167262284105	-0.25911163207325
H	-8.22260665971239	1.66189339101088	1.53316921290601
H	-7.41959519612148	3.97989467223211	1.48067112273402
H	-5.88504496300104	0.78158214325790	-1.95985286084391
H	-5.08281919574564	3.08825462018021	-2.00869354909582
H	-0.99650586466628	-11.03904552212351	-2.11024155512120
H	1.25566462394544	-10.84538618647257	1.78634197405747

88

3LAB-0-Ph2, Eel(r2SCAN-3c) = -2177.519920116821 a.u.

C	-0.90377804772132	0.04244131621986	1.54381044719205
C	-0.77294722585818	0.78345191475216	2.72336185268758
C	-2.06958609966069	-0.70941805568197	1.32673725173947
C	-3.07036197516090	-0.71090432643026	2.27787379850491
C	-1.78340444227151	0.78012064156563	3.67014572017519
C	-2.95200627066175	0.03200402573884	3.46802828721159
H	-2.17336804965505	-1.27224489526866	0.40574867951920
H	-3.98320710352594	-1.26960041707323	2.09054482759794
H	0.14089136537276	1.34922770711604	2.87888338984650
H	-1.65469732096469	1.33573117696983	4.59435687388122
N	0.18456138147926	0.12290090626291	0.65312586490397
N	0.04731842543750	-0.53706150881394	-0.41005662550928
H	0.07640784992389	-1.73886268951309	-2.64889973416757
C	1.13628878138774	-0.45877331830514	-1.30005118325196
C	0.99074005656354	-1.17375468186912	-2.49386759421703
C	2.31592704229611	0.26709916333558	-1.06963601507122
C	3.31730853591881	0.26587726308230	-2.02020222505648
C	1.99793187680164	-1.16588190993591	-3.44403167701448
C	3.18226794955048	-0.44738316799590	-3.22660613683167
H	2.42023408742049	0.83043883548550	-0.14901027531208
H	4.21471790546634	0.85431350330921	-1.85010895764909
C	6.29952442067774	-0.42293896211712	-6.16097675202559

C	6.61159978796209	-0.42467341689671	-4.79727221328383
C	4.95378554309869	-0.42813289462526	-6.56091653631483
C	3.95580972206297	-0.43756651831401	-5.60670048500059
C	5.60467985469548	-0.42936513825551	-3.84654096443361
C	4.25645092831443	-0.43781723014391	-4.23133042824411
H	4.71532660051498	-0.41353552962336	-7.61860266935977
H	2.91636361917663	-0.41029292488083	-5.92194216499717
H	7.65813599514091	-0.43254216684980	-4.50728493132443
H	5.86251623750247	-0.46166110863355	-2.79204048380560
N	7.40420271071752	-0.41561478612771	-7.03627506946645
N	7.10812135959523	-0.40366808123081	-8.25938313348552
H	6.84779021206224	-0.37779247536964	-10.78690188910639
C	8.21055856580496	-0.39676896457252	-9.13665408714422
C	7.89505913381270	-0.38914123129676	-10.49954207378284
C	9.55712512708638	-0.39698962870343	-8.73981181760456
C	10.55278555053071	-0.38780852460967	-9.69711955216713
C	8.90033759569470	-0.38165765672961	-11.45262489375483
C	10.24842175395916	-0.37995793702694	-11.07082433401244
H	9.79831445133575	-0.41559823178894	-7.68277789429216
H	11.59357789098680	-0.41804660163187	-9.38618632993644
H	8.64213999204021	-0.34494048499942	-12.50703312576605
C	-8.43915453582090	0.82795288713424	8.89715629577448
C	-8.74050865901591	1.99060154676060	9.61440040754085
C	-9.17193530172747	-0.34400374373313	9.14272292281560
C	-10.18093128356128	-0.33540517857802	10.08597363120265
C	-9.75308986277364	1.98940092627640	10.55975574373853
C	-10.49372916712045	0.82746175636340	10.81372857329929
H	-8.92758540174532	-1.24676926362675	8.59394371667472
H	-10.72589485742754	-1.25222533880928	10.29407354655047
H	-8.17024688337496	2.89026780470265	9.40254246732213
H	-9.99744009472639	2.90655094122155	11.08788234616821
N	-7.39192931237750	0.94671972053363	7.96215768226282
N	-7.12634661606651	-0.10702043389374	7.32741234398435
H	-6.34926180347133	-2.04574651404536	5.87909937716988
C	-6.07890133589725	0.01439016177020	6.39227781302867
C	-5.77912016098939	-1.14544898176485	5.66982258478450

C	-5.34538680499800	1.18680443266397	6.15056395778775
C	-4.33752847291360	1.18179895953054	5.20670984703156
C	-4.76987344355392	-1.14083077999821	4.72160612795666
C	-4.02733608360398	0.02193822922086	4.47152510474400
H	1.88337522922923	-1.74993869278201	-4.35242843705373
H	-4.52843352691057	-2.05689488042486	4.19055102569105
H	-5.58890029265791	2.08747628533963	6.70309297754827
H	-3.79423441100734	2.10022018137772	5.00198509496753
C	-11.57409088357639	0.82108841546101	11.81721375653667
C	11.32267035541012	-0.36999635763511	-12.08082997158180
C	-12.75690701735341	0.10329103590472	11.59333893634003
C	-11.44348050065284	1.53363299046570	13.01705106590293
C	-12.46203303491511	1.52698032456314	13.96166139021770
C	-13.77599712966451	0.10114995744673	12.53744211864751
C	-13.63301847488352	0.81185070266836	13.72594695826459
H	-10.52141284175331	2.07041273973734	13.22216477224251
H	-12.33732789246241	2.07548249008448	14.89094116528724
H	-12.88930077013978	-0.43050385585143	10.65637351125744
H	-14.69034899112848	-0.45125401459522	12.34054056913568
H	-14.42936064132835	0.80815058409696	14.46434449079898
C	12.49048921657977	0.37813066268299	-11.87857745625268
C	11.20010547353473	-1.10740554817717	-13.26639024135059
C	12.21306631379710	-1.09756719174207	-14.21697489070702
C	13.50058563910994	0.38993417147210	-12.83222685345370
C	13.36714381355455	-0.34836018048305	-14.00490963337636
H	10.31596309266975	-1.71807761547927	-13.42696830226475
H	12.10504037011311	-1.68521771665664	-15.12415218092195
H	12.59097094533850	0.98014898545545	-10.97977066971633
H	14.39305621686037	0.98558896904944	-12.66296212434864
H	14.15807404849080	-0.34021056223086	-14.74906630151788

148

3LAB-1-Ph2, Eel(r2SCAN-3c) = -3563.541574048719 a.u.

C	-18.60864062192312	16.93536754585076	-0.23126843975271
C	-17.49275116009293	15.97642987497248	-0.16752043309635
C	-18.86599164902196	17.81691746140694	0.82764818762697

C	-19.44672213610497	16.99648861857619	-1.35313376957397
C	-20.49477339920639	17.90155304447312	-1.41390083817602
C	-19.91605863138357	18.71957598902605	0.76728894853722
C	-20.75234684352418	18.78183480792499	-0.35497643746646
H	-19.24630065461470	16.35483743460821	-2.20659991909694
H	-18.25996500935612	17.76303439744982	1.72773770828571
H	-20.11823666854953	19.36171696764434	1.62008781847308
C	-16.27788080775842	16.32269552070041	0.43963306826450
C	-17.61931564552132	14.68634576621647	-0.71562036376603
C	-16.58146762629686	13.77664892200539	-0.66232403862119
C	-15.23118449435618	15.41733751297637	0.49607053733271
C	-15.36982946237776	14.13764262730194	-0.05194164131404
H	-18.56524264015259	14.39133964656459	-1.16173857834336
H	-16.69034824602377	12.77995292332423	-1.07536127521812
H	-16.14546767031941	17.32308499478162	0.84143598372145
H	-14.28262448378103	15.68270996629883	0.95358863994954
N	-14.24104306686946	13.30257168041714	0.06392299001522
N	-14.38260892450481	12.15026018398576	-0.42224706003369
C	-13.25272838058770	11.31619869752905	-0.30683584719205
C	-12.03739235751114	11.68246712735207	0.29285487022473
C	-13.39384844896312	10.03246174597442	-0.84451765837975
C	-12.34641817606897	9.12792220945930	-0.78747544819315
C	-10.99868109979475	10.77378984160339	0.34681387098341
C	-11.12810021015150	9.47937516236492	-0.19028117205042
H	-14.34498108626156	9.76307923411477	-1.29427425960493
H	-12.48122777456202	8.12447171198509	-1.18076071726135
H	-11.92626546900003	12.68254875241661	0.69699901853049
H	-10.05010455724906	11.07331586978874	0.78419499786521
C	-10.01127869186387	8.52157340741671	-0.12560911723515
C	-9.75481919269682	7.63687961495275	-1.18207212345658
C	-9.17110642676290	8.46499151351671	0.99491558441392
C	-8.12311853631151	7.56039498429890	1.05765995679800
C	-8.70247216471687	6.73721247828792	-1.12182840107793
C	-7.86490046366099	6.67828621893275	0.00001218370924
H	-9.35709041069515	9.12485514971572	1.83765253283890
H	-7.47846598599523	7.55047795436456	1.93216169746470

H	-10.37669298396158	7.67165433776538	-2.07219877187544
H	-8.53762867472641	6.05253977964490	-1.94917421125360
C	-4.59815568575297	3.87868754217865	0.19132756971383
C	-3.47912861837061	2.92383038414911	0.25773027133160
C	-5.35950209651971	4.18104253607930	1.32882762344029
C	-4.93537617213587	4.51589654445693	-1.01085421099962
C	-5.98598782920443	5.41753957008605	-1.07193811831054
C	-6.41140427595262	5.08098135333933	1.26685226200554
C	-6.74712257444662	5.71959021432598	0.06533275540515
H	-4.33717350674041	4.33387249389632	-1.89923905886844
H	-6.19614672304704	5.92870951641148	-2.00730052490399
H	-5.14946439050713	3.67004152016243	2.26424702480839
H	-7.01064369096551	5.26270033516337	2.15468474390511
C	-2.65273212321332	2.85919804971809	1.38788742536521
C	-3.21166298032477	2.05159461469605	-0.81389553369968
C	-2.16572623239223	1.15096481078347	-0.76478432788748
C	-1.60050119618086	1.96029471135118	1.44494628002687
C	-1.34530104967994	1.09816866416117	0.37317849839983
H	-3.86221119283554	2.06708687267210	-1.68417002574622
H	-1.97304568951558	0.47293858420256	-1.58880219978169
H	-2.81990196190735	3.54408389106700	2.21417022006406
H	-0.94800992341100	1.91316958437079	2.31183742268490
N	-0.25028025908008	0.22773269337416	0.54262048568594
N	-0.03687861651464	-0.54886395748971	-0.42453450136023
C	1.05887694187069	-1.41846905048298	-0.25516789275537
C	1.88393733246646	-1.46516401074698	0.87971237364821
C	1.31042877214881	-2.28557103345153	-1.32376007463734
C	2.36317385968361	-3.18388792732679	-1.26630198438210
C	2.93061229822366	-2.36488747074236	0.92903943319213
C	3.19415438871379	-3.24246653965796	-0.13918911395237
H	0.65464536336872	-2.24288932660695	-2.18839780039791
H	2.52734153638955	-3.87278325692999	-2.08985065640875
H	1.69440729597275	-0.78295078505574	1.70099344760145
H	3.58493401478055	-2.37529635433613	1.79654787569984
C	4.31428835265381	-4.19601848833174	-0.07229652530218
C	5.07110872154097	-4.50413209815153	-1.21127335253003

C	4.65743291586309	-4.82564369980354	1.13222586608641
C	5.70975467159959	-5.72518729534739	1.19421227307761
C	6.12483720527970	-5.40190302229292	-1.14829054177850
C	6.46675138605284	-6.03248386622226	0.05564602268873
H	4.06271764381080	-4.63918044221479	2.02202374420023
H	5.92502418185158	-6.23040078373070	2.13166166396291
H	4.85621574713811	-3.99912607771503	-2.14884163568807
H	6.72075218088145	-5.58815390881391	-2.03743720873936
C	19.00314241475081	-16.49213458082425	-0.06355556669903
C	17.88500897119142	-15.53602622189680	-0.13079127050127
C	19.26475677498617	-17.37447703044716	-1.12075181164240
C	19.84040708746858	-16.54815775498314	1.05918085308005
C	20.89463981130003	-17.44598415872225	1.12048965458573
C	20.31735317848140	-18.27397672777407	-1.05734289953588
C	21.15524428370601	-18.32844384049317	0.06413420532342
H	19.68795238748727	-15.84826471396707	1.87600705253350
H	18.60669424991289	-17.38342472826791	-1.98515256419348
H	20.47101303453409	-18.97496338268756	-1.87312891011252
C	17.13255530597026	-15.22572846315660	1.01738321583739
C	17.54134220334003	-14.91164019998591	-1.33761365297801
C	16.48914480083350	-14.01271224690552	-1.39517810384757
C	16.08094658119169	-14.33171207742084	0.96885599604383
C	15.74868369488546	-13.71294982675610	-0.24672329208011
H	18.12735016079656	-15.11101769052194	-2.23013241982003
H	16.22485332443112	-13.51554885994237	-2.32388016938391
H	17.36576280381515	-15.72518796908565	1.95385895702735
H	15.49719631097504	-14.10695712029358	1.85462933699898
N	14.70249325310943	-12.78606890161210	-0.42412823735514
N	14.03985010003653	-12.53038171458958	0.61491265215350
C	12.99359611425058	-11.60333640248165	0.43673963321948
C	12.66134455103032	-10.98567363796801	-0.77934717826450
C	12.25318241554018	-11.30253832862577	1.58489540569840
C	11.20116083614540	-10.40343693384345	1.52662387897565
C	11.60973014009469	-10.09163928649437	-0.82873849247595
C	10.85753655223892	-9.78022310385544	0.31920925895295
H	12.51739348746658	-11.79897448303301	2.51399674972549

H	10.61527050960127	-10.20336510801482	2.41907724191825
H	13.24509088694414	-11.21132805244210	-1.66488840935643
H	11.37668448785582	-9.59309620415769	-1.76575828079475
C	9.73950270970254	-8.82422401532416	0.25126409987704
C	9.47701785954495	-7.94191458697147	1.30843191259790
C	8.90282816855345	-8.76801797164345	-0.87205840373829
C	7.84887813127223	-7.87035861976302	-0.93423037658543
C	8.42440474897666	-7.04290055735937	1.24487714111294
C	7.58699427771047	-6.98803379098695	0.12251037144667
H	9.05570041411438	-9.46796444034837	-1.68870377643471
H	7.19100622484422	-7.87920320601671	-1.79882225933808
H	10.13473711024156	-7.93277019134722	2.17303714743702
H	8.27153787663774	-6.34186505479285	2.06071145826082
H	21.55310927623679	-17.43848720001644	1.98468974563671
H	-21.10200060840607	17.95604481138892	-2.31323460845772
C	22.27681286184409	-19.28571464693835	0.13072491469577
C	-21.86964032962186	19.74420979276747	-0.41948996643326
C	-21.72986101852523	21.04117818110306	0.09167971159889
C	-23.09558533187855	19.38296553550146	-0.99353025195562
C	-24.14705666712687	20.28932509338141	-1.05354887105538
C	-22.78176936588099	21.94691141409217	0.02992327224275
C	-23.99506642019870	21.57529344317622	-0.54256437209598
H	-23.23268062409940	18.37222989304107	-1.36809322401801
H	-25.09343411309577	19.98693619691616	-1.49288809264809
H	-20.77700725353863	21.34913769984161	0.51347476607072
H	-22.65037953449183	22.95142424206973	0.42207317696114
H	-24.81706283743013	22.28345047643160	-0.59017054039721
C	23.05035682517563	-19.56445589826000	-1.00367069343489
C	22.59367298871066	-19.93752310427545	1.32975505424784
C	23.64927685408098	-20.83896709126663	1.39144048903251
C	24.10648977291293	-20.46515298887804	-0.94025431916116
C	24.41065550876232	-21.10668133843969	0.25717994457992
H	21.98635905833487	-19.75684272830610	2.21247938970404
H	23.87261584503269	-21.34247565530697	2.32775981222466
H	22.83856964116136	-19.04619569815717	-1.93492858334137
H	24.70139050106516	-20.65993386230528	-1.82808415058325

H 25.23591864669503 -21.81094015094234 0.30610118105819

22

biphenyl, Eel(r2SCAN-3c) = -463.186306528459 a.u.

C -0.73904920554449 0.00000137296204 -0.00007648645270
C 0.73904511428084 -0.00004858298858 0.00006086947771
C -1.45617368224788 1.20312420526602 -0.00258757079784
C -1.45625484675728 -1.20307330133530 0.00230011535207
C -2.84602685473718 -1.20250388169243 0.00269175969044
C -2.84594558206207 1.20264757390642 -0.00324034316009
C -3.54714064117589 0.00009528098082 -0.00034121812148
H -0.91635233881747 -2.14559014144921 0.03336330564269
H -3.38448406180873 -2.14596911463928 0.01506788176688
H -0.91620255465680 2.14560505743796 -0.03355027528998
H -3.38433764226120 2.14614865115581 -0.01571834462119
H -4.63321113305204 0.00013267766190 -0.00044245950020
C 1.45617508323423 0.91995964769768 0.77536060718417
C 1.45625575168514 -0.92010611957530 -0.77510568393370
C 2.84602765386844 -0.91942287218085 -0.77503657140844
C 2.84594688940550 0.91918037489927 0.77554921529053
C 3.54715339614085 -0.00014601305642 0.00032203720005
H 0.91634892969937 -1.62218404789969 -1.40469486403650
H 3.38447375971430 -1.63423208989462 -1.39093986389922
H 0.91620006785727 1.62207436607306 1.40485031386766
H 3.38432829114887 1.63395193330532 1.39155279682090
H 4.63322360608622 -0.00018197663460 0.00042277892823

127

3CAB-1_cis-cis-trans_1H, Eel(r2SCAN-3c) = -3100.684069387063 a.u.

C 3.58797789693198 -9.19944487508607 -0.28230188083298
C 4.82165931308014 -8.40363283998242 -0.19695189927067
C 2.80816679879172 -9.14101982385565 -1.44752568711080
C 3.02922544546614 -9.83214735391164 0.84170821233688
C 1.70290333811469 -10.22889095566743 0.85020460789010
C 1.48277123146395 -9.54211434791974 -1.43975081960828
C 0.87946248144353 -10.01318531710194 -0.26576749844277

H	3.63257395073195	-9.98169807545368	1.73257034292703
H	3.20258310315258	-8.65865896706144	-2.33654612351068
H	0.87194238120918	-9.36730255166968	-2.32068775009606
C	5.60068133117124	-8.09802816655230	-1.32685176672976
C	5.11790188381093	-7.73478577103702	1.00911382400800
C	6.03590703690916	-6.70978177530980	1.05422788752494
C	6.51844689471532	-7.06324142714387	-1.29457509373938
C	6.70050642940522	-6.31523005098223	-0.12130262772408
H	4.53396610354860	-7.95569195378228	1.89722384268633
H	6.18689064114221	-6.14095309834156	1.96489631160495
H	5.44570962366617	-8.64431519775019	-2.25239766389781
H	7.06538370506648	-6.77300047330410	-2.18692276913727
N	7.37245376571798	-5.09390275179070	-0.25963970320882
N	7.49725254195287	-4.43038160751046	0.80425463480273
C	7.76375442157497	-3.06418051454032	0.65940561421032
C	7.68663965530796	-2.36102263502964	-0.55629779172731
C	7.90592260312095	-2.35107845017392	1.85982592957324
C	7.85775925473487	-0.97079361756879	1.85958341315709
C	7.65183236006177	-0.98061346718693	-0.54753999125815
C	7.67139648715045	-0.26021579053268	0.66197788964973
H	7.99313071536014	-2.91015177529961	2.78671498020336
H	7.93710787310215	-0.43004476455313	2.79820495078660
H	7.57820327833088	-2.91484612401862	-1.48260895684360
H	7.50884009724545	-0.44608151079256	-1.48283121679197
C	7.33043447997708	1.16800315022756	0.67756157324484
C	6.47705370942689	1.65608356978334	1.67978037931972
C	7.72407660305378	2.05817323278659	-0.33488581043432
C	7.24537898093889	3.35689895093571	-0.36939587534992
C	5.98607806364450	2.94573971513804	1.63502735728694
C	6.33603564733615	3.82493600855815	0.59620059819078
H	8.42619959913221	1.72824770691667	-1.09540170239706
H	7.59333429802223	4.02186532492486	-1.15403872270052
H	6.12815808807681	0.98318800807027	2.45702971056608
H	5.25875476508687	3.24827650727501	2.38100847717296
C	4.14765361832607	7.51076981876744	0.26678200912262
C	3.23696748964337	8.64116430464872	0.13092814248373

C	4.79104499333148	6.95343685795082	-0.85216843454255
C	4.35740284431725	6.89802041966258	1.51584328507481
C	5.12621108229755	5.75879323170667	1.62909461374365
C	5.52605664093870	5.78756694041508	-0.74146432048417
C	5.68892398577693	5.13907023023291	0.49662435479495
H	3.94146571672371	7.34853825576475	2.41242793996848
H	5.30147332143482	5.34255534052596	2.61543477193324
H	4.63832449822046	7.39268194706619	-1.83395586186580
H	5.92621592875581	5.33591779183808	-1.64333154040378
C	3.46996264632153	9.69115309553708	-0.77947794876174
C	2.06156371394398	8.69081778025104	0.91592746187794
C	1.12151900161386	9.67577055802734	0.74551740146936
C	2.58021141227551	10.73654734969757	-0.89514807709955
C	1.36254767366276	10.72772329250852	-0.17443698676241
H	1.87191741760853	7.89997363022461	1.63533855975071
H	0.22075337113404	9.66370188479896	1.34876144111780
H	4.38967081931926	9.70532475637943	-1.35645589650472
H	2.78118361676088	11.57440672430751	-1.55559029677301
N	0.56545053459286	11.82558269730023	-0.34447746685320
N	-0.70525557109815	11.76810245887988	-0.08137632179411
C	-1.60179016812471	10.67228044638754	-0.10271114108698
C	-1.40182879206319	9.60663941636037	-0.98752185234596
C	-2.73538031311778	10.71027523527517	0.71442929480363
C	-3.64243338636506	9.66274494015948	0.67854632613027
C	-2.32741183378908	8.58609825420996	-1.03154936811109
C	-3.45601278404271	8.57503842332603	-0.18897670087955
H	-2.89647441723263	11.55056395322482	1.38517999024420
H	-4.52218219541600	9.69897692666040	1.31383193887361
H	-0.53538314351668	9.58533116473561	-1.63951803183382
H	-2.16389968913055	7.75593938187542	-1.71161584116249
C	-4.36929817316294	7.42743322341344	-0.22109872810592
C	-4.91277905085082	6.89422508978347	0.95703082595869
C	-4.66068926444551	6.78099296828551	-1.43291195123462
C	-5.40732861704103	5.61899033850132	-1.45661155393986
C	-5.63991185564101	5.71509499697901	0.93350338786578
C	-5.87297018870851	5.02888960172631	-0.26910042381175

H	-4.31750611308559	7.21246175493030	-2.36917471561457
H	-5.64018744437478	5.15867126077752	-2.41173213622987
H	-4.69289424006973	7.36358176318645	1.91215117910652
H	-5.97110218118157	5.28018009014793	1.87164937346958
C	-3.31709452850178	-9.25119779627979	-0.18928626535793
C	-4.57927554084003	-8.50100776447139	-0.31738062986280
C	-2.51737917844366	-9.05508146235005	0.94602697484232
C	-2.76134491168783	-9.98015257597460	-1.25396780013669
C	-1.42459685503549	-10.34673865849243	-1.24858160283353
C	-1.18605511123078	-9.43411261164934	0.95777063035692
C	-0.59152885218383	-10.01499814375728	-0.16998816543107
H	-3.37398149706313	-10.22946871236735	-2.11560122410822
H	-2.90461945267635	-8.49120000795081	1.78858321673869
H	-0.56690326105666	-9.16115477683152	1.80735143463165
C	-5.30659266433857	-8.07719912554790	0.81503248904688
C	-4.96380122298328	-7.99647981766180	-1.56847581206209
C	-5.92130983738177	-7.00204034402413	-1.66797342565843
C	-6.24116637049975	-7.06538350083049	0.73045889721903
C	-6.50223191795117	-6.46495643432664	-0.51340279986720
H	-4.43133853069572	-8.30511954746420	-2.46235998048894
H	-6.15437727110022	-6.54506224808555	-2.62498669588617
H	-5.09399119740518	-8.52062623459359	1.78358925241850
H	-6.73861968957257	-6.69137292293373	1.61877973014659
N	-7.15046198205475	-5.23419146860596	-0.70493946660095
N	-7.38157161793462	-4.57982092933123	0.34575607677062
C	-7.70276533955123	-3.22447487563699	0.12852186313014
C	-7.77730886023427	-2.42838688443524	1.27737052303576
C	-7.75814485959148	-2.61422841437596	-1.13650067015710
C	-7.76076253562491	-1.23886999661285	-1.23164864427268
C	-7.77789556097220	-1.04626303888902	1.17228583101052
C	-7.70361725829771	-0.42544531293810	-0.08247294924689
H	-7.74490115713442	-3.23472840567958	-2.02566862299052
H	-7.78215780007511	-0.77276243846311	-2.21293044318957
H	-7.74489883109400	-2.91465506318649	2.24776934505871
H	-7.73652286590904	-0.44341215865585	2.07487447733152
C	-7.40149113296886	1.00994490531674	-0.19494246080176

C	-6.50869092091832	1.44121586917249	-1.18799163046778
C	-7.86950953049269	1.96526721431380	0.72084960469522
C	-7.42120867238429	3.27659394401694	0.67808132598625
C	-6.06063925528621	2.74759611147626	-1.22955511569278
C	-6.48494184672866	3.69137372400608	-0.28230315089757
H	-8.60106359491158	1.67648121891379	1.47030814473502
H	-7.81466795872194	3.99397581804528	1.39291076117068
H	-6.09910923654681	0.71953601641919	-1.88783739095557
H	-5.30823801549666	3.02030985796762	-1.96316195589648
H	-1.00970109752272	-10.86789118559576	-2.10735050753769
H	1.28312456833212	-10.67446062770419	1.74802116446567
H	-1.13519952995030	12.68753406591150	-0.03621822030872

128

3CAB-1_cis-cis-trans_2H, Eel(r2SCAN-3c) = -3101.033125663281 a.u.

C	3.48806631211178	-9.10412736816706	-0.31131193013559
C	4.70470447177790	-8.28739649301366	-0.21495507951386
C	2.72637837289770	-9.08374464946252	-1.49141899865352
C	2.93362357503624	-9.75303764135636	0.80912232015709
C	1.62342155464714	-10.18935187238323	0.80243559541890
C	1.41941074721480	-9.53285303120551	-1.50388973952686
C	0.80956036941134	-10.01468826788878	-0.33270318392157
H	3.53211802500196	-9.88705739263931	1.70510372001995
H	3.12152387069805	-8.60113590520837	-2.37941426841118
H	0.82682344298195	-9.38972143034014	-2.40210337693994
C	5.48882620572945	-7.97316824272895	-1.33911661282120
C	4.97765976850840	-7.61499015177009	0.99382323091155
C	5.88694714238261	-6.58160616004071	1.04785410818381
C	6.40170811023826	-6.93510297662509	-1.29510877746799
C	6.56566257990831	-6.18816273655592	-0.11877019644661
H	4.38950385032887	-7.84211624272233	1.87766567358926
H	6.02540892091229	-6.01165019965291	1.95976099930584
H	5.35262475584895	-8.52269724563605	-2.26568871308766
H	6.96173871917260	-6.64506039684818	-2.17918411358988
N	7.24425629181078	-4.96618397990576	-0.24651462538467
N	7.39391515896930	-4.32693066088880	0.82764278881754

C	7.68689427929756	-2.96298055118423	0.70003953918993
C	7.65558765333762	-2.24811744047552	-0.51068549249678
C	7.81938327003365	-2.26739701896619	1.91133058698849
C	7.80216363707822	-0.88612321347796	1.92499398480024
C	7.65182816966007	-0.86787609341432	-0.48799996950307
C	7.65804193007786	-0.16122994797268	0.73041766933564
H	7.87734574077552	-2.83814531507481	2.83330669856867
H	7.87552920678177	-0.35674302809490	2.87039760446099
H	7.56329089939068	-2.78984466576178	-1.44575137027787
H	7.54796482858926	-0.32031122397371	-1.42073587364691
C	7.34727306465300	1.27415954885549	0.75171600934234
C	6.48071554853526	1.76901948110373	1.73928107604156
C	7.78124958309699	2.16454353042208	-0.24429549461864
C	7.32318842269980	3.47023314886731	-0.28180887629498
C	6.01111056810030	3.06606957646014	1.69191638612910
C	6.39628145947067	3.94498496892439	0.66459107265886
H	8.49755134121569	1.82944129231959	-0.98900595310668
H	7.70127208523114	4.13416166587331	-1.05305717425117
H	6.10623630995379	1.09783175394046	2.50591527239621
H	5.27425686345295	3.37538570516420	2.42539108503488
C	4.21763196514509	7.62705228858356	0.28047903527644
C	3.29614044617440	8.74030301559854	0.11330532461245
C	4.91126064926289	7.08448317629856	-0.81719755722455
C	4.38626514374204	7.00887475852570	1.53472849981944
C	5.15833066669058	5.87573650106633	1.66938204787058
C	5.64573773156858	5.92151032201373	-0.68682660762509
C	5.76134923309670	5.26288772430402	0.55240824012585
H	3.93838645113643	7.45279194789321	2.41898849828567
H	5.30367634723448	5.45758413663744	2.65953638514688
H	4.79635109955090	7.53044622342215	-1.80092928531685
H	6.08355668858081	5.47989979594504	-1.57581985994432
C	3.53575465608399	9.78842621262263	-0.79974331416077
C	2.09759951606834	8.77331216898261	0.86837272865554
C	1.14013225274217	9.73058564660565	0.65764281634517
C	2.63002575959040	10.81415750146016	-0.94666382621051
C	1.38791907276558	10.77892842619347	-0.26652760027077

H	1.90700357832068	7.98777070183445	1.59293743739851
H	0.22318810244432	9.70458302506775	1.23511143609609
H	4.47230129487624	9.81910337108168	-1.34782331386544
H	2.83386779515238	11.65461045876261	-1.60293762868160
N	0.57690805986361	11.85466551423398	-0.48239803946411
N	-0.69314305796223	11.79874945746299	-0.25968040298816
C	-1.57503346690129	10.68078337595632	-0.22893382356115
C	-1.38117714366264	9.61821599578526	-1.11593253185595
C	-2.67502582991498	10.70345283100446	0.62921167569981
C	-3.55918261511392	9.63325368385903	0.63211147893000
C	-2.28309429398298	8.57421810795947	-1.11894891368506
C	-3.37760324272820	8.54671595566791	-0.23506139593459
H	-2.83042288731431	11.54444001924143	1.30000511893122
H	-4.41590841030173	9.65293626219101	1.29890039863789
H	-0.54002065012353	9.61707050472567	-1.80127083720528
H	-2.12912780944792	7.74469476960788	-1.80214699501843
C	-4.27076601570465	7.37717484969781	-0.22916664571162
C	-4.71913548060364	6.81307314321624	0.97241147166901
C	-4.63262094335735	6.75200536371057	-1.43137681539477
C	-5.36166216094553	5.57656619948550	-1.42802826494260
C	-5.43240040295169	5.62375438336625	0.97441305652282
C	-5.73951893398721	4.96292985005632	-0.22368191832788
H	-4.35830509776449	7.20679153895563	-2.37920069379060
H	-5.65063425010270	5.12978309533474	-2.37449114803232
H	-4.44327219639763	7.26997209610490	1.91892401950207
H	-5.69681179347702	5.16804911309471	1.92399363897663
C	-3.40786104364414	-9.41452654619390	-0.27434102159536
C	-4.65812159297568	-8.68238433349528	-0.36488492572835
C	-2.64712773026081	-9.36786409783720	0.91383587268278
C	-2.78915146030718	-9.98797419695721	-1.40935259805198
C	-1.45032858179870	-10.31133163408197	-1.40360438868128
C	-1.30805623906339	-9.69368113920912	0.91803763012638
C	-0.65022306583337	-10.07839048942870	-0.26497917730921
H	-3.36228183436744	-10.16203872733684	-2.31421194374186
H	-3.07435001036090	-8.95858384271838	1.82255736803609
H	-0.73218437960370	-9.52783197722768	1.82259822266636

C	-5.35309123411783	-8.24575252991413	0.79993916564923
C	-5.13331989847882	-8.20999472995612	-1.61077791381784
C	-6.06651190067884	-7.20709926405346	-1.67772850096082
C	-6.27862024332538	-7.23801619043548	0.74980519448507
C	-6.57176862132098	-6.61195197352656	-0.49407450600397
H	-4.68001959393279	-8.55622902175186	-2.53223671489437
H	-6.35072134135924	-6.77054027292146	-2.62990967627890
H	-5.13314836141746	-8.70382862766009	1.75810528155005
H	-6.76629187482323	-6.93515410364523	1.67317681257630
N	-7.14640198996328	-5.40086969776567	-0.68694620397958
N	-7.40608237367491	-4.64676700492595	0.32617396892836
C	-7.64110042375508	-3.27538532727639	0.18720374529205
C	-7.65767645945022	-2.49684324298659	1.35075550554961
C	-7.71982590761618	-2.67181410922078	-1.07372472737217
C	-7.68858980009244	-1.29539188123489	-1.15458779711347
C	-7.62467514468431	-1.11799861035196	1.24923136384821
C	-7.57372468892964	-0.48837918029752	-0.00468972894338
H	-7.76214254716056	-3.28313237692276	-1.96735955341581
H	-7.73455229930840	-0.82630489746582	-2.13245620117681
H	-7.62732644368207	-2.96898145386951	2.33017559166888
H	-7.55307382434625	-0.52422050834543	2.15487940515307
C	-7.26625642118428	0.94413836614599	-0.11771967075217
C	-6.41302898033510	1.37803577698897	-1.14364173066963
C	-7.69278461601763	1.89003219617588	0.82718011105426
C	-7.24647520834225	3.20149039640816	0.77299885286255
C	-5.96006835290222	2.68322353140700	-1.18941050353073
C	-6.34652566858164	3.61937947798984	-0.21962293653151
H	-8.39743826382418	1.60087655515941	1.60198657540405
H	-7.61168744215937	3.91601360107194	1.50499948149606
H	-6.03612700460721	0.66431806309981	-1.86990877411896
H	-5.23953430526191	2.96327509200237	-1.95146434812276
H	-1.00041025707925	-10.72503032757408	-2.30094559273579
H	1.21537353051916	-10.65623978168180	1.69432116684919
H	-1.13562823710682	12.71422078516604	-0.28739588352960
H	-7.24784179714953	-4.97120338545776	1.28298221263386

129

3CAB-1_cis-cis-trans_3H, Eel(r2SCAN-3c) = -3101.32487943454 a.u.

C	3.53673882966696	-9.03016197393720	-0.31709148135671
C	4.78660624510877	-8.26739891274873	-0.23589913826843
C	2.75552748903278	-8.95483184901680	-1.48285068301063
C	2.97592934212405	-9.66925374108150	0.80552201109444
C	1.65074022896212	-10.06209843565684	0.81066380968247
C	1.43247566949541	-9.35591057996189	-1.47991994854481
C	0.82952687403539	-9.84048112701409	-0.30878903127845
H	3.57978751374601	-9.84485387038401	1.69070265672827
H	3.14930839733154	-8.47523476721156	-2.37302085645510
H	0.82879175771042	-9.18318248105891	-2.36546298051753
C	5.53611353694122	-7.93196293541732	-1.38064456375791
C	5.15826602783217	-7.67246950597517	0.98990266286488
C	6.10395021194824	-6.67432383370969	1.05200181937157
C	6.48752710683183	-6.93230341574164	-1.33852981398161
C	6.71925820670263	-6.25203300336530	-0.13426197428131
H	4.62041697515020	-7.92920859024462	1.89609417688374
H	6.31429730529505	-6.16605256964161	1.98542032050597
H	5.34740647621950	-8.43737248600235	-2.32185608211092
H	7.02047199081799	-6.65129052718958	-2.24412909575994
N	7.44416869977338	-5.05922489202825	-0.18260584857393
N	7.59807646917404	-4.31580197053311	0.84901092747197
C	7.92196463276850	-2.99756072241046	0.71705698859314
C	8.05150703809542	-2.26597299917905	-0.49120204309138
C	7.94015107410798	-2.29398013812677	1.94560909076799
C	7.92423382305140	-0.92056580911990	1.96203463859571
C	8.03496057315435	-0.89165084680829	-0.45897692352816
C	7.88348795547094	-0.18551196378558	0.75942957578990
H	7.90903550337870	-2.86829862306787	2.86616844063928
H	7.91167404423818	-0.39870147646569	2.91321623620337
H	8.09133313751695	-2.75402666572223	-1.46142580412422
H	8.04353753297653	-0.34248607261159	-1.39525592076859
C	7.53454587178665	1.23380756960752	0.75918491076852
C	6.62597818253413	1.70540892611790	1.72346099880741
C	7.96373979730100	2.13183623321511	-0.23508371897698

C	7.47121628302660	3.42439184638771	-0.28499639438559
C	6.11671772754740	2.98567924162117	1.65463970792798
C	6.50599481826768	3.87098717021998	0.63580720205956
H	8.71294389451665	1.82406979936972	-0.95889159914646
H	7.84914049056206	4.10103846909283	-1.04515752465133
H	6.24763155053627	1.03155996728485	2.48540193925087
H	5.35209205385194	3.27956701849494	2.36565207042928
C	4.24558179771608	7.49749622469710	0.24253675518668
C	3.29744945100818	8.60135692911974	0.08773514447856
C	4.89355473607683	6.93695192628914	-0.86995993958940
C	4.47630584271435	6.92388217286391	1.50483957422817
C	5.27165644526110	5.80331649862786	1.63772696773978
C	5.65924903606006	5.79064829235149	-0.73826234209037
C	5.83828611754511	5.17735444212053	0.51293834556576
H	4.05533524043799	7.38587373058695	2.39314721415130
H	5.46418217978916	5.41198791055845	2.63135827843317
H	4.72949548355034	7.35601826781788	-1.85860208484065
H	6.07179802639049	5.33190949099662	-1.63124930225895
C	3.52448834809092	9.66731949337886	-0.80174380590959
C	2.10093125718718	8.59544334838230	0.84227771488027
C	1.13118996877593	9.54908267607883	0.65880190475408
C	2.60559086147898	10.68934936103139	-0.92014264137086
C	1.37163895449805	10.62114808443534	-0.23448825174895
H	1.92454897310931	7.79049996140097	1.54912561199522
H	0.21627802336542	9.50003663028538	1.23826655928610
H	4.45919749176561	9.72074540503796	-1.35145184673044
H	2.80127044763678	11.55032417072601	-1.55187773997172
N	0.55973987515321	11.71702180364529	-0.38881224266868
N	-0.70232062273100	11.66706323943084	-0.18706100743822
C	-1.60852905512964	10.57085138317850	-0.17441862577102
C	-1.43130573564538	9.50597351227036	-1.06365424065874
C	-2.72508610001616	10.63053185606674	0.66312061868541
C	-3.63715025632220	9.58633610119347	0.65377871378197
C	-2.36909721962105	8.49635423392475	-1.08943500546327
C	-3.47645673219721	8.49763786051658	-0.21765253323027
H	-2.87013843464013	11.47800526954184	1.32832197185630

H	-4.50315139747041	9.63166767255993	1.30692589040540
H	-0.58222727661570	9.48158216276894	-1.73854719616739
H	-2.23560936671546	7.66942829938722	-1.77983969259717
C	-4.40310496463793	7.35808803009692	-0.22981642102393
C	-4.90086183987080	6.81554899718836	0.96380829396046
C	-4.75301096693777	6.73617109420175	-1.43892483807064
C	-5.51538930597067	5.58351405103955	-1.44767783282790
C	-5.64297452435174	5.64520350042083	0.95391939062293
C	-5.93606107567547	4.98369799336120	-0.24882170607992
H	-4.44784648770482	7.17953140763036	-2.38260306904411
H	-5.79813476734029	5.14522087094285	-2.39967465172497
H	-4.64029184348833	7.27115218273063	1.91510920266621
H	-5.94298662015895	5.20453722983757	1.89971553177404
C	-3.39015243879299	-9.23294843972678	-0.23658829305178
C	-4.66445752909880	-8.52912551059380	-0.33804656657856
C	-2.61557359206557	-9.10267725014022	0.93224371668922
C	-2.78791070996552	-9.85808164299230	-1.34890146224289
C	-1.44403737427122	-10.17393419644069	-1.34486966490871
C	-1.27204850988692	-9.42298429799973	0.93572815521856
C	-0.63742863454124	-9.88341029267472	-0.22884536692691
H	-3.37524089967426	-10.08333078814659	-2.23343380574928
H	-3.03548162797406	-8.64065182490495	1.81921599875402
H	-0.68170429613936	-9.20545840156245	1.82024956119138
C	-5.38716213969893	-8.12573436212757	0.81889154990823
C	-5.12570146824488	-8.06354158521014	-1.58724616049305
C	-6.09478409220916	-7.09217788062981	-1.66377806038006
C	-6.34846353520594	-7.14937394994138	0.75975015465220
C	-6.64251448357114	-6.53212636346318	-0.48517456917714
H	-4.63988324775792	-8.38564948405464	-2.50119738382869
H	-6.37678201770865	-6.65684706721050	-2.61714484201454
H	-5.16366011048181	-8.58376887437904	1.77665238058555
H	-6.86044861529765	-6.86797475035271	1.67681124118826
N	-7.25760704375919	-5.33511204077853	-0.68741832765389
N	-7.55368693949994	-4.58796577763464	0.31104382508260
C	-7.84030494628585	-3.22765489790362	0.16530621034252
C	-7.88627717663939	-2.44672632022352	1.32766121427099

C	-7.92734332558031	-2.63219385346966	-1.10019155917410
C	-7.92089330683708	-1.25754571409868	-1.18613686008999
C	-7.87886290973338	-1.06882145342523	1.22104368199288
C	-7.82085221202211	-0.44373008909358	-0.03644808120581
H	-7.95799056335515	-3.24764279030956	-1.99164595387399
H	-7.97547540841270	-0.79325041543461	-2.16562156156556
H	-7.85769763987614	-2.91453919634730	2.30920371990877
H	-7.82887887558490	-0.47113746592157	2.12532998919710
C	-7.51694646381173	0.98697959318793	-0.15351840242102
C	-6.67414569538683	1.41884578597977	-1.19051211576289
C	-7.92559566018143	1.93443843722641	0.79977372681607
C	-7.46706948177933	3.24064798994155	0.74602225470123
C	-6.20652696922665	2.71786144011808	-1.23376168019792
C	-6.56875201052203	3.65322162811870	-0.25240742254487
H	-8.62687405938617	1.65141158781322	1.57967469054056
H	-7.82110282502034	3.95486454845072	1.48345654708576
H	-6.31419822924782	0.70717262852629	-1.92699389729799
H	-5.49263545577399	2.99172039541705	-2.00384820235690
H	-1.00220685134631	-10.63155794818720	-2.22514365111294
H	1.23782620740995	-10.52984862370600	1.69975863827164
H	-1.12995120346618	12.59256100837690	-0.16110981976485
H	-7.41382777517652	-4.90670581152181	1.27456455765724
H	7.70764484012852	-4.73982990746518	-1.11992352872144

127

3CAB-1_cis-trans-trans_1H, Eel(r2SCAN-3c) = -3100.682585096291 a.u.

C	3.39532022980138	-6.16920979919219	-0.29604805453692
C	4.85492507559182	-6.13606606066663	-0.29212235421158
C	2.67495951124985	-6.18947550264194	-1.50234353641853
C	2.66243045073531	-6.17001281124125	0.90329207108280
C	1.28123302179352	-6.20269577204219	0.89434585837723
C	1.29330472157848	-6.17389812917120	-1.50697948826162
C	0.55314538195764	-6.18693801381489	-0.30989443931169
H	3.18707430961377	-6.20738901576805	1.85383251290921
H	3.20977355399237	-6.15193678166574	-2.44726503942477
H	0.77962227976706	-6.11539770802432	-2.46046454050820

C	5.60753746981237	-6.86974546620755	-1.23001837341203
C	5.55407377362412	-5.36782693901764	0.66576831363555
C	6.92446409421855	-5.26638311388195	0.64735306551536
C	6.98459323372184	-6.85101541438030	-1.20322931954125
C	7.67428392000399	-6.01437933916404	-0.29534741391532
H	4.99222561790252	-4.80831220688861	1.40802272876679
H	7.42489518814033	-4.64947812385553	1.38567339770100
H	5.09528884665205	-7.50793676391273	-1.94362937562950
H	7.56784856800417	-7.46060584765040	-1.88640482029615
N	9.03923809130596	-6.10818343103913	-0.32866599552564
N	9.78549005312367	-5.14477222442378	0.12436974262324
C	9.55410781976508	-3.75567078619433	0.22027482050263
C	8.65695336345314	-3.10888121350342	-0.63840905070103
C	10.31487819267838	-3.01493321528034	1.13206408924375
C	10.14580059342279	-1.64306048416489	1.21609969749553
C	8.51798851378624	-1.74031695325647	-0.55918927660190
C	9.23725762465928	-0.97465828046112	0.37967497310725
H	11.02849646202676	-3.51739208824381	1.78054732234508
H	10.74311692447320	-1.07699629769747	1.92426325317305
H	8.08212425997538	-3.67115430237216	-1.36543959649232
H	7.80868861868706	-1.24714928376931	-1.21615592139521
C	8.97827480812901	0.46469822704589	0.48323832579137
C	8.91965729805435	1.10437095555840	1.73129486762969
C	8.67866939177845	1.22131942167310	-0.66062145161170
C	8.25602758491044	2.53277289597368	-0.55437579509087
C	8.47488395126810	2.41133776228078	1.83767659331525
C	8.08898180314056	3.13924312357916	0.70076402634125
H	8.76890144379250	0.77024102239852	-1.64473111489806
H	7.97773297916865	3.07618684177904	-1.45236159740960
H	9.15812799281747	0.54883420815778	2.63417993429912
H	8.40136939429230	2.87102802924785	2.81903608706252
C	5.55846713846568	6.58931002887819	0.70998100940642
C	4.41306431334218	7.48949188954116	0.51685023866923
C	6.59322194774604	6.47300338356590	-0.23437230439209
C	5.54076481314846	5.66815708897930	1.76965980320348
C	6.43214808461707	4.61178723094097	1.81512136241078

C	7.47735825877216	5.40913290290091	-0.19627738200210
C	7.37772641020229	4.42167289552708	0.79749311537592
H	4.74973832331780	5.71680297137837	2.51098804947329
H	6.32109944284729	3.86172311983681	2.59261898555303
H	6.69078563377907	7.21555669423528	-1.02086877065229
H	8.25246980411886	5.33346634370621	-0.95397220484825
C	3.95828183241632	7.77115894991557	-0.78138441907426
C	3.61831174609014	7.91356464782241	1.60274402438090
C	2.35896130844371	8.43792925552027	1.40560660706779
C	2.69989461596491	8.30571959981399	-0.98936690587038
C	1.85618625952260	8.57009622449444	0.09761537860792
H	3.98687314301601	7.78688210267752	2.61674890359597
H	1.72281105926748	8.69165546940702	2.24667803862381
H	4.54806215916289	7.46305482231845	-1.63900800438416
H	2.30474116558742	8.42947590313499	-1.99314958853466
N	0.50153057862861	8.72831314603402	-0.22659216213582
N	-0.28266982243310	8.81591380715698	0.75679066959075
C	-1.62685410383916	8.53894307823893	0.46794686001845
C	-2.09062809247896	8.08905533152244	-0.78156885861850
C	-2.48927300559042	8.50421607523196	1.57349722223665
C	-3.73228976067509	7.90675203016442	1.46837181456661
C	-3.33279601710535	7.49907736158388	-0.87703796629454
C	-4.14768015190624	7.32524097087849	0.25923548445386
H	-2.13104310387988	8.88991182177831	2.52346916443485
H	-4.37462828196706	7.84699336244099	2.34207830245444
H	-1.42860120367611	8.13063959988116	-1.63925795910732
H	-3.64239853895610	7.06531657713070	-1.82362465740675
C	-5.27750608123299	6.38684509458431	0.20192601437583
C	-5.49273063127257	5.51201758234893	1.27735704496464
C	-6.04855272459236	6.19991753304054	-0.95635193403141
C	-6.92501279227288	5.13258482180494	-1.06522414266250
C	-6.36029324546406	4.44115202958805	1.16375850318850
C	-7.06187106789417	4.19838289469951	-0.02607451596349
H	-5.95505342703657	6.89871340949593	-1.78305716952287
H	-7.50851449785663	5.01535976352417	-1.97399607985439
H	-4.89557429696492	5.61446900563594	2.17837963564167

H	-6.42474774191392	3.72881905218865	1.98046540672231
C	-3.75635409669587	-6.12277599114350	-0.34457080794217
C	-5.22683883486125	-6.08341020801299	-0.36698941419906
C	-3.03676302842671	-5.74618838642442	0.79800993629959
C	-3.02925201907058	-6.53464243907676	-1.47058236402518
C	-1.64626493142072	-6.57443576428227	-1.45220707610252
C	-1.65270969753693	-5.75707011449600	0.80520233267040
C	-0.91816839110759	-6.17543406189439	-0.31802796223345
H	-3.56258970180222	-6.87257242463976	-2.35466513088661
H	-3.57415480679602	-5.39316296174831	1.67384851583208
H	-1.13723602439680	-5.39683367199991	1.68946052594255
C	-5.97846203204776	-6.59412267688159	0.70248510181176
C	-5.91000035626817	-5.55565896671257	-1.47278585064589
C	-7.29224725450427	-5.49734455865123	-1.49626790857965
C	-7.36283368432208	-6.60810034110359	0.65611215894769
C	-8.02623146620680	-6.01928795918361	-0.42392145343474
H	-5.34365000281566	-5.15159377573698	-2.30745247276333
H	-7.81198299963134	-5.06687881342146	-2.34674922222970
H	-5.46598550472289	-7.03810871775880	1.55163876453758
H	-7.94354189027290	-7.06134744942684	1.45422354684980
N	-9.44204527261489	-6.15024679774366	-0.51283446525061
N	-10.19724908659209	-5.20435306198259	-0.78112618149892
C	-9.77739471581720	-3.84605460045582	-0.71926882360580
C	-9.08751698883334	-3.34826270214689	0.39267622942649
C	-10.23032243373942	-2.96942795278340	-1.70953203469375
C	-9.90671293660274	-1.62392585871696	-1.63862383150878
C	-8.82376066159482	-1.99457559271332	0.48300509478768
C	-9.20120979172308	-1.11015635307894	-0.53966945633283
H	-10.82163749342404	-3.36018932658826	-2.53242871818599
H	-10.24395858201766	-0.94947540331461	-2.42077805325642
H	-8.77569209020776	-4.02117518902826	1.18471697442237
H	-8.27732545279352	-1.61418907885709	1.34121081836368
C	-8.82597804611539	0.30963927146299	-0.44762355526304
C	-8.32943915252670	0.99789535214621	-1.56219501887598
C	-8.87494205764873	0.98975837232496	0.77809507083365
C	-8.37915676097850	2.27593842100547	0.89927690876704

C	-7.81804284763821	2.27996567790409	-1.43637363379742
C	-7.79579578932414	2.93303108821095	-0.19513493260616
H	-9.32058182676076	0.50402876246138	1.64177989964361
H	-8.44531171409173	2.78193822979318	1.85798420420186
H	-8.26949211380927	0.49100128433040	-2.52144905927236
H	-7.35732052717151	2.75166517817329	-2.29927630948028
H	-1.12815701371693	-6.95663690979923	-2.32560795453959
H	0.75837099954710	-6.27190025006377	1.84216734537415
H	10.75148421678351	-5.43254426959407	0.25165552311312

128

3CAB-1_cis-trans-trans_2H, Eel(r2SCAN-3c) = -3101.034765652084 a.u.

C	3.53261263626009	-6.41229018538246	-0.07076865894756
C	4.99332845776902	-6.33397803492340	-0.00529298486625
C	2.88397995318408	-6.76199815884883	-1.26706299641724
C	2.73882961094067	-6.11691388748391	1.05046272404157
C	1.35831175897708	-6.16118307954145	0.97548885612022
C	1.50298947307449	-6.77391401188168	-1.34878654472007
C	0.71079013989840	-6.46970876076334	-0.23070557960937
H	3.21015498923519	-5.87803196199025	1.99921405054224
H	3.47020278838054	-6.98688721375332	-2.15322805098237
H	1.02745571604322	-7.04318240593531	-2.28737275834027
C	5.81032080895493	-7.22529700053085	-0.72550987562554
C	5.62243106310991	-5.35049745594421	0.79078180286845
C	6.98915863356344	-5.20555970326997	0.81436318930395
C	7.18394373391137	-7.13983465464055	-0.65416413151601
C	7.80315771821648	-6.10207966538003	0.07911039485177
H	5.01188586026719	-4.66301320464313	1.36814271150150
H	7.43021240982101	-4.42951143593404	1.42920408926762
H	5.35646371227498	-8.02714307743875	-1.29937555747489
H	7.81745626179065	-7.85835918706178	-1.16476522517257
N	9.17269413555722	-6.16840478434748	0.11874933778758
N	9.90699754882662	-5.15305103374629	0.42347808626571
C	9.67306053600443	-3.76203170411872	0.38030033180116
C	8.79254989600650	-3.19801346162679	-0.55128615610743
C	10.41663702596158	-2.94300731159932	1.23881176770901

C	10.22137627565819	-1.57410152482253	1.21948861493864
C	8.63133614022634	-1.83069011440380	-0.57879266158433
C	9.30930454398549	-0.98574442155124	0.32548791057279
H	11.12869452027296	-3.38263900776341	1.93306413465098
H	10.79839894179310	-0.94772654923493	1.89227805686196
H	8.24624999585267	-3.82411531657621	-1.24789987022609
H	7.93342560311193	-1.40079927780127	-1.28986164047391
C	9.00255163382830	0.44491118439030	0.34986195988337
C	8.95483053530988	1.14827856435791	1.56568428834893
C	8.64548388723058	1.13059997758450	-0.82371162532869
C	8.18208286043213	2.43095513140756	-0.77287093350524
C	8.46648786674278	2.44077340868619	1.61765444813879
C	8.02516764829049	3.09554002407676	0.45522227793553
H	8.72971735343482	0.63587528138725	-1.78694886500141
H	7.86786938803017	2.91882391263153	-1.69055521285419
H	9.23817460498962	0.65235989965489	2.48963173615439
H	8.40784863700122	2.94897713653949	2.57551224913191
C	5.44367767816338	6.51159254625080	0.43164500745283
C	4.29953510612687	7.41740633372137	0.27813954555386
C	6.42209049582462	6.35464088127447	-0.56737407331012
C	5.48965160542457	5.63516179767378	1.52939544697869
C	6.39075897734232	4.58894662794881	1.57242080244582
C	7.31539500812106	5.29959253380145	-0.53129436777859
C	7.28411576596099	4.36090425659815	0.51443749196207
H	4.74463584671205	5.71374090425822	2.31410663191382
H	6.33223294312017	3.87765146491304	2.39063647219707
H	6.47460519852274	7.06337292163756	-1.38824493481223
H	8.05052299973177	5.19832787676611	-1.32467293571693
C	3.79262798807917	7.70379476530163	-1.00130679746529
C	3.56490283486280	7.86697558777183	1.39835822955551
C	2.31184329782653	8.41827139604554	1.25655426520651
C	2.54164513036705	8.27012540028067	-1.15522287965484
C	1.75547222186931	8.56038975893811	-0.03024558761336
H	3.98040351807371	7.74808717649008	2.39450499048612
H	1.72478118658361	8.70076592210958	2.12372699364586
H	4.33699865007881	7.38365122329178	-1.88378901331057

H	2.10746156889168	8.40708147916893	-2.14085182081126
N	0.39832254045416	8.76726852009233	-0.28988978634910
N	-0.34228284259200	8.87816047433473	0.72371530883164
C	-1.70095807702638	8.62849689662127	0.50279550065683
C	-2.23794465998809	8.20573985394067	-0.72798337172130
C	-2.50527498557792	8.60226015912071	1.65344804749423
C	-3.76216367387736	8.02948398193932	1.60767278679407
C	-3.49931850235232	7.65363781211346	-0.76643228429373
C	-4.25465500241975	7.48009966801607	0.41151329369648
H	-2.09070852965901	8.97311691026134	2.58604368054178
H	-4.35942453386013	7.97256187751922	2.51288151372308
H	-1.62269468136063	8.25032738668158	-1.61966754455356
H	-3.87413226295379	7.25162035842789	-1.70335701876576
C	-5.40823556531379	6.57294767983379	0.39641945230417
C	-5.58510999117011	5.67527320857901	1.46125586516098
C	-6.25048125431895	6.43777693664484	-0.72052413496621
C	-7.15121624149541	5.39167525318777	-0.80998753242634
C	-6.47467604761968	4.62257806195204	1.36482523751456
C	-7.24272834050868	4.42366030992684	0.20604265878993
H	-6.19319416222123	7.16194583767774	-1.52810945189974
H	-7.79036574801224	5.32109119452852	-1.68499871665906
H	-4.94141090464280	5.74456305986704	2.33264766173588
H	-6.50349584329891	3.89190347824370	2.16658926317139
C	-3.57599532285306	-6.29791351860297	-0.48766881171606
C	-5.03385130909928	-6.19159372067973	-0.56199211477003
C	-2.94508315274455	-6.85384966149840	0.63815773583913
C	-2.76626581872501	-5.83407904391244	-1.53853784352494
C	-1.38827793657137	-5.92022336303154	-1.46492712021536
C	-1.56503269974989	-6.90827584222258	0.72411560447605
C	-0.75734650970492	-6.44209781720863	-0.32508000845650
H	-3.22405573655859	-5.43108695312553	-2.43699126838974
H	-3.54345854755639	-7.21137682317611	1.47105670314987
H	-1.10299327942863	-7.33957103170714	1.60737922551170
C	-5.87358627971052	-7.18173903627806	-0.01775219821356
C	-5.63760968987299	-5.08571312583820	-1.20245809317729
C	-7.00241262445554	-4.93491393947918	-1.25385993676949

C -7.24330778046609 -7.08237240668631 -0.12599617515551
C -7.84102661042765 -5.94378588879364 -0.71583692335084
H -5.00847880042843 -4.31309158222260 -1.63353488164948
H -7.41958122058300 -4.06543435796086 -1.74755443259673
H -5.43854984618751 -8.06690692971146 0.43551404384933
H -7.89250877990921 -7.87349201985384 0.23600347705480
N -9.20327176598529 -6.02256390327035 -0.84317857474604
N -9.93985860107783 -4.99104815262214 -1.07749296566697
C -9.71734520993811 -3.60987835000967 -0.86365259593212
C -8.99608978911783 -3.16881245831981 0.25140183421770
C -10.29477146199295 -2.69406906518521 -1.74877154990768
C -10.08034940843483 -1.33928328071774 -1.56232985274779
C -8.82443603267108 -1.81514304840385 0.44514842418876
C -9.32648172720257 -0.87052976477605 -0.47311175578725
H -10.88344765977543 -3.04476498391923 -2.59274412349567
H -10.51952319120466 -0.63072152582949 -2.25769550897332
H -8.58186640873241 -3.88087909011776 0.95756908302434
H -8.25483539096872 -1.47274806346253 1.30348539507623
C -8.99871374828275 0.54672381124693 -0.30558620379489
C -8.66862844573711 1.33857432004765 -1.41650652453876
C -8.90442853750484 1.12774327298457 0.97035501737997
C -8.42848233088225 2.41426714691438 1.13043139875986
C -8.16222817634029 2.61488250556304 -1.25125286001422
C -7.98682900200413 3.16946403718802 0.02835627302857
H -9.23122475304408 0.56895007126352 1.84293751691928
H -8.39450042048802 2.84251316929566 2.12706052748216
H -8.72212689168052 0.91556121016014 -2.41572597035450
H -7.82282402089369 3.15998887206856 -2.12601111761330
H -0.78666930773471 -5.54646653631016 -2.28822430250627
H 0.76917732928889 -5.91785672869583 1.85486197562683
H 10.87026448518702 -5.42184466925193 0.61336604768307
H -10.88577560383708 -5.24708843538050 -1.35246615483316

129

3CAB-1_cis-trans-trans_3H, Eel(r2SCAN-3c) = -3101.329519927805 a.u.

C 3.53996686923870 -6.58091914810657 -0.03875845131459

C	4.99814263214364	-6.46909197395009	0.03016772330017
C	2.90506915800575	-6.94377656621117	-1.23894324735784
C	2.73672977602468	-6.29657790980620	1.07899730112479
C	1.35752616635983	-6.35846961160307	0.99486308951445
C	1.52484474534980	-6.96805094740317	-1.33091242588673
C	0.72310042622750	-6.66857784217303	-0.21818621817229
H	3.19874376261889	-6.05419554224380	2.03140718666784
H	3.49955387332585	-7.16517627451623	-2.12040811133372
H	1.05879273732043	-7.24291675410246	-2.27258384276763
C	5.83797351013914	-7.34680796993546	-0.68039758926384
C	5.59844874135763	-5.45870862339130	0.81806491998796
C	6.95861946914843	-5.26849816864882	0.83235291947336
C	7.20863119933281	-7.22149793313388	-0.60918229592605
C	7.79416020782818	-6.14976984998953	0.10316847415921
H	4.96793192345575	-4.78754696647861	1.39288123849440
H	7.37875392023176	-4.47261544423532	1.43645457288044
H	5.40714202891371	-8.16914974608466	-1.24267308580031
H	7.86346265269889	-7.92982947608605	-1.10712723316481
N	9.16543245375412	-6.17318413347337	0.13023806670013
N	9.88366522930260	-5.14826011731297	0.39700338168317
C	9.61798276508443	-3.75620141332140	0.37303780597832
C	8.71177826301375	-3.20689263080099	-0.54074443988707
C	10.37055324068964	-2.93154574098686	1.21606063213294
C	10.15925097772787	-1.56364468195837	1.19906971758829
C	8.53411839883832	-1.84115650656073	-0.56540019575890
C	9.22428268661463	-0.98850687631437	0.32193382346977
H	11.10420262643745	-3.36137372963438	1.89368890807265
H	10.74514973131270	-0.93047751498658	1.85762636009743
H	8.16249260073021	-3.83938393312936	-1.22936810558169
H	7.81952231834765	-1.41971276144821	-1.26473532102287
C	8.91239565082076	0.44461670804192	0.33932435766108
C	8.88548816424189	1.16051041317910	1.54744393021543
C	8.53902155273538	1.11607925990536	-0.83628781193838
C	8.07985000300065	2.41923369432156	-0.79315529697781
C	8.40633183559040	2.45813316805732	1.59152907240629
C	7.95173640716091	3.09959880531790	0.42829214892688

H	8.60953302210106	0.61195144498695	-1.79560314048508
H	7.75469921895326	2.89648663680286	-1.71257831828845
H	9.18272554237127	0.67560359958528	2.47276766672359
H	8.37121189955315	2.97846371458503	2.54402851301505
C	5.42418048348882	6.55662594509884	0.39360710887340
C	4.28766726744157	7.47057460602296	0.24331757850354
C	6.37761828834184	6.36628238720486	-0.62265879828766
C	5.47337750635190	5.70101025449275	1.50716052359154
C	6.35395293955761	4.63644935641055	1.54891426398524
C	7.25878372313785	5.30100893478557	-0.57917968573995
C	7.22534081032099	4.37809531998187	0.48044942510892
H	4.74812852942411	5.80814700851555	2.30735516524921
H	6.29932380642088	3.94098426770233	2.38059550670283
H	6.42919766197531	7.05987283869626	-1.45680943320590
H	7.98266085320824	5.17793474765028	-1.37939822712626
C	3.74287996106643	7.71724491217161	-1.02992838222352
C	3.59819366327067	7.98175325907234	1.36618927656682
C	2.34809607615199	8.54852055749826	1.24932265806970
C	2.49543897613731	8.29088942473546	-1.16790607313605
C	1.76377308991526	8.63150927245182	-0.02220144219169
H	4.04432645694331	7.90013429886696	2.35208240927209
H	1.80120939703875	8.88109614556959	2.12408887345679
H	4.25780524748861	7.36668893100532	-1.91781956569826
H	2.05586421163033	8.40050010300477	-2.15680927448355
N	0.40154194430926	8.88152554698132	-0.19199965024641
N	-0.41907836027275	8.96026863966484	0.78948949675089
C	-1.75217273807589	8.77738886445167	0.56573573758456
C	-2.37714058167333	8.53057445067745	-0.68380376988216
C	-2.51527822920970	8.62640524447055	1.74861528650689
C	-3.76454702317380	8.05492654254048	1.70039163270985
C	-3.62668596646369	7.96339066338254	-0.71628850550631
C	-4.30803761965287	7.61195998046897	0.47630835747570
H	-2.05724636597200	8.89890962364989	2.69429165806722
H	-4.31703408471564	7.90664400074827	2.62229183596954
H	-1.87945102932230	8.72644954330786	-1.63009197006767
H	-4.05677749187615	7.69621578968747	-1.67607653443023

C	-5.42389715440362	6.67177949590789	0.43020902491297
C	-5.57131978365731	5.74192945385943	1.47590793629928
C	-6.25834057499811	6.53048491201074	-0.69543241235299
C	-7.12626479400060	5.46001520244418	-0.80596806460065
C	-6.42555171111107	4.66477404643119	1.35445252573081
C	-7.18691022335575	4.46929209031711	0.19087616495898
H	-6.23691982828006	7.27446738855822	-1.48631497595493
H	-7.76619686739875	5.38717023727204	-1.68007696621180
H	-4.93493515686629	5.80621286918941	2.35240196264108
H	-6.43726866970048	3.91675607589697	2.14033092426919
C	-3.55726147963590	-6.44698894957425	-0.50135718544860
C	-5.01057732428364	-6.30345489545279	-0.57415160994150
C	-2.94280594619621	-7.03824365487329	0.61658037683101
C	-2.73550749545849	-5.97820325678306	-1.54165452894426
C	-1.36006638207298	-6.08952272459914	-1.46278600543975
C	-1.56435411735529	-7.11199291822077	0.71055057678217
C	-0.74472014313803	-6.63611139664330	-0.32540106006613
H	-3.18147259347588	-5.55448902500973	-2.43646315267631
H	-3.55080395235790	-7.40384288928276	1.43884012791545
H	-1.11386856546696	-7.56537369329154	1.58862715089213
C	-5.87617128863451	-7.28224124347759	-0.04878813334974
C	-5.58278412620091	-5.16481912060222	-1.19180758511482
C	-6.94057667095801	-4.96885725208254	-1.23027499110676
C	-7.24179761108581	-7.14076997111863	-0.15420309623167
C	-7.80479501933897	-5.96640251141272	-0.71005014679130
H	-4.93085088811645	-4.40497491334366	-1.61100901127902
H	-7.33397660735402	-4.07611373397957	-1.70120406885090
H	-5.46645845755716	-8.19018666360276	0.38205899494818
H	-7.91440043497778	-7.92172145851647	0.18681540803323
N	-9.16845558833417	-6.00575210725409	-0.82048338677068
N	-9.89483560111441	-4.97401697769998	-1.02865516673116
C	-9.63665228256317	-3.58433995392829	-0.85405677953550
C	-8.92622811820901	-3.13882548235575	0.26386346347303
C	-10.18445929443583	-2.68150489369931	-1.76727848330019
C	-9.95260519275798	-1.32484410233349	-1.60001648899822
C	-8.73465762439439	-1.78334946314550	0.43500602388147

C	-9.21319822763835	-0.85071927893450	-0.50570760699133
H	-10.76670497744556	-3.03808657190102	-2.61302870710467
H	-10.37151748382708	-0.62248132267111	-2.31402970919366
H	-8.54025944635792	-3.84469652569788	0.99237317590881
H	-8.17698817020718	-1.43336806542589	1.29812583177988
C	-8.88422201648642	0.57288254410024	-0.34507750564167
C	-8.49971086670802	1.34456109974502	-1.45061513545900
C	-8.85665222222163	1.17019849717478	0.92495474829513
C	-8.38925293768973	2.46090638610364	1.09004633957791
C	-8.01261562245031	2.62988804631864	-1.28095388673281
C	-7.90687213219892	3.20058387229938	-0.00291128299569
H	-9.22469089495684	0.62276123565424	1.78812091440276
H	-8.40220781832621	2.90385404103950	2.08109547897820
H	-8.50580373877709	0.90654779551190	-2.44471309103870
H	-7.64030180804469	3.16864614428420	-2.14668769108752
H	-0.74761555403869	-5.71465735269809	-2.27744008189485
H	0.75949773893563	-6.12513862010182	1.87087022899491
H	10.86125730223853	-5.39589290985574	0.54851385983816
H	-10.85869091156233	-5.21864733472791	-1.25370079391085
H	0.06643656569229	8.79992768245293	-1.15633060916176

127

3CAB-1_cis-cis-trans_trans-1H, Eel(r2SCAN-3c) = -3100.679546356550

C	3.58458756624353	-6.06246016417369	-0.21601839646501
C	5.05735562437310	-6.08669347633597	-0.21031500726916
C	2.87669235102585	-5.69533095755120	-1.36831546087241
C	2.84758776543466	-6.40690852099771	0.92491503194902
C	1.46172593291222	-6.39677877433474	0.90934046176943
C	1.49177447273071	-5.66327194885830	-1.37545141778389
C	0.74955818528035	-6.02005676759133	-0.23933505820330
H	3.36969407619081	-6.73582836407131	1.81933693152612
H	3.42369838485334	-5.38997907775845	-2.25628629518306
H	0.97783035948213	-5.32307075016329	-2.26943015615495
C	5.76892825641320	-6.62624724633771	-1.29302205792689
C	5.78364199613917	-5.59157672049941	0.88373994730821
C	7.16802121602460	-5.58405119337326	0.87996240756222

C	7.15257593709895	-6.68290035170455	-1.27885610130196
C	7.85993413419151	-6.11660389402527	-0.21462741096390
H	5.24960000608054	-5.17509134115668	1.73343288881759
H	7.71843114794398	-5.17904282954854	1.72374255980441
H	5.22348111179237	-7.05516572608751	-2.12899188647245
H	7.70247870577909	-7.15014835050385	-2.09055451865190
N	9.27557735537086	-6.26477000406572	-0.21201447515728
N	10.05837870761895	-5.35215085574166	0.09350976490725
C	9.67443388050933	-3.98687165353255	0.19355813767491
C	8.87255110558363	-3.36523837002663	-0.77253944353398
C	10.28283242954739	-3.21645487539522	1.18985688296727
C	10.00955105098157	-1.86212446365252	1.28158855283451
C	8.64193488433821	-2.00562677057378	-0.69767074537026
C	9.17943635287357	-1.22866881920310	0.34299025531207
H	10.95978383587097	-3.69889588697206	1.88861449708891
H	10.48027719015946	-1.27679438326955	2.06616351023100
H	8.43341056824491	-3.95018163064111	-1.57387431458912
H	7.99221251873600	-1.54087456096261	-1.43310363935918
C	8.83630277640168	0.19708757637269	0.44519850735426
C	8.64423075029709	0.80831347196675	1.69391917732807
C	8.61024837333674	0.97127140703812	-0.70298037573713
C	8.13335371750306	2.26640835246058	-0.60868360265865
C	8.16049232583247	2.10177383027360	1.78860511453948
C	7.85492709675285	2.84604442824354	0.63856704808175
H	8.80059663723994	0.54605079446131	-1.68387488919184
H	7.91301986065768	2.81546782405081	-1.51940165995005
H	8.82133575395118	0.24050241495036	2.60270858920131
H	8.00243243295342	2.53725888772104	2.77107174417189
C	5.35930638842588	6.33262008007795	0.61724423682491
C	4.25203128512061	7.27339150639338	0.43612247321278
C	6.39281015306859	6.19130574266034	-0.32544748615735
C	5.30567064480867	5.40249011939866	1.66923200779459
C	6.16752042075299	4.32364879808640	1.71312602021528
C	7.25951833210101	5.11485095256918	-0.27570141955025
C	7.12945835545196	4.12160960308677	0.71136645646704
H	4.51020192614586	5.46418657911314	2.40528427953675

H	6.02843521485426	3.57120484635476	2.48292091171674
H	6.51724009273557	6.93493814528394	-1.10762174162907
H	8.04775965813656	5.03332635471256	-1.01826993514968
C	3.80238801278975	7.60037310402617	-0.85615365509867
C	3.48579520594229	7.72923444333866	1.53035772667611
C	2.25638835179254	8.32684828525462	1.35583751358713
C	2.57697783942837	8.20439918709927	-1.05108365939850
C	1.76456097315990	8.49612524370454	0.05357747769500
H	3.85196823280827	7.57324487595893	2.54019261103359
H	1.64685658036283	8.61099785395935	2.20553184776602
H	4.37377087664883	7.28425905024779	-1.72253932862984
H	2.21001945676139	8.37227890064828	-2.06125490356408
N	0.42822691160969	8.79515857906712	-0.19093089987392
N	-0.44956404777012	8.87855116976153	0.75989655076257
C	-1.75757785071585	8.65726032619309	0.46107257177594
C	-2.29087861174014	8.30882882655912	-0.80828561128625
C	-2.61539446948467	8.58857297896037	1.58764810272095
C	-3.85795956313251	8.01327742560267	1.48576340887445
C	-3.53663143341468	7.73696571208112	-0.89440206612845
C	-4.31547967498343	7.48172810096573	0.25950712568209
H	-2.23165248840398	8.93116591780238	2.54353055257531
H	-4.47792802869269	7.93029283411848	2.37250056096398
H	-1.71474500061421	8.40607878537891	-1.72447597094959
H	-3.88171650621384	7.38090812439274	-1.85972009802397
C	-5.42715907978678	6.54199975864160	0.19593132616073
C	-5.68296577196211	5.70318364040592	1.29713960794603
C	-6.15214477542208	6.29889760185002	-0.98752322139866
C	-7.00626815273318	5.21856666466356	-1.09105682473964
C	-6.52378444698014	4.61537799875026	1.18547891050212
C	-7.16719973815301	4.31110018052201	-0.02654792331558
H	-6.04966563053237	6.97199538418212	-1.83380352130368
H	-7.55636164073265	5.06616243195936	-2.01460546298993
H	-5.13075878514672	5.84142348682833	2.22105471156243
H	-6.60921176393042	3.93384578953299	2.02536249882452
C	-3.56006816960462	-5.96905096410737	-0.29942639857045
C	-5.03283610761043	-5.95247506436049	-0.32770559599975

C	-2.85316792054059	-5.75277683047959	0.89077928533106
C	-2.82262429925012	-6.20762863684935	-1.46687319080733
C	-1.43784068381897	-6.23573185279873	-1.44308840530696
C	-1.46719825873578	-5.75771111013647	0.90848994746640
C	-0.72534200459696	-6.00338213495623	-0.25690656953598
H	-3.34580994755851	-6.42396792793057	-2.39431879285006
H	-3.39958248982292	-5.53486436674584	1.80457988445693
H	-0.95167501955178	-5.53331166398426	1.83753982670699
C	-5.78129457157101	-6.59775555760895	0.66873849704151
C	-5.72168388469600	-5.32060050327012	-1.37418303771861
C	-7.10504456489780	-5.29735132869134	-1.41009167370961
C	-7.16464485111134	-6.63671123647188	0.60697421227034
C	-7.83520402654142	-5.95096148466502	-0.40986664347479
H	-5.15847123139143	-4.81728574401798	-2.15519869655617
H	-7.62442386569068	-4.79596205490488	-2.22079839578793
H	-5.26515625288406	-7.12612486119721	1.46550671858845
H	-7.74194965340759	-7.19060943804105	1.34152683337070
N	-9.24791248824196	-6.11037769546111	-0.49772889937458
N	-10.01975478889915	-5.17577667415087	-0.76132998439867
C	-9.63565445707703	-3.80756089244711	-0.70258849007415
C	-9.00295387994749	-3.28399283233419	0.43172032622629
C	-10.07920357575497	-2.94872861663705	-1.71280761415700
C	-9.79926282745350	-1.59440247192056	-1.63760344375978
C	-8.78245079749666	-1.92310073597763	0.52419323754684
C	-9.14786975155633	-1.05493521024644	-0.51744895100932
H	-10.62822315509190	-3.36019521313880	-2.55462911061190
H	-10.13022369676332	-0.93531168388140	-2.43543619044435
H	-8.69289210984360	-3.94724947766261	1.23296530317607
H	-8.27218615411206	-1.52619423727185	1.39708677663789
C	-8.81195259749125	0.37266596911635	-0.42880507466324
C	-8.33553033429671	1.07291365539002	-1.54654727754256
C	-8.88320615015216	1.05891190553316	0.79432893449359
C	-8.42310533237799	2.35690478841355	0.91150106713833
C	-7.86681091894012	2.36991983581262	-1.42777650714552
C	-7.86208337331146	3.02925609695846	-0.18758975472122
H	-9.31822243796491	0.56594517507524	1.65886613075048

H	-8.50841514941983	2.86206527800919	1.86895103411695
H	-8.25753666114178	0.56399336519636	-2.50299351508423
H	-7.42161745549765	2.84802624783596	-2.29503503677654
H	-0.90120577570381	-6.48231459421362	-2.35423000255042
H	0.92334425933335	-6.72673539071616	1.79277141194485
H	0.14026312537301	8.71758652131221	-1.16768803395581

128

3CAB-1_all-cis_cis-2H, Eel(r2SCAN-3c) = -3101.010927894287 a.u.

C	-6.86125630215048	3.50583115473653	0.14958902674616
C	-6.67052678529159	4.95381927357587	0.18452445013814
C	-6.73174175731873	2.73010194895652	1.31575950469917
C	-7.13250070042688	2.83299595056167	-1.05701302068696
C	-7.23559318932293	1.45812910867321	-1.09953519712816
C	-6.80750244957113	1.35324951292600	1.26694547033646
C	-7.04543647272864	0.67289606757536	0.05562509334389
H	-7.30582166566876	3.40604689974311	-1.96305339995121
H	-6.49731024014660	3.21443090265888	2.25902506398146
H	-6.62297818305222	0.79312399570525	2.17712582804070
C	-7.07907312862390	5.73081023570642	1.28439251671084
C	-6.02137744136269	5.61074726803878	-0.87831330277524
C	-5.75341853397044	6.96246367387712	-0.83713606319915
C	-6.84057430657356	7.08969538452850	1.32855974359243
C	-6.16443948163143	7.72077718248748	0.27034792093185
H	-5.68173291081001	5.03540466279146	-1.73419847751393
H	-5.22325771510286	7.43460694003933	-1.65715839608419
H	-7.61637010486960	5.26306550796640	2.10384338637123
H	-7.17700456914167	7.67287411157820	2.18239764001880
N	-5.93277097635831	9.08528073304808	0.33086671596649
N	-4.89968147252853	9.68020233854559	-0.35199419810663
C	-3.60264544288448	9.19747132105033	-0.28975612840572
C	-3.15333172524410	8.46199225165029	0.81824855131719
C	-2.71661008695844	9.46690406296697	-1.34693434233775
C	-1.42083935395689	8.99326949562388	-1.30108179285191
C	-1.84904760685276	8.01743297554536	0.86104402334367
C	-0.95404924917728	8.25076978222795	-0.20062147839829

H	-3.05206149545452	10.05005413818715	-2.20116144153008
H	-0.74592951874933	9.22435672484366	-2.11966439470905
H	-3.82845339352957	8.23916977042043	1.63737920703992
H	-1.52202486881755	7.43538247399494	1.71730793476087
C	0.39508173894985	7.69148567324468	-0.16386803066783
C	1.00339744690470	7.19078057978750	-1.32907464579674
C	1.11192369037265	7.59027627081344	1.04361507154590
C	2.35404766559746	6.99202525080175	1.08787561330206
C	2.23352489236324	6.56780716126306	-1.27850322891905
C	2.94040704845395	6.43421605512254	-0.06634211107404
H	0.70124970189690	8.02724150573624	1.94901815082299
H	2.89137673928896	6.98026954426202	2.03011999869387
H	0.46788946705232	7.22963575096534	-2.27300816614008
H	2.62729735514890	6.12759824854475	-2.18810638394995
C	6.55302696228607	4.10638625376281	0.08370703108664
C	7.70366165859411	3.20689162120701	0.11455780359907
C	5.82124519086144	4.38556174623750	1.25181371914950
C	6.11106518991221	4.68275575691443	-1.12175012564077
C	4.97919104174684	5.46967561585292	-1.16129680642482
C	4.67074150673624	5.14641386245493	1.20455797515542
C	4.20482169995299	5.69861066352501	-0.00551231411460
H	6.69258497436727	4.54356503160328	-2.02820819377702
H	4.70368429043674	5.93558485906778	-2.10122512572098
H	6.12246793260440	3.93720086364533	2.19392466306326
H	4.09415772971373	5.26872071321565	2.11493029536079
C	8.59390848468385	3.18950775933689	1.20687104070772
C	7.93808444624439	2.32076441634537	-0.95637570065493
C	8.96890694083150	1.40955404734043	-0.91426471931593
C	9.67570606386659	2.33202424612337	1.22868538463424
C	9.82890602370779	1.39087575232276	0.19974913778287
H	7.26349790514442	2.31844171710703	-1.80700137584558
H	9.12724086754813	0.71315313994299	-1.73147980401570
H	8.46059160229974	3.89813990119950	2.01884983882569
H	10.39476035621525	2.35465451187814	2.04169739496232
N	10.95657622233863	0.55629734009693	0.20269279393516
N	10.96872014847401	-0.62917179193926	-0.11993867344774

C	9.82652013235162	-1.45406765827367	-0.09943229162604
C	8.89661338249676	-1.38440513680455	0.95069593808770
C	9.71054733669915	-2.43919745142785	-1.08868141786860
C	8.59989287037263	-3.26370825520616	-1.09600453291644
C	7.83355546588780	-2.26388657100504	0.96940642827076
C	7.64514226780578	-3.19521252226178	-0.06666015241858
H	10.47940735592329	-2.52055345981035	-1.85092627659829
H	8.48829485862377	-4.00868013462534	-1.87844314817079
H	9.02879492085405	-0.66242644580176	1.75012554621234
H	7.11126969627581	-2.21588937930898	1.77913089489631
C	6.46312859207074	-4.07083674258466	-0.05903600538936
C	5.70185625110998	-4.25047493558174	-1.22059472635396
C	6.04199585575673	-4.70572859279746	1.11691647473414
C	4.89236766860846	-5.47847693150819	1.13269653375283
C	4.53205456895977	-4.99309347531753	-1.19218559998186
C	4.09649113136260	-5.61651923091589	-0.01402972616498
H	6.64468747672305	-4.62377478285621	2.01734851899544
H	4.62035492149954	-5.99826057679148	2.04617460339226
H	5.99812566372374	-3.75214792072953	-2.13969357425739
H	3.92665543923946	-5.05277623958627	-2.09127745185004
C	-6.83376725953912	-3.61964699261821	-0.08887826587564
C	-6.62909863006114	-5.06576917531853	-0.11748120085603
C	-6.70868921536087	-2.84775727170684	-1.25782306981899
C	-7.11397264347402	-2.94737512195384	1.11537862985621
C	-7.23029761339377	-1.57371332471329	1.15305555935416
C	-6.79319343547216	-1.47094145299282	-1.21243187427138
C	-7.04022884457572	-0.78999552799620	-0.00348082081969
H	-7.28517186567062	-3.51955033741895	2.02229858625269
H	-6.46947946764934	-3.33383220667269	-2.19901892731079
H	-6.60986757976722	-0.91143229211875	-2.12321756174638
C	-7.05712119539495	-5.84733718708153	-1.20926084370830
C	-5.98001451562478	-5.70991605054321	0.95531075407582
C	-5.70563309882676	-7.05819648850060	0.91570608555530
C	-6.85479083129100	-7.21286857773401	-1.22865306086914
C	-6.11765626138882	-7.81423185390237	-0.19765480305696
H	-5.64203437721767	-5.12544877685071	1.80549267583748

H	-5.18254657768071	-7.54201317922637	1.73435983791399
H	-7.60321112558346	-5.37915125540424	-2.02279286172045
H	-7.23254712646717	-7.82570457070728	-2.04122098324422
N	-5.95868345020436	-9.20808044242353	-0.19801993412259
N	-4.93858832793256	-9.81117441493162	0.12637816833391
C	-3.65291342652067	-9.23473982047125	0.10633446736995
C	-3.24759594268200	-8.39433861417931	-0.94329947205315
C	-2.74236077986795	-9.62746247091084	1.09584087226267
C	-1.47272195934951	-9.07842793738608	1.10381986738145
C	-1.95426782584281	-7.91378012689975	-0.96123896282495
C	-1.05397663297962	-8.21705880530617	0.07501403746354
H	-3.05691306456800	-10.33412751841560	1.85772189788050
H	-0.77218718489304	-9.35493793699365	1.88641089770818
H	-3.93850147981912	-8.14726257851890	-1.74294063658247
H	-1.63418511438858	-7.26400869382024	-1.77056028920445
C	0.29557817410798	-7.63171229097488	0.06784841850950
C	0.83200142833632	-7.06308787722472	1.22969317373664
C	1.05591823436864	-7.58395334184482	-1.10809387116095
C	2.29994050157680	-6.97469703997610	-1.12368664906863
C	2.06013280976161	-6.42143607779332	1.20154301868969
C	2.81759080163245	-6.35506778592516	0.02329239118383
H	0.68350529332728	-8.06443056016505	-2.00874906663043
H	2.88595667847663	-6.99847354498390	-2.03727075237042
H	0.25242472514029	-7.07101058664358	2.14886659962819
H	2.41470013403469	-5.92769430080307	2.10095879683265
H	-7.49752775053330	-1.10105901098229	2.09201870938273
H	-7.49438385462883	0.98566973536697	-2.04108422957461
H	-6.29207815608558	9.60328815086836	1.12284799670261
H	-5.16728487869153	10.25073705998045	-1.14414829054927

128

3CAB-1_cis-cis-trans_trans-2H, Eel(r2SCAN-3c) = -3101.001828479822 a.u.

C	3.59109307749314	-6.76958297411204	-0.27301605716124
C	5.04363441221986	-6.62138995482040	-0.25161309319690
C	2.86255447433256	-6.56548374531099	-1.45947097113929
C	2.86905544309307	-7.07823026547004	0.89567341724120

C	1.49199332103985	-7.15296406062468	0.88270037288029
C	1.48383490433779	-6.61222459911721	-1.46437528025799
C	0.75494551620183	-6.89481056463473	-0.29145876828201
H	3.40347277719351	-7.30711183092129	1.81277429598098
H	3.38652385167853	-6.29816543518253	-2.37219211324719
H	0.96116623881567	-6.37286525061331	-2.38400524524092
C	5.83484388031429	-7.00951759244173	-1.35193681923500
C	5.68786556412005	-6.06725279359857	0.87410490827938
C	7.04603276212056	-5.84282873543738	0.88027108503669
C	7.20584322198216	-6.85374862569716	-1.33322460944719
C	7.81016335928224	-6.20575406125859	-0.24503594995182
H	5.09791700832915	-5.75902479708941	1.73174589698260
H	7.53098629327100	-5.38835692889751	1.73846052894622
H	5.36726679726479	-7.48780614148230	-2.20728493350448
H	7.82208141561822	-7.19974656879013	-2.15721628509101
N	9.20615956056922	-6.09340123563268	-0.21006214677450
N	9.86156724212415	-5.11732829494862	0.14009457871791
C	9.34724029739640	-3.80146011198830	0.16653040742482
C	8.44387395213854	-3.33450306461872	-0.79948235321002
C	9.85945048814517	-2.93502852694763	1.13949699201009
C	9.37852086566570	-1.63895855722235	1.21627288485700
C	8.01801250579488	-2.02189080760605	-0.74498049849911
C	8.45070546021469	-1.16114394153314	0.27762548141680
H	10.60865950353475	-3.30177047951687	1.83440705430042
H	9.75559687867692	-0.97048670258295	1.98469419332714
H	8.10089574658486	-3.98842202577993	-1.59451450216727
H	7.31583362113263	-1.65528625717262	-1.48805466516531
C	7.91079994112781	0.20943762124493	0.35228001334124
C	7.45855480126016	0.72710052990330	1.57209953221786
C	7.77686029318686	0.99485963467465	-0.79933846591872
C	7.17608499403957	2.24313677091267	-0.73941269963545
C	6.83338038980755	1.96345082983542	1.62651547892274
C	6.66486368078813	2.73361956645965	0.46848708118928
H	8.15406768761793	0.62630854642696	-1.74925354151545
H	7.05875165554560	2.82628894437987	-1.64852281987807
H	7.55811901134347	0.13517983645434	2.47785378537374

H	6.47512145904172	2.34223791389183	2.57965148554848
C	4.29762729029189	6.33022052618941	0.45968277689699
C	3.41829950328198	7.49976426329806	0.38935345626951
C	5.53361790856623	6.28131025567322	-0.21205501501552
C	3.89993610380115	5.18496054918081	1.17493021850814
C	4.68476833326090	4.04873035105240	1.20176784256236
C	6.31227021569261	5.13662551415382	-0.19640111715919
C	5.89862824245621	3.99275588575394	0.50217917030574
H	2.93482077966455	5.16199668921962	1.66993811047853
H	4.32933489346928	3.16484208292070	1.72347272224657
H	5.90505833403413	7.15959187652819	-0.73078244580471
H	7.26939385083350	5.13289202882037	-0.71034757701124
C	3.45771117064184	8.39413977822606	-0.70244596781937
C	2.45042109210230	7.73915007438783	1.38246403109593
C	1.52764330333029	8.75821758779215	1.27132956494193
C	2.56060345051619	9.43388281222471	-0.81783217146027
C	1.55407804602020	9.60538068262101	0.15061096165812
H	2.42033036451145	7.11366622495359	2.26803244833636
H	0.77932574186131	8.88787459326814	2.04499056429637
H	4.17783722547722	8.24178144097878	-1.49955635547677
H	2.60272512941448	10.09597066973200	-1.67900299473285
N	0.60918907449087	10.60429229358441	-0.02156163019547
N	-0.52077384733692	10.63445036357816	0.76153726431480
C	-1.52061416265996	9.68945396167258	0.57924317279704
C	-1.55197577860538	8.87050962211049	-0.56190048162144
C	-2.51994213552536	9.54983876088285	1.56058764712649
C	-3.47104938062840	8.55932653932522	1.43680583616593
C	-2.53843484365161	7.91330801173042	-0.69053862072233
C	-3.49984920874403	7.70793353812674	0.31359562428742
H	-2.52763635562477	10.20895566876729	2.42505926725670
H	-4.22511547542926	8.45035141722748	2.21076392470573
H	-0.78606220118851	8.95659666498333	-1.32436816206364
H	-2.52444429856610	7.25848365454150	-1.55635528514883
C	-4.43535509403883	6.58693235700495	0.21608151120482
C	-4.74350960962709	5.81297540309808	1.34772280272805
C	-4.98513851934359	6.19713963002587	-1.01781697715336

C	-5.76946696131407	5.06392440536007	-1.12054841391308
C	-5.49995829413201	4.66107790449973	1.23561657743780
C	-6.01383789307415	4.24462316188204	-0.00403007858789
H	-4.81935632307918	6.81270276078037	-1.89741685927317
H	-6.21283645495784	4.81542105814679	-2.07969600869129
H	-4.31699827525084	6.07684151633356	2.31123346412015
H	-5.64571996449361	4.04242946321984	2.11532769739642
C	-3.54005773622813	-6.67360689431286	-0.29869192219211
C	-4.98921703978742	-6.49107967954577	-0.31400265815593
C	-2.81047590643201	-6.58407125169855	0.90097350243722
C	-2.82299022862356	-6.90800116597020	-1.48733595944325
C	-1.44862579812402	-7.02034140930853	-1.47916046441881
C	-1.43313944827259	-6.66813441693626	0.90347295003106
C	-0.70861065679726	-6.87571339432161	-0.28764336953664
H	-3.36076938137052	-7.04963893021407	-2.41993953362627
H	-3.33051815345964	-6.37738375681046	1.83156852593410
H	-0.90692309366736	-6.51754900887380	1.83981972190106
C	-5.79776946767577	-6.95718762762648	0.74208700004941
C	-5.61201543325343	-5.83250839445506	-1.39411461917330
C	-6.96803060905079	-5.59438322283131	-1.39857940655308
C	-7.16729844423226	-6.78617123194616	0.71879460040084
C	-7.75177050143654	-6.04818578625043	-0.32133785999663
H	-5.00735230761069	-5.45887127647613	-2.21472006316540
H	-7.43683077979360	-5.06509728269507	-2.22211876814462
H	-5.34627266080625	-7.51041035903009	1.56012460521925
H	-7.79746141172448	-7.19563062666348	1.50221077803853
N	-9.14936735701544	-5.94161120565069	-0.38251694182637
N	-9.80057542301716	-4.94198025429625	-0.66575549986248
C	-9.28579985993546	-3.62640834679573	-0.57100163253888
C	-8.45369605790365	-3.23464303164729	0.48632241189634
C	-9.73343222279889	-2.69217320917226	-1.51140638723465
C	-9.25728311198559	-1.39246164357927	-1.45591538429186
C	-8.03949285083384	-1.91790531911912	0.56524456780642
C	-8.40884193252968	-0.98529243268579	-0.41599293320502
H	-10.42843581952540	-3.00691487695212	-2.28387176281267
H	-9.57877278463123	-0.66779747642163	-2.19836226007951

H	-8.16243176926915	-3.94811346485574	1.25043190938505
H	-7.40116311867704	-1.60223419773168	1.38564971123805
C	-7.89153836702355	0.39600457036051	-0.33660362877697
C	-7.25162036492103	0.97742590645490	-1.43572684945859
C	-7.97770111909083	1.12947149541192	0.85243854098546
C	-7.41528124280684	2.39259823428719	0.94734611205354
C	-6.66833615246270	2.23097113639260	-1.33181527324808
C	-6.72294922239886	2.95924296121129	-0.13368518219710
H	-8.51258319949012	0.71340683252570	1.70193602463110
H	-7.53154481697063	2.95181009251600	1.87056240940969
H	-7.16395789543553	0.41848104150730	-2.36352342102099
H	-6.11974505552662	2.62553927276572	-2.18131748623966
H	-0.94101339521098	-7.25367151030864	-2.40891555592934
H	0.97893050050881	-7.44517096256800	1.79266118549167
H	0.49680902810280	11.00251457292688	-0.94507750622087
H	-0.37519166169719	10.99553722440009	1.69582262167685

128

3CAB-1_cis-cis-trans_trans-2H, Eel(r2SCAN-3c) = -3101.018102089375 a.u.

C	3.63948646389508	-6.29114392354172	-0.30049501140249
C	5.10164348759026	-6.23377897257466	-0.31898746818928
C	2.89331315341078	-6.05929088594382	-1.46910026035672
C	2.93428024621760	-6.56543224647129	0.88454535017311
C	1.55390211779090	-6.60546042559831	0.89878983483644
C	1.51265398570978	-6.06807670615963	-1.44582819756967
C	0.80066172120901	-6.34197167073720	-0.26196746502247
H	3.48122014094196	-6.80447248607965	1.79187701678415
H	3.40705613011634	-5.81066115560254	-2.39316455887199
H	0.97653169398445	-5.81683419181658	-2.35473935784990
C	5.83008087686914	-6.72365704798444	-1.42070562701733
C	5.81572667579699	-5.70024870040088	0.77250630992140
C	7.19029989718791	-5.60013139909263	0.74622655938297
C	7.21017474981632	-6.69886959607128	-1.42760195798996
C	7.89663511374973	-6.07984296129872	-0.37202548524086
H	5.27235147948258	-5.32239768576991	1.63339618827756
H	7.72954044002144	-5.17190822594213	1.58539068809842

H	5.30191830153690	-7.18458448823146	-2.24999928243214
H	7.77684410308357	-7.13115221658780	-2.24653936247160
N	9.29911977580551	-6.11913738530338	-0.39087432502100
N	10.04361148354807	-5.22425922595496	0.01222781692062
C	9.68699642946177	-3.87153206703249	0.12187125607126
C	8.87868633734468	-3.24129180145767	-0.84144717481733
C	10.30704145309030	-3.11195877745395	1.12585876811700
C	10.01836617137802	-1.76672285424982	1.24170003579895
C	8.64598825234614	-1.88771286139117	-0.74771001579196
C	9.17910652770531	-1.12370875148072	0.31088455374289
H	10.99183375344863	-3.60141970617156	1.81146477616130
H	10.48610932372670	-1.18968100557985	2.03352354748573
H	8.45077726674470	-3.81730251588796	-1.65538062037928
H	8.00022656823478	-1.41212611913094	-1.47904755502028
C	8.82894054426354	0.29154569965447	0.43619137635361
C	8.64575727385544	0.88070655057770	1.70020903363840
C	8.58503378337237	1.08172888325219	-0.70105713969610
C	8.10794779981863	2.37178895504068	-0.58117090776128
C	8.13984190406207	2.16077891240165	1.81936162928993
C	7.82191450183183	2.92264166132267	0.68082120064518
H	8.77441101954224	0.67666151978310	-1.69045670628033
H	7.88784839524428	2.94029783273100	-1.47954925060163
H	8.84157532220883	0.30280551332152	2.59847522398764
H	7.98091017663389	2.57820325284172	2.80909930979105
C	5.30199922077272	6.38705977549231	0.73641144308098
C	4.20635506270355	7.33947792533243	0.58091206360942
C	6.34963583252160	6.28005348445536	-0.20172306161420
C	5.24881310368059	5.42262103503119	1.76212014595903
C	6.11968952574143	4.35377062265330	1.78474485856618
C	7.21543877363787	5.20540345528543	-0.18360506534069
C	7.08732246902812	4.18527613231822	0.77903045235515
H	4.45288832689417	5.45667681996322	2.49789930401193
H	5.98641607825784	3.58325684855028	2.53738517030448
H	6.48443700319099	7.05028650442056	-0.95449114964884
H	8.00935875021955	5.15131914921780	-0.92249083288373
C	3.85205722159849	7.82405212117396	-0.69574297865871

C	3.36443574180394	7.67145558146163	1.67228705279824
C	2.15554706660275	8.29005119784545	1.47793989994115
C	2.64676160515394	8.45906745776040	-0.90157270853232
C	1.74660227245226	8.63039039412790	0.16775757591602
H	3.66688917667337	7.41230967597119	2.68169421187696
H	1.48819596638103	8.48858581819802	2.30992129371064
H	4.48245976910328	7.60843349228396	-1.55160950928939
H	2.33090787721683	8.75078532936408	-1.89842824065752
N	0.44278868638474	8.92495264458497	-0.18420913928990
N	-0.39588981453135	9.01083057564323	0.75742905941383
C	-1.71414007022265	8.73686378472411	0.43971773800206
C	-2.13210015453882	8.22757258274134	-0.81018613000180
C	-2.62640508819833	8.77266959214487	1.51402138164429
C	-3.86327531731030	8.18000828702829	1.38783355435605
C	-3.37847031046225	7.66405506366424	-0.93176449608752
C	-4.24032021200633	7.54789082741753	0.18387468375993
H	-2.30667968834202	9.21377608703196	2.45327482137620
H	-4.54405137379400	8.17827470257198	2.23331466555929
H	-1.43710030830228	8.22805162841725	-1.64266394246127
H	-3.66191875300280	7.20736525745012	-1.87507909853318
C	-5.38038176328208	6.64054214646001	0.11465326169195
C	-5.71949040403722	5.86488589663099	1.23956036683375
C	-6.06094153600813	6.37455787635863	-1.09091936211104
C	-6.94498776730371	5.32082813490985	-1.19071349601290
C	-6.58571568306248	4.79787504415024	1.13201879707486
C	-7.18015214973082	4.45847794773964	-0.09913626602908
H	-5.89556755109093	7.01114171742963	-1.95498066268929
H	-7.46066709401762	5.15678077774327	-2.13131064845691
H	-5.21188015931342	6.03534720575726	2.18350871838396
H	-6.73171722325008	4.16166584775783	1.99800609320302
C	-3.50574838602165	-6.24862233442916	-0.20339050553948
C	-4.96731317016836	-6.16962162373908	-0.19378085652982
C	-2.75927043751404	-6.09999559181710	0.97869286889000
C	-2.79842062112207	-6.46191178952881	-1.40033571521639
C	-1.41893797943896	-6.51704867773682	-1.41462983445590
C	-1.37844353867523	-6.12932018358717	0.95894078526735

C	-0.66605822320699	-6.33554800194260	-0.23836736214683
H	-3.34587987529389	-6.63808241105555	-2.32161910772018
H	-3.27246945716031	-5.90092501677316	1.91499798778795
H	-0.84206002074291	-5.94436724174029	1.88367518644689
C	-5.72634582636433	-6.69352684241954	0.86797622023634
C	-5.65985764357559	-5.55096497176053	-1.25075565481933
C	-7.03577458900016	-5.44003765740582	-1.24980112290925
C	-7.10480970731306	-6.61099013790099	0.87243904603927
C	-7.77737963095234	-5.98409833437776	-0.19012028272849
H	-5.09998908153844	-5.11695740858210	-2.07387162718960
H	-7.53928674893230	-4.93632612831938	-2.06798597781286
H	-5.22633414846818	-7.20467656301316	1.68522624521410
H	-7.67159274372072	-7.04268547617093	1.69387485459227
N	-9.16333894816890	-5.94590566223454	-0.18198366324394
N	-9.87769942428983	-5.01500729185149	-0.89428720920459
C	-9.64182067056850	-3.65593549453062	-0.77813024559451
C	-9.03256540697211	-3.11817294548924	0.36594200352460
C	-10.04182217792160	-2.79688245463576	-1.81690251001394
C	-9.81697815800254	-1.43858309810482	-1.71775398470586
C	-8.83608407806077	-1.75671233261850	0.46004359039459
C	-9.20151772431427	-0.88237659630033	-0.58121155131451
H	-10.53123232109953	-3.20334205036078	-2.69868481977654
H	-10.14569432333707	-0.78999839830557	-2.52423675319194
H	-8.70953557899126	-3.76927378212787	1.17090051691355
H	-8.34236689514085	-1.35982951466498	1.34179071094645
C	-8.87135717002677	0.53801267743912	-0.48893011997800
C	-8.44411904866076	1.25782600109503	-1.61911233415635
C	-8.89014526055771	1.21230987963443	0.74751573678495
C	-8.42595464595100	2.50546409174829	0.86121543409826
C	-7.95948855433117	2.54513664155785	-1.50076448238369
C	-7.89442376113955	3.19080033389849	-0.25022583915569
H	-9.29705092145930	0.71477205169542	1.62281733622905
H	-8.48273014086821	2.99836832065304	1.82618213353735
H	-8.40976393505371	0.76696547527472	-2.58711716099973
H	-7.54985886636721	3.02862850021865	-2.38132064118959
H	-0.91771996443856	-6.74274376580229	-2.34997140852311

H 1.05159779530546 -6.88210589048104 1.81969736162098
H -10.34629258346811 -5.35777439018525 -1.72322406374185
H -9.65522635719665 -6.38549208641349 0.58533824489286

128
3CAB-1_cis-trans-trans_cis-2H, Eel(r2SCAN-3c) = -3101.016793920393 a.u.
C 3.16910542875328 -9.09829443303823 -0.02486105969063
C 4.47741773063694 -8.44200364553221 0.07878365534846
C 2.42532530504472 -8.94891376859338 -1.20555066113619
C 2.53486294469289 -9.68760848508615 1.08212852869535
C 1.18377876944497 -9.98797782992756 1.04874063861741
C 1.07124370622533 -9.23552075038179 -1.23161594261154
C 0.40967932581098 -9.69461251688589 -0.08485763414659
H 3.10400992609702 -9.89773047772135 1.98325487333062
H 2.88317663104011 -8.49399332307717 -2.07851828013388
H 0.49972343976558 -8.99882907213457 -2.12389477282263
C 5.32952912165301 -8.29200996632277 -1.03255543597541
C 4.81026861374283 -7.76651735500779 1.27494179627377
C 5.84214310322222 -6.86069963116637 1.31664264355344
C 6.37407141465250 -7.39094921090178 -1.00191677976621
C 6.59946277329052 -6.61406175372218 0.15089223231773
H 4.17666509849273 -7.88905222085419 2.14795492010262
H 6.04043572752003 -6.28152026652877 2.21218869988275
H 5.13885250596336 -8.86133479779966 -1.93698600323046
H 6.99588331032622 -7.22077312720076 -1.87565720642180
N 7.42202578402374 -5.51132775118324 -0.00582853577934
N 7.60799601427376 -4.78275052356573 1.00249357735984
C 7.90449203794777 -3.44747010845805 0.80985827087646
C 7.71552510492788 -2.78068811449347 -0.42049597345253
C 8.20081338378934 -2.69984492100371 1.96763493262046
C 8.19872812466299 -1.32390209788571 1.91852419511239
C 7.73799066651933 -1.40650703446288 -0.46045605542702
C 7.91058656713490 -0.64325263850043 0.71641136956116
H 8.37892854730967 -3.22940505545205 2.89860822831487
H 8.40819425465630 -0.75756127921893 2.82052468710438
H 7.49642545595017 -3.36175751500606 -1.31008327422027

H	7.52722665417006	-0.90184191129089	-1.39854244031897
C	7.64457116161658	0.79198770327795	0.69649053916035
C	7.02362075798897	1.40490034201732	1.80146751153043
C	7.87134967488695	1.58349574660516	-0.44716710935528
C	7.43329930699340	2.89046866567657	-0.50707305394615
C	6.56709306776963	2.70392931042740	1.73190370566726
C	6.72387827993916	3.47434754530715	0.56294085506051
H	8.41807294474657	1.16925543161566	-1.28898694543112
H	7.65258482907978	3.47535251977327	-1.39441738255338
H	6.81413260720109	0.82136115799094	2.69226346761077
H	6.01013181218374	3.10246211669504	2.57289626449822
C	4.57034485618255	7.17688956922910	0.22809600166369
C	3.68224635096411	8.33272843348809	0.12322942672031
C	4.99446984584297	6.47760082519200	-0.91665091090713
C	4.98587105630376	6.68987771023684	1.48255851318905
C	5.73224512811477	5.53529281810766	1.58817667849608
C	5.72449038681396	5.31067522071661	-0.80904703132085
C	6.08837483971020	4.78738479849769	0.44755976807497
H	4.74404305247772	7.24883275315925	2.38160039726092
H	6.06321402788434	5.21586086476749	2.57084603769096
H	4.67903642753915	6.81466020661861	-1.89956321351745
H	5.95753014709658	4.75814421379480	-1.71322064804676
C	3.75702714961584	9.23740944308722	-0.95220673859430
C	2.67950587679852	8.54580813309815	1.08871332515590
C	1.77629413812842	9.58245694908317	0.98086221125868
C	2.87434432237865	10.29265218130137	-1.06289194676052
C	1.86801079511461	10.47531508316880	-0.09802074031632
H	2.58247409607739	7.85375237219790	1.91961491678473
H	0.99770081226774	9.70238769816200	1.72637623915029
H	4.53958030790203	9.12613526826586	-1.69663012535106
H	2.96169818858653	10.98844574436690	-1.89386147279552
N	1.00265010620233	11.54868517211258	-0.22308993377505
N	-0.22318774350164	11.58430269992623	0.38975102631517
C	-1.15885141769679	10.57413115475387	0.24775402025149
C	-1.12495564349136	9.69235490712077	-0.84359423454938
C	-2.17756322169899	10.44616488670763	1.20849881046493

C	-3.12896000981299	9.45440196866109	1.08162520631273
C	-2.09563764163643	8.72046710151894	-0.96738926373917
C	-3.11263458440338	8.56184831851676	-0.00635862665028
H	-2.21976627052650	11.13451455950644	2.04911920579589
H	-3.91903000621770	9.38564458910000	1.82322380960485
H	-0.33833076295969	9.76993185425496	-1.58627077334990
H	-2.04355370754456	8.03512881540747	-1.80783562011056
C	-4.07602098475597	7.46974147453622	-0.12856042080017
C	-4.54895003148255	6.78489663905697	1.00575412089972
C	-4.52003465532043	7.02877712727723	-1.39028372144434
C	-5.34186387245479	5.92841246072724	-1.51301988368630
C	-5.35529315416866	5.67116545641219	0.88089977671394
C	-5.75038793573975	5.19071186689634	-0.38336067989705
H	-4.23890545429537	7.58241639525121	-2.28115275444260
H	-5.69100010097572	5.64536188549508	-2.50055047833173
H	-4.21406347258691	7.08649741938550	1.99373311375445
H	-5.62711150339382	5.12322338694583	1.77700132299574
C	-3.77816643113272	-8.86502975268596	-0.09150020266428
C	-5.03928282651204	-8.12102650836990	-0.18758249189066
C	-3.02655147611065	-8.77893532761078	1.09052134524717
C	-3.18438537884913	-9.48315669167336	-1.20518563139599
C	-1.85647290872521	-9.87369420021813	-1.17564314981892
C	-1.69469798108981	-9.15586703120543	1.11289039177387
C	-1.06491685598038	-9.64543820938563	-0.03913064192917
H	-3.76598732865808	-9.64450188058889	-2.10841378423446
H	-3.45319911937478	-8.30406504026409	1.96866068683848
H	-1.10895204837090	-8.96787978303730	2.00752936476432
C	-5.88033511529993	-7.92668164525645	0.92528507003161
C	-5.32464770752249	-7.41083378208798	-1.37588643604165
C	-6.29275105245877	-6.43677753617161	-1.40723531883174
C	-6.86156793717014	-6.95677432749848	0.90487587636342
C	-7.03267306818375	-6.15296029581867	-0.23877323700130
H	-4.70001663176214	-7.56596229839668	-2.25018111035624
H	-6.45058230771404	-5.83522929948935	-2.29605215497355
H	-5.72937746632280	-8.51795490811721	1.82311621809730
H	-7.47128018244758	-6.75504596491093	1.78042519300143

N	-7.77865210002573	-4.99883279298308	-0.06897459144876
N	-7.91237022819676	-4.24661646607802	-1.06818772338611
C	-8.11817345628949	-2.89686983569825	-0.85869282745775
C	-7.88799312050420	-2.26038962912062	0.38063691622585
C	-8.35982957854184	-2.11601255884296	-2.00718191601513
C	-8.26471086066491	-0.74411328676050	-1.94008645975909
C	-7.81756694254303	-0.88848084250394	0.43853626427425
C	-7.93476089573310	-0.10016282808146	-0.72857640909948
H	-8.57069021213967	-2.62023799093714	-2.94535327555576
H	-8.43265149520957	-0.15325986511179	-2.83502915282104
H	-7.71124087432271	-2.86640657902880	1.26296719045052
H	-7.57584043901661	-0.41134342307499	1.38354146115398
C	-7.57235531123939	1.31343267931494	-0.68916025883555
C	-6.90762509489978	1.89723804693380	-1.78437999291168
C	-7.74892191662912	2.10343785374894	0.46433963079530
C	-7.22365534091960	3.37685047158151	0.54268862298629
C	-6.36442355663501	3.16134520420797	-1.69642544462426
C	-6.47271013640943	3.92524264158590	-0.51773840066266
H	-8.32524965974410	1.71611813676800	1.29920509999993
H	-7.40595155540690	3.96363610602107	1.43711818785142
H	-6.73510406475200	1.31250946807688	-2.68230211078423
H	-5.77885880907162	3.53223236790473	-2.53061518156613
H	-1.41621159803901	-10.33320323319716	-2.05602463378776
H	0.71340988662947	-10.42675169732054	1.92411434757779
H	-0.31203699407953	12.22516251137214	1.16788335212702
H	1.13164452658372	12.19022064091733	-0.99513243805446

128

3CAB-1_cis-trans-trans_trans-2H, Eel(r2SCAN-3c) = -3101.012812133042 a.u.

C	3.50551827501687	-8.34526599627630	0.02816079459393
C	4.89562779943513	-7.90110696762128	0.06037097645763
C	2.78364203652326	-8.36912177370565	-1.17956334055888
C	2.80846510327268	-8.65951248455231	1.21218204971467
C	1.44832060952525	-8.88018096521027	1.20071668536723
C	1.41863508332287	-8.57177756106655	-1.18793834926476
C	0.70198342388349	-8.77935554229139	0.00763401792512

H	3.35260599383027	-8.74416160829161	2.14810525131766
H	3.28637025734613	-8.12922725542247	-2.11153088534054
H	0.88695962570149	-8.47968877309294	-2.12898769443759
C	5.78747887774835	-8.14084966448974	-1.00527001877598
C	5.35517332151115	-7.11568725840419	1.13277907911592
C	6.61042159623395	-6.54272432266694	1.12642949846654
C	7.05940933836696	-7.61050413849612	-1.00861628653258
C	7.47472955261261	-6.77037216056041	0.04258975562756
H	4.68374245663975	-6.88888011133147	1.95496223249379
H	6.90637400620195	-5.88790013580054	1.93829758989603
H	5.48105516528884	-8.77681901882099	-1.83047116785653
H	7.73817718130164	-7.82799939337274	-1.82929796812478
N	8.72913585647348	-6.19163327901808	-0.00919023021107
N	9.11400276333882	-5.24818337821623	0.90901985078535
C	8.67732730879047	-3.93596283847988	0.81088641341143
C	8.02224529551899	-3.46378175487619	-0.33778518155471
C	8.91295896824477	-3.05651605739246	1.88347826350433
C	8.45394788535564	-1.75796222480263	1.82369955411735
C	7.60748915551896	-2.14735299573650	-0.39814566157484
C	7.78960514953508	-1.26675246192851	0.68203571938032
H	9.45045761029106	-3.40403323371480	2.76217650331830
H	8.64185119650165	-1.09403491402690	2.66231812007099
H	7.81415390085304	-4.13270390633991	-1.16560436758088
H	7.07277375869769	-1.80743251937999	-1.27988795019396
C	7.25956252304316	0.09468409904037	0.63285364398686
C	6.69798997666729	0.69375731995014	1.77502611461525
C	7.25340667444648	0.83618266480607	-0.56337017506942
C	6.69800156206527	2.09831308603872	-0.61799017916145
C	6.11868567898615	1.94482895657502	1.71244077507629
C	6.09104968342086	2.68288429247549	0.51226165607579
H	7.73339173675951	0.42892521499803	-1.44841041261793
H	6.76325733378078	2.65380933842297	-1.54745837455511
H	6.65799949483238	0.13811168336992	2.70724197916033
H	5.63349308178530	2.33429783319592	2.60080146655197
C	4.01721166114393	6.44951902559624	0.30739055997940
C	3.24165005455928	7.68720971183724	0.24635560942691

C	4.31367102477577	5.71947511190819	-0.85761547171264
C	4.47025986158098	5.93514440797531	1.53622001578405
C	5.16835979552713	4.74671709587205	1.59716378946457
C	4.98403080588665	4.51448589538834	-0.79126143557555
C	5.42551537645060	3.98607696769903	0.43842308108822
H	4.31310300438881	6.50524500905968	2.44702300170227
H	5.54759208924208	4.41743435859704	2.55851006050145
H	3.95390117346455	6.07414837712509	-1.81899234113610
H	5.12561507118612	3.95044015915357	-1.70677224332681
C	3.40411199704060	8.59878263379856	-0.81556538894824
C	2.31223773249049	7.99842667854594	1.25939431149581
C	1.53910938620858	9.13674433234830	1.19419138973924
C	2.68776081558865	9.77829486890415	-0.85679092443002
C	1.70815064905619	10.02560239127726	0.11650636881098
H	2.16638127785782	7.30619039069726	2.08309497168478
H	0.80991227550561	9.35785108977404	1.96728757291525
H	4.14749586674295	8.40198227134119	-1.58233027185790
H	2.85668538383705	10.51028313643605	-1.64052101052233
N	1.04678582431300	11.26360720605128	0.11409294245448
N	-0.14582123634465	11.45982442882403	0.33058930956692
C	-1.14066349624540	10.46586750386904	0.19475465629941
C	-1.10299988949279	9.51865209804473	-0.83893489773858
C	-2.24532884828738	10.53873022165723	1.05203210179065
C	-3.25183117084793	9.59394229833924	0.94701560455691
C	-2.14509414948637	8.62209879958357	-0.96995203036925
C	-3.22047749312717	8.62246040336557	-0.06574508120049
H	-2.28402509932543	11.32372323929959	1.80107665218009
H	-4.09863557113115	9.63217438223542	1.62607053242741
H	-0.27686099091188	9.50609270186549	-1.54237796859255
H	-2.12065114657893	7.88461438591385	-1.76696547135158
C	-4.27015974218883	7.59512420221873	-0.18987231215876
C	-4.70862601565270	6.88667855983059	0.93444906903600
C	-4.79415365335918	7.24861627944828	-1.44214983482974
C	-5.68034091323225	6.19201696738262	-1.57176076745307
C	-5.57910763710451	5.81530119705989	0.80023128247072
C	-6.05786167669342	5.42679168636218	-0.45865462628272

H	-4.51585800352028	7.82603018364767	-2.31951162101107
H	-6.08870474394612	5.96094948248150	-2.55108445445350
H	-4.30926876828813	7.13257725191194	1.91462418441083
H	-5.83779000831577	5.23329613607677	1.67965050444390
C	-3.52352007865621	-8.10663435532144	-0.06724108670286
C	-4.83765534084776	-7.48236442650431	-0.15866249517358
C	-2.79517651457661	-8.02430053165514	1.13557016745016
C	-2.86442933856472	-8.64478500600155	-1.19258506582484
C	-1.52347992326187	-8.96027312982414	-1.14769043486967
C	-1.44986402719694	-8.32108608723182	1.17331755470836
C	-0.75874590540445	-8.73613191324234	0.01738593480895
H	-3.41866579240688	-8.81897231054004	-2.10989977328764
H	-3.26569834543486	-7.61461361685011	2.02340273148973
H	-0.90598047724489	-8.13441215924556	2.09272602702801
C	-5.68212553202271	-7.35124385916405	0.96582160472884
C	-5.20869254779602	-6.82266457306633	-1.35377923130050
C	-6.26476643021964	-5.94551678646026	-1.38193332275215
C	-6.75786620584587	-6.49295928630090	0.94333539944115
C	-7.02164269366224	-5.72347536227994	-0.20939112576272
H	-4.58796944337522	-6.92827423830375	-2.23814202976545
H	-6.48643582747892	-5.36770190641306	-2.27286337392339
H	-5.46791794321539	-7.91380579642320	1.86902266968462
H	-7.37894339856832	-6.34607449330434	1.82176519989276
N	-7.86015811052097	-4.64024756471183	-0.04532468682382
N	-8.14360306362916	-3.95274670837534	-1.06299544245079
C	-8.42611118256880	-2.61484493662691	-0.87198787675535
C	-8.26351747334580	-1.94653041179616	0.36278469970873
C	-8.68095426424426	-1.86759076416388	-2.03996976957630
C	-8.63518817502494	-0.49105079226341	-1.99966065359079
C	-8.23975547914804	-0.57359363255556	0.39201438824265
C	-8.34355863474407	0.18448096125165	-0.79721163088814
H	-8.84963155518995	-2.39914612631006	-2.97162246006227
H	-8.79759457264476	0.07873458915679	-2.90920428544483
H	-8.09644818059454	-2.52753274636560	1.26351803095854
H	-8.03962770918683	-0.06479624962704	1.33014237506678
C	-7.98616366739441	1.60352232472502	-0.78155882766329

C	-7.23899766488687	2.13368133534626	-1.84671295026513
C	-8.21977199183135	2.42725563528674	0.33342177466925
C	-7.66762206976021	3.69377736271204	0.40833010428675
C	-6.66521968442254	3.38793050221952	-1.75598470153559
C	-6.83303694181376	4.18140996295367	-0.61119461945057
H	-8.85298051583887	2.07453024914341	1.14254905767347
H	-7.88116592749617	4.31495956719665	1.27302259235535
H	-7.02370876777990	1.51619985059490	-2.71337427667322
H	-6.01259739447033	3.72591731617160	-2.55466205405991
H	-1.05438649432255	-9.38098190656216	-2.03114992293500
H	0.95249909039076	-9.13827813130497	2.13072893361015
H	9.34946547675290	-5.61033638876167	1.82407608076229
H	9.22906354805886	-6.17596578149170	-0.88817139592775

128

3CAB-1_all-trans_trans-2H, Eel(r2SCAN-3c) = -3101.008535441152 a.u.

C	4.82644625569431	6.25193293976391	0.51585776600569
C	3.72867957271063	7.22787896689554	0.44580985511849
C	5.06764957349609	5.41671388031417	-0.58372142939706
C	5.55595171993010	6.02625660041352	1.69204288106725
C	6.44848791311650	4.96891331119165	1.78134448825283
C	5.94337758965940	4.34849131069693	-0.48491148783754
C	6.62771054426435	4.08326476741940	0.70854132254634
H	5.43159722321920	6.69281435498698	2.54105175012213
H	4.49985463354552	5.55464992605417	-1.49921146794609
H	6.04313883963578	3.66933218384616	-1.32608497505177
C	3.45024835841838	7.93461778150399	-0.73746885259793
C	2.84249461783658	7.36063731077931	1.53692881548148
C	1.66419105883826	8.05966489131802	1.41465628879258
C	2.27642003992356	8.64917690908839	-0.87048016625969
C	1.34489097857523	8.67208530545559	0.18385662745027
H	3.05651353116309	6.83039822292223	2.46023308310694
H	0.95139958801773	8.10176812830897	2.23151090770293
H	4.15414185890279	7.89566833627096	-1.56312742105859
H	2.02751684635019	9.15677451051715	-1.79743909024500
N	0.08348452741648	9.14848318793732	-0.13683468274730

N	-0.79188012519631	9.13705183034267	0.76345693415079
C	-2.12078052023613	9.05095042899184	0.39525910918224
C	-2.53048861648243	8.62813391896713	-0.88778017481073
C	-3.07702165104399	9.17691247715217	1.42387151871306
C	-4.37563911170056	8.77274418877017	1.20927008570571
C	-3.83364979765728	8.24570046362632	-1.09445729382768
C	-4.76952511987944	8.23099982336312	-0.03427537809529
H	-2.75629959614876	9.54386134207820	2.39411599033590
H	-5.09634689251032	8.84938621789800	2.01723483732229
H	-1.79314242742126	8.54781270636440	-1.67952942815182
H	-4.11740305741491	7.85265169395920	-2.06568524866119
C	-6.01320952490095	7.48699597249551	-0.19793455535788
C	-6.54649961631730	6.76595817656909	0.88868873520448
C	-6.61427682061957	7.29886053694869	-1.46165938870624
C	-7.57390975292984	6.32966020664555	-1.65431870456846
C	-7.49836386094907	5.78820446039413	0.69282657986421
C	-7.97793100566294	5.48476507612648	-0.59749865513929
H	-6.31259443838028	7.91905480246987	-2.30016451255300
H	-8.00888500252466	6.20865045546067	-2.64129310521424
H	-6.12171622288366	6.88976832894436	1.87938775783915
H	-7.79072801462411	5.17365265183786	1.53742835958202
C	-9.41942519609348	1.50961839509951	-1.12774280030602
C	-9.44025086139276	0.05247160355463	-1.18598136202110
C	-8.91509404645805	2.24739703008108	-2.21695237274089
C	-9.70878776630003	2.21721694254980	0.05938750259737
C	-9.35905637503396	3.54213361382575	0.19952894305945
C	-8.55465512989215	3.57008788445603	-2.07202550161313
C	-8.68855776679234	4.23152737693661	-0.83446128306140
H	-10.20647166987208	1.71147895482778	0.88114385226724
H	-9.59075434537758	4.05203335393113	1.12920067598517
H	-8.69031796695262	1.74471318799026	-3.15185091391473
H	-8.05669748216992	4.06457329702483	-2.89908616726024
C	-9.55981267910714	-0.64967419948113	-2.40570409812498
C	-9.13484672015796	-0.68559312326999	-0.01890071136991
C	-8.80825180115279	-2.01832624106496	-0.08510198821339
C	-9.25325485479257	-1.98984463408044	-2.48192835409279

C	-8.81213981077041	-2.67817479082371	-1.33306276644411
H	-9.05597173882597	-0.17266747049504	0.93449874532956
H	-8.48251868559814	-2.55755317041579	0.79814733178282
H	-9.87224003226746	-0.12427981955672	-3.30261592798278
H	-9.29179090982310	-2.52166735641008	-3.42774199337745
N	-8.19582168890185	-3.89840063237401	-1.53383218175793
N	-7.89615270422262	-4.59422221045700	-0.53238123499383
C	-6.83181810643773	-5.47379638130602	-0.65316830204409
C	-5.99077613851600	-5.53915365957186	-1.78465012512670
C	-6.49438354775488	-6.18911723724020	0.51059218742256
C	-5.29009379950174	-6.86049720202131	0.58185442458726
C	-4.79911366930845	-6.22134067190713	-1.70334946503720
C	-4.39324296673090	-6.84005588107528	-0.50089441299861
H	-7.17202524765539	-6.15561432957027	1.35825730286768
H	-5.01999279823194	-7.38803553444835	1.49149702214090
H	-6.26900603588202	-5.00475947519244	-2.68693043382451
H	-4.11922024387403	-6.21318646787432	-2.55018091735393
C	-3.00747443322673	-7.31092292606261	-0.35760247483732
C	-2.30761886002645	-7.02138786608216	0.82186305963625
C	-2.30893061128745	-7.91949811865168	-1.41029574993250
C	-0.94983623960154	-8.17381691555023	-1.30630212943337
C	-0.94607605954900	-7.25649453663118	0.91508450603615
C	-0.23302842063842	-7.80789761041894	-0.15754316999945
H	-2.83801572332974	-8.20724525672889	-2.31465116165416
H	-0.43679325848769	-8.66205767664451	-2.12980435416422
H	-2.81878584669840	-6.52894039362480	1.64389882772913
H	-0.41697797986581	-6.94183365202499	1.80941857693600
C	8.60184989818433	0.27966020692689	1.00951869995557
C	9.05165751276648	-1.11569820122738	1.05622083547789
C	8.01267813866572	0.87756225534255	2.13459194447499
C	8.65525946524735	1.02334989314727	-0.18089389742139
C	8.08592277811214	2.28196275954850	-0.25879440298655
C	7.42238466100522	2.12756429957523	2.04731882879957
C	7.41606178500035	2.84170457928950	0.84063514697514
H	9.16706538460841	0.61431547279624	-1.04749887368893
H	7.94341974018991	0.32169811559239	3.06532192790786

H	6.89789495187278	2.52576832014712	2.91074217990934
C	9.66278547747326	-1.66690884256427	2.19990332912597
C	8.79751335764805	-1.97190848044520	-0.02840695273413
C	9.08076383382521	-3.32252714970485	0.03207016349276
C	9.98435551837421	-3.00651004131561	2.26540644013654
C	9.66238772877219	-3.85887604933245	1.19241061645143
H	8.30300909149024	-1.57903082184222	-0.91142610399481
H	8.81037762446820	-3.96640373956287	-0.79749868664629
H	9.90629112059993	-1.02280153776637	3.03970691085258
H	10.47344826969531	-3.40809026200731	3.14926431064441
N	9.92550138403070	-5.21558208481605	1.30691331773598
N	9.48978676020869	-6.10156996584609	0.35681535555877
C	8.17327666288507	-6.53658125864663	0.33641026261708
C	7.27994244342825	-6.21096240633641	1.37002250212940
C	7.72742609507064	-7.31820380729124	-0.74611067694351
C	6.40368775727261	-7.69810426035651	-0.82079392882685
C	5.96584214008662	-6.62977452716972	1.29439601348744
C	5.48537187014381	-7.35561871482023	0.19150701250749
H	8.42411150243712	-7.60954061799291	-1.52804776214857
H	6.07059507032957	-8.29127998299750	-1.66733846616757
H	7.60111623387611	-5.59549970235960	2.20303397602657
H	5.27418498652998	-6.32943321316725	2.07542376993745
C	4.05376086843582	-7.65385142580180	0.07771834643297
C	3.39398492940987	-7.52654105361153	-1.15471728306629
C	3.28442402413808	-7.97731379200920	1.20757714713910
C	1.90936389719663	-8.10243470833053	1.11911916584027
C	2.01503607456965	-7.62854399864025	-1.23599626413469
C	1.24026966817794	-7.88451062822248	-0.09563824521441
H	3.77610655373948	-8.15107240089135	2.16063167455040
H	1.34273267770443	-8.37529869505405	2.00451553321262
H	3.95996987906553	-7.26255249513055	-2.04354382879920
H	1.52620740811992	-7.43830035619378	-2.18679131824691
H	8.16199959480960	2.84298052940122	-1.18558122228926
H	7.01338953339367	4.82727949625750	2.69817577068253
H	10.05761766126487	-5.60717547825754	2.23065100613120
H	10.01039910900499	-6.09130901262387	-0.51089755490076

S24. References

(1) Tsuchido, Y.; Abe, R.; Ide, T.; Osakada, K. *Angew. Chem. Int. Ed.* **2020**, *59*, 22928–22932.

(2) Carle, M. S.; Shimokura, G. K.; Murphy, G. K. Eur. *J. Org. Chem.* **2016**, 3930–3933.

(3) (a) Sheldrick, G. M. *Acta Cryst.* **2015**, *A71*, 3–8. (b) Sheldrick, G. M. *Acta Cryst.* **2015**, *C71*, 3–8.

(4) Dolomanov, V.; Bourhis, L. J.; Gildea, R. J.; Howard, J. A. K.; Puschmann, H. *J. Appl. Cryst.* **2009**, *42*, 339–341.

(5) Babaei, M.; Clement, J. A.; Dayal, K.; Shankar, M. R. *RSC Adv.* **2017**, *7*, 52510–52516.

(6) Zhang, J.; Wang, L.; Li, N.; Liu, J.; Zhang, W.; Zhang, Z.; Zhou, N.; Zhu, X. *CrystEngComm* **2014**, *16*, 6547–6551.

(7) Neese, F. *WIREs Comput. Mol. Sci.* **2018**, *8*, e1327.

(8) Neese, F.; Wennmohs, F.; Becker, U.; Riplinger, C. *J. Chem. Phys.* **2020**, *152*, 224108.

(9) Grimme, S.; Hansen, A.; Ehlert, S.; Mewes, J. M. *J. Chem. Phys.* **2021**, *154*, 064103.

(10) Mardirossian, N.; Head-Gordon, M. *Phys. Chem. Chem. Phys.* **2014**, *16*, 9904–9924.

(11) Weigend, F.; Ahlrichs, R. *Phys. Chem. Chem. Phys.* **2005**, *7*, 3297–3305.

(12) Casanova-Páez, M.; Goerigk, L. *J. Chem. Theory Comput.* **2021**, *17*, 5165–5186.

(13) Dunning Jr. T. H. *J. Chem. Phys.* **1989**, *90*, 1007–1023.

(14) Yanai, T.; Tew, D. P.; Handy, N. C. *Chem. Phys. Lett.* **2004**, *393*, 51–57.

(15) Perdew, J. P.; Ruzsinszky, A.; Csonka, G. I.; Constantin, L. A.; Sun, J. *Phys. Rev. Lett.* **2009**, *103*, 026403.

(16) Helmich-Paris, B.; de Souza, B.; Neese, F.; Izsák, R. *J. Chem. Phys.* **2021**, *155*, 104109.

(17) Weigend, F. *Phys. Chem. Chem. Phys.* **2006**, *8*, 1057–1065.

(18) (a) Weigend, F.; Köhn, A.; Hättig, C. *J. Chem. Phys.* **2002**, *116*, 3175–3183. (b) Haettig, C. *Phys. Chem. Chem. Phys.* **2005**, *7*, 59–66.

(19) A. V. Marenich, C. J. Cramer, D. G. Truhlar, *J. Phys. Chem. B* **2009**, *113*, 6378–6396.

(20) J. Zheng, X. Xu, D. G. Truhlar, *Theor. Chem. Acc.* **2011**, *128*, 295–305.

(21) Allouche, A.-R. *J. Comput. Chem.* **2011**, *32* 174–182.

(22) The PyMOL Molecular Graphics System, Version 2.5.0 Schrödinger, LLC.

(23) Żuchowski, P. S.; Podeszwa, R.; Moszyński, R.; Jeziorski, B.; Szalewicz, K. *J. Chem. Phys.* **2008**, *129*, 084101.

(24) Papajak, E.; Zheng, J.; Xu, X.; Leverentz, H. R.; Truhlar, D. G. *J. Chem. Theory Comput.* **2011**, *7*, 3027–3034.

(25) Parker, T. M.; Burns, L. A.; Parrish, R. M.; Ryno, A. G.; Sherrill, C. D. *J. Chem. Phys.* **2014**, *140*, 094106.
(26) Smith, D. G. A.; Burns, L. A.; Simmonett, A. C.; Parrish, R. M.; Schieber, M. C.; Galvelis, R.; Kraus, P.; Kruse, H.; Di Remigio, R.; Alenaizan, A.; James, A. M.; Lehtola, S.; Misiewicz, J. P.; Scheurer, M.; Shaw, R. A.; Schriber, J. B.; Xie, Y.; Glick, Z. L.; Sirianni, D. A.; O'Brien, J. S.; Waldrop, J. M.; Kumar, A.; Hohenstein, E. G.; Pritchard, B. P.; Brooks, B. R.; Schaefer III, H. F.; Yu. Sokolov, A.; Patkowski, K.; DePrince III, A. E.; Bozkaya, U.; King, R. A.; Evangelista, F. A.; Turney, J. M.; Crawford, T. D.; Sherrill, C. D. *J. Chem. Phys.* **2020**, *152*, 184108.
